# Supplementary material for: Comparison of efficacy and safety of different anticoagulation regimens in plasma exchange: A systematic review and meta-analysis
Source: PLoS One. 2024 Oct 24;19(10):e0311603. doi: 10.1371/journal.pone.0311603 (PMC11500872; doi:10.1371/journal.pone.0311603)
Supplement: S1 File — (DOCX) [file pone.0311603.s001.docx]

**TableS1 Literature search strategies.**

| Database | Strategies |
| --- | --- |
| Medline | ((plasma apheresis) OR plasmapheresis OR (plasma exchange)) AND (anticoagulation OR heparin OR citrate OR nafamostat OR argatroban) |
| Embase | 1. plasmapheresis.mp.  2. plasma apheresis.mp.  3. plasma exchange.mp.  4. anticoagulation.mp.  5. nafamostat.mp.  6. argatroban.mp.  7. heparin.mp.  8. citrate.mp.  9. 4 OR 5 OR 6 OR 7 OR 8  10. 1 OR 2 OR 3  11. 9 AND 10 |
| Cochrane Central Library | 1. plasmapheresis.mp.  2. plasma apheresis.mp.  3. plasma exchange.mp.  4. anticoagulation.mp.  5. nafamostat.mp.  6. argatroban.mp.  7. heparin.mp.  8. citrate.mp.  9. 4 OR 5 OR 6 OR 7 OR 8  10. 1 OR 2 OR 3  11. 9 AND 10 |
| CNKI | ((plasma apheresis) OR plasmapheresis OR (plasma exchange)) AND (anticoagulation OR heparin OR citrate OR nafamostat OR argatroban) |
