# Supplementary material for: Comparison of efficacy and safety of different anticoagulation regimens in plasma exchange: A systematic review and meta-analysis
Source: PLoS One. 2024 Oct 24;19(10):e0311603. doi: 10.1371/journal.pone.0311603 (PMC11500872; doi:10.1371/journal.pone.0311603)
Supplement: S2 File — (DOCX) [file pone.0311603.s002.docx]

Table S2. The data extracted from the studies included in this systematic review that would be needed to replicate this meta-analysis

| Author/Year | Region | Study Design | Population, n | Age | Outcomes | | | | Name of data extractors | Date of data extraction |
| --- | --- | --- | --- | --- | --- | --- | --- | --- | --- | --- |
|  |  |  |  |  | Extracorporeal circuit clotting (Event/Total) | Bleeding events (Event/Total) | APTT (Mean ± SD) | Platelet counts (Mean ± SD) |  |  |
| Brunetta, 2017 | Croatia | Respective observation | 1140 | NR | UFH:183/7733; Saline: 75/1193; LMWH: 69/1193 | UFH: 9/7733; Saline: 2/1193; LMWH: 2/1193 | NR | NR | R.S. and H.L.M. | 13nd May, 2023 |
| Yuan, 2018 | China | Prospective randomized trial | 164 | Median: 45 | NR | UFH: 23/168; Saline: 9/230 | UFH: 104.1 ± 41.3; Saline: 60.6 ± 24.8 | UFH: 81.8 ± 45.7; Saline: 84.9 ± 29.2 | R.S. and H.L.M. | 13nd May, 2023 |
| Yuan, 2020 | China | Respective observation | 85 | Mean: 54.0 | UFH: 9/120; RCA: 0/93; Saline: 14/42 | UFH: 4/120; RCA: 0/93; Saline: 0/42 | UFH: 48.7 ± 3.6; RCA: 40.1 ± 6.7; Saline: 41 ± 5.3 | UFH: 98 ± 11.2; Saline: 99 ± 9.7 | R.S. and H.L.M. | 13nd May, 2023 |
| Teh S, 2022 | Singapore | Retrospective cohort study | 23 | NR | UFH: 3/50; RCA: 10/62 | UFH: 0/50; RCA: 3/62 | NR | NR | R.S. and H.L.M. | 13nd May, 2023 |
| Ma, 2019 | China | Prospective nonrandomized controlled trial | 52 | NR | UFH: 20/94; RCA: 16/106 | UFH: 3/84; RCA: 0/106 | UFH: 162.7 ± 27.2; RCA: 122.5+29.3 | NR | R.S. and H.L.M. | 13nd May, 2023 |
| Zhang, 2022 | China | Respective observation | 62 | Mean: 50.0 | UFH:5/62; LMWH: 0/21 | UFH: 3/62; LMWH: 1/21 | UFH: 61.37 ± 38.74; LMWH: 49.38 ± 16.93 | UFH: 110.58 ± 57.06; LMWH: 128.42 ± 72.83 | R.S. and H.L.M. | 13nd May, 2023 |
| Pan, 2015 | China | Respective observation | 112 | Mean: 39.0 | UFH:19/108; LMWH: 3/264 | UFH: 32/108; LMWH: 0/264 | UFH: 71.67 ± 22.47; LMWH: 62.15 ± 23.32 | UFH: 88.76 ± 26.7; LMWH: 114.4 ± 17.2 | R.S. and H.L.M. | 13nd May, 2023 |

Note: UFH: unfractionated heparin; RCA: regional citrate acid; LMWH: low molecular weight heparin
