# Supplementary material for: Comparison of efficacy and safety of different anticoagulation regimens in plasma exchange: A systematic review and meta-analysis
Source: PLoS One. 2024 Oct 24;19(10):e0311603. doi: 10.1371/journal.pone.0311603 (PMC11500872; doi:10.1371/journal.pone.0311603)
Supplement: S3 File — (DOCX) [file pone.0311603.s003.docx]

In total, 5412 records were identified after removing duplicates, among which 754 records were removed for being studies before 1990 [1-754], 415 records were removed for being non-human studies [755-1169], 227 records were removed as being pediatric studies [1170-1396], 115 records were removed for being neither Chinese or English[1397-1511], 738 were removed for being case reports[1512-2249], 25 were removed for being commentaries [2250-2274], 5 were removed for being protocols [2275-2279], 590 were removed for being reviews [2280-2869], 2125 records were removed for being studies irrelevant to plasma exchange (PE) [2870-4994], 311 records were removed for as being PE studies irrelevant to anticoagulation[4995-5305], 3 records were removed for being PE study without any anticoagulation agent [5306-5308], 9 records were removed for being PE studies which exclusively used heparin or low molecular weight heparin[5309-5317], 37 records were removed for being PE studies which exclusively used citrate acid sodium [5318-5354], 4 records were removed for being PE studies which exclusively used nafamostat [5355-5358], 17 records were removed for having no comparison of anticoagulation agents [5359-5375], and 30 records were removed for lacking of sufficient information[5376-5405], leaving 7 studies to be included in the final analysis [5406-5412].

**References**

1. Abdullaev, O.M., et al., *[Effectiveness of plasmapheresis on the PF-0.5 blood separator in patients with paraproteinemic hemoblastoses].* Ter Arkh, 1987. **59**(10): p. 77-81.

2. Achenbach, H., et al., *[Discontinuous plasma exchange with the conventional hemodialysis technic and blood cell centrifugation].* Z Urol Nephrol, 1983. **76**(7): p. 465-72.

3. Adams, M.B., et al., *Plasmapheresis in the treatment of renal allograft rejection.* Proc Clin Dial Transplant Forum, 1979. **9**: p. 252-5.

4. Adams, M.B., et al., *Plasmapheresis in the treatment of renal allograft rejection.* Proceedings of the Clinical Dialysis and Transplant Forum, 1979. **9**: p. 252-255.

5. Ahlborg, G. and L. Hagenfeldt, *Effect of heparin on the substrate utilization during prolonged exercise.* Scand J Clin Lab Invest, 1977. **37**(7): p. 619-24.

6. Ahmad, S.S., et al., *Rapid purification of factor IX, factor X and prothrombin by immunoaffinity and ion exchange chromatography.* Thromb Res, 1989. **55**(1): p. 121-33.

7. Aiach, M., et al., *Antithrombin III Avranches, a new variant with defective serine-protease inhibition--comparison with antithrombin III Charleville.* Thromb Haemost, 1988. **60**(1): p. 94-6.

8. Albers, J.J., et al., *Isolation and characterization of human plasma lipid transfer proteins.* Arteriosclerosis, 1984. **4**(1): p. 49-58.

9. Albers, J.W. and J.J. Kelly Jr, *Acquired inflammatory demyelinating polyneuropathies: Clinical and electrodiagnostic features.* Muscle and Nerve, 1989. **12**(6): p. 435-451.

10. Anderson, D.F. and J.J. Faber, *Water flux due to colloid osmotic pressures across the haemochorial placenta of the guinea-pig.* J Physiol, 1982. **332**: p. 521-7.

11. Anderson, L. and F.A. Ziter, *Plasmapheresis via central catheter in dermatomyositis: a new method for selected pediatric patients.* J Pediatr, 1981. **98**(2): p. 240-1.

12. Andrew, M., et al., *A low molecular weight heparin alters the fetal coagulation system in the pregnant sheep.* Thromb Haemost, 1986. **55**(3): p. 342-6.

13. Anghileri, L.J., *The binding of iron to plasma proteins-the effects of chelating agents.* Int Z Klin Pharmakol Ther Toxikol, 1968. **1**(6): p. 523-8.

14. Anghileri, L.J., *Interaction of ethylenediaminetetraacetic acid with plasma proteins. II. Its effects on the binding of indium and chromium.* Int Z Klin Pharmakol Ther Toxikol, 1969. **2**(3): p. 253-8.

15. Anonymous, *Approach to the treatment of glomerulonephritis.* Japanese Journal of Nephrology, 1982. **24**(7): p. 803-813.

16. Antwiler, G.D., P.C. Dau, and D.D. Lobdell, *Treatment of hypercholesterolemia by precipitation of lipoproteins with dextran sulfate.* J Clin Apher, 1988. **4**(2-3): p. 72-7.

17. Arisz, L., G.A. Andres, and J.R. Brentjens, *The morphological basis of the glomerular permeability to proteins.* Ric Clin Lab, 1977. **7**(4): p. 312-27.

18. Armstrong, V.W., et al., *Effect of HELP-LDL-apheresis on serum concentrations of human lipoprotein(a): kinetic analysis of the post-treatment return to baseline levels.* Eur J Clin Invest, 1989. **19**(3): p. 235-40.

19. Armstrong, V.W., et al., *Selective continuous extracorporal elimination of low-density lipoproteins with heparin at acidic pH.* Transactions - American Society for Artificial Internal Organs, 1983. **29**: p. 323-328.

20. Armstrong, V.W., et al., *Selective continuous extracorporal elimination of low-density lipoproteins with heparin at acidic pH.* Trans Am Soc Artif Intern Organs, 1983. **29**: p. 323-8.

21. Arnau de Bolós, J.M., C. Pigrau Serrallach, and J. Bosch Gil, *[Thrombocytopenic thrombotic purpura: pathogenic and therapeutic advances].* Med Clin (Barc), 1982. **78**(3): p. 113-7.

22. Asaba, H., et al., *Clinical trial of plasma exchange with a membrane filter in treatment of crescentic glomerulonephritis.* Clinical Nephrology, 1980. **14**(2): p. 60-65.

23. Asaba, H., et al., *Clinical trial of plasma exchange with a membrane filter in treatment of crescentic glomerulonephritis.* Clin Nephrol, 1980. **14**(2): p. 60-5.

24. Ascari, E., E. Bobbio Pallavicini, and R. Adami, *Thrombotic thrombocytopenic purpura (TTP) treatment: Italian cooperative retrospective study on 29 cases.* Haematologica, 1986. **71**(1): p. 39-43.

25. Asmal, A.C., et al., *The effects of sodium pentosan polysulphate on peripheral metabolism.* S Afr Med J, 1975. **49**(27): p. 1091-4.

26. Assogba, U., et al., *[Plasma exchange with very low molecular weight heparin CY 222. Biological profile and therapeutic value].* Ann Med Interne (Paris), 1988. **139 Suppl 1**: p. 66-8.

27. Assogba, U., et al., *Plasma exchange combined with a very low molecular weight heparin CY 222. Biologic profile and therapeutic interest.* Annales de Medecine Interne, 1988. **139**(SUPPL. 1): p. 66-68.

28. Athari, F., L. Feige, and C.B. Cook, *Apheresis in a community hospital: six years' experience.* South Med J, 1987. **80**(4): p. 454-8.

29. Aubert, I., et al., *[Removal of low density lipoproteins on dextrans sulfate in 2 patients with familial monogenic hypercholesterolemia].* Ann Med Interne (Paris), 1988. **139 Suppl 1**: p. 72-6.

30. Aul, C., et al., *[Differential therapy of thrombotic-thrombopenic purpura. Administration of fresh plasma versus plasma separation].* Dtsch Med Wochenschr, 1984. **109**(50): p. 1922-6.

31. Bagger, J.P., T.T. Nielsen, and P. Henningsen, *Myocardial exchange of metabolites after nitroglycerin in patients with coronary artery disease.* Int J Cardiol, 1984. **5**(5): p. 599-612.

32. Baggio, G., et al., *LDL-apheresis with the HELP system: a 16-month study in severe hypercholesterolemic patients with ischemic heart disease.* Beitr Infusionsther, 1988. **23**: p. 146-51.

33. Bahmer, F.A., R. Bambauer, and D. Stenger, *Penicillamine-induced pemphigus foliaceus-like dermatosis: A case with unusual features, successfully treated by plasmapheresis.* Archives of Dermatology, 1985. **121**(5): p. 665-668.

34. Bajaj, S.P., et al., *Heterogeneity in human prothrombin: analysis of cause.* Blood, 1981. **58**(5): p. 886-91.

35. Bambauer, R., W. Cremer, and G.A. Jutzler, *Treatment concepts for acute renal failure based on etiopathological aspects.* Nieren- und Hochdruckkrankheiten, 1983. **12**(8): p. 310-318.

36. Barbero-Mari, P., L. Garcia-Marcos, and C. Sanchez Lopez, *Actual concepts on the management of the hyperviscosity syndrome.* Acta Pediatrica Espanola, 1985. **43**(6): p. 189-192.

37. Barkagan, Z.S., et al., *[Experience in the use of staged plasmapheresis for preventing fetal loss in women with lupus-type anticoagulants circulating in their blood].* Ter Arkh, 1989. **61**(7): p. 124-8.

38. Barkagan, Z.S. and N. Shoĭkhet Ia, *[Substantiation, tactics and effectiveness of frozen plasma-enzyme inhibitor therapy in septicemia and infectious-destructive processes].* Gematol Transfuziol, 1989. **34**(10): p. 8-12.

39. Barkagan, Z.S. and I.N. Shoikhet, *Substantiation, tactics and effectiveness of frozen plasma-enzyme inhibitor therapy in septicemia and infectious-destructive processes.* Gematologiia i transfuziologiia, 1989. **34**(10): p. 8-12.

40. Barkagan, Z.S., O.A. Tsiguleva, and I.S. Abelovich, *[Complex intensive therapy of terminal disseminated intravascular coagulation and thromboembolic syndrome in Marchiafava-Micheli anemia].* Gematol Transfuziol, 1987. **32**(9): p. 42-4.

41. Barrett, J.D., P. Eggena, and M.P. Sambhi, *Extraction and measurement of circulating angiotensins I and II.* Clin Chem, 1977. **23**(3): p. 464-8.

42. Barth, P., B. Kommerell, and U. Beckmann, *[Isolation of the contact activation product of the blood coagulation system from pig plasma].* Thromb Diath Haemorrh, 1969. **21**(3): p. 500-7.

43. Bartolo, M., *[Hemodilution, defibrinogenation and plasmapheresis in hemorheology].* Ric Clin Lab, 1985. **15 Suppl 1**: p. 439-54.

44. Becker, G.J., et al., *Plasmapheresis in the treatment of glomerulonephritis.* Med J Aust, 1977. **2**(21): p. 693-6.

45. Beckerhoff, R., et al., *Problems connected with plasma renin activity measurements by angiotensin I radioimmunoassay.* Horm Metab Res, 1975. **7**(4): p. 342-7.

46. Benarous, R., D. Labie, and F. Josso, *Human prothrombin: a new method of preparation from a single individual.* Thromb Diath Haemorrh, 1973. **30**(3): p. 425-36.

47. Benayahu, D. and M. Aronson, *Comparative study of protamine chloride and sulphate in relation to the heparin rebound phenomenon.* Thromb Res, 1983. **32**(2): p. 109-14.

48. Benichou, J.J. and B. Labrune, *Kawasaki disease. Current aspects.* Annales de Pediatrie, 1988. **35**(5): p. 297-301.

49. Bensinger, W.I., C.D. Buckner, and R.A. Clift, *Whole blood immunoadsorption of anti-A or anti-B antibodies.* Vox Sang, 1985. **48**(6): p. 357-61.

50. Berg, D., et al., *[Studies on exchange transfusion. II. Comparative studies on the behavior of pH, pC02, pO2 and base excess during the use of heparin and ACD blood].* Monatsschr Kinderheilkd (1902), 1967. **115**(8): p. 448-52.

51. Bergstein, J.M., *Anticoagulant therapy in human renal disease.* Int J Pediatr Nephrol, 1981. **2**(1): p. 1-8.

52. Bernhardt, W. and A. Nováková-Banet, *Antithrombin III concentrates in intensive care.* Ric Clin Lab, 1983. **13**(1): p. 61-6.

53. Berning, T., et al., *[Plasma perfusion in life-threatening exogenous poisoning].* Schweiz Med Wochenschr, 1987. **117**(37): p. 1368-73.

54. Bernstein, M.L., B.K. Schneider, and J.L. Naiman, *Plasma exchange in refractory acute autoimmune hemolytic anemia.* Journal of Pediatrics, 1981. **98**(5): p. 774-775.

55. Berr, F., R. Eckel, and F. Kern, Jr., *Plasma decay of chylomicron remnants is not affected by heparin-stimulated plasma lipolytic activity in normal fasting man.* J Lipid Res, 1985. **26**(7): p. 852-9.

56. Berr, F., R. Eckel, and F. Kern Jr, *Plasma decay of chylomicron remnants is not affected by heparin-stimulated plasma lipolytic activity in normal fasting man.* Journal of Lipid Research, 1985. **26**(7): p. 852-859.

57. Bieri, J.G., R.P. Evarts, and S. Thorp, *Factors affecting the exchange of tocopherol between red blood cells and plasma.* Am J Clin Nutr, 1977. **30**(5): p. 686-90.

58. Biggs, J.T., Jr., et al., *Renal and biliary disposition of dapsone in the dog.* Antimicrob Agents Chemother, 1975. **7**(6): p. 816-24.

59. Birnstingl, M., *Raynaud's syndrome: Diagnosis and management.* British Journal of Hospital Medicine, 1979. **21**(6): p. 602-611.

60. Biro, G.P., *Fluorocarbon and dextran hemodilution in myocardial ischemia.* Can J Surg, 1983. **26**(2): p. 163-8.

61. Blache, D., D. Bouthillier, and J. Davignon, *Simple, reproducible procedure for selective measurement of lipoprotein lipase and hepatic lipase.* Clin Chem, 1983. **29**(1): p. 154-8.

62. Blumenstein, M., B. Schmidt, and T. Bosch, *Reduced complement activation in membrane plasma filtration with citrate anticoagulation.* Life Support Systems, 1986. **4**(SUPPL. 2): p. 204-206.

63. Bock, G.H. and R.L. Vernier, *Immunologic kidney disease.* Am Fam Physician, 1980. **22**(5): p. 87-96.

64. Bönner, G., et al., *Measurement of plasma prokallikrein independent of inhibitors and interfering enzymes.* Adv Exp Med Biol, 1986. **198 Pt B**: p. 87-98.

65. Bookchin, R.M., et al., *Dehydration and delayed proton equilibria of red blood cells suspended in isosmotic phosphate buffers. Implications for studies of sickled cells.* J Lab Clin Med, 1984. **104**(6): p. 855-66.

66. Borisova, A.M., et al., *[Clinical efficiency of various intermittent plasmapheresis regimens in patients with rheumatoid arthritis (according to data of two clinics].* Ter Arkh, 1989. **61**(6): p. 82-5.

67. Boroń, P., et al., *[Critical analysis of complex therapeutic methods in acute hepatic insufficiency with encephalopathy in the course of viral hepatitis].* Pol Arch Med Wewn, 1980. **63**(4): p. 327-33.

68. Bouvier, J.L., et al., *[Thrombopenia caused by heparin. Treatment by plasmapheresis].* Presse Med, 1986. **15**(42): p. 2115.

69. Bouvier, J.L., et al., *Treatment of serious heparin-induced thrombocytopenia by plasma exchange: report on 4 cases.* Thromb Res, 1988. **51**(3): p. 335-6.

70. Bovill, E.G., et al., *A human myeloma-produced monoclonal protein directed against the active subpopulation of von Willebrand factor.* Am J Clin Pathol, 1986. **85**(1): p. 115-23.

71. Boyle, P.D., et al., *Multiple-site avascular necrosis. A complication of Goodpasture's syndrome treated with plasma exchange and corticosteroids.* Medical Journal of Australia, 1982. **2**(9): p. 437-438.

72. Branson, H.E., et al., *Trials of commercial reagents in anion-exchange coagulation procedures: ortho diagnostics.* Am J Clin Pathol, 1984. **82**(4): p. 432-5.

73. Bray, B.A., *Quantification of tissue fibronectin from terminal villi of placenta.* Biochem J, 1985. **226**(3): p. 811-5.

74. Breckenridge Jr, R.L., et al., *Treatment of thrombotic thrombocytopenic purpura with plasma exchange, antiplatelet agents, corticosteroid, and plasma infusion: Mayo clinic experience.* Journal of Clinical Apheresis, 1982. **1**(1): p. 6-13.

75. Brenner, T. and E. Shafrir, *Lipoprotein lipid and protein synthesis in experimental nephrosis and plasmapheresis: II. Perfused rat liver.* Lipids, 1980. **15**(9): p. 637-43.

76. Briginshaw, G.F. and J.N. Shanberge, *Identification of two distinct heparin cofactors in human plasma. Separation and partial purification.* Arch Biochem Biophys, 1974. **161**(2): p. 683-90.

77. Broze, G.J., Jr. and J.P. Miletich, *Human Protein Z.* J Clin Invest, 1984. **73**(4): p. 933-8.

78. Broze, G.J., Jr. and J.P. Miletich, *Isolation of the tissue factor inhibitor produced by HepG2 hepatoma cells.* Proc Natl Acad Sci U S A, 1987. **84**(7): p. 1886-90.

79. Brunner, G., *[Acute liver failure--therapeutic aspects 1982].* Fortschr Med, 1982. **100**(27-28): p. 1290-4.

80. Bucher, U., *The use of blood components in the treatment of haemorrhage.* Wiener Klinische Wochenschrift, 1979. **91**(12): p. 408-414.

81. Buchholz, D.H., et al., *Extended storage of single-donor platelet concentrate collected by a blood cell separator.* Transfusion, 1985. **25**(6): p. 557-62.

82. Buckner, C.D. and R. Eisel, *Plasma exchange iransfusion for hepatic coma: new technic.* Transfusion, 1970. **10**(1): p. 26-32.

83. Buckner, D., et al., *Leukapheresis by continuous flow centrifugation (CFC) in patients with chronic myelocytic leukemia (CML).* Blood, 1969. **33**(2): p. 353-369.

84. Buckner, D., et al., *Leukapheresis by continuous flow centrifugation (CFC) in patients with chronic myelocytic leukemia (CML).* Blood, 1969. **33**(2): p. 353-69.

85. Buffaloe, G.W., R.R. Erickson, and P.C. Dau, *Evaluation of a parallel plate membrane plasma exchange system.* J Clin Apher, 1983. **1**(2): p. 86-94.

86. Bukowski, R.M., et al., *Therapy of thrombotic thrombocytopenic purpura: an overview.* Semin Thromb Hemost, 1981. **7**(1): p. 1-8.

87. Bukowski, R.M., J.W. King, and J.S. Hewlett, *Plasmapheresis in the treatment of thrombotic thrombocytopenic purpura.* Blood, 1977. **50**(3): p. 413-7.

88. Burge, J., A. Nicholson-Weller, and K.F. Austen, *Isolation of C4-binding protein from guinea pig plasma and demonstration of its function as a control protein of the classical complement pathway C3 convertase.* J Immunol, 1981. **126**(1): p. 232-5.

89. Burgstaler, E.A., A.A. Pineda, and R.D. Ellefson, *Removal of plasma lipoproteins from circulating blood with a heparin-agarose column.* Mayo Clin Proc, 1980. **55**(3): p. 180-4.

90. Burhop, K.E., et al., *Effect of heparin on increased pulmonary microvascular permeability after bone marrow embolism in awake sheep.* Am Rev Respir Dis, 1987. **136**(1): p. 134-41.

91. Burka, E.R. and T.A. Lane, *Optimal conditions for in vitro carbamylation of sickle cell (SS) RBC.* Clinical Research, 1975. **23**(3): p. 401A.

92. Burka, E.R., T. Puffer, and J. Martinez, *The influence of donor characteristics and preparation methods on the potency of human cryoprecipitate.* Transfusion, 1975. **15**(4): p. 323-8.

93. Burke, P.E., et al., *Activity and transport of antithrombin during acute limb ischemia.* J Vasc Surg, 1989. **9**(5): p. 740-6.

94. Burnouf, T., et al., *Properties of a highly purified human plasma factor IX:c therapeutic concentrate prepared by conventional chromatography.* Vox Sang, 1989. **57**(4): p. 225-32.

95. Burrowes, C.E. and H.Z. Movat, *Isolation of antithrombin III from human plasma: its separation from alpha1-antitrypsin1.* Biochem Biophys Res Commun, 1977. **74**(1): p. 140-9.

96. Buselmeier, T.J., et al., *Dialyzer-augmented whole blood and plasma exchange for patients with hepatic or hepatorenal failure.* Crit Care Med, 1975. **3**(5): p. 204-9.

97. Busnach, G., et al., *Polymorphonuclear cell phagocytosis and surface receptor modulation after extracorporeal circulation.* ASAIO Transactions, 1989. **35**(3): p. 361-364.

98. Bussel, A., X. Sitthy, and J. Reviron, *[Technological aspects and complications of plasma exchange].* Rev Fr Transfus Immunohematol, 1982. **25**(5): p. 547-76.

99. Bussel, A., X. Sitthy, and J. Reviron, *Technical aspects and complications of plasma-exchange.* Ric Clin Lab, 1983. **13**(1): p. 111-32.

100. Byrnes, J.J., *Plasma infusion in the treatment of thrombotic thrombocytopenic purpura.* Seminars in Thrombosis and Hemostasis, 1981. **7**(1): p. 9-14.

101. Byrnes, J.J. and E.C. Lian, *Recent therapeutic advances in thrombotic thrombocytopenic purpura.* Semin Thromb Hemost, 1979. **5**(3): p. 199-215.

102. Caillot, G., et al., *[Circulating immune complexes: optimization and characteristics of the C1q(I125) fixation test].* Pathol Biol (Paris), 1984. **32**(9): p. 931-7.

103. Caines, G.H., et al., *Transverse relaxation of saline and plasma using Mn(II), HSA-EDTA-Mn, and HSA-EDTA-Gd: application to erythrocyte water exchange.* Magn Reson Med, 1987. **5**(3): p. 269-77.

104. Cameron, J.S., *Treatment of glomerulonephritis by drugs.* Br Med J, 1977. **1**(6074): p. 1457-9.

105. Camici, M., et al., *Plasma exchange with prostaglandin I2 and ACD solution: comparative effects.* Int J Artif Organs, 1986. **9**(6): p. 439-42.

106. Cantrell Jr, J.E., T.M. Phillips, and P.S. Schein, *Carcinoma-associated hemolytic-uremic syndrome: A complication of mitomycin C chemotherapy.* Journal of Clinical Oncology, 1985. **3**(5): p. 723-734.

107. Casper, J.T., et al., *Exchange transfusion in Reye's syndrome with saline-washed red blood cells.* Transfusion, 1976. **16**(2): p. 130-4.

108. Cassetti, M., G.P. Palazzesi, and L. Lorenzoni, *Plasmapheresis and antiplatelet drugs in treatment on thrombotic thrombocytopenic purpura.* Trasfusione del Sangue, 1985. **30**(1): p. 59-62.

109. Chirnside, A., et al., *Coagulation abnormalities following intensive plasma exchange on the cell separator. II. Effects on factors I, II, V, VII, VIII, IX, X and antithrombin III.* Br J Haematol, 1981. **48**(4): p. 627-34.

110. Christensen, N.J., *A sensitive assay for the determination of dopamine in plasma.* Scand J Clin Lab Invest, 1973. **31**(3): p. 343-6.

111. Chu, S.Y. and L.T. Sennello, *High-pressure liquid chromatographic determination of 2-aminoethyl hydrogen sulfate in plasma and urine.* J Chromatogr, 1976. **117**(2): p. 415-23.

112. Clark, R.A., et al., *Cryptic chemotactic activity of fibronectin for human monocytes resides in the 120-kDa fibroblastic cell-binding fragment.* J Biol Chem, 1988. **263**(24): p. 12115-23.

113. Clemmensen, I., *Inhibition of urokinase by complex formation with human antithrombin III in absence and presence of heparin.* Thromb Haemost, 1978. **39**(3): p. 616-23.

114. Clemmensen, I., L.C. Petersen, and C. Kluft, *Purification and characterization of a novel, oligomeric, plasminogen kringle 4 binding protein from human plasma: tetranectin.* Eur J Biochem, 1986. **156**(2): p. 327-33.

115. Coe, F.L., *An inventory of our therapeutic possessions.* Med Clin North Am, 1978. **62**(6): p. 1147-56.

116. Cohen, E., *A perspective of Soviet blood banking.* Transfusion, 1974. **14**(4): p. 315-27.

117. Cole, D.E. and C.R. Scriver, *Microassay of inorganic sulfate in biological fluids by controlled flow anion chromatography.* J Chromatogr, 1981. **225**(2): p. 359-67.

118. Coleman, P.S., *Membrane cholesterol and tumor bioenergetics.* Ann N Y Acad Sci, 1986. **488**: p. 451-67.

119. Connolly, B.M. and H.O. Goodman, *Potential sources of errors in cation-exchange chromatographic measurement of plasma taurine.* Clin Chem, 1980. **26**(3): p. 508-10.

120. Consoli, A., et al., *Determination of Krebs cycle metabolic carbon exchange in vivo and its use to estimate the individual contributions of gluconeogenesis and glycogenolysis to overall glucose output in man.* J Clin Invest, 1987. **80**(5): p. 1303-10.

121. Copplestone, J.A., *Bleeding and coagulation disorders in the elderly.* Baillieres Clin Haematol, 1987. **1**(2): p. 559-80.

122. Cordonnier, D., F.C. Vert-Pre, and F. Bayle, *Mitomycin C nephrotoxicity.* Nephrologie, 1985. **6**(1): p. 19-26.

123. Couser, W.G., *Idiopathic rapidly progressive glomerulonephritis.* Am J Nephrol, 1982. **2**(2): p. 57-69.

124. Cowan, J.F., et al., *An improved method for evaluation of blood coagulation in heparinized blood.* Am J Clin Pathol, 1981. **75**(1): p. 60-4.

125. Crain, S.M. and A.M. Choudhury, *Thrombotic thrombocytopenic purpura. A reappraisal.* Jama, 1981. **246**(11): p. 1243-6.

126. Crastes De Paulet, P., P. Bouyard, and A. Crastes De Paulet, *[Action of k-strophanthoside on erythrocyte-plasma exchange of the potassium ion in human heparinized blood: study in vitro and in vivo].* C R Hebd Seances Acad Sci, 1959. **249**: p. 1829-31.

127. Crastes De Paulet, P. and A. Crastes De Paulet, *Erythrocyte-plasma exchanges of na and K ions in heparinized normal human blood.* Comptes rendus des seances de la Societe de biologie et de ses filiales, 1955. **149**(23-24): p. 2191-2196.

128. Crastes De Paulet, P., A. Crastes De Paulet, and P. Bouyard, *[Exchange of sodium and potassium ions between blood cells and plasma of human blood treated with heparin. II. Study of subjects in hypoglycemic coma and in diabetics].* C R Seances Soc Biol Fil, 1956. **150**(8-9): p. 1607-11.

129. Crespo, B., et al., *Plasma exchange with a very low molecular weight (VLMW) heparin fragment CY 222. Evolution of the coagulation factors.* Thrombosis and haemostasis, 1987. **58**: p. 121.

130. Crone, C., *Exchange of molecules between plasma, interstitial tissue and lymph.* Pflugers Arch, 1972: p. Suppl:65-79.

131. Cser, A., *Metabolic and hormonal changes during and after exchange transfusion with heparinized or ACD blood.* Arch Dis Child, 1974. **49**(12): p. 940-5.

132. Cser, A. and R.D. Milner, *Metabolic and hormonal consequences of exchange transfusion via the umbilical artery or vein.* Biol Neonate, 1975. **27**(1-2): p. 61-70.

133. Cser, A. and R.D. Milner, *Glucose tolerance and insulin secretion in very small babies.* Acta Paediatr Scand, 1975. **64**(3): p. 457-63.

134. Cumming, A.M., et al., *In vitro neutralization of heparin in plasma prior to the activated partial thromboplastin time test: an assessment of four heparin antagonists and two anion exchange resins.* Thromb Res, 1986. **41**(1): p. 43-56.

135. Cuttner, J., *Thrombotic thrombocytopenic purpura: a ten-year experience.* Blood, 1980. **56**(2): p. 302-6.

136. Dahlbäck, B., *Human coagluation factor V purification and thrombin-catalyzed activation.* J Clin Invest, 1980. **66**(3): p. 583-91.

137. Dahlbäck, B., *Purification of human vitamin K-dependent protein S and its limited proteolysis by thrombin.* Biochem J, 1983. **209**(3): p. 837-46.

138. Dahlbäck, B. and E.R. Podack, *Characterization of human S protein, an inhibitor of the membrane attack complex of complement. Demonstration of a free reactive thiol group.* Biochemistry, 1985. **24**(9): p. 2368-74.

139. Damen, J., J. Regts, and G. Scherphof, *Transfer of [14C]phosphatidylcholine between liposomes and human plasma high density lipoprotein. Partial purification of a transfer-stimulating plasma factor using a rapid transfer assay.* Biochim Biophys Acta, 1982. **712**(3): p. 444-52.

140. Danao, T. and E.G. Camara, *The anticardiolipin syndrome.* Am Fam Physician, 1989. **39**(1): p. 107-10.

141. Danesh, B.J., *Influence of acid citrate in stored blood on the level of electrolytes in plasma.* Arch Dis Child, 1971. **46**(250): p. 879.

142. d'Apice, A.J., et al., *Treatment of severe pre-eclampsia by plasma exchange.* Aust N Z J Obstet Gynaecol, 1980. **20**(4): p. 231-5.

143. D'Apice, A.J.F., L.L. Reti, and R.J. Pepperell, *Treatment of severe pre-eclampsia by plasma exchange.* Australian and New Zealand Journal of Obstetrics and Gynaecology, 1980. **20**(4): p. 231-235.

144. Dau, P.C. and T. Wolff, *Therapeutic plasma exchange in neurological diseases.* J Clin Apher, 1985. **2**(4): p. 381-4.

145. De Gineste, J., et al., *Tkombcxjyte transfusions in pediatrics.* Archives Francaises de Pediatrie, 1966. **23**(9): p. 1089-1096.

146. De Wolf, M.J., A.R. Lagrou, and H.J. Hilderson, *Subcellular structure of bovine thyroid gland. The localization of the peroxidase activity in bovine thyroid.* Biochem J, 1978. **174**(3): p. 939-49.

147. del Zoppo, G.J., *Antiplatelet therapy in thrombotic thrombocytopenic purpura.* Semin Hematol, 1987. **24**(2): p. 130-9.

148. Dennis, M.B., Jr., et al., *Successful long-term use of a miniaturized plasmapheresis circuit in rabbits.* ASAIO Trans, 1988. **34**(3): p. 651-4.

149. Dietrich, W., et al., *Autotransfusion and hemoseparation in cardiac surgery. What can be saved in cardiac reoperations and operations of thoracic aortic aneurysms?* Thorac Cardiovasc Surg, 1989. **37**(2): p. 84-8.

150. Dippell, J., K.M. Koch, and W. Fassbinder, *Syndrome of glomerulonephritis and pulmonary hemorrhage in pediatrics.* Monatsschrift fur Kinderheilkunde, 1976. **124**(5): p. 450-452.

151. Dodds, A. and M. Nicholls, *Haematological aspects of renal disease.* Anaesth Intensive Care, 1983. **11**(4): p. 361-8.

152. Doll, N.J. and J.E. Salvaggio, *Stroke and gangrene: Complications of therapeutic plasma exchange therapy.* Clinical and Experimental Dialysis and Apheresis, 1981. **5**(4): p. 415-421.

153. Dominey, A.M. and J.A. Tschen, *Cutaneous manifestations of dysproteinemias.* Dermatologic Clinics, 1989. **7**(3): p. 449-466.

154. Domula, M. and G. Weissbach, *[Heparin and antiheparin in childhood. 3. Heparin level measurements and their importance in heparin monitoring].* Folia Haematol Int Mag Klin Morphol Blutforsch, 1983. **110**(1): p. 146-61.

155. Donaldson, L.A., R.W. Williams, and W.G. Schenk, Jr., *Experimental pancreatitis: Effect of plasma and dextran on pancreatic blood flow.* Surgery, 1978. **84**(3): p. 313-21.

156. Dormandy, J.A., *Practical impact of hemorheology on the treatment of chronic peripheral ischemia.* Angiology, 1981. **32**(10): p. 710-4.

157. Drescher, W.P., et al., *Massive extracorporeal blood clotting during discontinuous flow leukapheresis.* Transfusion, 1978. **18**(1): p. 89-90.

158. D'Souza, S. and R. Ananthakrishnan, *Affinity chromatography in the separation of human alpha 1-antitrypsin (alpha 1-AT) and antithrombin-III (AT-III).* Aust J Exp Biol Med Sci, 1979. **57**(3): p. 245-50.

159. Duc, G. and G. De Muralt, *[Acid-bases and electrolytes during exchange transfusion with citrated blood. Preliminary communication].* Acta Paediatr Scand, 1967: p. Suppl 172:46+.

160. Duc, G. and G. de Muralt, *[Acid-base quilibrium, lactate and pyruvate, electrolytes during exchange transfusion performed with citrated blood].* Schweiz Med Wochenschr, 1967. **97**(36): p. 1176-9.

161. Dyfverman, A. and J. Sjövall, *Ion-pair extraction of bile acids with Lipidex gel.* Anal Biochem, 1983. **134**(2): p. 303-8.

162. Ebbesen, F., *Effect of exchange transfusion on serum reserve albumin for binding of bilirubin and index of serum bilirubin toxicity.* Acta Paediatr Scand, 1981. **70**(5): p. 643-8.

163. Ehnholm, C., H. Greten, and W.V. Brown, *A comparative study of post-heparin lipolytic activity and a purified human plasma triacylglycerol lipase.* Biochim Biophys Acta, 1974. **360**(1): p. 68-77.

164. Ehnholm, C. and T. Kuusi, *Preparation, characterization, and measurement of hepatic lipase.* Methods Enzymol, 1986. **129**: p. 716-38.

165. Eisenberg, S., et al., *On the metabolic conversion of human plasma very low density lipoprotein to low density lipoprotein.* Biochim Biophys Acta, 1973. **326**(3): p. 361-77.

166. Eisenhauer, T., V.W. Armstrong, and H. Wieland, *Selective removal of low density lipoproteins (LDL) by precipitation at low pH: First clinical application of the HELP system.* Klinische Wochenschrift, 1987. **65**(4): p. 161-168.

167. Eisenhauer, T., et al., *Selective removal of low density lipoproteins (LDL) by precipitation at low pH: first clinical application of the HELP system.* Klin Wochenschr, 1987. **65**(4): p. 161-8.

168. Ellsworth, J.L., L. McVittie, and R.L. Jackson, *Human plasma lipid exchange protein(s): a method for separation of donor and acceptor lipoproteins by heparin-Sepharose chromatography.* J Lipid Res, 1982. **23**(4): p. 653-9.

169. Elphick, M.C. and D. Hull, *Rabbit placental clearing-factor lipase and transfer to the foetus of fatty acids derived from triglycerides injected into the mother.* J Physiol, 1977. **273**(2): p. 475-87.

170. Endo, Y., et al., *Applications of protease inhibitor to hemoperfusion and plasma exchange as a regional anticoagulant.* Trans Am Soc Artif Intern Organs, 1985. **31**: p. 429-33.

171. Endo, Y., et al., *Efficacy of Nafamostat mesilate as a regional anticoagulant on clinical plasma exchange in the patient with bleeding.* Japanese Journal of Artificial Organs, 1988. **17**(1): p. 306-309.

172. Ermolenko, I.N., et al., *[New polymers for transfusiology].* Polim Med, 1976. **6**(3): p. 135-42.

173. Exner, T. and R. Vaasjoki, *Characterisation and some properties of the protein C activator from Agkistrodon Contortrix Contortrix venom.* Thromb Haemost, 1988. **59**(1): p. 40-4.

174. Fabris, F., et al., *Effect of heparin and aspirin on platelet and clotting activation during leukapheresis.* Eur J Clin Invest, 1985. **15**(4): p. 188-91.

175. Fahn, S., A.L. Prasad, and R. Delesie, *A reliable and simple method for simultaneous determination of DOPA and 3-O-methyldopa in plasma and brain.* Anal Biochem, 1972. **46**(2): p. 557-75.

176. Fairwell, T., et al., *Human plasma apolipoprotein C-II: total solid-phase synthesis and chemical and biological characterization.* Proc Natl Acad Sci U S A, 1987. **84**(14): p. 4796-800.

177. Falco, C., et al., *LDL-apheresis by dextran sulfate cellulose adsorption columns: two methods.* Beitr Infusionsther, 1988. **23**: p. 191-4.

178. Farrell, P.C., *Kinetic modeling: applications in renal and related diseases.* Kidney Int, 1983. **24**(4): p. 487-95.

179. Feliu, E., et al., *Comparative morphology of granulocytes collected by three methods of leukapheresis. A light microscopy and transmission electron microscopy study.* Transfusion, 1981. **21**(5): p. 517-26.

180. Fenton, E., H.G. Britton, and D.A. Nixon, *A study of the permeability of the guinea pig placenta to citrate using recirculating placental perfusion technique.* Biol Neonate, 1976. **29**(5-6): p. 299-305.

181. Fenton, E., H.G. Britton, and D.A. Nixon, *The supply of citrate to the sheep fetus.* Biol Neonate, 1978. **34**(1-2): p. 92-6.

182. Ferguson, E., A. Vaughan, and J. Swale, *A method for the estimation of total calcium in serum or heparinized plasma.* Clin Chim Acta, 1976. **67**(3): p. 281-6.

183. Finck, M., et al., *Autotransfusion and plasmapheresis in order to prepare for operation. Reaction of coagulation parameters.* Anaesthesist, 1985. **34**(12): p. 675-680.

184. Fischer, M., *[Transfusion therapy of non-hemophilic hemorrhagic diatheses: thrombopenias, thrombopathies, hemorrhagic thrombocytemias, consumption coagulopathies, anticoagulant induced hemorrhages].* Bibl Haematol, 1967. **27**: p. 211-27.

185. Flaum, M.A., et al., *The hemostatic imbalance of plasma-exchange transfusion.* Blood, 1979. **54**(3): p. 694-702.

186. Flessner, M.F., R.L. Dedrick, and J.S. Schultz, *Exchange of macromolecules between peritoneal cavity and plasma.* Am J Physiol, 1985. **248**(1 Pt 2): p. H15-25.

187. Forestier, F., et al., *Absence of transplacental passage of pentosan polysulfate during mid trimester of pregnancy.* Thromb Haemost, 1986. **56**(3): p. 247-9.

188. Fosburg, M., et al., *Intensive plasma exchange in small and critically ill pediatric patients: techniques and clinical outcome.* J Clin Apher, 1983. **1**(4): p. 215-24.

189. Fourrier, F., et al., *Decrease of angiotensin-converting enzyme activity after plasma exchange.* Crit Care Med, 1988. **16**(2): p. 105-10.

190. Fourrier, F., et al., *[Multiple thrombosis and thrombocytopenia due to heparin occurring after plasma exchange].* Presse Med, 1989. **18**(7): p. 359.

191. Francès, C., et al., *Cutaneous necrosis associated with the lupus anticoagulant.* Dermatologica, 1989. **178**(4): p. 194-201.

192. Franceschini, G., et al., *Apheretic treatment of severe familial hypercholesterolemia: comparison of dextran sulfate cellulose and double membrane filtration methods for low density lipoprotein removal.* Atherosclerosis, 1988. **73**(2-3): p. 197-202.

193. Franceschini, G., et al., *Comparison of dextran sulphate cellulose and double filtration plasmaphereses for the treatment of severe hypercholesterolemia.* Beitr Infusionsther, 1988. **23**: p. 112-7.

194. Frappaz, D., et al., *[Multiple vascular thromboses in severe acute autoimmune hemolytic anemia with Mycoplasma pneumoniae serology treated by plasma exchange and immunosuppressive agents].* Pediatrie, 1983. **38**(6): p. 411-9.

195. Frascà, G.M., et al., *Optimization of heparin anticoagulation during membrane plasma separation.* Int J Artif Organs, 1988. **11**(4): p. 313-6.

196. Freed, L.E., et al., *A novel bioreactor based on suspended particles of agarose-immobilized species.* ASAIO Trans, 1988. **34**(3): p. 732-8.

197. Freireich, E.J., *Future trends in apheresis.* Prog Clin Biol Res, 1981. **65**: p. 155-62.

198. French, J.E., J.M. Solomon, and J.C. Fratantoni, *Survey on the current use of leukapheresis and the collection of granulocyte concentrates.* Transfusion, 1982. **22**(3): p. 220-5.

199. Friedman, Z., W.B. Hanley, and I.C. Radde, *Ionized calcium in exchange transfusion with THAM-buffered ACD blood.* Can Med Assoc J, 1972. **107**(8): p. 742-5.

200. Friman, C. and M. Juvani, *Acid glycosaminoglycans in plasma. I. Determination.* Scand J Rheumatol, 1977. **6**(2): p. 87-91.

201. Fujii, H., *[Plasmapheresis therapy in primary macroglobulinemia with liver cirrhosis: replacement with dextran 40].* Rinsho Ketsueki, 1985. **26**(11): p. 1860-4.

202. Fujii, H., *[Plasmapheresis with a single use of dextran 40 in primary macroglobulinemia].* Nihon Ketsueki Gakkai Zasshi, 1985. **48**(5): p. 1214-20.

203. Fujii, H., *Plasma exchange using dextran 40-electrolyte solution as the sole replacement fluid in malignant paraproteinemia.* Transfusion, 1988. **28**(1): p. 42-5.

204. Funakoshi, T., et al., *Human placental anticoagulant protein: isolation and characterization.* Biochemistry, 1987. **26**(17): p. 5572-8.

205. Gajdos, P., et al., *Testing a cellulose diacetate membrane for plasma exchange.* Nouvelle Presse Medicale, 1981. **10**(42): p. 3469-3471.

206. Gambino, S.R., *Heparinized vacuum tubes for determination of plasma pH, plasma CO2 content, and blood oxygen saturation with an extensive discussion of pH methodology.* Tech Bull Regist Med Technol, 1959. **29**: p. 123-31.

207. Ganichkin, A.M., et al., *Plasmapheresis in the complex treatment of nonspecific ulcerative colitis.* Vestnik khirurgii imeni I. I. Grekova, 1988. **140**(2): p. 106-108.

208. Ganichkin, A.M., et al., *[Plasmapheresis in the complex treatment of nonspecific ulcerative colitis].* Vestn Khir Im I I Grek, 1988. **140**(2): p. 106-8.

209. Ganz, P.R., et al., *Human factor VIII from heparinized plasma. Purification and characterization of a single-chain form.* Eur J Biochem, 1988. **170**(3): p. 521-8.

210. Garcia, M.A. and E.F. Graham, *Factors affecting the removal of low-molecular-weight fractions (LMWF) from egg yolk and seminal plasma in extended semen by dialysis: effect on post-thaw sperm survival.* Cryobiology, 1987. **24**(5): p. 437-45.

211. Garel, J.M., et al., *Fetal-maternal plasma calcium relationships in rat and sheep.* J Physiol (Paris), 1972. **64**(4): p. 387-98.

212. Gavrilov, O.K., et al., *[Evaluation of the antithrombotic effect of plasma and thrombocyte apheresis in the complex therapy of patients with unstable stenocardia].* Kardiologiia, 1988. **28**(5): p. 60-4.

213. Geddes, V.A., et al., *A moderate form of hemophilia B is caused by a novel mutation in the protease domain of factor IXVancouver.* J Biol Chem, 1989. **264**(8): p. 4689-97.

214. Gelb, A.F., et al., *Immune complexes, gallium lung scans, and bronchoalveolar lavage in idiopathic interstitial pneumonitis-fibrosis. A structure-function clinical study.* Chest, 1983. **84**(2): p. 148-153.

215. Gerron, G.G., et al., *Technical pitfalls in measurement of venous plasma NH3 concentration.* Clin Chem, 1976. **22**(5): p. 663-6.

216. Geyer, R.P., *Substitutes for blood and its components.* Prog Clin Biol Res, 1978. **19**: p. 1-26.

217. Glaser, M. and K. Schirmer, *The IgA-nephropathy.* Zeitschrift fur die Gesamte Innere Medizin und Ihre Grenzgebiete, 1987. **42**(24): p. 711-714.

218. Glas-Greenwalt, P., et al., *Fibrinolysis in health and disease: abnormal levels of plasminogen activator, plasminogen activator inhibitor, and protein C in thrombotic thrombocytopenic purpura.* J Lab Clin Med, 1986. **108**(5): p. 415-22.

219. Glassock, R.J., *Immunosuppressive treatment in the prevention of renal failure in primary glomerular diseases.* Clin Exp Dial Apheresis, 1981. **5**(1-2): p. 21-46.

220. Glassock, R.J., *Are anticoagulants of value in the treatment of renal disease? An introduction to the controversy.* Am J Kidney Dis, 1984. **3**(4): p. 297-8.

221. Gmür, J., J. Deluigi, and P.W. Straub, *[Improvement in the granulocyte harvest from normal donors by means of a combination of "continuous flow" centrifugation and filter leukapheresis].* Schweiz Med Wochenschr, 1975. **105**(47): p. 1589-90.

222. Godeau, P. and B. Wechsler, *[Behçet's disease, a current disease].* J Mal Vasc, 1988. **13**(3): p. 215-9.

223. Goldsmith, J.C. and H.E. Hamilton, *An idiopathic factor VIII anticoagulant: resolution following plasmapheresis and cytotoxic therapy.* Am J Med Sci, 1985. **290**(6): p. 246-8.

224. Gordon, B.J., et al., *Evaluation of leukapheresis and thrombocytapheresis in the horse.* Am J Vet Res, 1986. **47**(5): p. 997-1001.

225. Gottschall, J.L., et al., *Thrombotic thrombocytopenic purpura: Experience with whole blood exchange transfusion.* Seminars in Thrombosis and Hemostasis, 1981. **7**(1): p. 25-32.

226. Grabensee, B. and J. Passlick, *Treatment of the hemolytic-uremic syndrome in adults.* Deutsche Medizinische Wochenschrift, 1987. **112**(27): p. 1089-1091.

227. Grasso, J.A., T.J. Hillis, and J.A. Mooney-Frank, *Mobilization of ferritin iron in erythroblasts by chelating agents.* Biochim Biophys Acta, 1985. **845**(1): p. 109-18.

228. Graw Ji, R.G., C.D. Buckner, and K. Eiscl, *Plasma exchange transfusion for hepatic coma: New technic.* Transfusion, 1970. **10**(1): p. 781-783.

229. Graw Jr, R.G., C.D. Buckner, and R. Eisel, *Plasma exchange transfusion for hepatic coma. New technic.* Transfusion, 1970. **10**(1): p. 26-32.

230. Graw, R.G., Jr., C.D. Buckner, and R. Eisel, *Plasma exchange transfusion for hepatic coma. New technic.* Transfusion, 1970. **10**(1): p. 26-32.

231. Graybeal, F.Q., Jr., D.E. Mooreside, and R.D. Langdell, *Clotting factor activity in cryoprecipitates and supernatant plasma prepared from blood collected into ACD, ACD-adenine, CPD, and CPD-adenine and from plasma collected by plasmapheresis.* Transfusion, 1969. **9**(3): p. 135-40.

232. Grem, J.L., J.A. Merritt, and P.P. Carbone, *Treatment of mitomycin-associated microangiopathic hemolytic anemia with vincristine.* Arch Intern Med, 1986. **146**(3): p. 566-8.

233. Gries, A., et al., *Characterization of isoelectric subspecies of asialo-beta 2-glycoprotein I.* Biochem J, 1989. **260**(2): p. 531-4.

234. Grindon, A.J., *Adverse reactions to whole blood donation and plasmapheresis.* Crit Rev Clin Lab Sci, 1982. **17**(1): p. 51-75.

235. Guillevin, L., et al., *[Periarteritis nodosa: clinical and therapeutic study of 126 patients followed-up during 23 years].* Ann Med Interne (Paris), 1985. **136**(1): p. 6-12.

236. Guillevin, L., C. Lok, and A. Bussel, *Side effects during the treatment of polyarteritis nodosa and Churg-Strauss angiitis with corticosteroids, plasma exchange, and cyclophosphamide: 377 sessions in 30 patients.* Plasma Therapy and Transfusion Technology, 1985. **6**(3): p. 557-560.

237. Gupta, B.B., M.Y. Jaffrin, and L.H. Ding, *Modelling of plasma-separation through microporous membranes.* Int J Artif Organs, 1989. **12**(1): p. 51-8.

238. Gupta, V.J. and D.E. Wilcken, *The detection of cysteine-homocysteine mixed disulphide in plasma of normal fasting man.* Eur J Clin Invest, 1978. **8**(4): p. 205-7.

239. Gus'kova, A.K., et al., *[The diagnosis, clinical picture and treatment of acute radiation sickness in the victims of the Chernobyl Atomic Electric Power Station. II. Non-bone marrow syndromes of radiation lesions and their treatment].* Ter Arkh, 1989. **61**(8): p. 99-103.

240. Gutiérrez, J.M., B. Lomonte, and L. Cerdas, *Isolation and partial characterization of a myotoxin from the venom of the snake Bothrops nummifer.* Toxicon, 1986. **24**(9): p. 885-94.

241. Gutschmidt, H.J., et al., *Cyclophosphamide stoss-therapy synchronized with plasmapheresis in rapidly progressive glomerulonephritis.* Deutsche Medizinische Wochenschrift, 1986. **111**(38): p. 1439-1444.

242. Haagensen, D.E., Jr., et al., *Buffer-exchange column for rapid separation of carcinoembryonic antigen from perchloric acid.* Clin Chem, 1978. **24**(1): p. 135-7.

243. Haanen, C. and R. Holdrinet, *Pathogenesis and treatment of disseminated thrombotic microangiopathy (Moschcowitz' syndrome).* Clinical Hemorheology, 1982. **2**(5-6): p. 733-743.

244. Haave, N.C. and S.M. Innis, *Effects of cholestyramine feeding on tissue lipase activities and plasma fatty acids in the pregnant rat.* J Dev Physiol, 1989. **12**(1): p. 11-4.

245. Hagler, H.K., et al., *Five-day partial bypass using a membrane oxygenator without systemic heparinzation.* Trans Am Soc Artif Intern Organs, 1975. **21**: p. 178-87.

246. Hahn, D. and A.M. Ganzoni, *Functional heterogeneity of the transport iron compartment II. In vivo differences between transferrin iron binding sites, and in vitro interbinding site iron exchange.* Acta Haematol, 1975. **53**(6): p. 321-8.

247. Hall, I.H., et al., *Antihyperlipidemic activity of saccharin analogues in rodents.* J Pharm Sci, 1983. **72**(10): p. 1192-8.

248. Hammer, C.H., et al., *Large scale isolation of functionally active components of the human complement system.* J Biol Chem, 1981. **256**(8): p. 3995-4006.

249. Harbaugh, M.E., E.M. Hill, and R.B. Conn, *Antithrombin and antithromboplastin activity accompanying IgG myeloma. Report of a case with a severe bleeding tendency.* Am J Clin Pathol, 1975. **63**(1): p. 57-67.

250. Harisdangkul, V. and E.A. Kabat, *Studies on human antibodies. IX. Interaction of 1-(m-nitrophenyl)-flavazoles of isomaltose oligosaccharides with purified antidextrans: quantitative hapten-inhibition and fluorescence quenching studies.* J Immunol, 1972. **108**(5): p. 1232-43.

251. Harris, F. and J.A. Black, *Plasma citrate levels during exchange transfusion.* Arch Dis Child, 1971. **46**(249): p. 736.

252. Hashimoto, H., *[Clinical study of autoimmune diseases: recent trends--with special reference to progress in immunological tests and therapeutic methods. Systemic autoimmune diseases. 5) Periarteritis nodosa and necrotic vasculitis].* Nihon Rinsho, 1988. **46**(4): p. 888-93.

253. Haycock, G.B., *The treatment of glomerulonephritis in children.* Pediatr Nephrol, 1988. **2**(2): p. 247-55.

254. Heaf, J.G., F. Jørgensen, and L.P. Nielsen, *Treatment and prognosis of extracapillary glomerulonephritis.* Nephron, 1983. **35**(4): p. 217-24.

255. Hedley, R. and M.W. Bradbury, *Transport of polar non-electrolytes across the intact and perfused guinea-pig placenta.* Placenta, 1980. **1**(4): p. 277-85.

256. Held, E., *Therapy of rapidly progressive glomerulonephritis with anticoagulants?* Nieren- und Hochdruckkrankheiten, 1981. **10**(3): p. 115-119.

257. Hendrick, A.M., *Risk factors associated with thrombosis following plasma exchange.* Plasma Therapy and Transfusion Technology, 1986. **7**(2): p. 213-218.

258. Hendriks, D., et al., *Characterisation of a carboxypeptidase in human serum distinct from carboxypeptidase N.* J Clin Chem Clin Biochem, 1989. **27**(5): p. 277-85.

259. Heppner, R.L., R.B. Harvey, and J.B. Bassingthwaighte, *Creatinine distribution dynamics in the dog.* J Appl Physiol, 1972. **32**(4): p. 495-500.

260. Herrmann, J., et al., *Plasmapheresis in the treatment of thyrotoxic crisis.* Deutsche Medizinische Wochenschrift, 1974. **99**(17): p. 888-892.

261. Herrmann, J. and E. Kallee, *[Letter: Therapy of thyrotoxic crises].* Dtsch Med Wochenschr, 1974. **99**(36): p. 1788-9.

262. Herzig, G.P., *Leukocyte donor and recipient reactions with filtration leukapheresis: their character, frequency, and management.* Exp Hematol, 1979. **7**(4 Suppl): p. 31-5.

263. Hester, J.P., *Donor symptomatology and safety in blood cell separation procedures.* Prog Clin Biol Res, 1982. **88**: p. 115-24.

264. Hetenyi, G., Jr., *Correction for the metabolic exchange of 14C for 12C atoms in the pathway of gluconeogenesis in vivo.* Fed Proc, 1982. **41**(1): p. 104-9.

265. Hiraishi, M., et al., *Anticoagulation for the hepatic assist system using FUT-175 and dipyridamole.* Japanese Journal of Artificial Organs, 1988. **17**(1): p. 231-234.

266. Hiraishi, M., et al., *Plasma collection using nafamostat mesilate and dipyridamole as an anticoagulant.* Int J Artif Organs, 1988. **11**(3): p. 212-6.

267. Holderman, C. and R.G. Schlesinger, *Modified plasma therapy using the haemonetics30 blood processor.* Plasma Therapy and Transfusion Technology, 1981. **2**(1): p. 31-33.

268. Holland, P., et al., *Hepatitis-associated antigen and antibody in cold ethanol fractionates of human blood.* Vox Sang, 1971. **20**(5): p. 464-5.

269. Holmsen, I. and H. Holmsen, *Partial purification and characterization of an ADP phosphohydrolase from human plasma.* Thromb Diath Haemorrh, 1971. **26**(1): p. 177-91.

270. Hölscher, B. and S. Kagel, *[Blood regeneration after blood substitution with dextran 60 or hydroxyethyl starch of an identical volume].* Infusionsther Klin Ernahr, 1976. **3**(4): p. 250-3.

271. Homma, Y., et al., *Comparison of selectivity of LDL removal by double filtration and dextran-sulfate cellulose column plasmapheresis.* Atherosclerosis, 1986. **60**(1): p. 23-7.

272. Horiuchi, T., et al., *Membrane plasma filtration (MPF): effect of temperature on heparin and macromolecule sieving.* Trans Am Soc Artif Intern Organs, 1985. **31**: p. 692-7.

273. Horiuchi, T., et al., *Effect of plasma solute-membrane interaction on mean pore diameter.* ASAIO Trans, 1986. **32**(1): p. 429-34.

274. Hortin, G., D.M. Tollefsen, and A.W. Strauss, *Identification of two sites of sulfation of human heparin cofactor II.* J Biol Chem, 1986. **261**(34): p. 15827-30.

275. Hosokawa, S., A. Oyamaguchi, and O. Yoshida, *Optimization of heparinization in clinical double filtration plasmapheresis.* Int J Artif Organs, 1989. **12**(8): p. 544-8.

276. Howard, A.N. and D.E. Hyams, *Combined use of clofibrate and cholestyramine or DEAE sephadex in hypercholesterolaemia.* Br Med J, 1971. **3**(5765): p. 25-7.

277. Howe, H.S., et al., *Pulmonary haemorrhage, pulmonary infarction, and the lupus anticoagulant.* Ann Rheum Dis, 1988. **47**(10): p. 869-72.

278. Howland, W.S., et al., *Factors influencing the ionization of calcium during major surgical procedures.* Surg Gynecol Obstet, 1976. **143**(6): p. 895-900.

279. Hoyoux, P., M. Malaise, and J.P. Kinet, *Therapeutic value of plasmapheresis techniques.* Revue Medicale de Liege, 1982. **37**(8): p. 303-312.

280. Huestis, D.W., *Adverse effects of granulocyte donations.* Prog Clin Biol Res, 1982. **88**: p. 101-14.

281. Huestis, D.W., et al., *Use of hydroxyethyl starch to improve granulocyte collection in the Latham blood processor.* Transfusion, 1975. **15**(6): p. 559-64.

282. Hugentobler, G. and P.J. Meier, *Multispecific anion exchange in basolateral (sinusoidal) rat liver plasma membrane vesicles.* Am J Physiol, 1986. **251**(5 Pt 1): p. G656-64.

283. Hunt, F.C., *Phenolic aminocarboxylic acids as gallium-binding radiopharmaceuticals.* Nuklearmedizin, 1984. **23**(3): p. 123-5.

284. Huttunen, J.K., et al., *Effect of fasting on two postheparin plasma triglyceride lipases and triglyceride removal in obese subjects.* Eur J Clin Invest, 1975. **5**(6): p. 435-45.

285. Iacone, A., et al., *Plasma exchange as intensive therapy in thrombotic thrombocytopenic purpura.* Haematologica, 1985. **70**(3): p. 225-31.

286. Ingram, G.I. and G. Hambleton, *Anticoagulation and pregnancy.* Lancet, 1970. **2**(7687): p. 1359.

287. Ingram, S.B., S.H. Goodnight, Jr., and R.M. Bennett, *An unusual syndrome of a devastating noninflammatory vasculopathy associated with anticardiolipin antibodies: report of two cases.* Arthritis Rheum, 1987. **30**(10): p. 1167-72.

288. Inoue, N., *Approach to hepatic assist utilized in Japan.* Artif Organs, 1988. **12**(4): p. 296-9.

289. Inoue, N., et al., *Clinical application of nafamostat mesilate (FUT-175) as an anticoagulant during extracorporeal circulation for therapeutic plasmapheresis.* Japanese Pharmacology and Therapeutics, 1987. **15**(10): p. 425-434.

290. Island, D.P., et al., *A METHOD FOR SEPARATING SMALL QUANTITIES OF MSH AND ACTH WITH GOOD RECOVERY OF EACH.* J Clin Endocrinol Metab, 1965. **25**: p. 975-83.

291. Israel, L., R. Edelstein, and P. Mannoni, *Plasmapheresis in patients with disseminated cancer: clinical results and correlation with changes in serum protein. The concept of 'nonspecific blocking factors'.* Plasma Therapy and Transfusion Technology, 1979. **1**(1): p. 57-68.

292. Itagaki, N., et al., *[Plasma exchange in non-hemophilic patient with circulating anticoagulant responsive to factor VIII].* Rinsho Ketsueki, 1983. **24**(7): p. 904-9.

293. Jaffe, P. and D.F. Mosher, *Plasma antithrombin III and plasminogen levels in chronic plasmapheresis.* N Engl J Med, 1981. **304**(13): p. 789.

294. Jansson, E. and L. Kaijser, *Substrate utilization and enzymes in skeletal muscle of extremely endurance-trained men.* J Appl Physiol (1985), 1987. **62**(3): p. 999-1005.

295. Jindal, B.K., M.F. Martin, and A. Gayner, *Gangrene developing after minor surgery in a patient with undiagnosed systemic lupus erythematosus and lupus anticoagulant.* Ann Rheum Dis, 1983. **42**(3): p. 347-9.

296. Jobard, J., et al., *[Hemorrhagic syndrome caused by a circulating antifactor V anticoagulant. Correction by transfusion of fresh frozen plasma and plasma exchange].* Presse Med, 1983. **12**(25): p. 1606.

297. Johnson, A., et al., *Fibrin degradation products increase lung transvascular fluid filtration after thrombin-induced pulmonary microembolism.* Thromb Res, 1985. **37**(4): p. 543-54.

298. Johnson, F.B. and D.M. Donaldson, *Purification of staphylocidal beta-lysin from rabbit serum.* J Bacteriol, 1968. **96**(3): p. 589-95.

299. Johnson, S.A. and J.W. Rebuck, *Platelet transfusions.* CRC Crit Rev Clin Lab Sci, 1970. **1**(1): p. 25-43.

300. Jones, P.H., *The use of combined LDL affinity apheresis utilizing dextran sulfate cellulose columns and hypolipidemic medications in patients with severe hypercholesterolemia to assess regression of atherosclerosis.* Beitr Infusionsther, 1988. **23**: p. 142-5.

301. Jørgensen, M., L.C. Petersen, and S. Thorsen, *Purification and characterization of hereditary abnormal antithrombin III with impaired thrombin binding.* J Lab Clin Med, 1984. **104**(2): p. 245-56.

302. Kahn, S.I., et al., *Spontaneous recovery of the hemolytic uremic syndrome with prolonged renal and neurological manifestations.* Nephron, 1982. **32**(2): p. 188-191.

303. Kasper, C.K., *Blood-its derivatives and its problems--factor IX.* Ann N Y Acad Sci, 1975. **240**: p. 172-80.

304. Kasper, C.K., et al., *Determinants of factor VIII recovery in cryoprecipitate.* Transfusion, 1975. **15**(4): p. 312-22.

305. Katsume, C., et al., *Cryogel studies for the optimization of cryofiltration (CF) therapy.* Trans Am Soc Artif Intern Organs, 1983. **29**: p. 463-7.

306. Kawara, A., et al., *[Experimental study on diabetes and pregnancy: with special reference to the effects of insulin treatment on fetuses of diabetic dams].* Nihon Naibunpi Gakkai Zasshi, 1983. **59**(8): p. 1067-85.

307. Kazibutowska, Z., et al., *[Treatment of Guillain-Barré syndrome by the method of fractionated plasma exchange with controlled hemodilution].* Neurol Neurochir Pol, 1985. **19**(5): p. 374-80.

308. Keller, F., et al., *Effect of repeated plasma exchange on steady state kinetics of digoxin and digitoxin.* Arzneimittelforschung, 1984. **34**(1): p. 83-6.

309. Kihlström, I., *Placental transfer of benzo(a)pyrene and its hydrophilic metabolites in the guinea pig.* Acta Pharmacol Toxicol (Copenh), 1986. **58**(4): p. 272-6.

310. Kikugawa, K. and K. Minoshima, *Filter columns for preparation of leukocyte-poor blood for transfusion.* Vox Sang, 1978. **34**(5): p. 281-90.

311. Kincaid-Smith, P., *The treatment of glomerulonephritis.* Australian and New Zealand Journal of Medicine, 1980. **10**(3): p. 340-345.

312. Kincaid-Smith, P., *Anticoagulants are of value in the treatment of renal disease.* Am J Kidney Dis, 1984. **3**(4): p. 299-307.

313. Kincaid-Smith, P., *Prevention of kidney arteriosclerosis: heparin, dipyridamole, warfarin, and plasma exchange.* Transplant Proc, 1987. **19**(4 Suppl 5): p. 82-4.

314. Kincaid-Smith, P., K.F. Fairley, and M. Kloss, *Lupus anticoagulant associated with renal thrombotic microangiopathy and pregnancy-related renal failure.* Q J Med, 1988. **68**(258): p. 795-815.

315. Kingsley, J.R., et al., *Citrate anticoagulation and on-line cell washing in intraoperative autotransfusion in the baboon.* Surg Forum, 1973. **24**: p. 258-60.

316. Kingsley, J.R., et al., *Citrate anticoagulation and cell washing for intraoperative autotransfusion in the baboon.* Am J Surg, 1976. **131**(6): p. 717-21.

317. Kinkade, J.M., Jr., W.R. Vogler, and P.G. Dayton, *Plasma levels of methotrexate in cancer patients as studied by an improved spectrophotofluorometric method.* Biochem Med, 1974. **10**(4): p. 337-50.

318. Kirichenko, L.L., et al., *Status of the thrombocytic link of hemostasis and microcirculation in patients with coronary heart disease during plasmapheresis treatment.* Kardiologiya, 1989. **29**(12): p. 85-90.

319. Kirichenko, L.L., et al., *[State of the blood platelet link of hemostasis and microcirculation in patients with ischemic heart disease during plasmapheresis treatment].* Kardiologiia, 1989. **29**(12): p. 85-90.

320. Kirsch, W., M. Büttner, and E. Wenzel, *[Diagnostic therapeutic problems of defibrination syndrome in shock, sepsis, and neonatal hypoxia (author's transl)].* Monatsschr Kinderheilkd (1902), 1977. **125**(6): p. 621-7.

321. Kjellén, L., I. Pettersson, and M. Höök, *Cell-surface heparan sulfate: an intercalated membrane proteoglycan.* Proc Natl Acad Sci U S A, 1981. **78**(9): p. 5371-5.

322. Klein, J.D. and F.J. Walker, *Purification of a protein C activator from the venom of the southern copperhead snake (Agkistrodon contortrix contortrix).* Biochemistry, 1986. **25**(15): p. 4175-9.

323. Kleinman, S., et al., *Exchange red blood cell pheresis in the management of complications of sickle cell anemia.* Plasma Therapy and Transfusion Technology, 1980. **1**(3): p. 27-34.

324. Kling, D., et al., *[Heparin elimination and free hemoglobin following cell separation and washing of autologous blood with Cell Saver 4].* Anasth Intensivther Notfallmed, 1988. **23**(2): p. 88-90.

325. Klock, J.C. and T.P. Stossel, *Detection, pathogenesis, and prevention of damage to human granulocytes caused by interaction with nylon wool fiber. Implications for filtration leukapheresis.* J Clin Invest, 1977. **60**(5): p. 1183-90.

326. Kluthe, R., *Experimental data on pathogenesis of the 'nephrotic syndrome'. I. Dysproteinaemia and hypercholesterolaemia in experimental chronic depletion of protein by plasmapheresis in the rabbit.* Z. Ges. Exp. Med., 1959. **130**(6): p. 613-619.

327. Koepke, J.A., et al., *A comparison of platelet production methods suitable for a service-oriented blood donor center.* Transfusion, 1975. **15**(1): p. 39-42.

328. Koide, T., et al., *Isolation and characterization of a hereditary abnormal antithrombin III 'Antithrombin III Toyama'.* Thromb Res, 1983. **31**(2): p. 319-28.

329. Kolff, W.J., *The artificial kidney.* Thesis, 1946.

330. Kolff, W.J., *New methods in combating uraemia. Peritoneal irrigation, and use of the artificial kidney.* Belgisch tijdschrift voor geneeskunde, 1946. **2**(8): p. 449-463.

331. Komoriyama, H., et al., *Plasma collection by on-line membrane plasmapheresis with plasmax.* Trans Am Soc Artif Intern Organs, 1983. **29**: p. 468-74.

332. Konovalov, G.A., et al., *Methodology of plasmapheresis in the treatment of patients with hereditary hypercholesterolemia.* Kardiologiia, 1986. **26**(10): p. 42-44.

333. Konovalov, G.A., et al., *[Methodology of plasmapheresis in the treatment of patients with hereditary hypercholesterolemia].* Kardiologiia, 1986. **26**(10): p. 42-4.

334. Körbling, M., et al., *Albumin density gradient purification of canine hemopoietic blood stem cells (HBSC): long-term allogeneic engraftment without GVH-reaction.* Exp Hematol, 1979. **7**(6): p. 277-88.

335. Kotenko, S.I. and I. Lisunkin Iu, *[Use of polymers in medicine].* Farm Zh, 1971. **26**(1): p. 17-24.

336. Koziner, V.B., V.M. Korolev, and V.S. Iarochkin, *[Effectiveness of polyglucin, gelatinol and plasma in the treatment of hemorrhage].* Probl Gematol Pereliv Krovi, 1978. **23**(2): p. 25-30.

337. Kozlowski, C.L., et al., *Lung cancer, immune thrombocytopenia and the lupus inhibitor.* Postgrad Med J, 1987. **63**(743): p. 793-5.

338. Krajewski, T., P. Nowak, and C.S. Cierniewski, *Chemical structure and properties of duck and goose fibrinogen.* Biochim Biophys Acta, 1980. **622**(1): p. 94-104.

339. Krakauer, R.S., et al., *Therapeutic trial of cryofiltration in patients with rheumatoid arthritis.* American Journal of Medicine, 1983. **74**(6): p. 951-955.

340. Krause, M., et al., *[Microangiopathic hemolytic anemias. Clinical pattern, therapy and clinical course in 14 patients with thrombotic thrombocytopenic purpura and hemolytic uremic syndrome].* Schweiz Med Wochenschr, 1986. **116**(48): p. 1666-74.

341. Krstulović, A.M., et al., *Plasma catecholamines in hypertension and pheochromocytoma determined using ion-pair reversed-phase chromatography with amperometric detection: investigation of the separation mechanism and clinical methodology.* J Chromatogr, 1981. **217**: p. 523-37.

342. Kulikov, V.I. and L.D. Bergel'son, *[Binding of 1-0-alkyl-2-0-acetyl-sn-glycero-3-phosphocholine (thrombocyte activating factor) by plasma components. Exchange of the thrombocyte activating factor between lipoproteins and thrombocytes].* Biokhimiia, 1984. **49**(8): p. 1310-5.

343. Künzer, W., *[Blood coagulation and its disorders in the newborn].* Klin Wochenschr, 1971. **49**(1): p. 1-13.

344. Kurtz, S.R., P.M. Carey, and M. McGill, *Evaluation of a new microporous filtration membrane system for therapeutic plasma exchange.* Vox Sanguinis, 1987. **53**(2): p. 89-95.

345. Kurtz, S.R., et al., *Evaluation of a new microporous filtration membrane system for therapeutic plasma exchange.* Vox Sang, 1987. **53**(2): p. 89-95.

346. Kurz, C.S., L. Heilmann, and H. Ludwig, *[Effect of low molecular dextran on maternal microrheologic parameters, fetal heart rate and transcutaneous fetal oxygen partial pressure in labor].* Z Geburtshilfe Perinatol, 1985. **189**(1): p. 25-33.

347. Kutushev, F.K. and V.V. Chalenko, *[Progressive and nonprogressive tendencies in the treatment of endotoxemia].* Vestn Khir Im I I Grek, 1988. **141**(7): p. 80-3.

348. Kuznetsova, O.P., et al., *[Treatment of acute glomerulonephritis in middle-aged patients].* Ter Arkh, 1987. **59**(8): p. 128-31.

349. Kuznetsova, O.P., P.A. Vorobiev, and L.I. Dvoretsky, *Management of acute glomerulonephritis in middle-aged patients.* Terapevticheskii Arkhiv, 1987. **59**(8): p. 128-131.

350. Kvasnicka, J., J. Svejda, and J. Rennerova, *Exchange plasmapheresis and heparin in treatment of coma in viral hepatitis.* Vnitrni Lekarstvi, 1978. **24**(5): p. 440-444.

351. Kvasnicka, J., et al., *[Exchange plasmapheresis and heparin in the treatment of coma in viral hepatitis].* Vnitr Lek, 1978. **24**(5): p. 440-4.

352. Kwaan, H.C., *Thrombotic thrombocytopenic purpura.* Journal of the American Medical Association, 1982. **247**(22): p. 3119-3120.

353. Kwaan, H.C., *Thrombotic thrombocytopenic purpura and hemolytic uremic syndrome in pregnancy.* Clin Obstet Gynecol, 1985. **28**(1): p. 101-6.

354. La Celle, P.L. and R.I. Weed, *The contribution of normal and pathologic erythrocytes to blood rheology.* Prog Hematol, 1971. **7**(0): p. 1-31.

355. Labat, J., et al., *A sialic acid-free beta 1-glycoprotein of normal human plasma.* J Biol Chem, 1969. **244**(18): p. 4975-80.

356. Lambert, P.P., et al., *The basic proteins of bovine allantoic fluid.* Biol Reprod, 1987. **37**(4): p. 887-99.

357. Lämmle, B., T.H. Tran, and F. Duckert, *Assay of Factor XII clotting activity in heparinized plasma.* Am J Clin Pathol, 1983. **80**(4): p. 474-7.

358. Landin, B. and A. Nilsson, *Metabolism of chylomicron phosphatidylethanolamine in the rat.* Biochim Biophys Acta, 1984. **793**(1): p. 105-13.

359. Landthaler, M., *[Cryoproteinemias].* Hautarzt, 1980. **31**(12): p. 633-8.

360. Lane, D.A., et al., *Antithrombin III Northwick Park: demonstration of an inactive high MW complex with increased affinity for heparin.* Br J Haematol, 1987. **65**(4): p. 451-6.

361. Lane, T.A. and E.R. Burka, *Decreased life span and membrane damage of carbamylated erythrocytes in vitro.* Blood, 1976. **47**(6): p. 909-17.

362. Latini, R., G. Tognoni, and R.E. Kates, *Clinical pharmacokinetics of amiodarone.* Clin Pharmacokinet, 1984. **9**(2): p. 136-56.

363. Laupacis, A., et al., *Intraarterial methylprednisolone and heparin (IAT) for the treatment of refractory renal transplant rejection episodes.* Transplantation Proceedings, 1982. **14**(4): p. 693-695.

364. Le Grimellec, C., G. Friedlander, and M.C. Giocondi, *Asymmetry of plasma membrane lipid order in Madin-Darby Canine Kidney cells.* Am J Physiol, 1988. **255**(1 Pt 2): p. F22-32.

365. Lehmann, H., *[Therapy of inflammatory vascular diseases].* Dtsch Med Wochenschr, 1981. **106**(12): p. 370-2.

366. Lemaire, F., et al., *[Estimation of intrapulmonary shunt in resuscitation (use of a nomogram) (author's transl)].* Bull Physiopathol Respir (Nancy), 1975. **11**(5): p. 659-81.

367. Lemieux, G., et al., *The effect of ketone bodies on renal ammoniogenesis.* J Clin Invest, 1971. **50**(9): p. 1781-91.

368. Lemon, G.J., et al., *Transcapillary exchange and retention of fluoride, strontium, EDTA, sucrose, and antipyrine in bone.* Calcif Tissue Int, 1980. **31**(2): p. 173-81.

369. Letendre, E.D. and B.E. Holbein, *A sensitive and convenient assay procedure for transferrin and its application to the purification of mouse transferrin.* Can J Biochem, 1981. **59**(11-12): p. 906-10.

370. Levin, M., et al., *A highly cationic protein in plasma and urine of children with steroid-responsive nephrotic syndrome.* Kidney Int, 1989. **36**(5): p. 867-77.

371. Levin, M., et al., *Goodpasture's syndrome: treatment with plasmapheresis, immunosuppression, and anticoagulation.* Arch Dis Child, 1983. **58**(9): p. 697-702.

372. Levin, M., S.P.A. Rigden, and J.R. Pincott, *Goodpasture's syndrome: Treatment with plasmapheresis, immunosuppression, and anticoagulation.* Archives of Disease in Childhood, 1983. **58**(9): p. 697-702.

373. Levy, R.J. and J.B. Lian, *gamma-Carboxyglutamate excretion and warfarin therapy.* Clin Pharmacol Ther, 1979. **25**(5 Pt 1): p. 562-70.

374. Leypoldt, J.K., et al., *Dialysate to blood transport of macromolecules during peritoneal dialysis.* Am J Physiol, 1989. **257**(6 Pt 2): p. H1851-9.

375. Liapis, M.A., *[Immunotherapy of breast cancer (review of foreign literature)].* Vopr Onkol, 1983. **29**(8): p. 68-74.

376. Lieberman, J.D. and S. Schatten, *Treatment. Disease-modifying therapies.* Rheum Dis Clin North Am, 1988. **14**(1): p. 223-43.

377. Liebert, A., D. Quietzsch, and S. Zimmermann, *[Plasma exchange therapy (plasmapheresis/plasma filtration). I. Technic].* Z Gesamte Inn Med, 1984. **39**(16): p. 381-3.

378. Lietti, D., P. Cantone, and E. Grasso, *Therapy of vasculitis in childhood.* Pediatria Medica e Chirurgica, 1989. **11**(SUPPL. 1): p. 63-72.

379. Lionetti, F.J., et al., *Preservation of human granulocytes. II. Characteristics of granulocytes obtained by counterflow centrifugation.* Transfusion, 1977. **17**(5): p. 465-72.

380. Lippe, G., et al., *3-Hydroxy-3-methylglutaric, adipic, and 2-oxoglutaric acids measured by HPLC in the plasma from diabetic patients.* Clin Biochem, 1987. **20**(4): p. 275-9.

381. Litvinenko Iu, A., I.R. Kolonina, and N.A. Gorbunova, *[An experimental study of the water balance and acid-base state of the blood during infusions of the plasma substitute oksiamal].* Gematol Transfuziol, 1989. **34**(12): p. 25-8.

382. Litwiller, R.D., et al., *Monoclonal antibodies to human vitamin K-dependent protein S.* Blood, 1986. **67**(6): p. 1583-90.

383. Liu, E.T., C.A. Linker, and M.A. Shuman, *Management of treatment failures in thrombotic thrombocytopenic purpura.* Am J Hematol, 1986. **23**(4): p. 347-61.

384. Lloyd-Davies, K.A., R.H. Michell, and R. Coleman, *Glycerylphosphorylcholine phosphodiesterase in rat liver. Subcellular distribution and localization in plasma membranes.* Biochem J, 1972. **127**(2): p. 357-68.

385. Lockwood, C.M., et al., *Immunosuppression and plasma exchange in the treatment of Goodpasture's syndrome.* Lancet, 1976. **1**(7962): p. 711-715.

386. Long, R., et al., *Treatment of canine aspiration pneumonitis: Fluid volume reduction vs. fluid volume expansion.* Journal of Applied Physiology, 1988. **65**(4): p. 1736-1744.

387. Loughrey, J.R. and R.L. Meyer, *Plasmapheresis in hyperviscosity syndrome--a better way?* Jama, 1974. **229**(9): p. 1211.

388. Lowenthal, R.M. and D.S. Park, *The use of dextran as an adjunct to granulocyte collection with the continuous-flow blood cell separator.* Transfusion, 1975. **15**(1): p. 23-7.

389. Lundsgaard-Hansen, P., *Donor safety in plasmapheresis.* Dev Biol Stand, 1980. **48**: p. 287-95.

390. Lundsgaard-Hansen, P. and K. Deubelbeiss, *Computer simulation studies of therapeutic plasmapheresis.* Acta Haematologica Polonica, 1980. **11**(2): p. 117-120.

391. Lupien, P.J., S. Moorjani, and J. Awad, *A new approach to the management of familial hypercholesterolaemia: Removal of plasma-cholesterol based on the principle of affinity chromatography.* Lancet, 1976. **1**(7972): p. 1261-5.

392. Mabuchi, H., et al., *A new low density lipoprotein apheresis system using two dextran sulfate cellulose columns in an automated column regenerating unit (LDL continuous apheresis).* Atherosclerosis, 1987. **68**(1-2): p. 19-25.

393. Maeda, K., et al., *Plasma exchange for treatment of intractable psoriasis.* Artif Organs, 1983. **7**(4): p. 450-3.

394. Malchesky, P.S., *Immunomodulation: bioengineering aspects.* Artif Organs, 1986. **10**(2): p. 128-34.

395. Malchesky, P.S. and Y. Nosè, *Biomodulation effects of extracorporeal circulation in apheresis.* Semin Hematol, 1989. **26**(2 Suppl 1): p. 42-51.

396. Malchesky, P.S., et al., *The International Apheresis Registry: results of 1983 second pilot.* Artif Organs, 1987. **11**(2): p. 173-82.

397. Malinconico, S.M., J.B. Katz, and A.Z. Budzynski, *Hementin: anticoagulant protease from the salivary gland of the leech Haementeria ghilianii.* J Lab Clin Med, 1984. **103**(1): p. 44-58.

398. Mannhalter, C., *Purification of plasma protein.* Haemostasis, 1988. **18 Suppl 1**: p. 115-9.

399. Mannucci, P.M., et al., *Decrease and rapid recovery of protein C after plasma exchange.* Transfusion, 1986. **26**(2): p. 156-8.

400. Mannucci, P.M., et al., *Enhanced proteolysis of plasma von Willebrand factor in thrombotic thrombocytopenic purpura and the hemolytic uremic syndrome.* Blood, 1989. **74**(3): p. 978-83.

401. Manzano, L., et al., *[Plasmapheresis and heparin-induced thrombocytopenia-thrombosis].* Med Clin (Barc), 1989. **92**(16): p. 639.

402. Manzano, L., et al., *Plasmaphresis and thrombocytopenia and heparin-induced thrombosis.* Medicina Clinica, 1989. **92**(16): p. 639.

403. Marchuk, A.I., S.M. Sapin, and V.V. Golovteev, *[Complications in the performance of plasmapheresis and their prevention].* Gematol Transfuziol, 1987. **32**(10): p. 48-53.

404. Marcus, L., et al., *Extracorporeal removal of specific antibodies by hemoperfusion through the immunosorbent agarose-polyacrolein microsphere beads: removal of anti-bovine serum albumin in animals.* J Biomed Mater Res, 1984. **18**(9): p. 1153-67.

405. Margulis, E. and V.G. Savchenko, *[The results of 5 years' use of a method for heparin cryoprecipitation of plasma proteins (selective plasmapheresis) in patients with an immune-complex pathology].* Ter Arkh, 1989. **61**(7): p. 65-9.

406. Margulis, E., et al., *[Clinical effectiveness of the method of extracorporeal heparin precipitation of plasma proteins (selective plasmapheresis) in patients with immune complex pathology].* Ter Arkh, 1985. **57**(7): p. 125-31.

407. Margulis, E., et al., *[Effect of cryoapheresis on the course of immune complex diseases].* Ter Arkh, 1987. **59**(6): p. 80-5.

408. Margulis, E.I. and V.G. Savchenko, *The results of 5 years' use of a method for heparin cryoprecipitation of plasma proteins (selective plasmapheresis) in patients with an immune-complex pathology.* Terapevticheskii arkhiv, 1989. **61**(7): p. 65-69.

409. Margulis, E.I., et al., *Effect of cryoapheresis on the course of immune complex diseases.* Terapevticheskii arkhiv, 1987. **59**(6): p. 80-85.

410. Margulis Ya, E., V.G. Savchenko, and S.A. Vasilyev, *The clinical efficacy of extracorporeal heparin precipitation of plasma proteins (selective plasmapheresis) in patients with immune complex pathology.* Terapevticheskii Arkhiv, 1985. **57**(7): p. 125-131.

411. Marini, C., et al., *Fibrinolytic effects of urokinase and heparin in acute pulmonary embolism: a randomized clinical trial.* Respiration, 1988. **54**(3): p. 162-73.

412. Markestad, T., et al., *Effect of exchange transfusions with citrated blood on plasma concentrations of vitamin D metabolites in neonates.* Pediatr Res, 1984. **18**(5): p. 429-31.

413. Markofsky, J. and N. Orentreich, *An improved method for repeated plasmapheresis in the rat.* Lab Anim Sci, 1976. **26**(1): p. 93-5.

414. Markova, O.A., V.V. Kalashnikov, and V.B. Khvatov, *[Isolation and characteristics of antithrombin III from human plasma].* Vopr Med Khim, 1987. **33**(3): p. 62-6.

415. Marlar, R.A. and J.H. Griffin, *Deficiency of protein C inhibitor in combined factor V/VIII deficiency disease.* J Clin Invest, 1980. **66**(5): p. 1186-9.

416. Martin, B.R., T. Clausen, and J. Gliemann, *Relationships between the exchange of calcium and phosphate in isolated fat-cells.* Biochem J, 1975. **152**(1): p. 121-9.

417. Martin Escobar, E., L. Orofino, and L. Orte, *Acute renal failure and multivisceral involvement in rifampicin induced hypersensitivity treated with plasmapheresis.* Nefrologia, 1983. **3**(4): p. 305-309.

418. Mathez, D., J. Roussi, and L. Houbouyan, *A new plasma exchange technique: Coagulation studies.* Nouvelle Revue Francaise d'Hematologie, 1981. **23**(5): p. 285-289.

419. Mathez, D., et al., *[A new plasma exchange technique : coagulation studies (author's transl)].* Nouv Rev Fr Hematol (1978), 1981. **23**(5): p. 285-9.

420. Matsuda, K., et al., *Experimental study on the adsorption of excess heparin with anion exchange resin fiber.* Artif Organs, 1989. **13**(6): p. 504-7.

421. Matsumoto, T., et al., *[Reevaluation of direct radioimmunoassay for plasma cAMP concentration].* Nihon Naibunpi Gakkai Zasshi, 1988. **64**(10): p. 1015-23.

422. McCredie, K.B., et al., *Platelet and leukocyte transfusions in acute leukemia.* Hum Pathol, 1974. **5**(6): p. 699-708.

423. McCullough, J., et al., *Rapid plasma exchange with the continuous flow centrifuge.* Transfusion, 1973. **13**(2): p. 94-9.

424. McLeod, B.C., A. Viernes, and R.J. Sassetti, *Complement activation by plasma separator membranes.* Transfusion, 1983. **23**(2): p. 143-7.

425. McLeod, B.C., A. Viernes, and R.J. Sassetti, *Complement metabolism during membrane plasma separation.* Artif Organs, 1983. **7**(4): p. 443-9.

426. McNeil, H.P., C.N. Chesterman, and S.A. Krilis, *Anticardiolipin antibodies and lupus anticoagulants comprise separate antibody subgroups with different phospholipid binding characteristics.* Br J Haematol, 1989. **73**(4): p. 506-13.

427. Middleton, S.M., I.H. Bennett, and J.K. Smith, *A therapeutic concentrate of coagulation factors II, IX and X from citrated, factor VIII-depleted plasma.* Vox Sang, 1973. **24**(5): p. 441-56.

428. Mielke, C.H., Jr. and M.R. Mielke, *Technical and therapeutic applications of plasma exchange.* Prog Clin Biol Res, 1981. **65**: p. 123-45.

429. Mijailovic, B., et al., *Treatment of porphyria cutanea tarda using plasma exchange.* Vojnosanitetski pregled. Military-medical and pharmaceutical review, 1989. **46**(3): p. 183-186.

430. Mijailović, B., et al., *[Treatment of porphyria cutanea tarda using plasma exchange].* Vojnosanit Pregl, 1989. **46**(3): p. 183-6.

431. Miletich, J.P., G.J. Broze, Jr., and P.W. Majerus, *The synthesis of sulfated dextran beads for isolation of human plasma coagulation factors II, IX, and X.* Anal Biochem, 1980. **105**(2): p. 304-10.

432. Milner, R.D., *Neonatal metabolism and endocrinology studied by exchange transfusion.* Clin Endocrinol Metab, 1976. **5**(1): p. 221-35.

433. Milner, R.D., et al., *Adrenocorticotrophin and glucocorticoid response to exchange transfusion.* Acta Paediatr Scand, 1976. **65**(4): p. 439-4.

434. Milner, R.D., M. Fekete, and R. Assan, *Glucagon, insulin, and growth hormone response to exchange transfusion in premature and term infants.* Arch Dis Child, 1972. **47**(252): p. 186-9.

435. Milner, R.D., et al., *Effect of glucose on plasma glucagon, growth hormone, and insulin in exchange transfusion.* Arch Dis Child, 1972. **47**(252): p. 179-85.

436. Milner, R.D. and J.G. Ratcliffe, *Thyroid function during exchange transfusion.* Arch Dis Child, 1975. **50**(1): p. 40-4.

437. Milner, R.D. and J.S. Woodhead, *Parathyroid hormone secretion during exchange transfusion.* Arch Dis Child, 1975. **50**(4): p. 298-303.

438. Miribel, L. and P. Arnaud, *Purification of human alpha 1-beta glycoprotein and study of its microheterogeneity and molecular variants.* J Chromatogr, 1987. **405**: p. 337-45.

439. Mishler, J.M., *Adverse reactions associated with heparin therapy for ischemic heart disease and thrombophlebitis.* Am J Hosp Pharm, 1973. **30**(12): p. 1158-61.

440. Mishler, J.M., *Plasmapheresis: the in vitro distribution of heparin in plasma and red cell fractions following centrifugation.* Vox Sang, 1975. **28**(3): p. 218-29.

441. Mishler, J.M., et al., *The utilization of a new strength citrate anticoagulant during centrifugal plateletpheresis. II. Assessment of in vitro platelet function.* Blut, 1977. **34**(3): p. 237-41.

442. Mishler, J.M., P. Lund, and H. Borberg, *Plateletpheresis with the IBM model 2997. I. Effects of ACD, NIH formula B on selected donor indices.* Vox Sang, 1980. **38**(1): p. 36-9.

443. Mishler, J.M.t., *Synthetic plasma volume expanders--their pharmacology, safety and clinical efficacy.* Clin Haematol, 1984. **13**(1): p. 75-92.

444. Miyabo, S. and L. Kornel, *Corticosteroids in human blood. VI. Isolation, characterization and quantitation of sulfate conjugated metabolites of cortisol in human plasma.* J Steroid Biochem, 1974. **5**(3): p. 233-47.

445. Mizukoshi, F., et al., *Cytochemical localization of Na-K ATPase in the guinea pig endolymphatic sac.* Acta Otolaryngol, 1988. **105**(3-4): p. 202-8.

446. Moghaddam, M., K.L. Goldsmith, and R.A. Kerwick, *The preparation of blood grouping serum from human citrated plasma.* Vox Sang, 1971. **20**(3): p. 277-80.

447. Monkhouse, F.C. and S. Milojevic, *Studies on the relation between plasma antithrombin and heparin-cofactor.* Can J Physiol Pharmacol, 1968. **46**(3): p. 347-50.

448. Morales, M., et al., *Use of heparin for cytapheresis and plasmapheresis in a continuous flow centrifuge.* Transfusion, 1982. **22**(5): p. 384-387.

449. Morales, M., et al., *Use of heparin for cytapheresis and plasmapheresis in a continuous flow centrifuge.* Transfusion, 1982. **22**(5): p. 384-7.

450. Morales, M.R., et al., *Systematic use of heparin in the continuous flow centrifuge (CFC) for blood cell separation.* Thrombosis and Haemostasis, 1981. **46**(1): p. No.-1197.

451. Morales-Polanco, M.R., et al., *Use of heparin in the continuous flow centrifuge (CFC) during cytapheresis and plasmapheresis.* Archivos de investigacion medica, 1983. **14**(2): p. 93-105.

452. Morales-Polanco, M.R., et al., *Use of heparin in the continuous flow centrifuge (CFC) during cytapheresis and plasmapheresis.* Arch Invest Med (Mex), 1983. **14**(2): p. 93-105.

453. Morse, E.E., et al., *Decreased ionized calcium during therapeutic plasma exchange pheresis and platelet pheresis.* Johns Hopkins Med J, 1980. **146**(6): p. 260-3.

454. Moss, G.S., et al., *Transport of oxygen and carbon dioxide by hemoglobin-saline solution in the red cell-free primate.* Surg Gynecol Obstet, 1976. **142**(3): p. 357-62.

455. Mottaghy, K., et al., *Technical aspects of plasma leakage prevention in microporous capillary membrane oxygenators.* ASAIO Trans, 1989. **35**(3): p. 640-3.

456. Mucklow, J.C., *The fate of drugs in pregnancy.* Clin Obstet Gynaecol, 1986. **13**(2): p. 161-75.

457. Muller, G.A. and T. Risler, *Diagnosis and therapy of different forms of rapidly progressive glomerulonephritis.* Internistische Welt, 1985. **8**(8): p. 239-244.

458. Murray, J.F. and E. Escobar, *Circulatory effects of blood viscosity: comparison of methemoglobinemia and anemia.* J Appl Physiol, 1968. **25**(5): p. 594-9.

459. Murthy, A.R., et al., *The Hypolipidemic Activity of a Series of 2,3-Dihydrophthalazine-l,4-dione Derivatives in Rodents.* Pharm Res, 1986. **3**(2): p. 93-101.

460. Myhre, K. and J.B. Steen, *The effect of plasma proteins on the capillary permeability in the rete mirabile of the eel (Anguilla vulgaris L.).* Acta Physiol Scand, 1977. **99**(1): p. 98-104.

461. Nagai, T., et al., *Characterization of fibronectin metabolites in normal rat urine.* Biochim Biophys Acta, 1988. **967**(2): p. 176-82.

462. Nagasawa, S., K. Takahashi, and J. Koyama, *Isolation of a complex of the subcomponents of the activated first component of complement, Clr--Cls, from ACD-human plasma.* FEBS Lett, 1974. **41**(2): p. 280-2.

463. Nagy, J.A., et al., *Exchange of macromolecules between plasma and peritoneal cavity in ascites tumor-bearing, normal, and serotonin-injected mice.* Cancer Res, 1989. **49**(19): p. 5448-58.

464. Nakamoto, Y., K. Miki, and K. Asakura, *[Progress in the treatment of nephrotic syndrome].* Nihon Rinsho, 1984. **42**(6): p. 1352-62.

465. Nalbandian, R.M. and R.L. Henry, *A proposed comprehensive pathophysiology of thrombotic thrombocytopenic purpura with implicit novel tests and therapies.* Semin Thromb Hemost, 1980. **6**(4): p. 356-90.

466. Nand, S. and J.A. Robinson, *Plasmapheresis in the management of heparin-associated thrombocytopenia with thrombosis.* Am J Hematol, 1988. **28**(3): p. 204-6.

467. Nayler, W.G., J. Dunnet, and A. Sullivan, *Drug-induced changes in the superficially located stores of calcium in heart sarcolemma.* Recent Adv Stud Cardiac Struct Metab, 1976. **9**: p. 53-70.

468. Nelsestuen, G.L., *Vitamin K-dependent plasma proteins.* Methods Enzymol, 1984. **107**: p. 507-16.

469. Nemark, A.I., A.V. Mazyrko, and I.I. Astakhov, *Use of plasmapheresis in multimodality correction of hemostasis disorders in patients with calculous pyelonephritis.* Terapevticheskii arkhiv, 1989. **61**(6): p. 86-88.

470. Nemark, A.I., A.V. Mazyrko, and I. Astakhov Iu, *[Use of plasmapheresis in multimodality correction of hemostasis disorders in patients with calculous pyelonephritis].* Ter Arkh, 1989. **61**(6): p. 86-8.

471. Nestel, P.J., et al., *Changes in the plasma lipoprotein distribution of apolipoproteins C-II, C-III1, C-III2 and apolipoprotein B after heparin-induced lipolysis.* Biochim Biophys Acta, 1982. **712**(1): p. 94-102.

472. Neuman, M.W., et al., *The calcium-buffering phase of bone mineral: some clues to its form and formation.* J Bone Miner Res, 1987. **2**(3): p. 171-81.

473. Nielsen, T.T., J.P. Bagger, and A. Thomassen, *Improved myocardial lactate extraction after propranolol in coronary artery disease: effected by peripheral glutamate and free fatty acid metabolism.* Br Heart J, 1986. **55**(2): p. 140-7.

474. Nigawara, K., et al., *A method for estimation of renin activity in plasma.* Tohoku J Exp Med, 1972. **107**(4): p. 323-36.

475. Nightingale, T.E., *Acute isovolemic anemia in anesthetized chickens.* Am J Physiol, 1976. **231**(5 Pt. 1): p. 1451-6.

476. Nilsson, I.M., et al., *Suppression of secondary antibody response by intravenous immunoglobulin in a patient with haemophilia b and antibodies.* Scand J Haematol, 1983. **30**(5): p. 458-64.

477. Nilsson, T., O. Rudolphi, and B. Cedergren, *Effects of intensive plasmapheresis on the haemostatic system.* Scand J Haematol, 1983. **30**(3): p. 201-6.

478. Novoa, E., W.H. Seegers, and H.I. Hassouna, *Improved procedures for the purification of selected vitamin K-dependent proteins.* Prep Biochem, 1976. **6**(5): p. 307-38.

479. Novotny, F., *New approach to internal therapy of psoriasis.* Casopis Lekaru Ceskych, 1988. **127**(42): p. 1283-1286.

480. Novotny, W.F., et al., *Purification and characterization of the lipoprotein-associated coagulation inhibitor from human plasma.* J Biol Chem, 1989. **264**(31): p. 18832-7.

481. Nusbacher, J., M.L. Scher, and J.L. MacPherson, *Plateletpheresis using the haemonetics model 30 cell separator.* Vox Sang, 1977. **33**(1): p. 9-15.

482. Odievre, M., et al., *[Intravascular coagulation and severe hepatic failure in infants].* Arch Fr Pediatr, 1976. **33**(1): p. 31-6.

483. Odink, J. and A. Brank, *Platelet preservation V. Survival, serotonin uptake velocity, and response to hypotonic stress of fresh and cryopreserved human platelets.* Transfusion, 1977. **17**(3): p. 203-9.

484. Olson, P.R., C. Cox, and J. McCullough, *Laboratory and clinical effects of the infusion of ACD solution during plateletpheresis.* Vox Sang, 1977. **33**(2): p. 79-87.

485. Omokawa, S., et al., *Anticoagulant and membrane effects on humoral and cellular changes during plasmapheresis.* ASAIO Transactions, 1988. **34**(3): p. 404-409.

486. Omokawa, S., et al., *Anticoagulant and membrane effects on humoral and cellular changes during plasmapheresis.* ASAIO Trans, 1988. **34**(3): p. 404-9.

487. Oon, C.J. and J.R. Hobbs, *Clinical applications of the continuous flow blood separator machine.* Clin Exp Immunol, 1975. **20**(1): p. 1-16.

488. Oppenheimer, L., et al., *Colorimetric device for measurement of transvascular fluid flux in blood-perfused organs.* J Appl Physiol (1985), 1987. **62**(1): p. 364-72.

489. O'Reilly, M.J., et al., *Controlled trial of plasma exchange in treatment of Raynaud's syndrome.* Br Med J, 1979. **1**(6171): p. 1113-5.

490. O'Reilly, M.J.G., G. Talpos, and V.C. Roberts, *Controlled trial of plasma exchange in treatment of Raynaud's syndrome.* British Medical Journal, 1979. **1**(6171): p. 1113-1115.

491. Osterud, B., *Meningococcal septicemia: The use of plasmapheresis or blood exchange and how to detect severe endotoxin induced white cell activation.* Scandinavian Journal of Clinical and Laboratory Investigation, 1985. **45**(SUPPL. 178): p. 47-51.

492. Ostlund, R.E., Jr., *Removal of apolipoprotein B from dog whole blood by ex vivo hemoadsorption on antibody-agarose beads.* Artif Organs, 1988. **12**(6): p. 491-6.

493. Overholser, K.A. and T.R. Harris, *Effect of exogenous adenosine on resistance, capillary permeability-surface area and flow in ischemic canine myocardium.* J Pharmacol Exp Ther, 1984. **229**(1): p. 148-53.

494. Owen, M.C., et al., *Mutation of antitrypsin to antithrombin. alpha 1-antitrypsin Pittsburgh (358 Met leads to Arg), a fatal bleeding disorder.* N Engl J Med, 1983. **309**(12): p. 694-8.

495. Owen, M.C., et al., *Molecular characterization of antithrombin Barcelona-2: 47 arginine to cysteine.* Thromb Res, 1989. **55**(4): p. 451-7.

496. Paaske, W.P. and P. Sejrsen, *Transcapillary exchange of 14C-inulin by free diffusion in channels of fused vesicles.* Acta Physiol Scand, 1977. **100**(4): p. 437-45.

497. Pandya, B.V. and A.Z. Budzynski, *Anticoagulant proteases from western diamondback rattlesnake (Crotalus atrox) venom.* Biochemistry, 1984. **23**(3): p. 460-70.

498. Paravicini, D., *[Intra- and postoperative autotransfusion--a quality analysis].* Klin Wochenschr, 1988. **66 Suppl 15**: p. 29-32.

499. Paravicini, D., *Intra- and postoperative autotransfusion - A quality analysis.* Klinische Wochenschrift, 1988. **66**(SUPPL. 15): p. 29-32.

500. Parvy, P.R., J.I. Bardet, and P.P. Kamoun, *EDTA in vacutainer tubes can interfere with plasma amino acid analysis.* Clin Chem, 1983. **29**(4): p. 735.

501. Passow, J., A.A. Pineda, and E.A. Burgstaler, *Responsibilities of the registered nurse in the apheresis laboratory.* J Clin Apher, 1984. **2**(1): p. 1-6.

502. Patten, E., *Therapeutic plasmapheresis and plasma exchange.* Crit Rev Clin Lab Sci, 1986. **23**(2): p. 147-75.

503. Pearl, R.G. and M.H. Rosenthal, *Metabolic alkalosis due to plasmapheresis.* Am J Med, 1985. **79**(3): p. 391-3.

504. Pedersen, R.S., et al., *Meningococcal septicaemia treated with combined plasmapheresis and leucapheresis or with blood exchange.* Br Med J (Clin Res Ed), 1984. **289**(6439): p. 254-5.

505. Peeters, L.L., et al., *Placental transfer of Org 10172, a low-molecular weight heparinoid, in the awake late-pregnant guinea pig.* Thromb Res, 1986. **44**(3): p. 277-83.

506. Penny, A.F., *Plasmapheresis procedure design and operation: A consideration of citrate anticoagulant usage.* Transfusion Science, 1989. **10**(1): p. 51-56.

507. Penny, A.F., et al., *Fractionation of plasma recovered from blood collected by metered anticoagulation.* Transfusion Science, 1989. **10**(4): p. 305-309.

508. Penny, R., P.A. Castaldi, and H.M. Whitsed, *Inflammation and haemostasis in paraproteinaemias.* Br J Haematol, 1971. **20**(1): p. 35-44.

509. Perez, M.C., W.A. Wilson, and E. Scopelitis, *Cyclophosphamide use in a young woman with antiphospholipid antibodies and recurrent cerebrovascular accident.* South Med J, 1989. **82**(11): p. 1421-4.

510. Perry, M.A., et al., *Restricted transport of cationic macromolecules across intestinal capillaries.* Am J Physiol, 1983. **245**(4): p. G568-72.

511. Pertuiset, N. and J.P. Grunfeld, *Acute renal failure in pregnancy.* Baillieres Clin Obstet Gynaecol, 1987. **1**(4): p. 873-90.

512. Pfister, R.R., et al., *The effects of oral or intravenous citrate on the activation of neutrophils in vitro.* Cornea, 1988. **7**(4): p. 244-51.

513. Phadke, K.P. and P. Isbister, *Post-transfusion purpura.* Medical Journal of Australia, 1980. **1**(9): p. 430-432.

514. Phillips, N.C., et al., *Mannosidosis in Angus cattle. The enzymic defect.* Biochem J, 1974. **137**(2): p. 363-71.

515. Pichlmayr, I., A.J. Coburg, and R. Pichlmayr, *[Special problems in anaesthesia of haemodiluted patients (author's transl)].* Anaesthesist, 1976. **25**(4): p. 156-60.

516. Pickert, H., *[Exchange processes between intra- and extravascular space regarding the behavior of dextran as an example for the dynamics of large-molecular substances].* Anaesthesist, 1959. **8**(2): p. 42-5.

517. Pilgrim, G., *[On the possibility of obtaining high value test serums by means of plasmapheresis].* Folia Haematol Int Mag Klin Morphol Blutforsch, 1966. **85**(4): p. 349-56.

518. Pineda, A.A., *Methods for selective removal of plasma constituents.* Prog Clin Biol Res, 1982. **106**: p. 361-73.

519. Pini, M., R. Potí, and A.G. Dettori, *[Thrombotic thrombopenic purpura. Description of 2 cases and review of the literature].* Recenti Prog Med, 1982. **73**(4): p. 429-42.

520. Pogliani, E.M. and E. Cofrancesco, *Thrombotic thrombocytopenic purpura: A review.* Haematologica, 1983. **68**(4): p. 546-557.

521. Pollitt, R.J. and F.A. Jenner, *Enzymatic cleavage of 2-acetamido-1-(beta'-L-aspartamido)-1,2-dideoxy-beta-D-glucose by human plasma and seminal fluid. Failure to detect the heterozygous state for aspartylglycosaminuria.* Clin Chim Acta, 1969. **25**(3): p. 413-6.

522. Porter, P., M.C. Porter, and J.N. Shanberge, *Interaction of heparin with the plasma proteins in relation to its antithrombin activity.* Biochemistry, 1967. **6**(6): p. 1854-63.

523. Pourchez, T., et al., *Use of Permcath (Quinton) catheter in uraemic patients in whom the creation of conventional vascular access for haemodialysis is difficult.* Nephron, 1989. **53**(4): p. 297-302.

524. Pourchez, T., et al., *Use of Permcath (Quinton) catheter in uraemic patients in whom the creation of conventional vascular access for haemodialysis is difficult.* Nephron, 1989. **53**(4): p. 297-302.

525. Preissner, K.T., R. Wassmuth, and G. Müller-Berghaus, *Physicochemical characterization of human S-protein and its function in the blood coagulation system.* Biochem J, 1985. **231**(2): p. 349-55.

526. Priollet, P., N. Baudot, and J.N. Fiessinger, *Treatment of progressive systemic sclerosis.* Annales de Dermatologie et de Venereologie, 1984. **111**(6-7): p. 595-607.

527. Prograis, L.J., Jr., et al., *Purification of C1 inhibitor. A new approach for the isolation of this biologically important plasma protease inhibitor.* J Immunol Methods, 1987. **99**(1): p. 113-22.

528. Prou-Wartelle, O. and C. Blatrix, *A circulating anticoagulant inhibiting the first stage of coagulation.* Sang, 1959. **30**(4): p. 378-395.

529. Puig, L., et al., *Adverse effects secondary to the treatment with plasma exchange.* Int J Artif Organs, 1985. **8**(3): p. 155-8.

530. Quietzsch, D., A. Liebert, and S. Zimmermann, *[Plasma exchange therapy (plasmapheresis/plasmafiltration). II. Indications and complications].* Z Gesamte Inn Med, 1984. **39**(20): p. 493-500.

531. Quietzsch, D., A. Liebert, and S. Zimmermann, *Plasma exchange therapy (plasmapherese/plasma filtration) 2nd part. Indications, complications.* Zeitschrift fur die Gesamte Innere Medizin und Ihre Grenzgebiete, 1984. **39**(20): p. 493-500.

532. Rabiner, S.F., *Hemoglobin solution as a plasma expander.* Fed Proc, 1975. **34**(6): p. 1454-7.

533. Radtke, K.P., T.W. Stief, and N. Heimburger, *A new and simple isolation procedure for human protein C inhibitor. Evidence for a second inhibitor for activated protein C present in human plasma.* Biol Chem Hoppe Seyler, 1988. **369**(9): p. 965-74.

534. Rák, K., *Problems of thrombocyte transfusion.* Ther Hung, 1968. **16**(2): p. 47-53.

535. Rao, A.K., et al., *The hemostatic system in children undergoing intensive plasma exchange.* J Pediatr, 1982. **100**(1): p. 69-75.

536. Rao, L.V. and S.P. Bajaj, *Purification of human factor VII utilizing O-(diethylaminoethyl)-Sephadex and Sulfopropyl-Sephadex chromatography.* Anal Biochem, 1984. **136**(2): p. 357-61.

537. Raschke, E., *Postoperative insufficiency of the liver.* Langenbecks Archiv fur Chirurgie, 1972. **332**(KONGR. B): p. 793-799.

538. Read, M.S., R.W. Shermer, and K.M. Brinkhous, *Venom coagglutinin: an activator of platelet aggregation dependent on von Willebrand factor.* Proc Natl Acad Sci U S A, 1978. **75**(9): p. 4514-8.

539. Rechthand, E., Q.R. Smith, and S.I. Rapoport, *A compartmental analysis of solute transfer and exchange across blood-nerve barrier.* Am J Physiol, 1988. **255**(2 Pt 2): p. R317-25.

540. Reding, R., et al., *Plasma exchange technique in the unheparinized, unanaesthetized rat.* Lab Anim, 1988. **22**(4): p. 293-6.

541. Reed, P.W., *Effects of divalent cation ionophore A23187 on potassium permeability of rat erythrocytes.* J Biol Chem, 1976. **251**(11): p. 3489-94.

542. Remuzzi, G., et al., *Haemolytic-uraemic syndrome: deficiency of plasma factor(s) regulating prostacyclin activity?* Lancet, 1978. **2**(8095): p. 871-2.

543. Richmond, J.M., et al., *Thrombotic thrombocytopaenic purpura and anuria: response to plasma exchange.* Australian and New Zealand Journal of Medicine, 1980. **10**(1): p. 48-50.

544. Richner, J., et al., *Thrombotic thrombocytopenic purpura: report on 5 cases and current therapy concept.* Schweizerische Medizinische Wochenschrift, 1981. **111**(11): p. 375-380.

545. Richter, E.A., et al., *Insulin action in human thighs after one-legged immobilization.* J Appl Physiol (1985), 1989. **67**(1): p. 19-23.

546. Richter, W.O., K. Vierneisel, and P. Schwandt, *Extracorporeal LDL elimination with immunoabsorption or heparin precipitation: a comparison in 10 patients.* Beitr Infusionsther, 1988. **23**: p. 127-31.

547. Riesen, W.F., et al., *[Selective elimination of atherogenic lipoproteins using dextran-sulfate cellulose. Experiences in 2 patients with heterozygote familial hypercholesterolemia].* Schweiz Med Wochenschr, 1989. **119**(2): p. 55-8.

548. Rifle, G., et al., *Treatment of idiopathic acute crescentic glomerulonephritis by immunodepression and plasma-exchanges. A prospective randomised study.* Proc Eur Dial Transplant Assoc, 1981. **18**: p. 493-502.

549. Rindi, G., L. De Giuseppe, and G. Sciorelli, *Thiamine monophosphate, a normal constituent of rat plasma.* J Nutr, 1968. **94**(4): p. 447-54.

550. Ritland, S., et al., *Effect of treatment with a bile-sequestering agent (Secholex) on intestinal absorption, duodenal bile acids, and plasma lipids.* Scand J Gastroenterol, 1975. **10**(8): p. 791-800.

551. Roberts, H.R., G.D. Penick, and K.M. Brinkhous, *INTENSIVE PLASMA THERAPY IN THE HEMOPHILIAS.* Jama, 1964. **190**: p. 546-8.

552. Roberts, T.K., J.C. Boursnell, and A.D. Brown, *The rôle of zinc in promoting the opalescence and cold precipitation of boar seminal plasma. II. Relationship of a zinc-precipitable protein with the haemagglutinin.* J Reprod Fertil, 1974. **37**(2): p. 373-86.

553. Roberts, W.H., R.E. Domen, and M.I. Walters, *Changes in calcium distribution during therapeutic plasmapheresis.* Arch Pathol Lab Med, 1984. **108**(11): p. 881-3.

554. Robinson, A.E., et al., *Pilot study for large-scale plasma procurement using automated plasmapheresis.* Vox Sang, 1983. **44**(3): p. 143-50.

555. Robinson, J., M. Viti, and M. Höök, *Structure and properties of an under-sulfated heparan sulfate proteoglycan synthesized by a rat hepatoma cell line.* J Cell Biol, 1984. **98**(3): p. 946-53.

556. Rock, G. and A. Figueredo, *Metabolic changes during platelet storage.* Transfusion, 1976. **16**(6): p. 571-9.

557. Rock, G., N. McCombie, and P. Tittley, *A new technique for the collection of plasma: machine plasmapheresis.* Transfusion, 1981. **21**(3): p. 241-6.

558. Rock, G., et al., *Formation of a cryogel during processing of cell-free plasma.* Transfusion, 1989. **29**(2): p. 165-9.

559. Rock, G., P. Titley, and N. McCombie, *Plasma collection using an automated membrane device.* Transfusion, 1986. **26**(3): p. 269-271.

560. Rock, G., P. Tittley, and V. Fuller, *Effect of citrate anticoagulants on factor VIII levels in plasma.* Transfusion, 1988. **28**(3): p. 248-52.

561. Rock, G., P. Tittley, and N. McCombie, *Plasma collection using an automated membrane device.* Transfusion, 1986. **26**(3): p. 269-71.

562. Rockel, A., W. Romen, and A. Heidland, *New aspects in treatment of rapidly progressive glomerulonephritis.* Deutsche Medizinische Wochenschrift, 1982. **107**(40): p. 1521-1525.

563. Röckel, A., W. Romen, and A. Heidland, *[Therapy of rapidly progressive glomerulonephritis].* Dtsch Med Wochenschr, 1982. **107**(40): p. 1521-5.

564. Rodnitzky, R.L. and J.A. Goeken, *Complications of plasma exchange in neurological patients.* Arch Neurol, 1982. **39**(6): p. 350-4.

565. Rodrigues De Miranda, J.F., *A method for the quantitative determination of the total concentration of radiopaque agents in plasma.* J Pharm Pharmacol, 1967. **19**(3): p. 161-6.

566. Rosenkvist, J., et al., *Harvesting of granulocytes for transfusion therapy by haemonetics 30. Yield, morphology and in vitro functions of collected granulocytes.* Scand J Haematol, 1978. **20**(5): p. 453-60.

567. Rosner, W., N.P. Christy, and W.G. Kelly, *Partial purification and preliminary characterization of estrogen-binding globulins from human plasma.* Biochemistry, 1969. **8**(7): p. 3100-8.

568. Rossi, E.C., F.A. Carone, and F. del Greco, *Platelets and the hemolytic-uremic syndrome.* Ann Clin Lab Sci, 1981. **11**(3): p. 269-73.

569. Roth, B., et al., *Deficiency of antithrombin III in children with hemolytic-uremic syndrome.* Eur J Pediatr, 1984. **142**(1): p. 16-20.

570. Roujeau, J.C., J.C. Guillaume, and P. Morel, *Plasma exchange in bullous pemphigoid.* Lancet, 1984. **2**(8401): p. 486-489.

571. Rouvier, B., et al., *[Treatment of fulminant falciparum malaria with erythrapheresis].* Ann Fr Anesth Reanim, 1988. **7**(3): p. 257-60.

572. Rovamo, L., et al., *Postheparin plasma lipoprotein and hepatic lipases in preterm neonates.* Pediatr Res, 1984. **18**(11): p. 1104-7.

573. Rovamo, L., et al., *Postheparin plasma lipase activities and plasma lipoproteins in newborn infants.* Pediatr Res, 1984. **18**(7): p. 642-7.

574. Roy, A.J., A. Brivkalns, and R.A. Yankee, *Use of postpheresis plasma to improve granulocyte yields for transfusion.* Blood, 1975. **45**(3): p. 345-53.

575. Ruberto, G., et al., *Plasmapheresis in the treatment of autoimmune haemolytic anaemias.* Haematologica, 1979. **64**(6): p. 759-765.

576. Rubin, R., J. Niemetz, and S. Estren, *Use of animal AHG concentrates (factor VIII) in the treatment of life-threatening hemorrhage in patients with factor VIII antibodies.* Ann N Y Acad Sci, 1975. **240**: p. 362-9.

577. Ruenwongsa, P. and M. Chulavatnatol, *A new acidic protease in human seminal plasma.* Biochem Biophys Res Commun, 1974. **59**(1): p. 44-50.

578. Rutili, G., J.C. Parker, and A.E. Taylor, *Fluid balance in ANTU-injured lungs during crystalloid and colloid infusions.* J Appl Physiol Respir Environ Exerc Physiol, 1984. **56**(4): p. 993-8.

579. Ryan, P.F.J., I.A. Cooper, and B.G. Firkin, *Plasmapheresis in the treatment of thrombotic thrombocytopenic purpura: A report of five cases.* Medical Journal of Australia, 1979. **1**(3): p. 69-72.

580. Rybakov, A.I., V.S. Kulikova, and N.V. Terechova, *[General factors in the pathogenesis of parodontosis and chronic recurring aphthous stomatitis].* Zahn Mund Kieferheilkd Zentralbl, 1975. **63**(5): p. 435-41.

581. Ryzhko, V.V., V.M. Gorodetskii, and B.A. Borisov, *Intensive plasmapheresis--possible difficulties and complications.* Terapevticheskii arkhiv, 1987. **59**(6): p. 70-75.

582. Ryzhko, V.V., V.M. Gorodetskií, and B.A. Borisov, *[Intensive plasmapheresis--possible difficulties and complications].* Ter Arkh, 1987. **59**(6): p. 70-5.

583. Sakurai, T., J.P. Boissel, and H.F. Bunn, *Non-enzymatic glycation of antithrombin III in vitro.* Biochim Biophys Acta, 1988. **964**(3): p. 340-7.

584. Salem, H.H., et al., *Isolation and characterization of thrombomodulin from human placenta.* J Biol Chem, 1984. **259**(19): p. 12246-51.

585. Samtleben, W., et al., *Membrane plasma exchange: principles and application techniques.* J Clin Apher, 1984. **2**(2): p. 163-9.

586. Sandler, S.G. and J. Nusbacher, *Health risk of leukapheresis donors.* Haematologia (Budap), 1982. **15**(1): p. 57-69.

587. Savchenko, V.G., et al., *[Effect of the method of extracorporeal heparin precipitation of plasma proteins (selective plasmapheresis) on the level of immune complexes in the blood].* Ter Arkh, 1985. **57**(7): p. 102-7.

588. Savchenko, V.G., et al., *Effect of the method of extracorporeal heparin precipitation of plasma proteins (selective plasmapheresis) on the level of immune complexes in the blood.* Terapevticheskii arkhiv, 1985. **57**(7): p. 102-107.

589. Scherf, H. and K. Hausmann, *Diagnosis of thrombotic thrombocytopenic purpura.* Deutsche Medizinische Wochenschrift, 1982. **107**(26): p. 1024-1026.

590. Schick, B.P. and P.K. Schick, *Cholesterol exchange in platelets, erythrocytes and megakaryocytes.* Biochim Biophys Acta, 1985. **833**(2): p. 281-90.

591. Schiffer, C.A., J. Aisner, and P.H. Wiernik, *Transient neutropenia induced by transfusion of blood exposed to nylon fiber filters.* Blood, 1975. **45**(1): p. 141-6.

592. Schiffer, C.A., et al., *Reversal of granulocyte adherence to nylon fibers using local anesthetic agents: possible application to filtration leukapheresis.* Blood, 1977. **50**(2): p. 213-25.

593. Schindhelm, K., C.G. Roberts, and J.E. Moran, *Plasmapheresis in nephrotoxic serum nephritis.* Plasma Therapy and Transfusion Technology, 1985. **6**(3): p. 641-645.

594. Schleuning, W.D., M. Sudol, and E. Reich, *A proenzyme from chicken plasma similar to human plasma prekallikrein.* J Biol Chem, 1983. **258**(23): p. 14106-15.

595. Schmidt, B., et al., *Plasma elimination of antithrombin III (heparin cofactor activity) is accelerated in term newborn infants.* Eur J Pediatr, 1984. **141**(4): p. 225-7.

596. Schneider, P.A., et al., *The role of splenectomy in multimodality treatment of thrombotic thrombocytopenic purpura.* Ann Surg, 1985. **202**(3): p. 318-22.

597. Schoengen, A., et al., *Cryocrystalglobulinemia: pH-dependent precipitation of a monoclonal IgG-kappa-immunoglobulin.* Blut, 1989. **58**(5): p. 255-60.

598. Schooneman, F. and F. Streiff, *Use of a new plasma separation membrane for plasma donation. Technical and biological results.* Artif Organs, 1988. **12**(6): p. 526-9.

599. Schuff-Werner, P., et al., *Treatment of severe hypercholesterolemia by heparin-induced extracorporeal LDL precipitation (HELP).* Beitr Infusionsther, 1988. **23**: p. 118-26.

600. Schuff-Werner, P., et al., *Improved haemorheology associated with a reduction in plasma fibrinogen and LDL in patients being treated by heparin-induced extracorporeal LDL precipitation (HELP).* European Journal of Clinical Investigation, 1989. **19**(1): p. 30-37.

601. Schuler, J. and A. von Felten, *[Simple method for controlling anticoagulation with vitamin K antagonists during heparin therapy: rapid determination after heparin absorption by ECTEOLA cellulose].* Schweiz Med Wochenschr, 1982. **112**(49): p. 1798-800.

602. Schumpelick, V. and R.W. Janzen, *[Thymectomy in myasthenia gravis].* Dtsch Med Wochenschr, 1984. **109**(30): p. 1166-72.

603. Schwartz, C.C., et al., *Central role of high density lipoprotein in plasma free cholesterol metabolism.* J Clin Invest, 1982. **70**(1): p. 105-16.

604. Schwartz, R.S., et al., *Interaction of phosphatidylserine-phosphatidylcholine liposomes with sickle erythrocytes. Evidence for altered membrane surface properties.* J Clin Invest, 1983. **71**(6): p. 1570-80.

605. Schwartzkopff, W., et al., *[Kinetics of lipids and lipoproteins with determination of the recovery rate in the non-steady state following plasma, membrane filtration and dextran sulfate adsorption apheresis in hypercholesterolemia].* Biomed Tech (Berl), 1989. **34**(10): p. 232-42.

606. Schwartzkopff, W., et al., *Efficacy of plasmapheresis, cascade-filtration and dextran-sulfate adsorption apheresis with calculation of the recovery rate of lipids and lipoproteins.* Beitr Infusionsther, 1988. **23**: p. 103-11.

607. Sebriakova, M. and J.A. Little, *A method for the determination of plasma insulin antibodies and its application in normal and diabetic subjects.* Diabetes, 1973. **22**(1): p. 30-40.

608. Seidel, D., et al., *Removal of low-density lipoproteins (LDL) and fibrinogen by precipitation with heparin at low pH: clinical application and experience.* J Clin Apher, 1988. **4**(2-3): p. 78-81.

609. Seitz, R., et al., *Impaired fibrinolysis and protein C increase after cadaver kidney transplantation.* Thromb Res, 1986. **42**(3): p. 277-88.

610. Serra, A., et al., *Vasculitis affecting the kidney: presentation, histopathology and long-term outcome.* Q J Med, 1984. **53**(210): p. 181-207.

611. Sgouris, J.T., *Advisory committee on serum -globulins. Suitability for clinical use of immune serum globulin prepared from CPD plasma. A brief review.* Vox Sang, 1971. **20**(5): p. 462-4.

612. Sgouris, J.T. and M. Wickerhauser, *Use of frozen cryoprecipitate for the preparation of clinical factor VIII concentrate.* Transfusion, 1973. **13**(6): p. 399-404.

613. Shafrir, E. and T. Brenner, *Lipoprotein lipid and protein synthesis in experimental nephrosis and plasmapheresis. I: Studies in rat in vivo.* Lipids, 1979. **14**(8): p. 695-702.

614. Sheldon, R. and D. Slaughter, *A syndrome of microangiopathic hemolytic anemia, renal impairment, and pulmonary edema in chemotherapy-treated patients with adenocarcinoma.* Cancer, 1986. **58**(7): p. 1428-1436.

615. Sheppard, C.W. and G.E. Beyl, *Cation exchange in mammalian erythrocytes. III. The prolytic effect of x-rays on human cells.* J Gen Physiol, 1951. **34**(5): p. 691-704.

616. Sheppard, C.W. and W.R. Martin, *Cation exchange between cells and plasma of mammalian blood; methods and application to potassium exchange in human blood.* J Gen Physiol, 1950. **33**(6): p. 703-22.

617. Sherman, L.A., *DIC in massive transfusion.* Prog Clin Biol Res, 1982. **108**: p. 171-89.

618. Shigematsu, Y., et al., *Organic acids and branched-chain amino acids in body fluids before and after multiple exchange transfusions in maple syrup urine disease.* J Inherit Metab Dis, 1983. **6**(4): p. 183-9.

619. Shimizu, N., et al., *STUDIES ON THE MELANOTROPIC ACITIVTY OF HUMAN PLASMA AND TISSUES.* J Clin Endocrinol Metab, 1965. **25**: p. 984-90.

620. Shimizu, T., et al., *New closed system using a sterile connection device and preconnected PRP pack for extended storage of apheresis platelet products.* Tohoku J Exp Med, 1988. **155**(3): p. 303-4.

621. Shinoda, A., H. Kitada, and S. Suzuki, *Accessible plasma exchange using membrane filter - A successfully treated case of TTP with repeated plasma exchanges.* Artificial Organs, 1981. **5**(3): p. 248-253.

622. Shiomi, T., et al., *Binding of heparin onto ethylene-vinyl alcohol copolymer membrane.* J Biomed Mater Res, 1988. **22**(3 Suppl): p. 269-80.

623. Shoji, M. and W.R. Vogler, *Effects of hydrocortisone on the yield and bactericidal function of granulocytes collected by continuous-flow centrifugation.* Blood, 1974. **44**(3): p. 435-43.

624. Sideman, S., N. Lotan, and A. Tabak, *Tailor-made agarose-based reactive beads for hemoperfusion and plasma perfusion.* Applied Biochemistry and Biotechnology, 1984. **VOL. 10**: p. 167-182.

625. Sideman, S., et al., *Tailor-made agarose-based reactive beads for hemoperfusion and plasma perfusion.* Appl Biochem Biotechnol, 1984. **10**: p. 167-82.

626. Sieberth, H.G. and N. Maurin, *The therapy of rapidly progressive glomerulonephritis.* Klin Wochenschr, 1983. **61**(20): p. 1001-10.

627. Siekmann, U., et al., *Simultaneous investigations of maternal cardiac output and fetal blood flow during hypervolemic hemodilution in preeclampsia--preliminary observations.* J Perinat Med, 1986. **14**(1): p. 59-69.

628. Silberstein, L.E., et al., *Calcium homeostasis during therapeutic plasma exchange.* Transfusion, 1986. **26**(2): p. 151-5.

629. Sills, R.H., *Thrombotic thrombocytopenic purpura. II. Principles of therapy and guidelines for management.* Am J Pediatr Hematol Oncol, 1984. **6**(4): p. 431-9.

630. Skjønsberg, O.H., et al., *Contaminating fibrin in CPD-blood: solubility in plasma and distribution in blood components following separation.* Thromb Res, 1986. **41**(1): p. 1-8.

631. Smalik, S., N. Halko, and V. Dzavik, *Preparation of thrombocyte concentrates from donors with the use of heparin and intensive plasmapheresis.* Bratislavske lekarske listy, 1972. **58**(4): p. 444-450.

632. Smálik, S., N. Halko, and V. Dzavík, *[Preparation of thrombocyte concentrates from donors with the use of heparin and intensive plasmapheresis].* Bratisl Lek Listy, 1972. **58**(4): p. 444-50.

633. Smolle, K.H. and H. Holzer, *First-line treatment of intoxications.* Wiener Medizinische Wochenschrift, 1986. **136**(5-6): p. 137-142.

634. Soennichsen, N., *Selected therapeutical problems for dermatological practice.* Dermatologische Monatsschrift, 1980. **166**(8): p. 513-522.

635. Sohn, H.E., et al., *[Scrutinization of the direct assay method for plasma cyclic AMP and clinical applications of nephrogenous cyclic AMP].* Nihon Naibunpi Gakkai Zasshi, 1985. **61**(9): p. 912-23.

636. Sontheimer, R.D., *The anticardiolipin syndrome. A new way to slice an old pie, or a new pie to slice?* Arch Dermatol, 1987. **123**(5): p. 590-5.

637. Soute, B.A., M.A. de Boer-vd Berg, and C. Vermeer, *The separation of bovine prothrombin and descarboxyprothrombin by high-performance liquid chromatography.* Anal Biochem, 1984. **137**(1): p. 227-9.

638. Spencer, C.D., et al., *Treatment of postpartum hemolytic uremic syndrome with plasma exchange.* Journal of the American Medical Association, 1982. **247**(20): p. 2808-2809.

639. Spencer, C.D., et al., *Treatment of postpartum hemolytic uremic syndrome.* Journal of the American Medical Association, 1982. **247**(20): p. 2808-2809.

640. Speroff, L., et al., *Hormone levels during prostaglandin F 2 infusions for therapeutic abortion.* J Clin Endocrinol Metab, 1972. **34**(3): p. 531-6.

641. Sprenger, K.B., H. Rasche, and H.E. Franz, *Membrane plasma separation: complications and monitoring.* Artif Organs, 1984. **8**(3): p. 360-3.

642. Sprenger, K.B.G., H. Rasche, and H.E. Franz, *Membrane plasma separation: Complications and monitoring.* Artificial Organs, 1984. **8**(3): p. 360-363.

643. Stafforini, D.M., et al., *Human plasma platelet-activating factor acetylhydrolase. Association with lipoprotein particles and role in the degradation of platelet-activating factor.* J Biol Chem, 1987. **262**(9): p. 4215-22.

644. Stairmand, J.W., et al., *Separation of plasma from whole blood by membrane filtration in oscillatory flows.* Life Support Syst, 1986. **4**(3): p. 193-204.

645. Stefanutti, C., et al., *Selective continuous removal of low density lipoproteins by dextran sulfate cellulose column adsorption apheresis in the therapy of familial hypercholesterolemia.* Beitr Infusionsther, 1988. **23**: p. 172-82.

646. Steffen, C. and R. Seitz, *Severe chlorate poisoning: report of a case.* Arch Toxicol, 1981. **48**(4): p. 281-8.

647. Stein, P. and M. Karl, *[Preparation and conservation of platelet concentrates].* Folia Haematol Int Mag Klin Morphol Blutforsch, 1969. **92**(4): p. 577-81.

648. Steinbrecher, U.P. and P.H. Pritchard, *Hydrolysis of phosphatidylcholine during LDL oxidation is mediated by platelet-activating factor acetylhydrolase.* J Lipid Res, 1989. **30**(3): p. 305-15.

649. Steinbuch, M., et al., *Studies on prothrombin complex concentrates contact factors, complement components and proteinase inhibitors.* Thromb Haemost, 1984. **52**(3): p. 256-62.

650. Steinhorn, R.H., et al., *Hemolysis during long-term extracorporeal membrane oxygenation.* J Pediatr, 1989. **115**(4): p. 625-30.

651. Stenflo, J. and P.O. Ganrot, *Vitamin K and the biosynthesis of prothrombin. I. Identification and purification of a dicoumarol-induced abnormal prothrombin from bovine plasma.* J Biol Chem, 1972. **247**(24): p. 8160-6.

652. Stenger, K.O., et al., *Hemolytic-uremic syndrome associated with an infection by verotoxin producing Escherichia coli 0111 in a woman on oral contraceptives.* Clin Nephrol, 1988. **29**(3): p. 153-8.

653. Stewart, G.N. and T.F. Zucker, *A COMPARISON OF THE ACTION OF PLASMA AND SERUM ON CERTAIN OBJECTS USED IN BIOLOGICAL TESTS FOR EPINEPHRIN.* J Exp Med, 1913. **17**(2): p. 152-73.

654. Stief, T.W., K.P. Radtke, and N. Heimburger, *Inhibition of urokinase by protein C-inhibitor (PCI). Evidence for identity of PCI and plasminogen activator inhibitor 3.* Biol Chem Hoppe Seyler, 1987. **368**(10): p. 1427-33.

655. Stigelman Jr, W.H., et al., *Removal of prednisone and prednisolone by plasma exchange.* Clinical Pharmacy, 1984. **3**(4): p. 402-407.

656. Stoffner, D., et al., *Plasma exchange and concomitant therapy in TTP.* Int J Artif Organs, 1984. **7**(4): p. 223-8.

657. Stoffner, D., F.C.A. Banthien, and R. Habersetzer, *Plasma exchange and concomitant therapy in TTP.* International Journal of Artificial Organs, 1984. **7**(4): p. 223-228.

658. Stowell, C.P., T.F. Scanlin, and M.C. Glick, *Characterization of human fibronectin glycopeptides from cystic fibrosis and control skin fibroblasts.* Carbohydr Res, 1986. **151**: p. 279-92.

659. Strauss, R.G., *In vitro comparison of the erythrocyte sedimenting properties of dextran, hydroxyethyl starch and a new low-molecular-weight hydroxyethyl starch.* Vox Sang, 1979. **37**(5): p. 268-71.

660. Strauss, R.G., et al., *Clinical and laboratory effects on donors of intermittent-flow centrifugation platelet-leukapheresis performed with hydroxyethyl starch and citrate.* Clin Lab Haematol, 1980. **2**(1): p. 1-11.

661. Strobel, E., et al., *Therapeutic lymphapheresis in priapism caused by leukostasis.* Deutsche Medizinische Wochenschrift, 1987. **112**(51-52): p. 1984-1985.

662. Stromberg, R.R., et al., *Development of a novel membrane apheresis system for plasma collection at mobile sites.* ASAIO Trans, 1987. **33**(3): p. 614-20.

663. Stuart, J. and M.W. Kenny, *Blood rheology.* J Clin Pathol, 1980. **33**(5): p. 417-29.

664. Stuby, U., G. Biesenbach, and J. Zazgornik, *[Therapy of severe malaria].* Wien Med Wochenschr, 1989. **139**(9): p. 222-7.

665. Suaudeau, J., et al., *The Ito "Flow-Through" Centrifuge. A new device for long-term (24 hours) plasmapheresis without platelet deterioration.* Transfusion, 1978. **18**(3): p. 312-9.

666. Suaudeau, J., et al., *The Ito 'flow-through' centrifuge. A new device for long-term (24 hours) plasmapheresis without platelet deterioration.* Transfusion, 1978. **18**(3): p. 312-319.

667. Suso, F.A. and H.M. Edwards, Jr., *Ethylenediaminetetraacetic acid and 65 Zn binding by intestinal digesta, intestinal mucosa and blood plasma.* Proc Soc Exp Biol Med, 1971. **138**(1): p. 157-62.

668. Sutton, D.M., R.C. Nair, and G. Rock, *Complications of plasma exchange.* Transfusion, 1989. **29**(2): p. 124-7.

669. Suzuki, K. and K. Scmid, *Basic proteins of Cohn fraction 3 of human plasma.* Arch Biochem Biophys, 1968. **123**(2): p. 421-2.

670. Swier, P., et al., *An in vitro test model to study the performance and thrombogenicity of cardiovascular devices.* ASAIO Trans, 1989. **35**(3): p. 683-7.

671. Tabak, A., et al., *Cholesterol removal by haemoperfusion of whole blood in vivo.* Life Support Syst, 1986. **4**(4): p. 355-65.

672. Takahara, H. and H. Sinohara, *Purification and characterization of rat plasma antithrombin III.* Biochim Biophys Acta, 1980. **612**(1): p. 185-94.

673. Takahashi, H., *Electron microscope studies on the localization of pseudocholinesterase activity in rat liver cells.* Journal of Nara Medical Association, 1969. **20**(1): p. 46-57.

674. Takashima, K., et al., *[Anti-glomerular basement membrane antibody-mediated glomerulonephritis remarkably improved by pulse therapy with methylprednisolone, plasmapheresis and continuous heparin infusion].* Nihon Jinzo Gakkai Shi, 1989. **31**(11): p. 1197-204.

675. Takashima, K., et al., *Anti-glomerular basement membrane antibody-mediated glomerulonephritis remarkably improved by pulse therapy with methylpredhisolone, plasma pherisis and continuous heparin infusion.* Japanese Journal of Nephrology, 1989. **31**(11): p. 1197-1204.

676. Tall, A.R., L.R. Forester, and G.L. Bongiovanni, *Facilitation of phosphatidylcholine transfer into high density lipoproteins by an apolipoprotein in the density 1.20-1.26 g/ml fraction of plasma.* J Lipid Res, 1983. **24**(3): p. 277-89.

677. Tan, M.H., *The lipoprotein lipase system: new understandings.* Can Med Assoc J, 1978. **118**(6): p. 675-80.

678. Tanaka, F., H. Komoriyama, and S. Hamabe, *Effect of gabexate mesilate on the anti-coagulant actions during the plasma separation for extracorporeal circulation.* Japanese Journal of Artificial Organs, 1985. **14**(1): p. 390-393.

679. Thaler, E. and G. Kleinberger, *[Hepatic coagulopathy--principles and therapeutic statements].* Leber Magen Darm, 1982. **12**(5): p. 193-7.

680. Thomas, D.B., *Detection and treatment of severe coagulation disturbances in the neonatal period.* Med J Aust, 1974. **1**(24): p. 962-4.

681. Thompson, A.R. and R.B. Counts, *Removal of heparin and protamine from plasma.* J Lab Clin Med, 1976. **88**(6): p. 922-9.

682. Thompson, H.W. and L.J. McCarthy, *Thrombotic thrombocytopenic purpura. Potential benefit of splenectomy after plasma exchange.* Arch Intern Med, 1983. **143**(11): p. 2117-9.

683. Thompson, W.L., Jr., *Hydroxyethyl starch.* Prog Clin Biol Res, 1978. **19**: p. 283-92.

684. Thysell, H., V.A. Oxelius, and M. Norlin, *Successful treatment of hemolytic uremic syndrome and thrombotic thrombocytopenic purpura with fresh frozen plasma and plasma exchange.* Acta Med Scand, 1982. **212**(5): p. 285-8.

685. Tollefsen, D.M., D.W. Majerus, and M.K. Blank, *Heparin cofactor II. Purification and properties of a heparin-dependent inhibitor of thrombin in human plasma.* J Biol Chem, 1982. **257**(5): p. 2162-9.

686. Tollefsen, D.M., M.E. Peacock, and W.J. Monafo, *Molecular size of dermatan sulfate oligosaccharides required to bind and activate heparin cofactor II.* J Biol Chem, 1986. **261**(19): p. 8854-8.

687. Tree, M., *Measurement of plasma renin-substrate in man.* J Endocrinol, 1973. **56**(2): p. 159-71.

688. Trice, J.M., R.S. Pinals, and G.I. Plitman, *Thrombotic thrombocytopenic purpura during penicillamine therapy in rheumatoid arthritis.* Archives of Internal Medicine, 1983. **143**(7): p. 1487-1488.

689. Triplett, D.A. and E.N. Harris, *Antiphospholipid antibodies and reproduction.* Am J Reprod Immunol, 1989. **21**(3-4): p. 123-31.

690. Uldall, R., *Subclavian cannulation for hemodialysis: the present state of the art.* Artif Organs, 1982. **6**(1): p. 73-6.

691. Umdenstock, R., *Pharmacotherapy in children.* Revue de Medecine de Limoges, 1980. **11**(4): p. 249-250.

692. Umlas, J., G. Gauvin, and R. Taff, *Heparin monitoring and neutralization during cardiopulmonary bypass using a rapid plasma separator and a fluorometric assay.* Ann Thorac Surg, 1984. **37**(4): p. 301-3.

693. Umlas, J. and S. Gootblatt, *The use of frozen blood in neonatal exchange transfusion.* Transfusion, 1976. **16**(6): p. 636-40.

694. Uteg, K.H. and K. Tausendfreund, *[Possibilities to elevate the factor VIII yield].* Folia Haematol Int Mag Klin Morphol Blutforsch, 1987. **114**(1): p. 153-69.

695. Valbonesi, M., et al., *Plasma exchange in management of a patient with diffuse necrotizing cutaneous vasculitis.* Vox Sang, 1980. **39**(5): p. 241-5.

696. Valbonesi, M., et al., *Plasma exchange in the management of a patient with diffuse necrotizing cutaneous vasculitis.* Vox Sanguinis, 1980. **39**(5): p. 241-245.

697. Vallejos, C.S., et al., *Biological effects of repeated leukapheresis of patients with chronic myelogenous leukemia.* Blood, 1973. **42**(6): p. 925-33.

698. Van Den Berg, C.J. and A.A. Pineda, *Plasma exchange in the treatment of acute renal failure due to low molecular-weight dextran.* Mayo Clin Proc, 1980. **55**(6): p. 387-9.

699. Van Obberghen-Schilling, E., et al., *alpha-Thrombin-induced early mitogenic signalling events and G0 to S-phase transition of fibroblasts require continual external stimulation.* Embo j, 1985. **4**(11): p. 2927-32.

700. Van Snick, J.L., P.L. Masson, and J.F. Heremans, *The involvement of lactoferrin in the hyposideremia of acute inflammation.* J Exp Med, 1974. **140**(4): p. 1068-84.

701. Vancura, S.J. and R. Clarenburg, *A model for ultracentrifugal quantification of (35-S) bromosulphthalein-binding to plasma proteins in the presence of radioimpurities.* Proc Soc Exp Biol Med, 1975. **148**(3): p. 720-4.

702. Vargo, J.J. and J.H. Joist, *Further evaluation of a heparin neutralizer and its effect on factor IX in normal and coumadin-plasma.* Thromb Res, 1983. **29**(3): p. 281-8.

703. Vartio, T., *Disulfide-bonded polymerization of plasma fibronectin in the presence of metal ions.* J Biol Chem, 1986. **261**(20): p. 9433-7.

704. Vender, J.S., E.B. Matthew, and I.M. Silverman, *Heparin-associated thrombocytopenia: Alternative managements.* Anesthesia and Analgesia, 1986. **65**(5): p. 520-522.

705. Vender, J.S., et al., *Heparin-associated thrombocytopenia: alternative managements.* Anesth Analg, 1986. **65**(5): p. 520-2.

706. Vendeville, B., et al., *Plasma exchange in a rat model of autoimmune glomerulonephritis.* Nephrol Dial Transplant, 1988. **3**(4): p. 405-11.

707. Vesconi, S., et al., *Urokinase treatment for severe neurological complications in a patient with thrombotic thrombocytopenic purpura.* Haemostasis, 1981. **10**(5): p. 289-95.

708. Vialtel, P., F. Chenais, and E. Dechelette, *Adult hemolytic uremic syndrome treated with plasma exchange.* Plasma Therapy and Transfusion Technology, 1980. **1**(4): p. 51-54.

709. Vialtel, P., et al., *[Hemolytic-uremic syndrome of adults successfully treated with massive plasmapheresis (proceedings)].* J Urol Nephrol (Paris), 1979. **85**(4-5): p. 331-2.

710. Villegas, A., J.L. Alvarez-Sala, and D. Espinós, *[Treatment of the hemolytic-uremic syndrome and thrombotic thrombopenic purpura].* Sangre (Barc), 1983. **28**(5): p. 635-41.

711. Vittal, S.B., *Fulminant viral hepatitis and hepatic failure.* Am Fam Physician, 1974. **9**(5): p. 110-4.

712. Vitti, M.P., et al., *[The role of plasmapheresis in the treatment of primary and secondary cryoglobulinemia].* Minerva Med, 1985. **76**(16): p. 793-6.

713. Vogel, W.C. and E.L. Bierman, *Correlation between post-heparin lipase and phospholipase activities in human plasma.* Lipids, 1970. **5**(4): p. 385-91.

714. Vogt, W. and J. Lyncker, *Differentiation between formation, in plasma, of anaphylatoxin and of endogenous pyrogen.* Naunyn Schmiedebergs Arch Pharmakol, 1969. **264**(1): p. 23-31.

715. Von dem Borne Kr, A.E.G., *Autoimmune thrombocytopenia.* Bailliere's Clinical Immunology and Allergy, 1987. **1**(2): p. 269-302.

716. von Finck, M., et al., *[Autotransfusion and plasmapheresis in preparation for surgery. Behavior of coagulation].* Anaesthesist, 1985. **34**(12): p. 675-80.

717. von Wendt, L., et al., *Failure of strychnine treatment during the neonatal period in three Finnish children with nonketotic hyperglycinemia.* Pediatrics, 1980. **65**(6): p. 1166-9.

718. Wadsworth, J.C., D.S. Kronfeld, and C.F. Ramberg, Jr., *Parathyrin and calcium homeostasis in the fetus.* Biol Neonate, 1982. **41**(3-4): p. 101-9.

719. Wagner, G.M., et al., *Red cell vesiculation--a common membrane physiologic event.* J Lab Clin Med, 1986. **108**(4): p. 315-24.

720. Walker, R.G., C. Scheinkestel, and G.J. Becker, *Clinical and morphological aspects of the management of crescentic anti-glomerular basement membrane antibody (anti-GBM) nephritis/Goodpasture's syndrome.* Quarterly Journal of Medicine, 1985. **54**(213): p. 75-89.

721. Warr, T.A., et al., *Human plasma extrinsic pathway inhibitor activity: I. Standardization of assay and evaluation of physiologic variables.* Blood, 1989. **74**(1): p. 201-6.

722. Watson, D.K., et al., *Citrate induced hypocalcaemia during cell separation.* Br J Haematol, 1980. **44**(3): p. 503-7.

723. Watson, D.K., et al., *Citrate induced hypocalcaemia during cell separation.* British Journal of Haematology, 1980. **44**(3): p. 503-507.

724. Wechsler, B., L.T.H. Du, and P. Godeau, *Management of Behcet disease.* Semaine des Hopitaux, 1986. **62**(19): p. 1341-1344.

725. Wechsler, B., D.U. Le Thi Huong, and P. Godeau, *[Medical treatment of Behçet's disease].* J Mal Vasc, 1988. **13**(3): p. 262-9.

726. Wegener, S., et al., *[Status of "blood coagulation active human citrate plasma" within the scope of blood component therapy].* Z Arztl Fortbild (Jena), 1987. **81**(22): p. 1151-4.

727. Wegener, S., et al., *Place of 'coagulation-active human citrate plasma' in blood-component therapy.* Zeitschrift fur Arztliche Fortbildung, 1987. **81**(22): p. 1151-1154.

728. Wegmüller, E., *[Current therapy of nephrotic syndrome].* Schweiz Med Wochenschr, 1984. **114**(11): p. 374-80.

729. Wegmuller, E., M.D. Kazatchkine, and U.E. Nydegger, *Complement activation during extracorporeal blood bypass.* Plasma Therapy and Transfusion Technology, 1983. **4**(4): p. 361-371.

730. Weinberger, M. and C. Chidsey, *Rapid analysis for theophylline in serum by use of high-pressure cation-exchange chromatography.* Clin Chem, 1975. **21**(7): p. 834-7.

731. Weissbach, G., M. Domula, and H. Lenk, *[Hemostasis disorders after transfusions].* Folia Haematol Int Mag Klin Morphol Blutforsch, 1981. **108**(3): p. 345-66.

732. Wenzel, E. and G. Christ, *[The formation of ammonia in citrated plasma following recalcification].* Thromb Diath Haemorrh, 1969. **22**(3): p. 575-6.

733. Werb, Z. and Z.A. Cohn, *Cholesterol metabolism in the macrophage. II. Alteration of subcellular exchangeable cholesterol compartments and exchange in other cell types.* J Exp Med, 1971. **134**(6): p. 1570-90.

734. White, R.L., W.R. Garnett, and J.H. Allen, *Phenytoin removal during plasma exchange.* Journal of Clinical Apheresis, 1987. **3**(3): p. 147-150.

735. White, R.L., et al., *Salicylate removal during plasma exchange in normal volunteers.* Clinical Pharmacy, 1984. **3**(4): p. 396-402.

736. Whiteside, C.I., C.J. Lumsden, and M. Silverman, *In vivo characterization of insulin uptake by dog renal cortical epithelium.* Am J Physiol, 1988. **255**(3 Pt 1): p. E357-65.

737. Wieland, H., et al., *Selective extracorporal removal of low density lipoproteins using heparin precipitation.* Arztliche Laboratorium, 1988. **34**(1): p. 28.

738. Williams, V. and J. White, *Purification and properties of a procoagulant from peninsula tiger snake (Notechis ater niger) venom.* Toxicon, 1989. **27**(7): p. 773-9.

739. Winchester, B.G., N.S. Van-de-Water, and R.D. Jolly, *The nature of the residual alpha-mannosidase in plasma in bovine mannosidosis.* Biochem J, 1976. **157**(1): p. 183-8.

740. Windorfer, A., Jr. and D. Karitzky, *[The importance of the albumin bilirubin binding in drug therapy in the newborn].* Monatsschr Kinderheilkd (1902), 1975. **123**(1): p. 27-30.

741. Wolff, T., *Clinical management of the patient receiving therapeutic plasma exchange (TPE).* J Clin Apher, 1985. **2**(4): p. 378-80.

742. Wood, L. and P. Jacobs, *The effect of serial therapeutic plasmapheresis on platelet count, coagulation factors, plasma immunoglobulin, and complement levels.* J Clin Apher, 1986. **3**(2): p. 124-8.

743. Wu, J.T., E. Mau, and J.A. Knight, *Interference with carcinoembryonic antigen radioimmunoassays by glycosaminoglycans, and their removal.* Clin Chem, 1983. **29**(12): p. 2049-53.

744. Yamada, K., *[Newborn infants and disseminated intravascular coagulation].* Rinsho Byori, 1985. **Spec No 63**: p. 183-9.

745. Yamazaki, Z., et al., *Pharmacodynamics of FUT-175 anticoagulant in adsorbent plasma perfusion.* ASAIO Trans, 1989. **35**(3): p. 567-9.

746. Yamazaki, Z., et al., *Extracorporeal methods of liver failure treatment.* Biomater Artif Cells Artif Organs, 1987. **15**(4): p. 667-75.

747. Yamazaki, Z., et al., *Extracorporeal methods of liver failure treatment.* Biomaterials, Artificial Cells, and Artificial Organs, 1988. **15**(4): p. 667-675.

748. Yanagishita, M. and D.J. McQuillan, *Two forms of plasma membrane-intercalated heparan sulfate proteoglycan in rat ovarian granulosa cells. Labeling of proteoglycans with a photoactivatable hydrophobic probe and effect of the membrane anchor-specific phospholipase C.* J Biol Chem, 1989. **264**(29): p. 17551-8.

749. Yin, E.T., S. Wessler, and P.J. Stoll, *Identity of plasma-activated factor X inhibitor with antithrombin 3 and heparin cofactor.* J Biol Chem, 1971. **246**(11): p. 3712-9.

750. Yokoyama, S., *Treatment of hypercholesterolemia by chemical adsorption of lipoproteins.* J Clin Apher, 1988. **4**(2-3): p. 66-71.

751. Yuasa, S., *[Complications and adverse effects of plasmapheresis and their incidences].* Nihon Rinsho, 1984. **42**(8): p. 1920-9.

752. Zarnegar, R. and G. Michalopoulos, *Purification and biological characterization of human hepatopoietin A, a polypeptide growth factor for hepatocytes.* Cancer Res, 1989. **49**(12): p. 3314-20.

753. Ziemer, S., et al., *Therapeutic plasmapheresis and haemostatic system.* Folia Haematol Int Mag Klin Morphol Blutforsch, 1988. **115**(4): p. 563-8.

754. Zwaveling, J.H., et al., *Renal failure associated with the use of dextran-40.* Neth J Med, 1989. **35**(5-6): p. 321-6.

755. Abdullah, K.M., et al., *A neutral glycoprotease of Pasteurella haemolytica A1 specifically cleaves O-sialoglycoproteins.* Infect Immun, 1992. **60**(1): p. 56-62.

756. Abe, H., et al., *Efficacy of nafamostat mesilate as a regional anticoagulant in experimental direct hemoperfusion and in plasma exchange on humans.* Artif Organs, 1992. **16**(2): p. 206-8.

757. Abouna, G.M., et al., *Comparison of the effect of plasmapheresis using human albumin or dextran 40 on the survival of pig-to-dog renal xenografts.* Transplant Proc, 1996. **28**(1): p. 212-4.

758. Absar, S., et al., *Serum albumin-protamine conjugate for biocompatible platform for targeted delivery of therapeutic macromolecules.* J Biomed Mater Res A, 2014. **102**(8): p. 2481-90.

759. Ahmad, Z., et al., *Sephadex and sephadex ion-exchange filtration improves the quality and freezability of low-grade buffalo semen ejaculates.* Theriogenology, 2003. **59**(5-6): p. 1189-202.

760. Aleksenko, S.S., et al., *Metallomics for drug development: an integrated CE-ICP-MS and ICP-MS approach reveals the speciation changes for an investigational ruthenium(III) drug bound to holo-transferrin in simulated cancer cytosol.* Metallomics, 2013. **5**(8): p. 955-63.

761. Alsaleh, K.A., et al., *Delayed-onset HIT caused by low-molecular-weight heparin manifesting during fondaparinux prophylaxis.* American Journal of Hematology, 2008. **83**(11): p. 876-878.

762. Al-Soud, W.A. and P. Rådström, *Purification and characterization of PCR-inhibitory components in blood cells.* J Clin Microbiol, 2001. **39**(2): p. 485-93.

763. Ameer, G.A., et al., *Ex vivo evaluation of a Taylor-Couette flow, immobilized heparinase I device for clinical application.* Proc Natl Acad Sci U S A, 1999. **96**(5): p. 2350-5.

764. Ameer, G.A., et al., *Investigation of a whole blood fluidized bed Taylor-Couette flow device for enzymatic heparin neutralization.* Biotechnology and Bioengineering, 1999. **62**(5): p. 602-608.

765. Ameer, G.A., et al., *Regional heparinization via simultaneous separation and reaction in a novel Taylor-Couette flow device.* Biotechnol Bioeng, 1999. **63**(5): p. 618-24.

766. Anghileri, L.J. and P. Thouvenot, *Non-transferrin-bound iron and tumor cells.* Anticancer Res, 1997. **17**(4a): p. 2529-33.

767. Angulo, Y., et al., *Isolation and characterization of a myotoxic phospholipase A2 from the venom of the arboreal snake Bothriechis (Bothrops) schlegelii from Costa Rica.* Arch Biochem Biophys, 1997. **339**(2): p. 260-6.

768. Ansari, M., et al., *Are nk cells important in the pathophysiology of thrombotic microangiopathy post hematopoietic stem cell transplantation?* Pediatric Blood and Cancer, 2010. **55**(5): p. 928-929.

769. Anthony, M.L., et al., *Studies of the biochemical toxicology of uranyl nitrate in the rat.* Arch Toxicol, 1994. **68**(1): p. 43-53.

770. Applegate, D., et al., *The alpha(E)C domain of human fibrinogen-420 is a stable and early plasmin cleavage product.* Blood, 2000. **95**(7): p. 2297-303.

771. Arakawa, M., et al., *Effects of dextran 70 on hemodynamics and lung liquid and protein exchange in awake sheep.* Circ Res, 1990. **67**(4): p. 852-61.

772. Arenas, J., et al., *Serum proteases prevent bacterial biofilm formation: role of kallikrein and plasmin.* Virulence, 2021. **12**(1): p. 2902-2917.

773. Arias-Borrego, A., T. García-Barrera, and J.L. Gómez-Ariza, *Speciation of manganese binding to biomolecules in pine nuts (Pinus pinea) by two-dimensional liquid chromatography coupled to ultraviolet and inductively coupled plasma mass spectrometry detectors followed by identification by electrospray ionization mass spectrometry.* Rapid Commun Mass Spectrom, 2008. **22**(19): p. 3053-60.

774. Assreuy, A.M., et al., *Vascular effects of a sulfated polysaccharide from the red marine alga Solieria filiformis.* Nat Prod Commun, 2010. **5**(8): p. 1267-72.

775. Babaie, M., et al., *Isolation and partial purification of anticoagulant fractions from the venom of the Iranian snake Echis carinatus.* Acta Biochim Pol, 2013. **60**(1): p. 17-20.

776. Bagnaresi, P., B. Basso, and P. Pupillo, *The NADH-dependent Fe(3+)-chelate reductases of tomato roots.* Planta, 1997. **202**(4): p. 427-34.

777. Baikar, V.M., et al., *Separation of antihemophilic factor VII from human plasma by column chromatography.* Indian J Clin Biochem, 2003. **18**(1): p. 80-6.

778. Bantan, T., R. Milacic, and B. Pihlar, *Possibilities for speciation of Al-citrate and other negatively charged Al complexes by anion-exchange FPLC-ICP-AES.* Talanta, 1998. **46**(1): p. 227-35.

779. Bapat, P., et al., *Rivaroxaban transfer across the dually perfused isolated human placental cotyledon.* Am J Obstet Gynecol, 2015. **213**(5): p. 710.e1-6.

780. Bar Barroeta, A., et al., *Nanobodies against factor XI apple 3 domain inhibit binding of factor IX and reveal a novel binding site for high molecular weight kininogen.* J Thromb Haemost, 2022. **20**(11): p. 2538-2549.

781. Ben Mansour, M., et al., *Characterization and anticoagulant activity of a fucosylated chondroitin sulfate with unusually procoagulant effect from sea cucumber.* Carbohydr Polym, 2017. **174**: p. 760-771.

782. Bergwik, J., et al., *Binding of the human antioxidation protein α(1)-microglobulin (A1M) to heparin and heparan sulfate. Mapping of binding site, molecular and functional characterization, and co-localization in vivo and in vitro.* Redox Biol, 2021. **41**: p. 101892.

783. Bert, J.L., et al., *A model of fluid and solute exchange in the human: validation and implications.* Acta Physiol Scand, 2000. **170**(3): p. 201-9.

784. Berthiaume, E.P., C. Medina, and J.A. Swanson, *Molecular size-fractionation during endocytosis in macrophages.* J Cell Biol, 1995. **129**(4): p. 989-98.

785. Bian, C., Z. Wang, and J. Shi, *Extraction Optimization, Structural Characterization, and Anticoagulant Activity of Acidic Polysaccharides from Auricularia auricula-judae.* Molecules, 2020. **25**(3).

786. Bijl, E., et al., *Protein, casein, and micellar salts in milk: current content and historical perspectives.* J Dairy Sci, 2013. **96**(9): p. 5455-64.

787. Bird, C.H., et al., *Cationic sites on granzyme B contribute to cytotoxicity by promoting its uptake into target cells.* Mol Cell Biol, 2005. **25**(17): p. 7854-67.

788. Blumberg, N., et al., *Sickle red blood cells are more susceptible to in vitro haemolysis when exposed to normal saline versus Plasma-Lyte A.* Vox Sang, 2019. **114**(4): p. 325-329.

789. Bode, D.C., et al., *Serum Albumin's Protective Inhibition of Amyloid-β Fiber Formation Is Suppressed by Cholesterol, Fatty Acids and Warfarin.* J Mol Biol, 2018. **430**(7): p. 919-934.

790. Boga, C., et al., *Alterations of circulating endothelial cells after apheresis in patients with sickle cell disease: a potential clue for restoration of pathophysiology.* Transfus Apher Sci, 2010. **43**(3): p. 273-279.

791. Bosch, T., et al., *Lipid apheresis by hemoperfusion: in vitro efficacy and ex vivo biocompatibility of a new low-density lipoprotein adsorber compatible with human whole blood.* Artif Organs, 1993. **17**(7): p. 640-52.

792. Brauer, K.I., et al., *Hypoproteinemia does not alter plasma volume expansion in response to a 0.9% saline bolus in awake sheep.* Critical Care Medicine, 2010. **38**(10): p. 2011-2015.

793. Brenner, P., et al., *The influence of antibody and complement removal with a Ig-Therasorb column in a xenogeneic working heart model.* Eur J Cardiothorac Surg, 1999. **15**(5): p. 672-9.

794. Brown, A.C., H.S. Macrae, and N.S. Turner, *Tricarboxylic-acid-cycle intermediates and cycle endurance capacity.* Int J Sport Nutr Exerc Metab, 2004. **14**(6): p. 720-9.

795. Brownie, C.F., et al., *Teratogenic effect of calcium edetate (CaEDTA) in rats and the protective effect of zinc.* Toxicol Appl Pharmacol, 1986. **82**(3): p. 426-43.

796. Buch, F., et al., *Secreted pitfall-trap fluid of carnivorous Nepenthes plants is unsuitable for microbial growth.* Ann Bot, 2013. **111**(3): p. 375-83.

797. Busund, R., et al., *Repeated plasma therapy induces fatal shock in experimental septicemia.* Circulatory Shock, 1993. **40**(4): p. 268-275.

798. Butty, E.M., et al., *Outcomes of nonsteroidal anti-inflammatory drug toxicosis treated with therapeutic plasma exchange in 62 dogs.* Journal of Veterinary Internal Medicine, 2022. **36**(5): p. 1641-1647.

799. Campbell, N.F., et al., *Development of a chromatographic method for the isolation and detection of hygromycin B in biological fluids.* J Chromatogr B Biomed Sci Appl, 1997. **692**(2): p. 367-74.

800. Cao, N.N., et al., *In vitro study of a novel low-density lipoprotein adsorbent.* Artif Cells Blood Substit Immobil Biotechnol, 2002. **30**(1): p. 53-61.

801. Cao, S., et al., *Anticoagulant and Antithrombotic Properties in Vitro and in Vivo of a Novel Sulfated Polysaccharide from Marine Green Alga Monostroma nitidum.* Mar Drugs, 2019. **17**(4).

802. Cappello, M., et al., *Ancylostoma caninum anticoagulant peptide: a hookworm-derived inhibitor of human coagulation factor Xa.* Proc Natl Acad Sci U S A, 1995. **92**(13): p. 6152-6.

803. Cardigan, R., et al., *In vitro function of buffy coat-derived platelet concentrates stored for 9 days in CompoSol, PASII or 100% plasma in three different storage bags.* Vox Sang, 2008. **94**(2): p. 103-12.

804. Carmen, R., *The selection of plastic materials for blood bags.* Transfus Med Rev, 1993. **7**(1): p. 1-10.

805. Caron, A., et al., *Measurement of blood volume after haemodilution with haemoglobin-based oxygen carriers by a radiolabelled-albumin method.* Transfus Med, 2001. **11**(6): p. 433-42.

806. Carrette, O., et al., *Purification and characterization of pig inter-alpha-inhibitor and its constitutive heavy chains.* Biochim Biophys Acta, 1997. **1338**(1): p. 21-30.

807. Carter, J.H., et al., *Euvolemic automated transfusion of red cells in volume-sensitive patients.* Transfusion, 2014. **54**(SUPPL. 2): p. 130A.

808. Carter, T.H., B.A. Everson, and O.D. Ratnoff, *Cabbage seed protease inhibitor: a slow, tight-binding inhibitor of trypsin with activity toward thrombin, activated Stuart factor (factor Xa), activated Hageman factor (factor XIIa), and plasmin.* Blood, 1990. **75**(1): p. 108-15.

809. Castellino, F.J., V.A. Ploplis, and L. Zhang, *gamma-Glutamate and beta-hydroxyaspartate in proteins.* Methods Mol Biol, 2008. **446**: p. 85-94.

810. Cavagnetto, C., et al., *Residual red cells in blood components: A multisite study of fully automated enumeration using a hematology analyzer.* Transfusion, 2021. **61**(2): p. 568-578.

811. Chaurra, A., et al., *Lucifer Yellow as a live cell fluorescent probe for imaging water transport in subcellular organelles.* Appl Spectrosc, 2011. **65**(1): p. 20-5.

812. Chaves-Filho, A.B., et al., *Futile cycle of β-oxidation and de novo lipogenesis are associated with essential fatty acids depletion in lipoatrophy.* Biochim Biophys Acta Mol Cell Biol Lipids, 2023. **1868**(3): p. 159264.

813. Chen, F., et al., *Complement proteins bind to nanoparticle protein corona and undergo dynamic exchange in vivo.* Nat Nanotechnol, 2017. **12**(4): p. 387-393.

814. Chen, J., et al., *Autocrine action and its underlying mechanism of nitric oxide on intracellular Ca2+ homeostasis in vascular endothelial cells.* J Biol Chem, 2000. **275**(37): p. 28739-49.

815. Chen, R.Y., et al., *Effects of dextran-induced hyperviscosity on regional blood flow and hemodynamics in dogs.* Am J Physiol, 1989. **256**(3 Pt 2): p. H898-905.

816. Chen, Z., et al., *Speciation of Zn-aminopolycarboxylic complexes by electrospray ionization mass spectrometry and ion chromatography with inductively coupled plasma mass spectrometry.* Rapid Commun Mass Spectrom, 2009. **23**(3): p. 419-24.

817. Chen, Z., et al., *Speciation of metal-EDTA complexes by flow injection analysis with electrospray ionization mass spectrometry and ion chromatography with inductively coupled plasma mass spectrometry.* J Sep Sci, 2008. **31**(21): p. 3796-802.

818. Chien, D., et al., *Malonyl-CoA content and fatty acid oxidation in rat muscle and liver in vivo.* Am J Physiol Endocrinol Metab, 2000. **279**(2): p. E259-65.

819. Chuang, C.K., et al., *Effects of anticoagulants in amino acid analysis: comparisons of heparin, EDTA, and sodium citrate in vacutainer tubes for plasma preparation.* Clin Chem, 1998. **44**(5): p. 1052-6.

820. Clifton, J., et al., *Use of proteomics for validation of the isolation process of clotting factor IX from human plasma.* J Proteomics, 2010. **73**(3): p. 678-88.

821. Cohen, I., et al., *A novel homozygous SLC25A1 mutation with impaired mitochondrial complex V: Possible phenotypic expansion.* Am J Med Genet A, 2018. **176**(2): p. 330-336.

822. Colahan, P.T., et al., *The effect of sildenafil citrate administration on selected physiological parameters of exercising Thoroughbred horses.* Equine Vet J Suppl, 2010(38): p. 606-12.

823. Conover, C.D., et al., *The effects of hemodilution with polyethylene glycol bovine hemoglobin (PEG-Hb) in a conscious porcine model.* J Investig Med, 1996. **44**(5): p. 238-46.

824. Cousineau, D., C.P. Rose, and C.A. Goresky, *Plasma expansion effect on cardiac capillary and adrenergic exchange in intact dogs.* J Appl Physiol (1985), 1986. **60**(1): p. 147-53.

825. Covell, D.G., P.H. Abbrecht, and M. Berman, *The effect of hepatic uptake on the disappearance of warfarin from the plasma of rats: a kinetic analysis.* J Pharmacokinet Biopharm, 1983. **11**(2): p. 127-45.

826. Crawmer, B.P., J.A. Cook, and R.R. Brown, *Determination of amifloxacin and two of its principal metabolites in plasma and urine by high-performance liquid chromatography using automated column switching.* J Chromatogr, 1990. **530**(2): p. 407-17.

827. Crissinger, K.D. and P. Tso, *The role of lipids in ischemia/reperfusion-induced changes in mucosal permeability in developing piglets.* Gastroenterology, 1992. **102**(5): p. 1693-9.

828. Cucnik, S., et al., *Concomitant isolation of protein C inhibitor and unnicked beta2-glycoprotein I.* Clin Chem Lab Med, 2004. **42**(2): p. 171-4.

829. Cui, G., et al., *Diagnosis of LVAD Thrombus using a High-Avidity Fibrin-Specific (99m)Tc Probe.* Theranostics, 2018. **8**(4): p. 1168-1179.

830. Culler, C.A., et al., *Centrifugal therapeutic plasma exchange in dogs with immune-mediated hemolytic anemia (2016-2018): 7 cases.* J Vet Emerg Crit Care (San Antonio), 2022. **32**(5): p. 645-652.

831. de Sousa, B.B., et al., *A New Platelet-Aggregation-Inhibiting Factor Isolated from Bothrops moojeni Snake Venom.* Biomed Res Int, 2017. **2017**: p. 4315832.

832. de Wit, C., et al., *Elevation of plasma viscosity induces sustained NO-mediated dilation in the hamster cremaster microcirculation in vivo.* Pflugers Arch, 1997. **434**(4): p. 354-61.

833. Desquesnes, M. and L. Tresse, *[Evaluation of sensitivity of PCR for detecting DNA of Trypanosoma vivax with several methods of blood sample preparations].* Rev Elev Med Vet Pays Trop, 1996. **49**(4): p. 322-7.

834. Diaz-Collier, J.A., et al., *Refold and characterization of recombinant tissue factor pathway inhibitor expressed in Escherichia coli.* Thromb Haemost, 1994. **71**(3): p. 339-46.

835. Dietrich, G.V., *[Autologous phasmapheresis--from euphoria to rationality: practical handling of scientific knowledge. Part 3. Autologous plasmapheresis: valuable coagulation treatment or expansive volume replacement? Physiology and pharmacology].* Anasthesiol Intensivmed Notfallmed Schmerzther, 2000. **35**(12): p. 763-5.

836. Dihazi, H., et al., *Protein adsorption during LDL-apheresis: Proteomic analysis.* Nephrology Dialysis Transplantation, 2008. **23**(9): p. 2925-2935.

837. Ding, Z. and Y. Xu, *A rapid simplified purification of bovine thrombin.* Prep Biochem, 1995. **25**(1-2): p. 21-8.

838. Döring, O., S. Lüthje, and M. Böttger, *Inhibitors of the plasma membrane redox system of Zea mays L. roots. The vitamin K antagonists dicumarol and warfarin.* Biochim Biophys Acta, 1992. **1110**(2): p. 235-8.

839. Dudchenko, N.O., O.M. Mykhaĭlik, and I.P. Lubianova, *[Effect of alpha-lipoic acid preparations on bilirubin and transferrin levels and ferritin iron and transferrin iron content].* Ukr Biokhim Zh (1999), 2001. **73**(2): p. 102-5.

840. Edwards, I.J. and W.D. Wagner, *Cell surface heparan sulfate proteoglycan and chondroitin sulfate proteoglycan of arterial smooth muscle cells.* Am J Pathol, 1992. **140**(1): p. 193-205.

841. Eerkes, A., T. Addison, and W. Naidong, *Simultaneous assay of sildenafil and desmethylsildenafil in human plasma using liquid chromatography-tandem mass spectrometry on silica column with aqueous-organic mobile phase.* J Chromatogr B Analyt Technol Biomed Life Sci, 2002. **768**(2): p. 277-84.

842. Eguchi, S., et al., *Effects of anticoagulants on porcine hepatocytes in vitro: Implications in the porcine hepatocyte-based bioartificial liver.* International Journal of Artificial Organs, 1999. **22**(5): p. 329-333.

843. Ehmke, H., et al., *Modulation of erythropoietin formation by changes in blood volume in conscious dogs.* J Physiol, 1995. **488 ( Pt 1)**(Pt 1): p. 181-91.

844. Electricwala, A., et al., *Isolation of thrombin inhibitor from the leech Hirudinaria manillensis.* Blood Coagul Fibrinolysis, 1991. **2**(1): p. 83-9.

845. Enkhbaatar, P., et al., *Combined anticoagulants ameliorate acute lung injury in sheep after burn and smoke inhalation.* Clin Sci (Lond), 2008. **114**(4): p. 321-9.

846. Eriksson, L., et al., *Platelet concentrates in an additive solution prepared from pooled buffy coats. In vivo studies.* Vox Sang, 1993. **64**(3): p. 133-8.

847. Ernst, S., et al., *Expression in Escherichia coli, purification and characterization of heparinase I from Flavobacterium heparinum.* Biochem J, 1996. **315 ( Pt 2)**(Pt 2): p. 589-97.

848. Evrard, J., et al., *Are the DOAC plasma level thresholds appropriate for clinical decision-making? A reappraisal using thrombin generation testing.* Int J Lab Hematol, 2021. **43**(1): p. e48-e51.

849. Eya, K., et al., *Development of a membrane oxygenator for long-term respiratory support and its experimental evaluation in prolonged ECMO.* Asaio j, 1996. **42**(5): p. M832-6.

850. Faivre, B., et al., *Methemoglobin formation after administration of hemoglobin conjugated to carboxylate dextran in guinea pigs. Attempts to prevent the oxidation of hemoglobin.* Artif Cells Blood Substit Immobil Biotechnol, 1994. **22**(3): p. 551-8.

851. Fallon, B.P., et al., *A pumpless artificial lung without systemic anticoagulation: The Nitric Oxide Surface Anticoagulation system.* J Pediatr Surg, 2022. **57**(1): p. 26-33.

852. Farrish, H.H., et al., *Validation of a liquid chromatography post-column derivatization assay for the determination of cisplatin in plasma.* J Pharm Biomed Anal, 1994. **12**(2): p. 265-71.

853. Fernández, R.G. and J.I. García Alonso, *Separation of rare earth elements by anion-exchange chromatography using ethylenediaminetetraacetic acid as mobile phase.* J Chromatogr A, 2008. **1180**(1-2): p. 59-65.

854. Finotti, P., P. Carraro, and A. Calderan, *Purification of proteinase-like and Na+/K(+)-ATPase stimulating substance from plasma of insulin-dependent diabetics and its identification as alpha 1-antitrypsin.* Biochim Biophys Acta, 1992. **1139**(1-2): p. 122-32.

855. Fischel, R.J., et al., *Safe and effective plasma exchange to remove antibodies prior to xenogeneic heart transplantation in small primates.* ASAIO Trans, 1991. **37**(3): p. M498-500.

856. Fischer, B.E., et al., *Effect of multimerization of human and recombinant von Willebrand factor on platelet aggregation, binding to collagen and binding of coagulation factor VIII.* Thromb Res, 1996. **84**(1): p. 55-66.

857. Fischer, B.E., et al., *Biochemical and functional characterization of recombinant von Willebrand factor produced on a large scale.* Cell Mol Life Sci, 1997. **53**(11-12): p. 943-50.

858. Fischer, B.E., et al., *Selectivity of von Willebrand factor triplet bands towards heparin binding supports structural model.* Eur J Haematol, 1999. **62**(3): p. 169-73.

859. Follea, G., et al., *Developing collaboration between blood establishments, suppliers and regulators: The "managed convergence".* Vox Sanguinis, 2011. **101**(SUPPL. 1): p. 92.

860. Francey, T. and A. Schweighauser, *Membrane-based therapeutic plasma exchange in dogs: Prescription, anticoagulation, and metabolic response.* J Vet Intern Med, 2019. **33**(4): p. 1635-1645.

861. Frickenstein, A.N., et al., *Quantification of monodisperse and biocompatible gold nanoparticles by single-particle ICP-MS.* Anal Bioanal Chem, 2023.

862. Fujikawa, K., et al., *Purification of human von Willebrand factor-cleaving protease and its identification as a new member of the metalloproteinase family.* Blood, 2001. **98**(6): p. 1662-6.

863. Gao, Y., et al., *Porcine acute liver failure model established by two-phase surgery and treated with hollow fiber bioartificial liver support system.* World J Gastroenterol, 2005. **11**(35): p. 5468-74.

864. Gawlowski, D.M., et al., *Atriopeptin does not augment the transvascular flux of macromolecules in the hamster cheek pouch.* Proc Soc Exp Biol Med, 1990. **194**(2): p. 131-5.

865. Ge, Z. and C. Wei, *Simultaneous analysis of SbIII, SbV and TMSb by high performance liquid chromatography-inductively coupled plasma-mass spectrometry detection: application to antimony speciation in soil samples.* J Chromatogr Sci, 2013. **51**(5): p. 391-9.

866. Gervasi, G.B., et al., *Disposition of a new heparan sulfate with fibrinolytic activity in the rat.* Arzneimittelforschung, 1993. **43**(4): p. 445-9.

867. Geyer, R.P., *"Bloodless" rats through the use of artificial blood substitutes.* Fed Proc, 1975. **34**(6): p. 1499-1505.

868. Gioglio, L., et al., *Localization of Ca-ATPase in frog crista ampullaris.* Neuroreport, 1998. **9**(7): p. 1309-12.

869. Gokhale, A., et al., *Clotting during autologous hematopoietic progenitor cells collection.* Journal of Clinical Apheresis, 2016. **31**(2): p. 92-93.

870. González, J.C., C. López, and J.U. Carmona, *Implications of anticoagulants and gender on cell counts and growth factor concentration in platelet-rich plasma and platelet-rich gel supernatants from rabbits.* Vet Comp Orthop Traumatol, 2016. **29**(2): p. 115-24.

871. Gosselin, A.R., et al., *Hyperfibrinolysis drives mechanical instabilities in a simulated model of trauma induced coagulopathy.* Thrombosis Research, 2022. **220**: p. 131-140.

872. Griffith, C.A., et al., *Development of a method to measure plasma and whole blood choline by liquid chromatography tandem mass spectrometry.* Ann Clin Biochem, 2010. **47**(Pt 1): p. 56-61.

873. Grillberger, R., et al., *Temperature-dependent irreversible conformational change of adamts13 upon metal ion chelation.* Blood, 2015. **126**(23): p. 2238.

874. Grinnell, B.W., et al., *Gamma-carboxylated isoforms of recombinant human protein S with different biologic properties.* Blood, 1990. **76**(12): p. 2546-54.

875. Groover, J., et al., *Extracorporeal blood purification in acutely intoxicated veterinary patients: A multicenter retrospective study (2011-2018): 54 cases.* J Vet Emerg Crit Care (San Antonio), 2022. **32**(1): p. 34-41.

876. Gruber, A. and J.H. Griffin, *Direct detection of activated protein C in blood from human subjects.* Blood, 1992. **79**(9): p. 2340-8.

877. Grützmacher, P., et al., *In vivo rheologic effects of lipid apheresis techniques: comparison of dextran sulfate LDL adsorption and heparin induced LDL precipitation.* ASAIO Trans, 1990. **36**(3): p. M327-30.

878. Gulliksson, H., *Platelet storage media.* Transfus Apher Sci, 2001. **24**(3): p. 241-4.

879. Guo, Z.X., Q. Cai, and Z. Yang, *Determination of glyphosate and phosphate in water by ion chromatography--inductively coupled plasma mass spectrometry detection.* J Chromatogr A, 2005. **1100**(2): p. 160-7.

880. Guo, Z.X., Q. Cai, and Z. Yang, *Ion chromatography/inductively coupled plasma mass spectrometry for simultaneous determination of glyphosate, glufosinate, fosamine and ethephon at nanogram levels in water.* Rapid Commun Mass Spectrom, 2007. **21**(10): p. 1606-12.

881. Hafeez, A., et al., *Bilirubin Interference in Plasma Amino Acid Analysis by Ion Exchange Chromatography.* J Coll Physicians Surg Pak, 2018. **28**(9): p. 667-671.

882. Hagisawa, K., et al., *H12-(ADP)-liposomes for hemorrhagic shock in thrombocytopenia: Mesenteric artery injury model in rabbits.* Research and Practice in Thrombosis and Haemostasis, 2022. **6**(2): p. e12659.

883. Halldórsdóttir, A.M., L. Zhang, and D.M. Tollefsen, *N-Acetylgalactosamine 4,6-O-sulfate residues mediate binding and activation of heparin cofactor II by porcine mucosal dermatan sulfate.* Glycobiology, 2006. **16**(8): p. 693-701.

884. Haltern, C., et al., *Impact of inverse plasma filtration on complement activation in porcine blood.* Artif Organs, 2005. **29**(4): p. 306-12.

885. Handley Jr, H.H., et al., *Slow continuous intracorporeal plasmapheresis for acute fluid overload.* Blood Purification, 2003. **21**(1): p. 72-78.

886. Hardersen, R., et al., *Comparison of cytokine changes in three different lipoprotein apheresis systems in an ex vivo whole blood model.* J Clin Apher, 2020. **35**(2): p. 104-116.

887. Hardersen, R.I., et al., *Role of complement factor c5 in leukocyte expression of cd11b and formation of leukocyte-platelet conjugates in an ex vivo model of plasma separation.* Journal of Clinical Apheresis, 2013. **28**(2): p. 109-110.

888. Hayakawa, Y., et al., *Selective activation of heparin cofactor II by a sulfated polysaccharide isolated from the leaves of Artemisia princeps.* Blood Coagul Fibrinolysis, 1995. **6**(7): p. 643-9.

889. Hendriks, D., et al., *Purification and characterization of a new arginine carboxypeptidase in human serum.* Biochim Biophys Acta, 1990. **1034**(1): p. 86-92.

890. Hessing, M., J. Paardekooper, and C.E. Hack, *Separation of different forms of the fourth component of human complement by fast protein liquid chromatography.* J Immunol Methods, 1993. **157**(1-2): p. 39-48.

891. Hill, W.G., et al., *Organ-specific over-sulfation of glycosaminoglycans and altered extracellular matrix in a mouse model of cystic fibrosis.* Biochem Mol Med, 1997. **62**(1): p. 113-22.

892. Hill, W.G., et al., *Sulfation of chondroitin/dermatan sulfate by cystic fibrosis pancreatic duct cells is not different from control cells.* Biochem Mol Med, 1997. **62**(1): p. 85-94.

893. Hirose, M., S. Kameyama, and H. Ohi, *Characterization of N-linked oligosaccharides attached to recombinant human antithrombin expressed in the yeast Pichia pastoris.* Yeast, 2002. **19**(14): p. 1191-202.

894. Høgåsen, K., T.E. Mollnes, and M. Harboe, *Heparin-binding properties of vitronectin are linked to complex formation as illustrated by in vitro polymerization and binding to the terminal complement complex.* J Biol Chem, 1992. **267**(32): p. 23076-82.

895. Hohtatsu, K., et al., *Effect of Various Anticoagulant Agents on Large-Volume Leukocytapheresis Using New Cellsorba CS-180S Filter.* Therapeutic Apheresis and Dialysis, 2011. **15**(4): p. 355-359.

896. Holbeck, S. and P.O. Grände, *Effects on capillary fluid permeability and fluid exchange of albumin, dextran, gelatin, and hydroxyethyl starch in cat skeletal muscle.* Crit Care Med, 2000. **28**(4): p. 1089-95.

897. Holden, A.J., D. Littlejohn, and G.S. Fell, *Determination of citrate in plasma protein solutions by UV-visible spectrophotometry and ion chromatography.* J Pharm Biomed Anal, 1996. **14**(6): p. 713-9.

898. Hollands, A., et al., *A bacterial pathogen co-opts host plasmin to resist killing by cathelicidin antimicrobial peptides.* J Biol Chem, 2012. **287**(49): p. 40891-7.

899. Horn, F., P.C. dos Santos, and C. Termignoni, *Boophilus microplus anticoagulant protein: an antithrombin inhibitor isolated from the cattle tick saliva.* Arch Biochem Biophys, 2000. **384**(1): p. 68-73.

900. Huang, Z., et al., *A novel solid-phase site-specific PEGylation enhances the in vitro and in vivo biostabilty of recombinant human keratinocyte growth factor 1.* PLoS One, 2012. **7**(5): p. e36423.

901. Huish, S., et al., *Effect of storage of plasma in the presence of red blood cells and platelets: re-evaluating the shelf life of whole blood.* Transfusion, 2019. **59**(11): p. 3468-3477.

902. Iarochkin, V.S., *[Changes in the hemodynamics and respiration of dogs after the transfusion of massive doses of freshly prepared plasma containing platelets or platelet-free].* Gematol Transfuziol, 1983. **28**(4): p. 32-8.

903. Ichinose, F., et al., *Nebulized sildenafil is a selective pulmonary vasodilator in lambs with acute pulmonary hypertension.* Crit Care Med, 2001. **29**(5): p. 1000-5.

904. Ierino, F.L., et al., *Disseminated intravascular coagulation in association with the delayed rejection of pig-to-baboon renal xenografts.* Transplantation, 1998. **66**(11): p. 1439-50.

905. Iida, J., et al., *Coordinate role for cell surface chondroitin sulfate proteoglycan and alpha 4 beta 1 integrin in mediating melanoma cell adhesion to fibronectin.* J Cell Biol, 1992. **118**(2): p. 431-44.

906. Illingworth, D.R. and O.W. Portman, *Exchange of phospholipids between low and high density lipoproteins of squirrel monkeys.* J Lipid Res, 1972. **13**(2): p. 220-7.

907. Iversen, V.V., et al., *Continuous measurements of plasma protein extravasation with microdialysis after various inflammatory challenges in rat and mouse skin.* Am J Physiol Heart Circ Physiol, 2004. **286**(1): p. H108-12.

908. Jagger, J.E., et al., *Role of erythrocyte in regulating local O2 delivery mediated by hemoglobin oxygenation.* Am J Physiol Heart Circ Physiol, 2001. **280**(6): p. H2833-9.

909. James, H.L., et al., *Isolation and characterization of mouse coagulation factor X -- biophysical and enzymological properties.* Thromb Haemost, 1997. **78**(3): p. 1049-54.

910. Janssen, M., J. Meier, and T.A. Freyvogel, *Purification and characterization of an antithrombin III inactivating enzyme from the venom of the African night adder (Causus rhombeatus).* Toxicon, 1992. **30**(9): p. 985-99.

911. Jiang, J.S., et al., *Effects of activated protein C on ventilator-induced lung injury in rats.* Respiration, 2010. **80**(3): p. 246-53.

912. John, H., et al., *Small-scale purification of butyrylcholinesterase from human plasma and implementation of a μLC-UV/ESI MS/MS method to detect its organophosphorus adducts.* Drug Test Anal, 2015. **7**(10): p. 947-56.

913. Johnson, R.F., et al., *Bupivacaine transfer across the human term placenta. A study using the dual perfused human placental model.* Anesthesiology, 1995. **82**(2): p. 459-68.

914. Jones, C., et al., *Donor plasma Ismore effective than 5% albumin for the treatment of heparin induced thrombocytopenia in an in vitromodel of TPE.* Journal of Clinical Apheresis, 2018. **33**(2): p. 133-134.

915. Jupin, M., et al., *NMR identification of endogenous metabolites interacting with fatted and non-fatted human serum albumin in blood plasma: Fatty acids influence the HSA-metabolite interaction.* J Magn Reson, 2013. **228**: p. 81-94.

916. Kabcenell, A.K., et al., *Binding and hydrolysis of guanine nucleotides by Sec4p, a yeast protein involved in the regulation of vesicular traffic.* J Biol Chem, 1990. **265**(16): p. 9366-72.

917. Kadar, J.G. and H. Borberg, *Biocompatibility of extracorporeal immunoadsorption systems.* Transfus Sci, 1990. **11**(2): p. 223-39.

918. Kano, F., et al., *A resealed-cell system for analyzing pathogenic intracellular events: perturbation of endocytic pathways under diabetic conditions.* PLoS One, 2012. **7**(8): p. e44127.

919. Karabel Ocal, S., et al., *Plasmonic assemblies of gold nanorods on nanoscale patterns of poly(ethylene glycol): Application in surface-enhanced Raman spectroscopy.* J Colloid Interface Sci, 2018. **532**: p. 449-455.

920. Karlsen, T.V., et al., *Neurogenic inflammation in mice deficient in heparin-synthesizing enzyme.* American Journal of Physiology - Heart and Circulatory Physiology, 2004. **286**(3 55-3): p. H884-H888.

921. Karmakar, S., et al., *Isolation of a haemorrhagic protein toxin (SA-HT) from the Indian venomous butterfish (Scatophagus argus, Linn) sting extract.* Indian J Exp Biol, 2004. **42**(5): p. 452-60.

922. Karpiel, A., et al., *Paired 42-day in vitro comparison of red cell concentrates produced from whole blood by spinning membrane technology or centrifugation.* Transfusion, 2013. **53**(SUPPL. 2): p. 81A.

923. Kawamura, A., et al., *Reduction of EDA (+) fibronectin and its clinical importance on cryofiltration.* International Journal of Artificial Organs, 1994. **17**(10): p. 559-564.

924. Kawauchi, M., et al., *Cardiac xenotransplantation from pig to Japanese monkey with splenectomy, tacrolims, filtration plasmapheresis, and nafamstat mesilate.* Transplant Proc, 1994. **26**(3): p. 1076-7.

925. Kicera-Temple, K., et al., *Treatment of a massive naproxen overdose with therapeutic plasma exchange in a dog.* Clinical Case Reports, 2019. **7**(8): p. 1529-1533.

926. Kim, D.K., et al., *Scalable Production of a Multifunctional Protein (TSG-6) That Aggregates with Itself and the CHO Cells That Synthesize It.* PLoS One, 2016. **11**(1): p. e0147553.

927. Kjaergaard, A.B., J.L. Davis, and M.J. Acierno, *Treatment of carprofen overdose with therapeutic plasma exchange in a dog.* Journal of Veterinary Emergency and Critical Care, 2018. **28**(4): p. 356-360.

928. Klainbart, S., et al., *Therapeutic plasma exchange for the management of a type III hypersensitivity reaction and suspected immune-mediated vasculitis assumed to be caused by human albumin administration in a dog.* Journal of Veterinary Emergency and Critical Care, 2022. **32**(4): p. 532-538.

929. Kleinert, P., et al., *Mass spectrometric analysis of human transferrin in different body fluids.* Clin Chem Lab Med, 2003. **41**(12): p. 1580-8.

930. Kleinova, M., et al., *Exact molecular mass determination of various forms of native and de-N-glycosylated human plasma-derived antithrombin by means of electrospray ionization ion trap mass spectrometry.* J Mass Spectrom, 2004. **39**(12): p. 1429-36.

931. Knauf, F., et al., *The life-extending gene Indy encodes an exchanger for Krebs-cycle intermediates.* Biochem J, 2006. **397**(1): p. 25-9.

932. Knöll, J. and A. Seubert, *Indirect ultra trace determination of aminopolycarboxylic acids in surface water using ion exchange chromatography coupled on-line to inductively coupled plasma mass spectrometry.* J Chromatogr A, 2012. **1270**: p. 219-24.

933. Kobayashi, J., et al., *Analysis of protein structure-function in vivo. Adenovirus-mediated transfer of lipase lid mutants in hepatic lipase-deficient mice.* J Biol Chem, 1996. **271**(42): p. 26296-301.

934. Kong, Y., et al., *An automatic system for multidimensional integrated protein chromatography.* J Chromatogr A, 2010. **1217**(44): p. 6898-904.

935. Kopitz, J., et al., *Partial characterization and enrichment of a membrane-bound sialidase specific for gangliosides from human brain tissue.* Eur J Biochem, 1997. **248**(2): p. 527-34.

936. Korniushina, E., et al., *Use of membrane plasmapheresis and intravenous immunoglobulin in antiphospholipid antibodies positive women undergoing in vitro fertilization.* Thrombosis Research, 2014. **133**(SUPPL. 3): p. S108.

937. Kostelijk, E.H., et al., *Comparison between a new PVC platelet storage container (UPX80) and a polyolefin container.* Transfus Med, 2000. **10**(2): p. 131-9.

938. Koster, A., et al., *An assessment of different filter systems for extracorporeal elimination of bivalirudin: an in vitro study.* Anesth Analg, 2003. **96**(5): p. 1316-1319.

939. Kovačič, J., B. Božič, and S. Svetina, *Budding of giant unilamellar vesicles induced by an amphitropic protein β2-glycoprotein I.* Biophys Chem, 2010. **152**(1-3): p. 46-54.

940. Kralj, B., et al., *Speciation of aluminium in tea infusions by use of SEC and FPLC with ICP-OES and ES-MS-MS detection.* Anal Bioanal Chem, 2005. **383**(3): p. 467-75.

941. Kruk, B., et al., *Enhanced glucose availability for working muscles reduces exercise hyperthermia in dogs.* Eur J Appl Physiol Occup Physiol, 1987. **56**(5): p. 577-82.

942. Kubota, K., et al., *Platelet-derived growth factor BB secreted from osteoclasts acts as an osteoblastogenesis inhibitory factor.* J Bone Miner Res, 2002. **17**(2): p. 257-65.

943. Kucera, J., *Fungal mycelium--the source of chitosan for chromatography.* J Chromatogr B Analyt Technol Biomed Life Sci, 2004. **808**(1): p. 69-73.

944. Kumar, R.V., et al., *Malabarase, a serine protease with anticoagulant activity from Trimeresurus malabaricus venom.* Comp Biochem Physiol B Biochem Mol Biol, 2013. **164**(2): p. 111-6.

945. Kumar, S., et al., *Heparin binding carboxypeptidase E protein exhibits antibacterial activity in human semen.* Int J Biol Macromol, 2014. **64**: p. 319-27.

946. Kuo, C.Y. and S.J. Jiang, *Determination of selenium and tellurium compounds in biological samples by ion chromatography dynamic reaction cell inductively coupled plasma mass spectrometry.* J Chromatogr A, 2008. **1181**(1-2): p. 60-6.

947. Lagerberg, J.W., et al., *In vitro evaluation of the quality of blood products collected and stored in systems completely free of di(2-ethylhexyl)phthalate-plasticized materials.* Transfusion, 2015. **55**(3): p. 522-31.

948. Lagrange, F., et al., *Absence of placental transfer of pentasaccharide (Fondaparinux, Arixtra) in the dually perfused human cotyledon in vitro.* Thromb Haemost, 2002. **87**(5): p. 831-5.

949. Lay, A.J., et al., *Mice with a severe deficiency in protein C display prothrombotic and proinflammatory phenotypes and compromised maternal reproductive capabilities.* J Clin Invest, 2005. **115**(6): p. 1552-61.

950. Layden, J.D., D. Malkova, and M.A. Nimmo, *During exercise in the cold increased availability of plasma nonesterified fatty acids does not affect the pattern of substrate oxidation.* Metabolism, 2004. **53**(2): p. 203-8.

951. Lee, S.Y., B.L. Lee, and K. Söderhäll, *Processing of an antibacterial peptide from hemocyanin of the freshwater crayfish Pacifastacus leniusculus.* J Biol Chem, 2003. **278**(10): p. 7927-33.

952. Lee, T.K., W.N. Drohan, and H. Lubon, *Proteolytic processing of human protein C in swine mammary gland.* J Biochem, 1995. **118**(1): p. 81-7.

953. Lee, Y., et al., *A Therapeutic Extracorporeal Device for Specific Removal of Pathologic Asymmetric Dimethylarginine from the Blood.* Blood Purification, 2022. **51**(11): p. 889-898.

954. Lefebvre, P., et al., *In vitro production of megakaryocytes from PIXY321 versus GM-CSF-mobilized peripheral blood progenitor cells.* Stem Cells, 1997. **15**(2): p. 112-8.

955. Leibl, H., et al., *Method for the isolation of biologically active monomeric immunoglobulin A from a plasma fraction.* J Chromatogr B Biomed Appl, 1996. **678**(2): p. 173-80.

956. Leitinger, N., et al., *Decreased susceptibility of low-density lipoproteins to in-vitro oxidation after dextran-sulfate LDL-apheresis treatment.* Atherosclerosis, 1996. **126**(2): p. 305-12.

957. Li, D., et al., *Hollow-core magnetic colloidal nanocrystal clusters with ligand-exchanged surface modification as delivery vehicles for targeted and stimuli-responsive drug release.* Chemistry, 2012. **18**(51): p. 16517-24.

958. Li, F., W. Goessler, and K.J. Irgolic, *Determination of selenium compounds by HPLC with ICP-MS or FAAS as selenium-specific detector.* Se Pu, 1999. **17**(3): p. 240-4.

959. Li, J., et al., *Recyclable heparin and chitosan conjugated magnetic nanocomposites for selective removal of low-density lipoprotein from plasma.* J Mater Sci Mater Med, 2014. **25**(4): p. 1055-64.

960. Li, Q.G., et al., *A heparin-binding erythroid cell stimulating factor from fetal bovine serum has the N-terminal sequence of insulin-like growth factor II.* Biochem Biophys Res Commun, 1990. **166**(2): p. 557-61.

961. Li, X.S., S. Glasauer, and X.C. Le, *Speciation of vanadium in oilsand coke and bacterial culture by high performance liquid chromatography inductively coupled plasma mass spectrometry.* Anal Chim Acta, 2007. **602**(1): p. 17-22.

962. Liesche-Starnecker, F., et al., *Hemorrhagic lesion with detection of infected endothelial cells in human bornavirus encephalitis.* Acta Neuropathologica, 2022. **144**(2): p. 377-379.

963. Limo, M.K., et al., *Purification and characterization of an anticoagulant from the salivary glands of the ixodid tick Rhipicephalus appendiculatus.* Exp Parasitol, 1991. **72**(4): p. 418-29.

964. Liu, S.J., et al., *Purification, characterization, crystallization and preliminary X-ray diffraction of acuthrombin-B, a thrombin-like enzyme from Agkistrodon acutus venom.* Acta Crystallogr D Biol Crystallogr, 1999. **55**(Pt 6): p. 1193-7.

965. Liu, X., et al., *A rhamnan-type sulfated polysaccharide with novel structure from Monostroma angicava Kjellm (Chlorophyta) and its bioactivity.* Carbohydr Polym, 2017. **173**: p. 732-748.

966. Loftus, J.P., et al., *Characterization of aminoaciduria and hypoaminoacidemia in dogs with hepatocutaneous syndrome.* Am J Vet Res, 2017. **78**(6): p. 735-744.

967. Lomonte, B., et al., *Isolation of basic myotoxins from Bothrops moojeni and Bothrops atrox snake venoms.* Toxicon, 1990. **28**(10): p. 1137-46.

968. Lu, C., et al., *Procoagulant activity of long-term stored red blood cells due to phosphatidylserine exposure.* Transfus Med, 2011. **21**(3): p. 150-7.

969. Lyon, M. and J.T. Gallagher, *Purification and partial characterization of the major cell-associated heparan sulphate proteoglycan of rat liver.* Biochem J, 1991. **273(Pt 2)**(Pt 2): p. 415-22.

970. Ma, J.F. and S. Hiradate, *Form of aluminium for uptake and translocation in buckwheat (Fagopyrum esculentum Moench).* Planta, 2000. **211**(3): p. 355-60.

971. Mackenzie, C.J., et al., *Comparison of two blood sampling techniques for the determination of coagulation parameters in the horse: Jugular venipuncture and indwelling intravenous catheter.* Equine Vet J, 2018. **50**(3): p. 333-338.

972. Majedi, S.M., B.C. Kelly, and H.K. Lee, *Efficient hydrophobization and solvent microextraction for determination of trace nano-sized silver and titanium dioxide in natural waters.* Anal Chim Acta, 2013. **789**: p. 47-57.

973. Mak, P., J.J. Enghild, and A. Dubin, *Hamster antithrombin III: purification, characterization and acute phase response.* Comp Biochem Physiol B Biochem Mol Biol, 1996. **115**(1): p. 135-41.

974. Malavolta, M., et al., *Speciation of trace elements in human serum by micro anion exchange chromatography coupled with inductively coupled plasma mass spectrometry.* Anal Biochem, 2012. **421**(1): p. 16-25.

975. Mamo, J.C., et al., *Hypertriglyceridemia is exacerbated by slow lipolysis of triacylglycerol-rich lipoproteins in fed but not fasted streptozotocin diabetic rats.* Biochim Biophys Acta, 1992. **1128**(2-3): p. 132-8.

976. Manco-Johnson, M.J., et al., *Hyperglycemia-induced hyperinsulinemia decreases maternal and fetal plasma protein C concentration during ovine gestation.* Pediatr Res, 1994. **36**(3): p. 293-9.

977. Manoharan, M. and P.O. Schwille, *Measurement of oxalate in human plasma ultrafiltrate by ion chromatography.* J Chromatogr B Biomed Sci Appl, 1997. **700**(1-2): p. 261-8.

978. Marcolino, V.A., T.C. Pimentel, and C.E. Barao, *What to expect from different drugs used in the treatment of COVID-19: A study on applications and in vivo and in vitro results.* European Journal of Pharmacology, 2020. **887**: p. 173467.

979. Marković, S., et al., *Speciation and Bio-Imaging of Chromium in Taraxacum officinale Using HPLC Post-column ID-ICP-MS, High Resolution MS and Laser Ablation ICP-MS Techniques.* Front Chem, 2022. **10**: p. 863387.

980. Marlaire, S. and C. Dehio, *Bartonella effector protein C mediates actin stress fiber formation via recruitment of GEF-H1 to the plasma membrane.* PLoS Pathog, 2021. **17**(1): p. e1008548.

981. Matsui, T., et al., *Purification and amino acid sequence of halystase from snake venom of Agkistrodon halys blomhoffii, a serine protease that cleaves specifically fibrinogen and kininogen.* Eur J Biochem, 1998. **252**(3): p. 569-75.

982. Matsumiya, H., H. Inoue, and M. Hiraide, *Separation of Gd-humic complexes and Gd-based magnetic resonance imaging contrast agent in river water with QAE-Sephadex A-25 for the fractionation analysis.* Talanta, 2014. **128**: p. 500-4.

983. Matsumoto, S., et al., *Metabolism of [carbonyl-14C]mosapride citrate after a single oral administration in rats, dogs and monkeys.* Arzneimittelforschung, 1993. **43**(10): p. 1095-102.

984. Meijers, B.K., et al., *Major coagulation disturbances during fractionated plasma separation and adsorption.* Am J Transplant, 2007. **7**(9): p. 2195-9.

985. Merces, A., et al., *Identification of blood plasma proteins using heparin-coated magnetic chitosan particles.* Carbohydr Polym, 2020. **247**: p. 116671.

986. Mercolini, L., et al., *Fast analysis of catecholamine metabolites MHPG and VMA in human plasma by HPLC with fluorescence detection and a novel SPE procedure.* Talanta, 2009. **78**(1): p. 150-5.

987. Milačič, R., et al., *Determination of Zn-citrate in human milk by CIM monolithic chromatography with atomic and mass spectrometry detection.* Talanta, 2012. **101**: p. 203-10.

988. Miller, R.K., et al., *Marginal transfer of ReoPro (Abciximab) compared with immunoglobulin G (F105), inulin and water in the perfused human placenta in vitro.* Placenta, 2003. **24**(7): p. 727-38.

989. Mitrović, B. and R. Milacic, *Speciation of aluminium in forest soil extracts by size exclusion chromatography with UV and ICP-AES detection and cation exchange fast protein liquid chromatography with ETAAS detection.* Sci Total Environ, 2000. **258**(3): p. 183-94.

990. Mojica-Henshaw, M.P., et al., *Serum-converted platelet lysate can substitute for fetal bovine serum in human mesenchymal stromal cell cultures.* Cytotherapy, 2013. **15**(12): p. 1458-68.

991. Moriarty, P., et al., *Comparison of different low density lipoprotein apheresis machines on brain natriuretic Peptide levels in patients with familial hypercholesterolemia.* Ther Apher Dial, 2010. **14**(1): p. 74-8.

992. Mozzi, R., et al., *Different mechanisms regulate phosphatidylserine synthesis in rat cerebral cortex.* Mol Cell Biochem, 1997. **168**(1-2): p. 41-9.

993. Murphy, M.F., et al., *Evaluation of Cobe Trima for the collection of blood components with particular reference to the in vitro characteristics of the red cell and platelet concentrates and the clinical responses to transfusion.* Transfus Sci, 2000. **22**(1-2): p. 39-43.

994. Nagaoka, M.H. and T. Maitani, *Differed preferential iron-binding lobe in human transferrin depending on the presence of bicarbonate detected by HPLC/high-resolution inductively coupled plasma mass spectrometry.* Biochim Biophys Acta, 2000. **1523**(2-3): p. 182-8.

995. Nakazawa, F., et al., *Extracellular RNA is a natural cofactor for the (auto-)activation of Factor VII-activating protease (FSAP).* Biochem J, 2005. **385**(Pt 3): p. 831-8.

996. Nandish, S.K.M., et al., *Flaxseed Cysteine Protease Exhibits Strong Anticoagulant, Antiplatelet, and Clot-Dissolving Properties.* Biochemistry (Mosc), 2020. **85**(9): p. 1113-1126.

997. Nette, D. and A. Seubert, *Determination of aminopolycarboxylic acids at ultra-trace levels by means of online coupling ion exchange chromatography and inductively coupled plasma-mass spectrometry with indirect detection via their Pd²⁺-complexes.* Anal Chim Acta, 2015. **884**: p. 124-32.

998. Nifong, T.P. and G.S. Gerhard, *Separation of IgG and IgM from albumin in citrated human plasma using electrodialysis and metal ion affinity precipitation.* Asaio j, 2002. **48**(6): p. 645-9.

999. Nishinaka, T., et al., *Up to 151 days of continuous animal perfusion with trivial heparin infusion by the application of a long-term durable antithrombogenic coating to a combination of a seal-less centrifugal pump and a diffusion membrane oxygenator.* J Artif Organs, 2007. **10**(4): p. 240-4.

1000. Nishinaka, T., et al., *At least thirty-four days of animal continuous perfusion by a newly developed extracorporeal membrane oxygenation system without systemic anticoagulants.* Artif Organs, 2002. **26**(6): p. 548-51.

1001. Norgard-Sumnicht, K.E., et al., *Unusual anionic N-linked oligosaccharides from bovine lung.* J Biol Chem, 1995. **270**(46): p. 27634-45.

1002. Nussbaum, R., et al., *Optical Detection of Heparin in Whole Blood Samples Using Nanosensors Embedded in an Agarose Hydrogel.* ACS Sens, 2022. **7**(12): p. 3956-3962.

1003. Obertacke, U., et al., *Local and systemic reactions after lung contusion: an experimental study in the pig.* Shock, 1998. **10**(1): p. 7-12.

1004. Oikawa, S., et al., *Impact of the platelet washing process on in vitro platelet properties, and the levels of soluble CD40 ligand and platelet-derived microparticles in the storage media.* Transfusion, 2019. **59**(3): p. 1080-1089.

1005. Oikawa, S., et al., *Comparative in vitro evaluation of apheresis platelets stored with 100% plasma versus bicarbonated Ringer's solution with less than 5% plasma.* Transfusion, 2013. **53**(3): p. 655-60.

1006. Oikawa, S., et al., *Storage of washed platelets in BRS-A platelet additive solutions based on two types of clinically available bicarbonated Ringer's solutions with different electrolyte concentrations.* Transfus Apher Sci, 2015. **53**(2): p. 233-7.

1007. Orthner, C.L., et al., *Large-scale production and properties of immunoaffinity-purified human activated protein C concentrate.* Vox Sang, 1995. **69**(4): p. 309-18.

1008. Ott, P., L. Bass, and S. Keiding, *Hepatic ICG removal in the pig depends on plasma protein and hematocrit: evidence of sinusoidal binding disequilibrium and unstirred water layer effects.* Hepatology, 1997. **26**(3): p. 679-90.

1009. Oupický, D., et al., *Steric stabilization of poly-L-Lysine/DNA complexes by the covalent attachment of semitelechelic poly[N-(2-hydroxypropyl)methacrylamide].* Bioconjug Chem, 2000. **11**(4): p. 492-501.

1010. Ouyang, X.K., et al., *Characterization and determination of chlorophacinone in plasma by ion chromatography coupled with ion trap electrospray ionization mass spectrometry.* Biomed Chromatogr, 2009. **23**(5): p. 524-30.

1011. Palm, M. and A. Lundblad, *Creatinine concentration in plasma from dog, rat, and mouse: a comparison of 3 different methods.* Vet Clin Pathol, 2005. **34**(3): p. 232-6.

1012. Parker, L.K., et al., *Dynamic monitoring of NET activity in mature murine sympathetic terminals using a fluorescent substrate.* Br J Pharmacol, 2010. **159**(4): p. 797-807.

1013. Passam, F.H., et al., *Redox control of β2-glycoprotein I-von Willebrand factor interaction by thioredoxin-1.* J Thromb Haemost, 2010. **8**(8): p. 1754-62.

1014. Peachey, E., et al., *Capabilities of mixed-mode liquid chromatography coupled to inductively coupled plasma mass spectrometry for the simultaneous speciation analysis of inorganic and organically-bound selenium.* J Chromatogr A, 2009. **1216**(42): p. 7001-6.

1015. Pellicer-Rubio, M.T., T. Magallon, and Y. Combarnous, *Deterioration of goat sperm viability in milk extenders is due to a bulbourethral 60-kilodalton glycoprotein with triglyceride lipase activity.* Biol Reprod, 1997. **57**(5): p. 1023-31.

1016. Persson, B.P., et al., *Heparin-binding protein (HBP/CAP37) - a link to endothelin-1 in endotoxemia-induced pulmonary oedema?* Acta Anaesthesiol Scand, 2014. **58**(5): p. 549-59.

1017. Persson, J. and P.O. Grände, *Plasma volume expansion and transcapillary fluid exchange in skeletal muscle of albumin, dextran, gelatin, hydroxyethyl starch, and saline after trauma in the cat.* Crit Care Med, 2006. **34**(9): p. 2456-62.

1018. Petzer, A.L., et al., *Evaluation of optimal survival of primitive progenitor cells (LTC-IC) from PBPC apheresis products after overnight storage.* Bone Marrow Transplant, 2000. **25**(2): p. 197-200.

1019. Peukert, A. and A. Seubert, *Characterization of an aluminium(III)-citrate species by means of ion chromatography with inductively coupled plasma-atomic emission spectrometry detection.* J Chromatogr A, 2009. **1216**(45): p. 7946-9.

1020. Pflaum, M., et al., *Towards Biohybrid Lung Development-Fibronectin-Coating Bestows Hemocompatibility of Gas Exchange Hollow Fiber Membranes by Improving Flow-Resistant Endothelialization.* Membranes (Basel), 2021. **12**(1).

1021. Pu, X.P. and S. Nagasawa, *Purification and characterization of PK-120, a novel substrate for plasma kallikrein, from guinea pig plasma.* Biol Pharm Bull, 1995. **18**(6): p. 837-41.

1022. Pulawski, E., et al., *Influence of single low-density lipoprotein apheresis on the adhesion molecules soluble vascular cellular adhesion molecule-1, soluble intercellular adhesion molecule-1, and P-selectin.* Ther Apher, 2002. **6**(3): p. 229-33.

1023. Rai, U., A. Rawal, and S. Singh, *Evaluation of the anti-inflammatory effect of an anti-platelet agent crinumin on carrageenan-induced paw oedema and granuloma tissue formation in rats.* Inflammopharmacology, 2018. **26**(3): p. 769-778.

1024. Raices, M. and M.A. D'Angelo, *Analysis of Nuclear Pore Complex Permeability in Mammalian Cells and Isolated Nuclei Using Fluorescent Dextrans.* Methods Mol Biol, 2022. **2502**: p. 69-80.

1025. Redl, H., et al., *Special collection and storage tubes for blood endotoxin and cytokine measurements.* Clinical Chemistry, 1992. **38**(5): p. 764-765.

1026. Reers, M., et al., *Synthesis and characterisation of novel thrombin inhibitors based on 4-amidinophenylalanine.* J Enzyme Inhib, 1995. **9**(1): p. 61-72.

1027. Reper, P., et al., *Early plasmapheresis as a successful treatment in hypertriglyceridemia-induced acute pancreatitis in first trimester pregnancy following in vitro fertilization.* European Journal of Obstetrics and Gynecology and Reproductive Biology, 2014. **179**: p. 257-258.

1028. Ricci, C.G., et al., *A thrombin inhibitor from the gut of Boophilus microplus ticks.* Exp Appl Acarol, 2007. **42**(4): p. 291-300.

1029. Richter, E., et al., *Platelet apheresis with Vivacell BT798 DEA, haemonetics V-50 and PCS-Plus: preparation efficiency and product quality using identical donors.* Infusionsther Transfusionsmed, 1992. **19**(5): p. 249-50.

1030. Richter, P., H. Fischer, and R. Dörfelt, *Immunoadsorption in a dog with severe immune mediated hemolytic anemia.* J Clin Apher, 2021. **36**(4): p. 668-672.

1031. Roberts, T.R., et al., *Tethered Liquid Perfluorocarbon Coating for 72 Hour Heparin-Free Extracorporeal Life Support.* Asaio j, 2021. **67**(7): p. 798-808.

1032. Rocha, J.C., et al., *Characterization of humic-rich hydrocolloids and their metal species by means of competing ligand and metal exchange--an on-site approach.* J Environ Monit, 2002. **4**(5): p. 799-802.

1033. Roeder, B., et al., *Evaluation of the Hemobag: a novel ultrafiltration system for circuit salvage.* J Extra Corpor Technol, 2004. **36**(2): p. 162-5.

1034. Roesner, J.P., et al., *A double blind, single centre, sub-chronic reperfusion trial evaluating FX06 following haemorrhagic shock in pigs.* Resuscitation, 2009. **80**(2): p. 264-71.

1035. Römisch, J., et al., *A protease isolated from human plasma activating factor VII independent of tissue factor.* Blood Coagul Fibrinolysis, 1999. **10**(8): p. 471-9.

1036. Römisch, J., et al., *The FVII activating protease cleaves single-chain plasminogen activators.* Haemostasis, 1999. **29**(5): p. 292-9.

1037. Rosenthal, M.G. and M.A. Labato, *Use of therapeutic plasma exchange to treat nonsteroidal anti-inflammatory drug overdose in dogs.* Journal of Veterinary Internal Medicine, 2019. **33**(2): p. 596-602.

1038. Roy, A.J., H.L. Bank, and W. Howard, *Perturbations of granulocyte counts induced by procedural, chemical and physiological events occurring during filtration leukapheresis in rats.* Vox Sang, 1983. **44**(1): p. 3-13.

1039. Rucavado, A., et al., *Characterization of aspercetin, a platelet aggregating component from the venom of the snake Bothrops asper which induces thrombocytopenia and potentiates metalloproteinase-induced hemorrhage.* Thromb Haemost, 2001. **85**(4): p. 710-5.

1040. Russo, G.E., et al., *Biocompatibility in hemapheresis: new materials.* Int J Artif Organs, 1993. **16 Suppl 5**: p. 214-6.

1041. Rydberg, L., et al., *Extracorporeal ("ex vivo") connection of pig kidneys to humans. II. The anti-pig antibody response.* Xenotransplantation, 1996. **3**(4): p. 340-53.

1042. S, J., et al., *In-line hemofiltration minimized extracorporeal membrane oxygenation-related inflammation in a porcine model.* Perfusion, 2014. **29**(6): p. 526-33.

1043. Sachin, H.R., et al., *Anticoagulant and antiplatelet activities of novel serine protease purified from seeds of Cucumis maderaspatensis.* 3 Biotech, 2021. **11**(1): p. 30.

1044. Saidenberg, E. and A. Tinmouth, *Ringer's lactate and red blood cells: Is there sufficient evidence to recommend for routine use?* Canadian Journal of Anesthesia, 2009. **56**(5): p. 343-347.

1045. San Agustin, J.T. and H.A. Lardy, *Bovine seminal plasma constituents modulate the activity of caltrin, the calcium-transport regulating protein of bovine spermatozoa.* J Biol Chem, 1990. **265**(12): p. 6360-7.

1046. Sandgren, P., et al., *Storage of Buffy-coat-derived platelets in additive solutions: in vitro effects on platelets prepared by the novel TACSI system and stored in plastic containers with different gas permeability.* Vox Sang, 2010. **99**(4): p. 341-7.

1047. Sandström, J., et al., *10-fold increase in human plasma extracellular superoxide dismutase content caused by a mutation in heparin-binding domain.* J Biol Chem, 1994. **269**(29): p. 19163-6.

1048. Saracino, M.A., et al., *Chromatographic analysis of serotonin, 5-hydroxyindolacetic acid and homovanillic acid in dried blood spots and platelet poor and rich plasma samples.* J Chromatogr A, 2010. **1217**(29): p. 4808-14.

1049. Schermuly, R.T., et al., *Chronic sildenafil treatment inhibits monocrotaline-induced pulmonary hypertension in rats.* Am J Respir Crit Care Med, 2004. **169**(1): p. 39-45.

1050. Schmitt, D. and F.H. Frimmel, *Ligand exchange rate of metal-NOM complexes by EDTA.* Environ Sci Pollut Res Int, 2003. **10**(1): p. 9-12.

1051. Schubert, P., et al., *Minimal impact of anticoagulant on in vitro whole blood quality throughout a 35-day cold-storage regardless of leukoreduction timing.* Transfusion, 2022. **62 Suppl 1**: p. S98-s104.

1052. Schwartz, G.J., et al., *Multicenter Laboratory Comparison of Iohexol Measurement.* J Appl Lab Med, 2018. **2**(5): p. 711-724.

1053. Schweighauser, A. and T. Francey, *Evaluation of prescription, anticoagulation, and metabolic response to membrane-based therapeutic plasma exchange in dogs.* Journal of Veterinary Internal Medicine, 2019. **33**(2): p. 1054-1055.

1054. Schwille, P.O., et al., *Media calcification, low erythrocyte magnesium, altered plasma magnesium, and calcium homeostasis following grafting of the thoracic aorta to the infrarenal aorta in the rat--differential preventive effects of long-term oral magnesium supplementation alone and in combination with alkali.* Biomed Pharmacother, 2003. **57**(2): p. 88-97.

1055. Seedevi, P., et al., *Structural characterization and bioactivities of sulfated polysaccharide from Monostroma oxyspermum.* Int J Biol Macromol, 2015. **72**: p. 1459-65.

1056. Sekiguchi, T., et al., *Clinical Application of Apheresis in Very Small Dogs Weighing <8 kg to Pediatric Patients.* Therapeutic Apheresis and Dialysis, 2020. **24**(3): p. 333-342.

1057. Seré, K.M., et al., *Protein S multimers are generated in vitro and affect protein S structure-function analyses.* Semin Hematol, 2006. **43**(1 Suppl 1): p. S111-20.

1058. Serrano, K., et al., *Plasma and cryoprecipitate manufactured from whole blood held overnight at room temperature meet quality standards.* Transfusion, 2010. **50**(2): p. 344-53.

1059. Serrick, C.J., et al., *Quality of red blood cells using autotransfusion devices: a comparative analysis.* J Extra Corpor Technol, 2003. **35**(1): p. 28-34.

1060. Shaw, K. and J.H. Exton, *Identification in bovine liver plasma membranes of a Gq-activatable phosphoinositide phospholipase C.* Biochemistry, 1992. **31**(27): p. 6347-54.

1061. Shimamoto, A., et al., *Biocompatibility of silicone-coated oxygenator in cardiopulmonary bypass.* Ann Thorac Surg, 2000. **69**(1): p. 115-20.

1062. Shupe, J.L., et al., *Placental transfer of fluoride in Holstein cows.* Vet Hum Toxicol, 1992. **34**(1): p. 1-4.

1063. Singh, B., et al., *Monoclonal and Oligoclonal Anti-PF4 Antibodies Mediate VITT.* Blood, 2021. **138**(Supplement 1): p. 3220.

1064. Skoog, D.J., et al., *Fourteen Day In Vivo Testing of a Compliant Thoracic Artificial Lung.* Asaio j, 2017. **63**(5): p. 644-649.

1065. Smeland, S., et al., *Binding of perlecan to transthyretin in vitro.* Biochem J, 1997. **326 ( Pt 3)**(Pt 3): p. 829-36.

1066. Smith, J. and G. Rock, *Protein quality in Mirasol pathogen reduction technology-treated, apheresis-derived fresh-frozen plasma.* Transfusion, 2010. **50**(4): p. 926-31.

1067. Snyder, E.L., et al., *In vitro and in vivo evaluation of a whole blood platelet-sparing leukoreduction filtration system.* Transfusion, 2010. **50**(10): p. 2145-51.

1068. Soda, Y., et al., *Protamine/heparin optical nanosensors based on solvatochromism.* Chem Sci, 2021. **12**(47): p. 15596-15602.

1069. Sohrabipour, S., et al., *Mechanistic Studies of DNase I Activity: Impact of Heparin Variants and PAD4.* Shock, 2021. **56**(6): p. 975-987.

1070. Solovyev, N., et al., *Redox speciation of iron, manganese, and copper in cerebrospinal fluid by strong cation exchange chromatography - sector field inductively coupled plasma mass spectrometry.* Anal Chim Acta, 2017. **973**: p. 25-33.

1071. Soltys, P.J. and M.R. Etzel, *In vitro characterization of a membrane-based low-density lipoprotein affinity adsorption device.* Blood Purif, 1998. **16**(3): p. 123-34.

1072. Sone, J., et al., *Assessment of bilirubin clearance capacity of a newly developed ion-exchange adsorption column and its possible use as a supportive therapy in hepatorenal syndrome.* J Clin Apher, 1990. **5**(3): p. 123-7.

1073. Sorensen, B.S., A.M. Thomsen, and B.K. Moller, *Collection of peripheral blood progenitor cells for autologous use: Performance enhancements of COBE spectra, auto-PBSC.* Journal of Clinical Apheresis, 2011. **26**(6): p. 307-314.

1074. Soubeyrand, S., et al., *Purification of a novel phospholipase A2 from bovine seminal plasma.* J Biol Chem, 1997. **272**(1): p. 222-7.

1075. Souri, M., et al., *Unique secretion mode of human protein Z: its Gla domain is responsible for inefficient, vitamin K-dependent and warfarin-sensitive secretion.* Blood, 2009. **113**(16): p. 3857-64.

1076. Spannagl, M., et al., *A purified antithrombin III--heparin complex as a potent inhibitor of thrombin in porcine endotoxin shock.* Thromb Res, 1991. **61**(1): p. 1-10.

1077. Srivastava, P., et al., *Bis (histidine) with N2 vehicle: an important skeleton for MR/chelation therapy.* Chem Biol Drug Des, 2014. **83**(6): p. 682-7.

1078. Staff, K., et al., *Recovering Ga(III) from coordination complexes using pyridine 2,6-dicarboxylic acid chelation ion chromatography.* Biomed Chromatogr, 2010. **24**(9): p. 1015-22.

1079. Stanislawska, M., B. Janasik, and W. Wasowicz, *Application of high performance liquid chromatography with inductively coupled plasma mass spectrometry (HPLC-ICP-MS) for determination of chromium compounds in the air at the workplace.* Talanta, 2013. **117**: p. 14-9.

1080. Steiner, V., et al., *Isolation and purification of novel hirudins from the leech Hirudinaria manillensis by high-performance liquid chromatography.* J Chromatogr, 1990. **530**(2): p. 273-82.

1081. Stulc, J. and B. Stulcová, *Placental transfer of phosphate in anaesthetized rats.* Placenta, 1996. **17**(7): p. 487-93.

1082. Suenaga, E., et al., *Experimental use of a compact centrifugal pump and membrane oxygenator as a cardiopulmonary support system.* Artif Organs, 2000. **24**(11): p. 912-5.

1083. Sugihara, T., et al., *Isolation of recombinant human antithrombin isoforms by Cellufine Sulfate affinity chromatography.* J Chromatogr B Analyt Technol Biomed Life Sci, 2018. **1095**: p. 198-203.

1084. Sukavaneshvar, S., et al., *Mitigation of coagulation by removing clotting factors part 2: heparin-free extracorporeal circulation in a porcine model.* Asaio j, 2007. **53**(4): p. 421-7.

1085. Suleria, H.A., et al., *In vitro Anti-Thrombotic Activity of Extracts from Blacklip Abalone (Haliotis rubra) Processing Waste.* Mar Drugs, 2016. **15**(1).

1086. Suleria, H.A.R., et al., *In vitro anti-thrombotic and anti-coagulant properties of blacklip abalone (Haliotis rubra) viscera hydrolysate.* Anal Bioanal Chem, 2017. **409**(17): p. 4195-4205.

1087. Sumida, E., et al., *Platelet separation from whole blood in an aqueous two-phase system with water-soluble polymers.* J Pharmacol Sci, 2006. **101**(1): p. 91-7.

1088. Sun, Q.B., et al., *Phosphorus enhances Al resistance in Al-resistant Lespedeza bicolor but not in Al-sensitive L. cuneata under relatively high Al stress.* Ann Bot, 2008. **102**(5): p. 795-804.

1089. Susa, J.B., et al., *Chronic hyperinsulinemia in the fetal rhesus monkey: effects of physiologic hyperinsulinemia on fetal substrates, hormones, and hepatic enzymes.* Am J Obstet Gynecol, 1984. **150**(4): p. 415-20.

1090. Szuchet, S., K. Watanabe, and Y. Yamaguchi, *Differentiation/regeneration of oligodendrocytes entails the assembly of a cell-associated matrix.* Int J Dev Neurosci, 2000. **18**(7): p. 705-20.

1091. Tam, S.C., J. Blumenstein, and J.T. Wong, *Blood replacement in dogs by dextran-hemoglobin.* Can J Biochem, 1978. **56**(10): p. 981-4.

1092. Tanaka, A.S., et al., *Purification and primary structure determination of a Bowman-Birk trypsin inhibitor from Torresea cearensis seeds.* Biol Chem, 1997. **378**(3-4): p. 273-81.

1093. Tanaka, S., et al., *A hollow-fibre column system to effectively prepare washed platelets.* Vox Sanguinis, 2015. **109**(3): p. 239-247.

1094. Tanaka-Azevedo, A.M., A.S. Tanaka, and I.S. Sano-Martins, *A new blood coagulation inhibitor from the snake Bothrops jararaca plasma: isolation and characterization.* Biochem Biophys Res Commun, 2003. **308**(4): p. 706-12.

1095. Tanhehco, Y.C., A.H. Rux, and B.S. Sachais, *Low-density lipoprotein apheresis reduces platelet factor 4 on the surface of platelets: a possible protective mechanism against heparin-induced thrombocytopenia and thrombosis.* Transfusion, 2011. **51**(5): p. 1022-9.

1096. Tao, J., et al., *Umbilical cord blood-derived mesenchymal stem cells in treating a critically ill COVID-19 patient.* Journal of Infection in Developing Countries, 2020. **14**(10): p. 1138-1145.

1097. Tashiro, M., et al., *Experimental evaluation of the V-point heparin-bonding system applied to a dense-membrane artificial lung during 24-hour extracorporeal circulation in beagles.* Artif Organs, 2001. **25**(8): p. 655-63.

1098. Tatsumi, E., et al., *Development of an ultracompact integrated heart-lung assist device.* Artif Organs, 1999. **23**(6): p. 518-23.

1099. Taurino, G., et al., *Mesenchymal stromal cells cultured in physiological conditions sustain citrate secretion with glutamate anaplerosis.* Mol Metab, 2022. **63**: p. 101532.

1100. Taylor, S.J., J.A. Smith, and J.H. Exton, *Purification from bovine liver membranes of a guanine nucleotide-dependent activator of phosphoinositide-specific phospholipase C. Immunologic identification as a novel G-protein alpha subunit.* J Biol Chem, 1990. **265**(28): p. 17150-6.

1101. Teh, L.C. and M. Froger, *Direct capture of plasma factor VIII:C by ion exchange chromatography.* Vox Sang, 1994. **67**(1): p. 8-13.

1102. Teixeira, D.I., et al., *Ion-exchange chromatography used to isolate a spermadhesin-related protein from domestic goat (Capra hircus) seminal plasma.* Genet Mol Res, 2006. **5**(1): p. 79-87.

1103. Telgmann, L., et al., *Speciation of Gd-based MRI contrast agents and potential products of transmetalation with iron ions or parenteral iron supplements.* Anal Bioanal Chem, 2012. **404**(8): p. 2133-41.

1104. ten Brinke, M.J., et al., *Leukocyte removal efficiency of cell-washed and unwashed whole blood: an in vitro study.* Perfusion, 2005. **20**(6): p. 335-41.

1105. Tevaearai, H.T., et al., *Flow rate dependent ex vivo deheparinization with immobilized cationic ligand.* Asaio j, 1997. **43**(5): p. M487-9.

1106. Thérien, I., et al., *Isolation and characterization of glycosaminoglycans from bovine follicular fluid and their effect on sperm capacitation.* Mol Reprod Dev, 2005. **71**(1): p. 97-106.

1107. Thiele, T., et al., *Thrombin generation, ProC(®)Global, prothrombin time and activated partial thromboplastin time in thawed plasma stored for seven days and after methylene blue/light pathogen inactivation.* Blood Transfus, 2016. **14**(1): p. 66-72.

1108. Timmerman, M., et al., *Effect of dexamethasone on fetal hepatic glutamine-glutamate exchange.* Am J Physiol Endocrinol Metab, 2000. **278**(5): p. E839-45.

1109. Tiyanont, K., et al., *Insights into Notch3 activation and inhibition mediated by antibodies directed against its negative regulatory region.* J Mol Biol, 2013. **425**(17): p. 3192-204.

1110. Tongyai, S., et al., *Mechanism of increased erythrocyte membrane fluidity during magnesium deficiency in weanling rats.* Am J Physiol, 1989. **257**(2 Pt 1): p. C270-6.

1111. Torrent, L., et al., *Interaction of silver nanoparticles with mediterranean agricultural soils: Lab-controlled adsorption and desorption studies.* J Environ Sci (China), 2019. **83**: p. 205-216.

1112. Tripodi, A., M. Moia, and V. Pengo, *False-negative or false-positive: Laboratory diagnosis of lupus anticoagulant at the time of commencement of anticoagulant: A rebuttal.* Journal of Thrombosis and Haemostasis, 2011. **9**(7): p. 1435-1436.

1113. Tsai, A.G., P. Cabrales, and M. Intaglietta, *Microvascular perfusion upon exchange transfusion with stored red blood cells in normovolemic anemic conditions.* Transfusion, 2004. **44**(11): p. 1626-34.

1114. Tsai, C.Y. and S.J. Jiang, *Microwave-assisted extraction and ion chromatography dynamic reaction cell inductively coupled plasma mass spectrometry for the speciation analysis of arsenic and selenium in cereals.* Anal Sci, 2011. **27**(3): p. 271-6.

1115. Turaga, K.K., P. Chakradhara Rao, and G. Sripad, *Rapid purification of high purity thrombin and preparation of a novel hemostat for clinical purposes.* Indian J Hematol Blood Transfus, 2008. **24**(2): p. 54-8.

1116. Unal, S., et al., *A novel mutation in protein C gene (PROC) causing severe phenotype in neonatal period.* Pediatric Blood and Cancer, 2014. **61**(4): p. 763-764.

1117. Unger, J.K., et al., *Albumin and hydroxyethyl starch 130 kDa/0.4 improve filter clearance and haemocompatibility in haemo- and plasmafiltration - An in vitro study.* Nephrology Dialysis Transplantation, 2005. **20**(9): p. 1922-1931.

1118. Unger, J.K., et al., *Maximal flow rates and sieving coefficients in different plasmafilters: effects of increased membrane surfaces and effective length under standardized in vitro conditions.* J Clin Apher, 2002. **17**(4): p. 190-8.

1119. Unger, J.K., et al., *Influence of different heparin concentrations on the results of in vitro investigations in plasmaseparation technology using capillary membrane filters.* Artif Organs, 2003. **27**(7): p. 649-57.

1120. Unger, J.K., et al., *Hydroxyethyl starch 130 kd/0.4 and albumin improve CVVH biocompatibility whereas gelatin and hydroxyethyl starch 200 kd/0.5 lead to adverse side effects of CVVH in anesthetized pigs.* Shock, 2006. **25**(5): p. 533-45.

1121. Unger, J.K., et al., *The influence of hypoalbuminemia on maximal flow rates and transmembrane pressure during plasmapheresis--an in vitro study.* Blood Purif, 2001. **19**(4): p. 408-16.

1122. Unger, J.K., et al., *The influence of hypoalbuminemia on maximal flow rates and transmembrane pressure during plasmapheresis - An in vitro study.* Blood Purification, 2001. **19**(4): p. 408-416.

1123. Unger, J.K., et al., *Enhancing filtration rates by the use of blood flow around the capillaries of plasmafilters: an in vitro study.* Int J Artif Organs, 2001. **24**(11): p. 821-31.

1124. Vagianos, C., et al., *Reversal of lethal citrate intoxication by intravenous infusion of calcium. An experimental study in pigs.* Acta Chirurgica Scandinavica, 1990. **156**(10): p. 671-675.

1125. van Beek, A.E., et al., *Factor H-Related (FHR)-1 and FHR-2 Form Homo- and Heterodimers, while FHR-5 Circulates Only As Homodimer in Human Plasma.* Front Immunol, 2017. **8**: p. 1328.

1126. van der Weyden, L., P.G. Hains, and K.W. Broady, *Characterisation of the biochemical and biological variations from the venom of the death adder species (Acanthophis antarcticus, A. praelongus and A. pyrrhus).* Toxicon, 2000. **38**(12): p. 1703-13.

1127. Vassallo, R.R., et al., *In vitro and in vivo evaluation of apheresis platelets stored for 5 days in 65% platelet additive solution/35% plasma.* Transfusion, 2010. **50**(11): p. 2376-85.

1128. Vesterqvist, O., F. Nabbie, and B. Swanson, *Determination of metformin in plasma by high-performance liquid chromatography after ultrafiltration.* J Chromatogr B Biomed Sci Appl, 1998. **716**(1-2): p. 299-304.

1129. Vitalo, A., G. Buckley, and L. Londono, *Therapeutic plasma exchange as adjunct therapy in 3 dogs with myasthenia gravis and myasthenia-like syndrome.* Journal of Veterinary Emergency and Critical Care, 2021. **31**(1): p. 106-111.

1130. Waerhaug, K., et al., *Recombinant human activated protein C attenuates endotoxin-induced lung injury in awake sheep.* Crit Care, 2008. **12**(4): p. R104.

1131. Wagner, S.J., et al., *Calcium is a key constituent for maintaining the in vitro properties of platelets suspended in the bicarbonate-containing additive solution M-sol with low plasma levels.* Transfusion, 2010. **50**(5): p. 1028-35.

1132. Waidhet-Kouadio, P., et al., *Purification and characterization of a thrombin inhibitor from the salivary glands of a malarial vector mosquito, Anopheles stephensi.* Biochim Biophys Acta, 1998. **1381**(2): p. 227-33.

1133. Walton, S., et al., *Treatment of ibuprofen intoxication in a dog via therapeutic plasma exchange.* J Vet Emerg Crit Care (San Antonio), 2017. **27**(4): p. 451-457.

1134. Walton, S., et al., *Treatment of meloxicam overdose in a dog via therapeutic plasma exchange.* J Vet Emerg Crit Care (San Antonio), 2017. **27**(4): p. 444-450.

1135. Wang, L., et al., *Tissue distribution and elimination of [14C]apixaban in rats.* Drug Metab Dispos, 2011. **39**(2): p. 256-64.

1136. Wang, X., et al., *Development of a chiral micellar electrokinetic chromatography-tandem mass spectrometry assay for simultaneous analysis of warfarin and hydroxywarfarin metabolites: application to the analysis of patients serum samples.* J Chromatogr A, 2013. **1271**(1): p. 207-16.

1137. Wang, X., Q. Wang, and W. Qin, *A moving-part-free protamine-sensitive polymeric membrane electrode for sensitive biomedical analyses.* Biosens Bioelectron, 2012. **38**(1): p. 145-50.

1138. Wang, Z., et al., *Beneficial effects of recombinant human activated protein C in a ewe model of septic shock.* Crit Care Med, 2007. **35**(11): p. 2594-600.

1139. Warkentin, T.E., et al., *The platelet serotonin-release assay.* American Journal of Hematology, 2015. **90**(6): p. 564-572.

1140. Warner, T.G., et al., *Isolation and properties of a soluble sialidase from the culture fluid of Chinese hamster ovary cells.* Glycobiology, 1993. **3**(5): p. 455-63.

1141. Watson, K., et al., *Nucleosomes bind to cell surface proteoglycans.* J Biol Chem, 1999. **274**(31): p. 21707-13.

1142. Wearden, P.D., et al., *Respiratory dialysis with an active-mixing extracorporeal carbon dioxide removal system in a chronic sheep study.* Intensive Care Med, 2012. **38**(10): p. 1705-11.

1143. Weber, L.L., L.D. Roberts, and J.D. Sweeney, *Residual plasma in red blood cells and transfusion-related acute lung injury.* Transfusion, 2014. **54**(10): p. 2425-30.

1144. Weinstein, R.E., F.R. Rickles, and F.J. Walker, *Purification and preliminary characterization of rabbit vitamin K-dependent coagulation proteins.* Thromb Res, 1990. **59**(4): p. 759-72.

1145. Weinstein, S.A., *Bites from non-front-fanged snakes: Medical significance, troublesome terminology and taxing taxonomy.* Toxicon, 2020. **182**(Supplement 1): p. S20-S21.

1146. Weinstein, S.A., et al., *Non-front-fanged colubroid snakes: a current evidence-based analysis of medical significance.* Toxicon, 2013. **69**: p. 103-13.

1147. Wiegand, U.W. and G. Levy, *Effect of heparin injection on plasma protein binding of bilirubin and salicylate in rats.* J Pharm Sci, 1979. **68**(12): p. 1483-6.

1148. Wiltshire, M., R. Cardigan, and S. Thomas, *Manufacture of red cells in additive solution from whole blood refrigerated for 5 days or remanufactured from red cells stored in plasma.* Transfus Med, 2010. **20**(6): p. 383-91.

1149. Wu, Q., W.M. Pierce, Jr., and N.A. Delamere, *Cytoplasmic pH responses to carbonic anhydrase inhibitors in cultured rabbit nonpigmented ciliary epithelium.* J Membr Biol, 1998. **162**(1): p. 31-8.

1150. Wu, R.P., et al., *Protective effect of low potassium dextran solution on acute kidney injury following acute lung injury induced by oleic acid in piglets.* Chin Med J (Engl), 2012. **125**(17): p. 3093-7.

1151. Xu, Y., et al., *Simple emulsion template method towards self-anticoagulant and high-efficiency carboxymethyl chitosan-based adsorbent for low-density lipoprotein from whole blood.* J Colloid Interface Sci, 2023. **631**(Pt A): p. 231-244.

1152. Xue, Q., et al., *CEMIP regulates the proliferation and migration of vascular smooth muscle cells in atherosclerosis through the WNT-beta-catenin signaling pathway.* Biochem Cell Biol, 2020. **98**(2): p. 249-257.

1153. Yamada, T., et al., *Effect of in-vivo administration of nafamostat on the onset of renal hyperkalemia and association of urine kallikrein in rats.* Dokkyo Journal of Medical Sciences, 2021. **48**(1): p. 33-42.

1154. Yan, S.B., *Review of conformation-specific affinity purification methods for plasma vitamin K-dependent proteins.* J Mol Recognit, 1996. **9**(3): p. 211-8.

1155. Yang, X., et al., *Proteomic analysis for process development and control of therapeutic protein separation from human plasma.* Electrophoresis, 2009. **30**(7): p. 1185-93.

1156. Yin, S., et al., *Molecularly-imprinted hydrogel beads via self-sacrificing micro-reactors as safe and selective bilirubin adsorbents.* J Mater Chem B, 2022. **10**(14): p. 2534-2543.

1157. Yokel, R.A. and J.S. Crossgrove, *Manganese toxicokinetics at the blood-brain barrier.* Res Rep Health Eff Inst, 2004(119): p. 7-58; discussion 59-73.

1158. You, J.G., et al., *Colorimetric assay of heparin in plasma based on the inhibition of oxidase-like activity of citrate-capped platinum nanoparticles.* Biosens Bioelectron, 2017. **92**: p. 442-448.

1159. Yuan, C., et al., *Highly sensitive and selective measurement of underivatized methylmalonic acid in serum and plasma by liquid chromatography-tandem mass spectrometry.* Anal Bioanal Chem, 2012. **404**(1): p. 133-40.

1160. Yuasa, S., R. Akagi, and T. Ubuka, *Determination of hypotaurine and taurine in blood plasma of rats after the administration of L-cysteine.* Acta Med Okayama, 1990. **44**(1): p. 47-50.

1161. Zaman, S.U., et al., *Biocompatible chicken bone extracted dahllite/hydroxyapatite/collagen filler based polysulfone membrane for dialysis.* Int J Artif Organs, 2022. **45**(1): p. 14-26.

1162. Zanella, A., et al., *Ion-Exchange Resin Anticoagulation (I-ERA): A Novel Extracorporeal Technique for Regional Anticoagulation.* Shock, 2016. **46**(3): p. 304-11.

1163. Zhang, F., et al., *Microscale isolation and analysis of heparin from plasma using an anion-exchange spin column.* Anal Biochem, 2006. **353**(2): p. 284-6.

1164. Zhang, Y., et al., *Non-fusion expression in Escherichia coli, purification, and characterization of a novel Ca2+- and phospholipid-binding protein annexin B1.* Protein Expr Purif, 2004. **34**(1): p. 68-74.

1165. Zhao, M., et al., *Quantification of 5-azacytidine in plasma by electrospray tandem mass spectrometry coupled with high-performance liquid chromatography.* J Chromatogr B Analyt Technol Biomed Life Sci, 2004. **813**(1-2): p. 81-8.

1166. Zhao, Y. and D.C. Sane, *Expression of a recombinant baculovirus for vitronectin in insect cells: purification, characterization of post-translational modifications and functional studies of the recombinant protein.* Arch Biochem Biophys, 1993. **304**(2): p. 434-42.

1167. Zhou, G.X., L. Chao, and J. Chao, *Kallistatin: a novel human tissue kallikrein inhibitor. Purification, characterization, and reactive center sequence.* J Biol Chem, 1992. **267**(36): p. 25873-80.

1168. Zimmerli, B., B. O'Neill, and P.J. Meier, *Identification of sodium-dependent and sodium-independent dicarboxylate transport systems in rat liver basolateral membrane vesicles.* Pflugers Arch, 1992. **421**(4): p. 329-35.

1169. Zuo, Z., et al., *Simultaneous measurement of S-warfarin, R-warfarin, S-7-hydroxywarfarin and R-7-hydroxywarfarin in human plasma by liquid chromatography-tandem mass spectrometry.* J Pharm Biomed Anal, 2010. **52**(2): p. 305-10.

1170. *A Study to find out the effect of blood purification (replacing the liquid part of blood) in improving the survival of children with severe infection and organ failure.* Effect of Therapeutic Plasma Exchange in the outcome of Pediatric Severe Sepsis -A Pilot Randomized Controlled Trial, 2021.

1171. Affifi, M., *Plasmapheresis: As a modality of therapy for treatment of autoimmune disordersinchildren.* Nephrology Dialysis Transplantation, 2018. **33**(Supplement 1): p. i310.

1172. Agarwal, N., et al., *Catastrophic antiphospholipid antibody syndrome in a child with thrombotic microangiopathy.* Indian Journal of Nephrology, 2012. **22**(4): p. 310-313.

1173. Aguiar, C.L., et al., *Pediatric Antiphospholipid Syndrome.* Current Rheumatology Reports, 2015. **17**(4).

1174. Aksu Uzunhan, T., et al., *Cytotoxic lesions of the corpus callosum in children: Etiology, clinical and radiological features, and prognosis.* Brain and Development, 2021. **43**(9): p. 919-930.

1175. Akturk, A., et al., *Microsurgery for "wrist" arteriovenous fistula creation in children: a retrospective cohort study.* Journal of Vascular Access, 2018. **19**(2): p. 137-140.

1176. Aldarweesh, F., et al., *A problem with the mean: Impact of accurate hematocrit of blood prime units on post-pheresis hematocrit in very small children.* Journal of Clinical Apheresis, 2018. **33**(2): p. 176-177.

1177. Almoshary, M.A., R. Alswyeh, and B.M.B. Edrees, *Successful Treatment of Atypical Hemolytic Uremic Syndrome With Therapeutic Plasma Exchange in a 3.8-kg Neonate.* Therapeutic Apheresis and Dialysis, 2017. **21**(2): p. 207-208.

1178. Amin, R., et al., *Challenges associated with concurrent plasma exchange and continuous renal replacement therapy in a 3.9 kilogram infant.* Journal of Clinical Apheresis, 2015. **30**(2): p. 123-124.

1179. Anderson, M., et al., *Paediatric protein s deficiency-a case study.* Journal of Clinical Apheresis, 2013. **28**(2): p. 111.

1180. Andreoli, S.P., et al., *Hemolytic uremic syndrome: Epidemiology, pathophysiology, and therapy. Proceedings of the American Society of Pediatric Nephrology Educational Symposium, May 2000, Boston, Massachusetts, USA.* Pediatric Nephrology, 2002. **17**(4): p. 293-298.

1181. Arena, R., et al., *Management of anticoagulation during therapeutic plasma exchange in tandem with extracorporeal membrane oxygenation (ECMO) in critically Ill children.* Perfusion, 2021. **36**(1 SUPPL): p. 67-68.

1182. Arici, Z.S., et al., *Treatment-resistant patient with antiphospholipid syndrome associated with pediatric systemic lupus erythematosus.* Lupus, 2016. **25**(Supplement 1): p. 57.

1183. Arni, D., et al., *Successful liver transplantation in a child with acute-on-chronic liver failure and acquired thrombotic thrombocytopenic purpura.* Liver Transplantation, 2015. **21**(5): p. 704-706.

1184. Arrington, A.S., et al., *The safety of therapeutic plasma exchange in pediatric end stage liver disease.* Hepatology, 2014. **60**(SUPPL. 1): p. 551A.

1185. Askenazi, D., et al., *Smaller circuits for smaller patients: improving renal support therapy with AquadexTM.* Pediatric Nephrology, 2016. **31**(5): p. 853-860.

1186. Atay, G. and D. Demirkol, *Therapeutic Plasma Exchange Application in Children Requires Individual Decision.* Journal of Pediatric Intensive Care, 2021. **10**(2): p. 106-109.

1187. Aygün, F., et al., *Evaluation of Continuous Renal Replacement Therapy and Therapeutic Plasma Exchange, in Severe Sepsis or Septic Shock in Critically Ill Children.* Medicina (Kaunas), 2019. **55**(7).

1188. Baez, V.L., et al., *Security of therapeutic apheresis in pediatrics. prospective study during 2018 in 171 apheresis sesssions.* Nephrology Dialysis Transplantation, 2020. **35**(SUPPL 3): p. iii180.

1189. Basu, B., et al., *Efficacy and outcomes of continuous peritoneal dialysis versus daily intermittent hemodialysis in pediatric acute kidney injury.* Pediatric Nephrology, 2016. **31**(10): p. 1681-1689.

1190. Bayrakci, U.S., et al., *Direct adsorption of lipoproteins from whole blood by direct adsorption of lipoprotein apheresis: First experience in two hypercholesterolemic children.* Therapeutic Apheresis and Dialysis, 2005. **9**(6): p. 469-472.

1191. Belousova, T., et al., *Utilization of therapeutic plasma exchange for hyperbilirubinemia in a premature newborn on extracorporeal membrane oxygenation.* J Clin Apher, 2019. **34**(5): p. 615-622.

1192. Betti, S., et al., *Successful ABO-incompatible kidney transplantation in children.* Pediatric Nephrology, 2012. **27**(9): p. 1632-1633.

1193. Biasuzzi, A., et al., *Single-needle hemodialysis combined with plasma exchange in a child with difficult vascular access.* Pediatric Nephrology, 2012. **27**(9): p. 1801.

1194. Biasuzzi, A., et al., *Single-needle plasma-exchange in a child.* Hemodialysis International, 2012. **16**(1): p. 145.

1195. Bilen, O., L. Loftis, and J. Teruya, *Severe thrombotic and bleeding complications in a baby with heterozygous factor V Leiden and acquired von Willebrand disease on ECMO.* Journal of Extra-Corporeal Technology, 2011. **43**(2): p. 64-69.

1196. Bilginer, Y., et al., *Outcome of primary glomerular disease in pediatric renal transplantation: a single-center experience.* Transplant Proc, 2008. **40**(1): p. 129-31.

1197. Blanco, B., et al., *Plasma Lipoprotein(a) and factor VIII levels after pediatric renal transplantation: A 10-year experience from a tertiary center in Brazil.* Research and Practice in Thrombosis and Haemostasis, 2022. **6**(Supplement 1).

1198. Brancaccio, G., et al., *Mechanical Assist Device as a Bridge to Heart Transplantation in Children Less Than 10 Kilograms.* Annals of Thoracic Surgery, 2010. **90**(1): p. 58-62.

1199. Bruun, C.F., B. Langbakk, and S. Eriksson Steigen, *Purpura fulminans as a sequel to erythema nodosum in a child with homozygous Leiden mutation and acquired protein S deficiency.* Acta Paediatr, 2005. **94**(8): p. 1155-8.

1200. Chen, G.M., et al., *Plasma exchange parameter selection and safety observation of children with severe ricinism.* Genet Mol Res, 2015. **14**(2): p. 4169-76.

1201. Chen, J.M., et al., *A decade of pediatric mechanical circulatory support before and after cardiac transplantation.* Journal of Thoracic and Cardiovascular Surgery, 2012. **143**(2): p. 344-351.

1202. Chetan, D., et al., *Successful Treatment of Pediatric Ventricular Assist Device Thrombosis.* Asaio j, 2018. **64**(2): p. e28-e32.

1203. Choleva, L., C. Romero, and M. Wilkes, *Use of insulin in the acute management of severe hypertriglyceridemia in pediatric patients with hematologic malignancy: A case series.* Hormone Research in Paediatrics, 2020. **93**(SUPPL 1): p. 27-28.

1204. Ciechanska, E., et al., *Plasma exchange using a continuous venovenous hemofiltration machine in children.* Blood Purif, 2005. **23**(6): p. 440-5.

1205. Coppo, R., *Pediatric IgA Nephropathy: Clinical and Therapeutic Perspectives.* Seminars in Nephrology, 2008. **28**(1): p. 18-26.

1206. Cordoba Buritica, J.P., Z. Espitaleta Vergara, and C. Larrarte Arenas, *Plasmapheresis in children: A university hospital experience in Bogota, Colombia.* Transfusion and Apheresis Science, 2012. **47**(SUPPL. 1): p. S36-S37.

1207. Czubkowski, P., et al., *Immune-mediated hemolytic anemia in children after liver and small bowel transplantation.* Liver Transpl, 2011. **17**(8): p. 921-4.

1208. Datla, S., et al., *Use of therapeutic plasma exchange (TPE) using the spectra optiavr for acute disseminated encephalomyelitis/acute hemorrhagic encephalomyelitis in an 11.4 kg patient.* Journal of Clinical Apheresis, 2013. **28**(2): p. 137-138.

1209. de Geyer, A., et al., *EXTENSIVE NECROTIC SKIN LESIONS DUE TO POSTVARICELLA PROTEIN S DEFICIENCY.* Pediatric Infectious Disease Journal, 2018. **37**(7): p. E201-E203.

1210. Delaney, M., et al., *An international survey of pediatric apheresis practice.* Journal of Clinical Apheresis, 2014. **29**(2): p. 120-126.

1211. Delos Santos, N.M. and R.J. Wyatt, *Pediatric IgA nephropathies: Clinical aspects and therapeutic approaches.* Seminars in Nephrology, 2004. **24**(3): p. 269-286.

1212. Demir, S., et al., *The challenges in diagnosing pediatric primary antiphospholipid syndrome.* Lupus, 2022. **31**(10): p. 1269-1275.

1213. Demirkol, D., et al., *Hyperferritinemia in the critically ill child with secondary hemophagocytic lymphohistiocytosis/sepsis/multiple organ dysfunction syndrome/macrophage activation syndrome: What is the treatment?* Critical Care, 2012. **16**(2): p. R52.

1214. Di Mola, M., N. Jawa, and C. Licht, *Review of anticoagulation in pediatric vortex ports.* Journal of Clinical Apheresis, 2019. **34**(2): p. 129.

1215. Di Mola, M., N. Jawa, and C. Licht, *Single center evaluation of the quality of standard calcium replacement in pediatric therapeutic plasma exchange.* Journal of Clinical Apheresis, 2019. **34**(2): p. 158-159.

1216. Di Mola, M., et al., *Ldl apheresis in the treatment of recurrent focal segmental glomerular sclerosis in a pediatric kidney transplant patient: An initial canadian experience.* Journal of Clinical Apheresis, 2020. **35**(6): p. 562.

1217. Dinh, T.A., J. Friedman, and S. Higuera, *Plastic surgery management in pediatric meningococcal-induced purpura fulminans.* Clinics in Plastic Surgery, 2005. **32**(1): p. 117-121.

1218. Duman, M., et al., *COVID-19 disease in children presenting to the pediatric emergency department: A multicenter study with 8886 cases from Turkey.* American Journal of Emergency Medicine, 2022. **59**: p. 133-140.

1219. Duzova, A. and A. Bakkaloglu, *Central nervous system involvement in pediatric rheumatic diseases: Current concepts in treatment.* Current Pharmaceutical Design, 2008. **14**(13): p. 1295-1301.

1220. Dyer, M., et al., *Simultaneous extracorporeal membrane oxygenation and therapeutic plasma exchange procedures are safe and effective in both pediatric and adult patients.* Transfusion, 2012. **52**(SUPPL. 3): p. 21A-22A.

1221. Elbers, J., et al., *Vascular Imaging Outcomes of Childhood Primary Angiitis of the Central Nervous System.* Pediatric Neurology, 2016. **63**: p. 53-59.

1222. Elsheemy, M.S., et al., *Surgical complications and graft function following live-donor extraperitoneal renal transplantation in children 20 kg or less.* Journal of pediatric urology, 2014. **10**(4): p. 737.

1223. Emeksiz, S., et al., *Therapeutic plasma exchange: A potential management strategy for critically ill MIS-C patients in the pediatric intensive care unit.* Transfusion and Apheresis Science, 2021. **60**(3): p. 103119.

1224. Emirova, K., et al., *Renal recovery after long-term dialysis in a paediatric patient with atypical haemolytic uraemic syndrome treated with eculizumab.* Pediatric Nephrology, 2014. **29**(9): p. 1757.

1225. Emmerich, F., et al., *Successful desensitization of children with high levels of antihuman leukocyte antigen antibodies bridged to transplantation with the berlin heart excor pediatric ventricular assist device.* HLA, 2018. **92**(Supplement 1): p. 24-25.

1226. Even-Or, E., et al., *Comparison of two apheresis systems for autologous stem cell collections in pediatric oncology patients.* Transfusion, 2017. **57**(1): p. 122-130.

1227. Figueiredo, R., et al., *Granulomatosis with Polyangiitis in Adolescence: Two Distinct Presentations.* Case Rep Rheumatol, 2021. **2021**: p. 6642910.

1228. Fortenberry, J.D., *Pediatric Critical Care Management of Septic Shock Prior to Acute Kidney Injury and Renal Replacement Therapy.* Seminars in Nephrology, 2008. **28**(5): p. 447-456.

1229. Fortuny, L.R., et al., *Plasma exchange in children with severe sepsis and TAMOF.* Journal of Clinical Apheresis, 2015. **30**(2): p. 100-101.

1230. Fraga-Rodriguez, G.M., et al., *Eculizumab in a child with atypical haemolytic uraemic syndrome and haemophagocytic lymphohistiocytosis triggered by cytomegalovirus infection.* BMJ Case Rep, 2017. **2017**.

1231. Freilinger, M., et al., *Plasmapheresis in pediatric neurology: Indications, methods and outcome.* European Journal of Paediatric Neurology, 2011. **15**(SUPPL. 1): p. S18.

1232. Galacki, D.M., *An overview of therapeutic apheresis in pediatrics.* J Clin Apher, 1997. **12**(1): p. 1-3.

1233. Gander, R., et al., *Pediatric kidney retransplantation focused on surgical outcomes.* Journal of Pediatric Urology, 2022. **18**(6): p. 847.e1-847.e9.

1234. Gayretli Aydin, Z.G., et al., *The first pediatric case of hemophagocytic lymphohistiocytosis secondary to Crimean-Congo haemorrhagic fever successfully treated with therapeutic plasma exchange accompanying ribavirin and intravenous immunoglobulin.* Journal of Clinical Apheresis, 2021. **36**(5): p. 780-784.

1235. Geile, K. and A. Beck, *A single center's experience of maintaining the circuits: Therapeutic plasma exchange simultaneous with continuous renal replacement therapy and/ or extracoporeal membrane oxygenation.* Journal of Clinical Apheresis, 2015. **30**(2): p. 122-123.

1236. Genceli, M., et al., *Clinical and Laboratory Evaluations of Patients Diagnosed as Having Multisystem Inflammatory Syndrome Associated with Coronavirus Disease 2019 in Children: A Single Center Experience from Konya.* Journal of Pediatric Infectious Diseases, 2023. **18**(1): p. 17-24.

1237. Gittins, N., et al., *Cerebral vasculitis in a teenager with Goodpasture's syndrome.* Nephrology Dialysis Transplantation, 2004. **19**(12): p. 3168-3171.

1238. Gokce, M., et al., *Hematological features of pediatric systemic lupus erythematosus: suggesting management strategies in children.* Lupus, 2012. **21**(8): p. 878-84.

1239. Gokcebay, D.G., et al., *EVALUATION OF APPROPRIATE USE OF PEDIATRIC FRESH FROZEN PLASMA IN A TERTIARY CARE HOSPITAL.* Hematology, Transfusion and Cell Therapy, 2021. **43**(Supplement 3): p. S28.

1240. Gordjani, N., et al., *Hemolytic uremic syndromes in childhood.* Semin Thromb Hemost, 1997. **23**(3): p. 281-93.

1241. Gorlin, J.B., *Therapeutic plasma exchange and cytapheresis in pediatric patients.* Transfus Sci, 1999. **21**(1): p. 21-39.

1242. Grisham, J.M., A.H. Tran, and K. Ellery, *Hypertriglyceridemia-induced acute pancreatitis in children: A mini-review.* Frontiers in Pediatrics, 2022. **10**: p. 931336.

1243. Guzman, M., et al., *Libman sacks endocarditis in pediatric systemic lupus erythematosus: Clinical features and complications.* Arthritis and Rheumatology, 2018. **70**(Supplement 9): p. 505-506.

1244. Haberal, M., et al., *Pediatric liver transplant: Results of a single center.* Experimental and Clinical Transplantation, 2008. **6**(1): p. 7-13.

1245. Haller, W., et al., *Successful isolated liver transplantation in a child with atypical hemolytic uremic syndrome and a mutation in complement factor H: Brief communication.* American Journal of Transplantation, 2010. **10**(9): p. 2142-2147.

1246. Hoeg, J.M., *Pharmacologic and surgical treatment of dyslipidemic children and adolescents.* Ann N Y Acad Sci, 1991. **623**: p. 275-84.

1247. Hoppe, B., et al., *Simultaneous determination of oxalate, citrate and sulfate in children's plasma with ion chromatography.* Kidney Int, 1998. **53**(5): p. 1348-52.

1248. Irum, S., et al., *A Rare Case of Propionic Acidemia in a Six Months Female Child.* J Coll Physicians Surg Pak, 2022. **32**(8): p. S180-s182.

1249. Islabao, A.G., et al., *Managing Antiphospholipid Syndrome in Children and Adolescents: Current and Future Prospects.* Pediatric Drugs, 2022. **24**(1): p. 13-27.

1250. Ito, S., et al., *Nationwide survey of continuous renal replacement therapy for childhood acute kidney injury in Japan.* Pediatric Nephrology, 2013. **28**(8): p. 1354.

1251. Ito, S., M. Sako, and T. Igarashi, *Survey of deceased children with shiga toxin-associated hemolytic uremic syndrome in japan.* Journal of the American Society of Nephrology, 2017. **28**: p. 1090.

1252. Jalanko, H., et al., *Successful liver-kidney transplantation in two children with aHUS caused by a mutation in complement factor H.* Am J Transplant, 2008. **8**(1): p. 216-21.

1253. Jardim, H.M., et al., *Crescentic glomerulonephritis in children.* Pediatr Nephrol, 1992. **6**(3): p. 231-5.

1254. Javouhey, E., *Plasmapheresis in PICU.* European Journal of Pediatrics, 2016. **175**(11): p. 1427.

1255. Jenks, C.L., L. Raman, and H.J. Dalton, *Pediatric Extracorporeal Membrane Oxygenation.* Critical Care Clinics, 2017. **33**(4): p. 825-841.

1256. Jorgensen, M.H., et al., *Preliminary experience with high volume plasmapheresis inpediatric acute liver failure.* Nephrology Dialysis Transplantation, 2018. **33**(Supplement 1): p. i503-i504.

1257. Josephson, C.D., et al., *Safety and tolerability of solvent/detergent-treated plasma for pediatric patients requiring therapeutic plasma exchange: An open-label, multicenter, postmarketing study.* Transfusion, 2022. **62**(2): p. 396-405.

1258. Joshi, S., et al., *Triple tandem organ support procedures in critically ill children.* Journal of Clinical Apheresis, 2016. **31**(2): p. 117-118.

1259. Kara, T., M. Stack, and J. Ronaldson, *Epidurals in pediatric renal transplantation.* Pediatric Transplantation, 2015. **19**(SUPPL. 1): p. 140-141.

1260. Katlan, B., et al., *Application of therapeutic plasma exchange on extracorporeal membrane oxygenation circuit: Single pediatric center experience.* Perfusion, 2021. **36**(1 SUPPL): p. 60.

1261. Kawasaki, Y., *The pathogenesis and treatment of pediatric Henoch-Schonlein purpura nephritis.* Clinical and Experimental Nephrology, 2011. **15**(5): p. 648-657.

1262. Kazeem, O. and V. Kalra, *COAGULATION GONE AWRY.* Archives of Disease in Childhood, 2022. **107**(Supplement 2): p. A420-A421.

1263. Keles, E., et al., *Successful application of CytoSorb hemadsorption in an immunocompromised teenager with collapsing glomerulopathy, acute respiratory distress syndrome, and sepsis.* International Journal of Artificial Organs, 2019. **42**(12): p. 765-769.

1264. Kennedy, N.J. and A.W. Duncan, *Acute meningococcaemia: Recent advances in management (with particular reference to children).* Anaesthesia and Intensive Care, 1996. **24**(2): p. 197-216.

1265. Kim, Y.A. and S.R. Sloan, *Pediatric therapeutic apheresis. rationale and indications for plasmapheresis, cytapheresis, extracorporeal photopheresis, and ldl apheresis.* Pediatric Clinics of North America, 2013. **60**(6): p. 1569-1580.

1266. Komvilaisak, P., et al., *Lupus Anticoagulant-hypoprothrombinemia Syndrome (LAC-HPS) in Children With Systemic Lupus Erythematosus: Report of 3 Cases.* J Pediatr Hematol Oncol, 2017. **39**(8): p. e521-e524.

1267. Kosiak, M., et al., *Is chest sonography a breakthrough in diagnosis of pulmonary thromboembolism in children?* Pediatric Pulmonology, 2008. **43**(12): p. 1183-1187.

1268. Kropshofer, G., et al., *Plasmapheresis as treatment for transient latrogenic severe hyperlipidemia in a child with leukemia [1].* Medical and Pediatric Oncology, 2003. **41**(2): p. 177.

1269. Kwon, T., et al., *Immunoadsorption in pediatric patients.* Pediatric Nephrology, 2012. **27**(9): p. 1804-1805.

1270. Långström, S., et al., *Exchange transfusion activates coagulation and alters the coagulation profile in newborn infants.* Thromb Haemost, 2006. **96**(2): p. 142-8.

1271. Lasky, L.C., et al., *Collection and use of peripheral blood stem cells in very small children.* Bone Marrow Transplant, 1991. **7**(4): p. 281-4.

1272. Lazarev, V.N. and A.S. Skriabin, *[Natural humoral and cellular factors of nonspecific defense in the treatment of chronic sinusitis in children].* Vestn Otorinolaringol, 1998(1): p. 39-40.

1273. Leclerc, F., et al., *Specificities of DIC in neonates and children.* Reanimation, 2002. **11**(8): p. 656-666.

1274. Leshen, E., J. Anderson, and E. Nocera, *PULMONARY HEMORRHAGE DUE TO GRANULOMATOSIS WITH POLYANGIITIS IN A PEDIATRIC PATIENT.* Critical Care Medicine, 2023. **51**(1 Supplement): p. 341.

1275. Li, S., X.M. Sun, and Z. Wang, *Observation of short-term catheter induced thrombosis in children treated with blood purification.* Hippokratia, 2014. **18**(3): p. 245-250.

1276. Lu, D.F., et al., *Clinical features and outcomes of 98 children and adults with dense deposit disease.* Pediatric Nephrology, 2012. **27**(5): p. 773-781.

1277. Luban, N.L., *Review of neonatal red cell transfusion practices.* Blood Rev, 1994. **8**(3): p. 148-53.

1278. MacDermott, E.J. and T.J.A. Lehman, *Antiphospholipid syndrome in children: Review of pathogenesis, diagnosis and treatment.* Future Rheumatology, 2007. **2**(2): p. 203-211.

1279. Machovec, K.A., et al., *Cardiopulmonary Bypass Strategy for a Cyanotic Child With Hemoglobin SC Disease.* Ann Thorac Surg, 2016. **101**(6): p. 2373-5.

1280. Mandal, S. and S. Deora, *Semiselective LDL apheresis (evaflux 5a) and plasma exchanges for the management of a pediatric patient with signs of familial hypercholesterolemia and first in country (India) attempts of vortex port and cobe spectra for this purpose.* Journal of Clinical Apheresis, 2018. **33**(2): p. 189-190.

1281. Marchesi, A., et al., *Kawasaki disease: Guidelines of Italian Society of Pediatrics, part II - Treatment of resistant forms and cardiovascular complications, follow-up, lifestyle and prevention of cardiovascular risks.* Italian Journal of Pediatrics, 2018. **44**(1): p. 103.

1282. Maxted, A.P., R. Connell, and F. Hussain, *Double filtration plasmapheresis - 10-year pediatric experience as an alternative to plasma exchange.* Transfusion and Apheresis Science, 2020. **59**(6): p. 102928.

1283. McMichael, A., et al., *Prospective randomized pilot study comparing bivalirudin versus heparin in neonatal and pediatric extracorporeal membrane oxygenation.* Perfusion (germany), 2019. **34**(1): p. 246.

1284. Menezes, E.V., et al., *Reducing stillbirths: Prevention and management of medical disorders and infections during pregnancy.* BMC Pregnancy and Childbirth, 2009. **9**(SUPPL. 1): p. S4.

1285. Michon, B., et al., *Complications of apheresis in children.* Transfusion, 2007. **47**(10): p. 1837-42.

1286. Misanovic, V., et al., *Plasmapheresis in Pediatric Intensive Care Unit.* Med Arch, 2016. **70**(5): p. 332-335.

1287. Mola, M.D., et al., *First successful use of the vortex port for plasma exchange in a small child with hyperlipidemia.* Journal of Clinical Apheresis, 2011. **26**(2): p. 89.

1288. Monagle, P., *Thrombosis in Pediatric Cardiac Patients.* Seminars in Thrombosis and Hemostasis, 2003. **29**(6): p. 547-555.

1289. Nash, M.C., et al., *Anti-neutrophil cytoplasmic antibody-associated glomerulonephritis in children.* Pediatr Nephrol, 1993. **7**(1): p. 11-4.

1290. Negrier, C., et al., *Combined factor IX and protein C deficiency in a child: thrombogenic effects of two factor IX concentrates.* Am J Hematol, 1995. **48**(2): p. 120-4.

1291. Nellis, M.E., H. Dalton, and O. Karam, *Quantifiable bleeding in children supported by extracorporeal membrane oxygenation and outcome.* Critical Care Medicine, 2019. **47**(11): p. E886-E892.

1292. Nemeth, A., et al., *Homozygous familial hypercholesterolemia diagnosed in childhood in Hungary.* Cardiology in the Young, 2010. **20**(SUPPL. 1): p. 221.

1293. Nevard, C.H., et al., *Activation of coagulation and fibrinolysis in childhood diarrhoea-associated haemolytic uraemic syndrome.* Thromb Haemost, 1997. **78**(6): p. 1450-5.

1294. Nguyen, T., et al., *Microvascular thrombosis in pediatric multiple organ failure: Is it a therapeutic target?* Pediatr Crit Care Med, 2001. **2**(3): p. 187-196.

1295. O'Brien, N., *Case study: Therapeutic plasma exchange (TPE) on a paediatric patient using an extracorporeal membrane oxygenation (ECMO) circuit.* Transfusion Medicine, 2017. **27**(Supplement 2): p. 58-59.

1296. Odaman Al, I., et al., *Assessment of clinical characteristics and treatment outcomes of pediatric patients with intracardiac thrombosis: a single-center experience.* Blood Coagul Fibrinolysis, 2022. **33**(1): p. 34-41.

1297. O'Mahony, J., M. Shroff, and B. Banwell, *Mimics and Rare Presentations of Pediatric Demyelination.* Neuroimaging Clinics of North America, 2013. **23**(2): p. 321-336.

1298. Oulego-Erroz, I., et al., *Pediatric Catastrophic Antiphospholipid Syndrome Successfully Treated with Eculizumab.* Am J Respir Crit Care Med, 2021. **203**(5): p. 640-642.

1299. Pandey, P., et al., *Therapeutic Plasma Exchange (TPE) for Liver Transplant Associated-Thrombotic Microangiopathy (TA-TMA) in a 5 Month Old Infant.* Indian Journal of Hematology and Blood Transfusion, 2020. **36**(4): p. 760-762.

1300. Parco, S., et al., *Granulocyte transfusion in leukopenic children by simplified leukapheresis of related donors.* Int J Artif Organs, 1998. **21**(1): p. 63-4.

1301. Pekkucuksen, N.T., et al., *Tandem plasmapheresis on continuous renal replacement therapy in pediatrics.* Journal of the American Society of Nephrology, 2019. **30**: p. 321.

1302. Perk, O., et al., *Crimean-Congo hemorrhagic fever: A pediatric case responding to plasmapheresis treatment.* Transfusion and Apheresis Science, 2021. **60**(6): p. 103215.

1303. Pishko, A.M. and B.S. Doshi, *Acquired Hemophilia A: Current Guidance and Experience from Clinical Practice.* Journal of Blood Medicine, 2022. **13**: p. 255-265.

1304. Pongphitcha, P., et al., *Report on effective treatment and genetic predisposition in two children with refractory probable catastrophic antiphospholipid syndrome.* Thrombosis Research, 2021. **208**: p. 117-120.

1305. Pongphitcha, P., et al., *Successfully treated pediatric catastrophic antiphospholipid syndrome with combination of thrombolytic agent, rituximab and sirolimus.* Research and Practice in Thrombosis and Haemostasis, 2021. **5**(SUPPL 2).

1306. Ponikvar, R., et al., *Hyperbaric oxygenation, plasma exchange, and hemodialysis for treatment of acute liver failure in a 3-year-old child.* Artif Organs, 1998. **22**(11): p. 952-7.

1307. Ponikvar, R., et al., *Continuous renal replacement therapy and plasma exchange in newborns and infants.* Artif Organs, 2002. **26**(2): p. 163-8.

1308. Pottel, H., et al., *51Cr-EDTA plasma clearance in children: One, two, or multiple samples?* Medicine (Baltimore), 2022. **101**(3): p. e28608.

1309. Prasun Giri, P., et al., *Therapeutic plasma exchange in paediatric SLE: a case series from India.* Lupus, 2015. **24**(8): p. 889-91.

1310. Preston, T.J., et al., *Plasma exchange on venovenous extracorporeal membrane oxygenation with bivalirudin anticoagulation.* World Journal for Pediatric and Congenital Heart Surgery, 2014. **6**(1): p. 119-122.

1311. Preston, T.J., et al., *Plasma exchange on venovenous extracorporeal membrane oxygenation with bivalirudin anticoagulation.* World J Pediatr Congenit Heart Surg, 2015. **6**(1): p. 119-22.

1312. Proesmans, W., *Hemolytic uremic syndrome in children.* Tijdschrift voor Geneeskunde, 2000. **56**(5): p. 370-377.

1313. Rabik, C.A., et al., *Treatment of an acquired Factor XIII inhibitor in an adolescent with systemic lupus erythematosus and renal failure.* Transfusion, 2017. **57**(9): p. 2159-2163.

1314. Rajendran, R., et al., *Late effects of childhood cancer treatment: Severe hypertriglyceridaemia, central obesity, non alcoholic fatty liver disease and diabetes as complications of childhood total body irradiation.* Diabetic Medicine, 2013. **30**(8): p. e239-e242.

1315. Ravelli, A. and A. Martini, *Antiphospholipid Syndrome in Pediatrics.* Rheumatic Disease Clinics of North America, 2007. **33**(3): p. 499-523.

1316. Renaud, C., et al., *Haemolytic uraemic syndrome: Prognostic factors in children over 3 years of age.* Pediatric Nephrology, 1995. **9**(1): p. 24-29.

1317. Rigante, D., et al., *Anti-phospholipid syndrome: Clinical spectrum and therapeutical/prophylactic strategies in the pediatric population.* European Review for Medical and Pharmacological Sciences, 2008. **12**(1): p. 47-53.

1318. Rubik, J., et al., *[Clinical aspects of plasmapheresis therapy in children: single center experience].* Pol Merkur Lekarski, 2003. **14**(82): p. 304-10.

1319. Sabia, J., et al., *Effective therapeutic anticoagulation, reduced blood product use and lower financial cost following implementation of anti-Xa based anticoagulation protocol for neonatal respiratory ECMO support.* ASAIO Journal, 2020. **66**(SUPPL 3): p. 45.

1320. Sagliocchi, A., et al., *Total plasma exchange (TPE) and citrate crrt in a low-weight pediatric patient with macrophage activation syndrome (MAS): Feasibility and complexities of both treatments.* Nephrology Dialysis Transplantation, 2020. **35**(SUPPL 3): p. iii1448.

1321. Saji, T., *Guidelines for medical treatment of acute Kawasaki disease: Report of the Research Committee of the Japanese Society of Pediatric Cardiology and Cardiac Surgery (2012 revised version).* Pediatrics International, 2014. **56**(2): p. 135-158.

1322. Schaefer, B., et al., *Combined hemodialysis and plasma exchange versus sequential treatment in children.* Pediatric Nephrology, 2011. **26**(9): p. 1622-1623.

1323. Schaefer, B., et al., *Bleeding complications in pediatric ABO-incompatible kidney transplantation.* Pediatric Nephrology, 2013. **28**(2): p. 327-332.

1324. Schaefer, B., et al., *Safety and efficacy of tandem hemodialysis and plasma exchange in children.* Clin J Am Soc Nephrol, 2014. **9**(9): p. 1563-70.

1325. Schmidt, B., et al., *A placebo-controlled randomized trial of antithrombin therapy in neonatal respiratory distress syndrome.* Am J Respir Crit Care Med, 1998. **158**(2): p. 470-6.

1326. Shah, S., C. Joseph, and P. Srivaths, *Role of therapeutic apheresis in the treatment of pediatric kidney diseases.* Pediatric Nephrology, 2022. **37**(2): p. 315-328.

1327. Shen, X., R. Wile, and G. Young, *FondaKIDS III: A long-term retrospective cohort study of fondaparinux for treatment of venous thromboembolism in children.* Pediatric Blood and Cancer, 2020. **67**(8): p. e28295.

1328. Shimizu, A., et al., *A large superior mesenteric artery aneurysm and ileal obstruction: A rare presentation of polyarteritis nodosa in an infant.* Oxford Medical Case Reports, 2019. **2019**(9): p. 401-404.

1329. Sık, G., et al., *Therapeutic plasma exchange in pediatric intensive care: Indications, results and complications.* Ther Apher Dial, 2020. **24**(2): p. 221-229.

1330. Silverman, R.B. and S.M. Quinn, *Successful ICU Care of a 14-Year-Old With Total Artificial Circulation Using Two Ventricular Assist Devices for 118 Days.* ICU Director, 2012. **3**(5): p. 240-246.

1331. Sirignano, R.M., et al., *Pediatric tandem therapeutic apheresis a multidisciplinary approach.* ASAIO Journal, 2018. **64**(3): p. 382-388.

1332. Sirignano, R.M., et al., *Epidemiology of therapeutic apheresis with a multidisciplinary approach at a high volume pediatric center.* Journal of Clinical Apheresis, 2018. **33**(3): p. 297-302.

1333. Sonmez, H.E., et al., *The Multifaceted Presentation of the Multisystem Inflammatory Syndrome in Children: Data from a Cluster Analysis.* Journal of Clinical Medicine, 2022. **11**(6): p. 1742.

1334. Souid, A.K. and P.D. Sadowitz, *Acute childhood immune thrombocytopenic purpura: Diagnosis and treatment.* Clinical Pediatrics, 1995. **34**(9): p. 487-494.

1335. Soundar, E., et al., *Citrate toxicity in pediatric liver failure patients on therapeutic plasma exchange.* Laboratory Investigation, 2015. **95**(SUPPL. 1): p. 424A.

1336. Soybilgic, A. and T. Avcin, *Pediatric APS: State of the Art.* Current Rheumatology Reports, 2020. **22**(3): p. 9.

1337. Sozeri, B., et al., *The clinical course and short-term health outcomes of multisystem inflammatory syndrome in children in the single pediatric rheumatology center.* Postgraduate Medicine, 2021. **133**(8): p. 994-1000.

1338. Srivaths, P.R., et al., *Therapeutic plasma exchange (TPE) in tandem with cardiopulmonary bypass (CPB) during heart transplantexperience from a pediatric program.* Journal of Clinical Apheresis, 2015. **30**(2): p. 116-117.

1339. Stefanutti, C., S. Di Giacomo, and C. Morozzi, *Therapeutic apheresis in very low weight patients including newborns and children.* Therapeutic Apheresis and Dialysis, 2018. **22**(4): p. 419-420.

1340. Stepanova, N.A., et al., *[Infusion-transfusion therapy of acute blood loss during early surgeries in children with severe thermal trauma].* Anesteziol Reanimatol, 2004(2): p. 32-6.

1341. Stevens, P., et al., *VA-ECMO as a Bridge to Liver Re-transplantation in an Infant with Primary Graft Nonfunction.* ASAIO Journal, 2022. **68**(SUPPL 1): p. 25.

1342. Sun, X., et al., *Extracorporeal treatment in children with acute severe poisoning.* Medicine (United States), 2019. **98**(47): p. e18086.

1343. Tanyildiz, M., et al., *ECMO experiences of Ihsan Dogramaci children's hospital, Ankara, Turkey.* European Journal of Heart Failure, 2017. **19**(Supplement 2): p. 55-56.

1344. Taylan, C., et al., *Safety of Therapeutic Apheresis in Children and Adolescents.* Frontiers in Pediatrics, 2022. **10**: p. 850819.

1345. Thomson, J.J., A. Retter, and B.J. Hunt, *Novel management of post varicella purpura fulminans owing to severe acquired protein S deficiency.* Blood Coagul Fibrinolysis, 2010. **21**(6): p. 598-600.

1346. Tran, C.L., et al., *Parallel centrifugation plasma-exchange and continuous venovenous hemodiafiltration.* Pediatric Nephrology, 2015. **30**(12): p. 2241.

1347. Tsujii, N., et al., *Influenza-associated thrombotic microangiopathy with unbalanced von Willebrand factor and a disintegrin and metalloproteinase with a thrombospondin type 1 motif, member 13 levels in a heterozygous protein S-deficient boy.* Pediatr Int, 2016. **58**(9): p. 926-9.

1348. Twilt, M., et al., *Treatment and outcome of ANCA-associated vasculitis in children: A pilot study.* Arthritis and Rheumatism, 2012. **64**(SUPPL. 10): p. S124.

1349. Ulbrich, A., et al., *Plasmapheresis and Cyclophosphamide therapy in an eight year old girl with cerebral manifestation of systemic lupus erythematodes.* Pediatric Rheumatology, 2011. **9**(SUPPL. 1).

1350. Unuvar, A., et al., *Thrombocytopenia associated multiple organ failure in an infant - A challenging case.* Journal of Thrombosis and Haemostasis, 2009. **7**(S2): p. 467.

1351. Uyar, E., et al., *Can therapeutic plasma exchange be life-saving in life-threatening manganese intoxication?* Transfusion and Apheresis Science, 2022. **61**(4): p. 103417.

1352. Valaiyapathi, B. and A.P. Ashraf, *Hospital management of severe hypertriglyceridemia in children.* Current Pediatric Reviews, 2017. **13**(4): p. 225-231.

1353. van Vuurden, D.G., et al., *Therapeutic total plasma exchange in a child with neuroblastoma-related anti-Hu syndrome.* Pediatric Nephrology, 2005. **20**(11): p. 1655-1656.

1354. Vargha, R., et al., *Treatment with N-acetylcystein and total plasma exchange for extracorporeal liver support in children with paracetamol intoxication.* Klinische Padiatrie, 2014. **226**(2): p. 84-85.

1355. Vidal, E., et al., *Therapeutic Plasma Exchange in Neonates and Infants: Successful Use of a Miniaturized Machine.* Blood Purification, 2017. **44**(2): p. 100-105.

1356. Von Scheven, E., et al., *Thrombosis and Pediatric Wegener's Granulomatosis: Acquired and Genetic Risk Factors for Hypercoagulability.* Arthritis Care and Research, 2003. **49**(6): p. 862-865.

1357. Wagner, A.F., et al., *Pulmonary hemorrhage and liver failure: Does either preclude extracorporeal membrane oxygenaton candidacy?* ASAIO Journal, 2020. **66**(Supplement 1): p. 6.

1358. Webb, J., et al., *Modifications to terumo optia therapeutic plasma exchange to achieve rapid exchange on cardiopulmonary bypass prior to pediatric cardiac transplant.* Journal of Clinical Apheresis, 2019. **34**(2): p. 129-130.

1359. Webb, T.N., et al., *Retrospective analysis comparing complication rates of centrifuge vs membrane-based therapeutic plasma exchange in the pediatric population.* J Clin Apher, 2022. **37**(3): p. 263-272.

1360. Weller, J., et al., *Successful plasma exchange and surgical procedures on a pediatric patient anticoagulated with bivalirudin on extracorporeal membrane oxygenation.* American Journal of Respiratory and Critical Care Medicine, 2019. **199**(9).

1361. West, F.B., et al., *Magnesium supplementation during pediatric apheresis procedures.* Journal of Clinical Apheresis, 2011. **26**(2): p. 63-64.

1362. Willis, R., et al., *Plasmapheresis for pediatric abo incompatible liver transplant.* Journal of Clinical Apheresis, 2014. **29**(1): p. 39-40.

1363. Witt, V., et al., *Tandem procedure (TPE plus CVVHDF) in children, how to manage anticoagulation?* Journal of Clinical Apheresis, 2014. **29**(1): p. 43.

1364. Witt, V., et al., *World apheresis registry data from 2003 to 2007, the pediatric and adolescent side of the registry.* Transfus Apher Sci, 2008. **39**(3): p. 255-60.

1365. Wright, E.C., K. Tullus, and M.J. Dillon, *Retrospective study of plasma exchange in children with systematic lupus erythematosus.* Pediatric Nephrology, 2004. **19**(10): p. 1108-1114.

1366. Yang, X., et al., *Individualized medication based on pharmacogenomics and treatment progress in children with IgAV nephritis.* Frontiers in Pharmacology, 2022. **13**: p. 956397.

1367. Yoshikawa, N., K. Iijima, and H. Ito, *IgA nephropathy in children.* Nephron, 1999. **83**(1): p. 1-12.

1368. Yoshimoto, M., et al., *Effect of continuous LDL apheresis with dextran-sulfate cellulose column system on a child with homozygous familial hypercholesterolemia.* Acta Paediatr Jpn, 1990. **32**(2): p. 146-50.

1369. Zawitkowska, J., et al., *Severe drug-induced hypertriglyceridemia treated with plasmapheresis in children with acute lymphoblastic leukemia.* Transfusion and Apheresis Science, 2019. **58**(5): p. 634-637.

1370. Zheng, X., et al., *Recurrent ocular involvement in pediatric atypical hemolytic uremic syndrome.* J Pediatr Ophthalmol Strabismus, 2014. **51 Online**: p. e62-5.

1371. Akcan-Arikan, A., et al., *Safetyand efficacy of regional citrate anticoagulation in pediatric liver failure.* Pediatric Nephrology, 2015. **30**(12): p. 2241.

1372. Akcay, N., et al., *Therapeutic plasma exchange in pediatric patients with acute demyelinating syndromes of the central nervous system: A single-center experience.* Transfusion and Apheresis Science, 2022. **61**(4): p. 103421.

1373. Aplenc, L.M., C. George, and K. Mariot, *Therapeutic plasma exchange (TPE) performed in tandem with CRRT and/or ECMO: Experience of one pediatric center.* Journal of Clinical Apheresis, 2018. **33**(2): p. 159-160.

1374. Aplenc, L.M., et al., *Efficiency and safety of the therapeutic plasma exchange (TPE) performed in tandem with crrt and/or ecmo, experience of one pediatric center.* Journal of Clinical Apheresis, 2021. **36**(2): p. 241.

1375. Arena, R., et al., *Management of anticoagulation during therapeutic plasma exchange in tandem with extracorporeal membrane oxygenation (ECMO) in critically ill children.* Perfusion, 2020. **35**(1 SUPPL): p. 249-250.

1376. Badran, O., et al., *Improved clinical outcomes associated with anti-Xa based anticoagulation protocol for neonatal respiratory ECMO support.* ASAIO Journal, 2020. **66**(SUPPL 3): p. 43.

1377. Bungardi, A.M., et al., *THERAPEUTIC PLASMA EXCHANGE IN A PEDIATRIC NEPHROLOGY UNIT.* Pediatric Nephrology, 2022. **37**(11): p. 2916.

1378. Chomat, M., et al., *Bivalirudin dosing during plasma exchange in pediatric ECMO.* ASAIO Journal, 2022. **68**(SUPPL 1): p. 34.

1379. Cortina, G., et al., *Therapeutic plasma exchange in children: One center's experience.* J Clin Apher, 2017. **32**(6): p. 494-500.

1380. Cui, Y., et al., *Sequential Blood Purification for Pediatric Fatal Toxic Epidermal Necrolysis: A Case Series.* Blood Purification, 2022. **51**(7): p. 600-607.

1381. Daverio, M., et al., *Continuous Kidney Replacement Therapy Practices in Pediatric Intensive Care Units Across Europe.* JAMA Network Open, 2022. **5**(12): p. E2246901.

1382. De Palo, T., et al., *Therapeutic apheresis in children: experience in a pediatric dialysis center.* Int J Artif Organs, 2000. **23**(12): p. 834-9.

1383. Delaney, M., et al., *A survey of pediatric apheresis practice.* Journal of Clinical Apheresis, 2012. **27**(1): p. 2.

1384. Fernandez Sarmiento, J., M.A. Varela, and C.E. Pinzon, *Frequency of hemorrhagic complications in plasmapheresis without extracorporeal circuit anticoagulation, in children.* Transfusion and Apheresis Science, 2016. **55**(1): p. 136-140.

1385. Kevy, S.V. and M. Fosburg, *Therapeutic apheresis in childhood.* J Clin Apher, 1990. **5**(2): p. 87-90.

1386. Kreuzer, M., et al., *Regional citrate anticoagulation-a safe and effective procedure in pediatric apheresis therapy.* Pediatric Nephrology, 2011. **26**(1): p. 127-132.

1387. Marshall, C.S., R. Pretzlaff, and D. Dwyre, *Tandem therapeutic plasma exchange for neonates with mechanical hemolysis on ECMO.* Journal of Clinical Apheresis, 2009. **24**(2): p. 82.

1388. Ozturk, A.G., et al., *Use of Therapeutic Plasma Exchange in the Pediatric Intensive Care Unit.* Turkish Archives of Pediatrics, 2022. **57**(2): p. 186-192.

1389. Paglialonga, F., et al., *Tandem plasmapheresis and hemodialysis in a pediatric dialysis unit.* Hemodialysis International, 2011. **15**(1): p. 160.

1390. Paglialonga, F., et al., *Plasma-exchange in pediatric patients: A single-center experience.* Minerva Pediatrica, 2017. **69**(2): p. 113-120.

1391. Paglialonga, F., et al., *Indications, technique, and outcome of therapeutic apheresis in European pediatric nephrology units.* Pediatr Nephrol, 2015. **30**(1): p. 103-11.

1392. Rodl, S., et al., *Tandem treatment of plasmapheresis and hemodialysiswith citrate anticoagulation in children.* Pediatric Nephrology, 2015. **30**(12): p. 2244-2245.

1393. Shah, S.A., *Clinical evaluation of membrane therapeutic plasma exchange using prismaflex machines and fresh frozen plasma in pediatric patients.* Journal of the American Society of Nephrology, 2021. **32**: p. 602.

1394. Sigler, K., J. Lee, and P. Srivaths, *Regional citrate anticoagulation with calcium replacement in pediatric apheresis.* J Clin Apher, 2018. **33**(3): p. 274-277.

1395. Sigler, K., J.Y. Lee, and P. Srivaths, *Calcium replacement with regional citrate anticoagulation in pediatric apheresis therapy.* Journal of Clinical Apheresis, 2016. **31**(2): p. 140.

1396. Zhang, X., et al., *Optimal stage of initiating continuous renal replacement therapy in the treatment of neonatal acute kidney injury.* Experimental and Therapeutic Medicine, 2022. **24**(6): p. 733.

1397. Abe, N. and T. Atsumi, *[Neurological Manifestations in Antiphospholipid Syndrome].* Brain Nerve, 2021. **73**(5): p. 526-536.

1398. Akimoto, T. and H. Hashimoto, *[Antiphospholipid syndrome].* Ryoikibetsu Shokogun Shirizu, 2000(31): p. 392-5.

1399. Akizawa, T., *[Beneficial characteristics of protease inhibitor as an anticoagulant for extracorporeal circulation].* Rinsho Ketsueki, 1990. **31**(6): p. 782-6.

1400. Amemiya, H., et al., *[Anticoagulation for plasmapheresis].* Nihon Rinsho, 2004. **62 Suppl 5**: p. 323-7.

1401. Amrein, K., et al., *Plasmapheresis and Osteoporosis: The absence of evidence is not the evidence of absence.* Journal fur Mineralstoffwechsel, 2016. **23**(2): p. 44-47.

1402. Anonymous, *Reduction of donor blood in surgery: A necessity in heart surgery.* Fortschritte der Medizin, 1994. **112**(16): p. I-II.

1403. Anonymous, *The treatment of hemostatic complications.* Sang Thrombose Vaisseaux, 1995. **7**(8 SUPPL.): p. 47-58.

1404. Arai, M., *[Coagulopathy with autoantibodies to blood-clotting factors: overview and laboratory diagnosis].* Rinsho Byori, 2004. **52**(2): p. 178-81.

1405. Bolz, M. and K. Friese, *Concepts on drug therapy for pregnancy-induced hypertension and HELLP syndrome.* Gynakologe, 1998. **31**(11): p. 934-941.

1406. Canova, C.R., et al., *'White clot syndrome'.* Schweizerische Medizinische Wochenschrift, 1997. **127**(18): p. 762-765.

1407. Chen, Y., et al., *[Real time monitoring of heparin anticoagulant therapy in severe hepatitis patients treated with plasma exchange].* Zhonghua Gan Zang Bing Za Zhi, 2005. **13**(6): p. 465-6.

1408. Cong, H., et al., *[Study on the selective removal of plasma low-density lipoprotein and fibrinogen by degraded carrageenan].* Sheng Wu Yi Xue Gong Cheng Xue Za Zhi, 2010. **27**(4): p. 829-33, 846.

1409. De Wit, M., H.J. Weh, and D.K. Hossfeld, *Thrombotic thrombocytopenic purpura and hemolytic uremic syndrome.* Medizinische Welt, 1996. **47**(12): p. 528-532.

1410. Ding, M., *[A prospective trial of hemodilution therapy in acute cerebral infarction].* Zhonghua Shen Jing Jing Shen Ke Za Zhi, 1992. **25**(3): p. 146-9, 190.

1411. Dong, R., et al., *[The 455th case: swollen leg, jaundice and mental disturbance].* Zhonghua Nei Ke Za Zhi, 2017. **56**(4): p. 316-320.

1412. Eremeeva, D.R., et al., *Use of plasmapheresis in treatment of patients with unfavorable anamnestic pregnancy outcomes coupled to antiphospholipid antibodies.* Obstetrics, Gynecology and Reproduction, 2021. **15**(1): p. 22-31.

1413. Freund, M., *Hematologic disorders in pregnancy.* Gynakologe, 2004. **37**(5): p. 392-400.

1414. Funauchi, M. and T. Yamagata, *[Systemic lupus erythematosus].* Nihon Rinsho, 2004. **62 Suppl 5**: p. 559-62.

1415. Gu, J.Y., et al., *[Case series and clinical analysis of 14 cases of catastrophic antiphospholipid syndrome].* Beijing Da Xue Xue Bao Yi Xue Ban, 2018. **50**(6): p. 1033-1038.

1416. Han, H.L., et al., *[Clinical Analysis of ABO-Incompatible Living-Donor Liver Transplantation in Children].* Sichuan Da Xue Xue Bao Yi Xue Ban, 2022. **53**(5): p. 777-781.

1417. Harten, P., *Neuropsychiatric systemic lupus erythematosus.* Aktuelle Rheumatologie, 1996. **21**(2): p. 77-88.

1418. Hartung, H.D., *The prevention of rebound effect following LDL apheresis.* Fortschritte der Medizin, 1991. **109**(24): p. 59.

1419. Hartung, H.P., et al., *Treatment of acute Guillain-Barre syndrome.* Nervenarzt, 1994. **65**(12): p. 807-818.

1420. Hashimoto, H., *[Vascular involvements in systemic lupus erythematosus].* Nihon Rinsho, 1994. **52**(8): p. 2109-13.

1421. Hashimoto, H., *[Allergic granulomatosis angiitis].* Nihon Rinsho, 2005. **63 Suppl 5**: p. 165-72.

1422. Hattori, A. and W. Tatewaki, *[Treatment of thrombotic thrombocytopenic purpura--retrospective analysis on Japanese patients and review. Japan TTP Research Group].* Nihon Rinsho, 1993. **51**(1): p. 178-83.

1423. Herdegen, T., *Coagulopathy: Blood flow between congestion and excessive velocity.* Deutsche Apotheker Zeitung, 2009. **149**(31): p. 42-79.

1424. Hiepe, F. and G.R. Burmester, *Treatment of systemic lupus erythematosus.* Deutsche Medizinische Wochenschrift, 1996. **121**(37): p. 1129-1133.

1425. Hirayama, K. and A. Koyama, *[Adverse effects of plasma exchange].* Nihon Rinsho, 2004. **62 Suppl 5**: p. 319-22.

1426. Horie, Y. and H. Ishii, *[Severe alcoholic hepatitis in Japan].* Nihon Shokakibyo Gakkai Zasshi, 2002. **99**(11): p. 1326-33.

1427. Hospach, T., et al., *Purpura Schoenlein-Henoch - Results of the Worlitz 2005 Consensus Conference focussing on diagnosis and therapy.* Klinische Padiatrie, 2008. **220**(1): p. 47-52.

1428. Hu, Y.F., et al., *Catastrophic antiphospholipid syndrome.* Journal of Internal Medicine of Taiwan, 2005. **16**(1): p. 33-41.

1429. Ichikawa, K. and T. Koike, *[Current topics in vascular disorders].* Nihon Rinsho, 1994. **52**(8): p. 2152-7.

1430. Ichikawa, K. and T. Koike, *[Coping with pathological changes in nerves and blood vessels in collagen disease--antiphospholipid syndrome and thrombosis].* Nihon Naika Gakkai Zasshi, 1996. **85**(11): p. 1828-32.

1431. Ieko, M., *[Antiphospholipid syndrome: diagnosis and management].* Rinsho Ketsueki, 2021. **62**(5): p. 445-455.

1432. Inaba, S., *[Advancement in the preparation of red cell concentrates and platelet concentrates].* Masui, 2011. **60**(1): p. 23-30.

1433. Inoue, D., et al., *[Early diagnosis and successful treatment of catastrophic antiphospholipid syndrome complicated by multiple organ failure].* Nihon Rinsho Meneki Gakkai Kaishi, 2010. **33**(1): p. 24-30.

1434. Inoue, K. and M. Yoshiba, *[Blood purification for patients with chronic renal failure accompanied by severe liver disease].* Nihon Rinsho, 2004. **62 Suppl 6**: p. 66-9.

1435. Ito, K. and Y. Komatsu, *[Drug induced hemolytic uremic syndrome].* Nihon Rinsho, 1993. **51**(1): p. 204-9.

1436. Izumi, Y., et al., *[Enterohemorrhagic Escherichia coli O157 infection in an elderly patient with secondary hemolytic uremic syndrome who developed recurrent acute exacerbation of chronic cholecystitis].* Nihon Ronen Igakkai Zasshi, 1998. **35**(7): p. 559-65.

1437. Kajita, M., et al., *[Refractory thrombotic thrombocytopenic purpura complicated with multiple cerebral infarction].* Rinsho Ketsueki, 2022. **63**(1): p. 55-61.

1438. Karasawa, G., K. Hirata, and H. Hayasaka, *[Bacterial infection].* Nihon Rinsho, 1991. **49**(6): p. 1318-22.

1439. Kashima, K., K. Kataoka, and K. Umehara, *[Treatment of acute pancreatitis].* Nihon Naika Gakkai Zasshi, 1992. **81**(12): p. 1912-7.

1440. Katsadze, I.L., S.S. Bessmel'tsev, and K.M. Abdulkadyrov, *The effect of different programs of cytostatic therapy on the blood coagulation and rheological properties in multiple myeloma patients.* Terapevticheskii arkhiv, 1991. **63**(7): p. 61-64.

1441. Keller, F., H. Scharze, and A. Schwarz, *Clinical management of hemolytic-uremic syndrome and thrombotic-thrombocytopenic purpura (HUS-TTP).* Wiener Klinische Wochenschrift, 1994. **106**(19): p. 603-607.

1442. Klingell, R., et al., *Differential indication of lipoprotein apheresis during pregnancy.* Nieren- und Hochdruckkrankheiten, 2003. **32**(5): p. 179-188.

1443. Kodama, M., T. Tani, and N. Inoue, *[Therapeutic plasmapheresis to treat postoperative hepatic failure clinical course and therapeutic outcome in Japan].* Nihon Geka Gakkai Zasshi, 1993. **94**(7): p. 707-13.

1444. Koike, M. and K. Nitta, *[Treatment of hypertriglyceridemia: plasma exchange].* Nihon Rinsho, 2013. **71**(9): p. 1667-9.

1445. Komarov, V.T., et al., *Clinical variants of Churg-Strauss eosinophilic vasculitis.* Klinicheskaia meditsina, 2007. **85**(6): p. 63-66.

1446. Konstantinow, A., et al., *Toxic epidermal necrolysis (drug-induced Lyell's syndrome). Part 2: Treatment.* Deutsche Medizinische Wochenschrift, 2001. **126**(7): p. 177-179.

1447. Kotenko, O.N., et al., *Clinical course and approaches to therapy in kidney transplant recipients with the novel covid-19 disease.* Vestnik Transplantologii i Iskusstvennykh Organov, 2020. **22**(4): p. 69-74.

1448. Kozek-Langenecker, S.A., M. Duris, and B. Rottmann, *Anticoagulation and pharmacological reversal.* Anasthesiologie und Intensivmedizin, 2007. **48**(SUPPL. 5): p. S153-S158.

1449. Kudo, T., *[Molecular dynamics of human intrafollicular plasma kallikrein and the mechanism of activation of tissue-type plasminogen activator (tPA) in ovarian follicles].* Hokkaido Igaku Zasshi, 1998. **73**(6): p. 599-611.

1450. Kuznetsova, S.A., et al., *Effects of immune complexes isolated from the plasma of patients with rheumatoid arthritis on the secretion of pro- and antiinflammatory cytokines by normal human blood cells.* Immunologiya, 2000(4): p. 48-53.

1451. Li, T., et al., *[Purification, identification of acoagulatin, an anticoagulation factor from the venom of Chinese Agkistrodon, and observation of its anticoagulation effect].* Di Yi Jun Yi Da Xue Xue Bao, 2004. **24**(7): p. 836-8.

1452. Li, W., S.D. Lin, and J. Long, *Plasma from patients with liver failure inhibits the proliferation of HepG2 cells.* World Chinese Journal of Digestology, 2012. **20**(14): p. 1204-1209.

1453. Li, X., et al., *Recent advances in simple plasma exchange therapy with regional citrate anticoagulation.* Chinese Journal of Clinical Infectious Diseases, 2021. **14**(6): p. 475-480.

1454. Limbach, H.G., G. Loffler, and U.T. Seyfert, *Thrombolytic therapy with urokinase in hemolytic-uremic syndrome.* Hamostaseologie, 1996. **16**(4): p. 256-259.

1455. Liu, Y., et al., *[Establishment of method detecting CD36 expression on human platelet and its application].* Zhongguo Shi Yan Xue Ye Xue Za Zhi, 2013. **21**(4): p. 1042-5.

1456. Lukomskii, G.I., et al., *Plasmapheresis in preoperative care of patients with thyrotoxicosis.* Khirurgiia, 1991(4): p. 102-105.

1457. Ma, Y.J., et al., World Chinese Journal of Digestology, 2018. **26**(3): p. 165-173.

1458. Matsushima, H., et al., *[Case of acute kidney failure induced low-molecular weight dextran effectively treated by plasma exchange].* Nihon Naika Gakkai Zasshi, 1994. **83**(2): p. 294-5.

1459. Matthes, G., et al., *Implementation of preparative haemaphereses in the production of blood-derived cell concentrates - Recommendations for preparative haemapheresis of the German Society of Transfusion Medicine and Immunohaematology (DGTI).* Transfusion Medicine and Hemotherapy, 2007. **34**(5): p. 367-374.

1460. Matusiak-Kita, M., M. Murawski, and M. Grybos, *Antiphospholipid syndrome in pregnancy--actual therapeutic methods.* Wiadomosci lekarskie (Warsaw, Poland : 1960), 2008. **61**(10-12): p. 273-276.

1461. Mikhailov, A.A., et al., *Intensive therapy of DIC syndrome in acute renal failure due to crush syndrome.* Klinicheskaya Meditsina, 1991. **69**(5): p. 80-84.

1462. Minamoto, K., *[Beneficial effect of a stable PGI2 analogue (ONO-1301) on prostanoid release after reperfusion in canine left single lung allotransplantation model].* Nihon Kyobu Geka Gakkai Zasshi, 1997. **45**(12): p. 1931-42.

1463. Mo, J.K., et al., *[Heparin-induced extacorporeal low-density lipoprotein-lipoprotein alpha-fibrinogen precipitation in 36 cases].* Di Yi Jun Yi Da Xue Xue Bao, 2002. **22**(3): p. 278-9.

1464. Morishima, T., *[Treatment of influenza-associated encephalopathy].* Nihon Rinsho, 2003. **61**(11): p. 2006-12.

1465. Moskaleva, E.S., et al., *Plasmapheresis in the combined therapy of progressive forms of glomerulonephritis.* Terapevticheskii arkhiv, 1994. **66**(6): p. 42-45.

1466. Nakagawa, M., Y. Maruyama, and M. Osame, *[Therapy for HAM/TSP and AIDS].* Nihon Rinsho, 1994. **52**(11): p. 3019-25.

1467. Nakagawa, Y., K. Saito, and K. Takahashi, *[ABO-incompatible kidney transplantation].* Nihon Rinsho, 2005. **63 Suppl 4**: p. 700-5.

1468. Nakashima, S., et al., *[Intravenous cyclophosphamide pulse therapy for refractory juvenile dermatomyositis].* Ryumachi, 2002. **42**(6): p. 895-902.

1469. Neimark, A.I., I.I. Astakhov, and A.V. Mazyrko, *The treatment of the disseminated intravascular coagulation syndrome in urosepsis patients.* Urologiia i nefrologiia, 1990(4): p. 13-17.

1470. Neimark, A.I., I. Astakhov Yu, and A.V. Mazyrko, *Therapy of disseminated intravascular coagulation in patients with urosepsis.* Urologiya i Nefrologiya, 1990. **55**(4): p. 13-17.

1471. Piksin, I.N., et al., *Cryoapheresis in peritonitis treatment.* Fiziologiia cheloveka, 2006. **32**(5): p. 140-142.

1472. Piper, B., *Are you familiar with the antiphospholipid antibody syndrome? Also a cause of thromboembolism.* MMW-Fortschritte der Medizin, 2000. **142**(7): p. 40-41.

1473. Ranze, O. and A. Greinacher, *Current treatment concepts for heparin-induced thrombocytopenia.* Deutsche Medizinische Wochenschrift, 1999. **124**(28-29): p. 865-873.

1474. Rath, W. and L. Heilmann, *Treatment of coagulation disorders in pregnancy.* Gynakologe, 2005. **38**(9): p. 791-798.

1475. Riess, H. and W.O. Bechstein, *Hemostasis in liver diseases.* Intensivmedizin und Notfallmedizin, 1998. **35**(SUPPL. 1): p. 109-117.

1476. Romicka, A.M., *Juvenile systemic lupus erythematosus.* Reumatologia, 2004. **42**(SUPPL. 1): p. 39-45.

1477. Rubik, J., et al., *Clinical aspects of plasmapheresis therapy in children - Single-centre experience.* Polski Merkuriusz Lekarski, 2003. **14**(82): p. 304-310.

1478. Sajdak, S., et al., *Antiphospholipid syndrome in the course of pregnancy.* Ginekologia i Poloznictwo, 2011. **20**(2): p. 52-67.

1479. Sakamaki, Y., et al., *[Renal thrombotic microangiopathy and antiphospholipid syndrome nephropathy in a patient with lupus nephritis].* Nihon Jinzo Gakkai Shi, 2016. **58**(1): p. 45-54.

1480. Salinger, R., *Therapeutic plasma exchange in the treatment of rheumatic-immunologic diseases.* Therapiewoche, 1991. **41**(13): p. 798-804.

1481. Saulko, A.M., V.A. Tatsievskii, and M.G. Pavliutenkov, *Radioimmunodiagnosis in the correction of plasmapheresis methodology in thyrotoxicosis.* Meditsinskaia radiologiia, 1991. **36**(6): p. 23-25.

1482. Scharf, R.E., *Congenital and acquired thrombocytopenias.* Hamostaseologie, 2003. **23**(4): p. 159-169.

1483. Schilling, S., et al., *Plasma exchange therapy for steroid-unresponsive multiple sclerosis relapses. Clinical experience with 16 patients.* Nervenarzt, 2006. **77**(4): p. 430-438.

1484. Schmitt, C., et al., *Natural human IgM antibodies in neuroblastoma therapy: Preliminary results from a phase I/II therapy study.* Klinische Padiatrie, 1999. **211**(4): p. 314-318.

1485. Schroder, J.O., R.A. Zeuner, and C. Specker, *Systemic lupus erythematosus and antiphospholipid syndrome.* Aktuelle Rheumatologie, 2010. **35**(1): p. 24-32.

1486. Seczynska, B., et al., *[Plasmapheresis--possible complications and their prevention].* Przeglad lekarski, 2011. **68**(9): p. 637-640.

1487. Seidel, D. and J. Thiery, *Extracorporeal plasma therapy in lipid metabolism disturbances: Experience with the heparin-induced extracorporeal LDL precipitation (HELP) system.* Internist, 1992. **33**(1): p. 54-61.

1488. Shafro, L.I., et al., *Possible complications of therapeutic plasmapheresis.* Anesteziologiya i Reanimatologiya, 1991. **1**(5): p. 52-53.

1489. Shibuya, N. and S. Fujishita, *[Complications with plasmapheresis].* Nihon Rinsho, 1991. **49 Suppl**: p. 567-71.

1490. Sirenko, I.N., et al., *The initial experience of using fraxiparin in extracorporeal detoxication in clinical cardiology.* Klinicheskaia khirurgiia, 1994(12): p. 23-25.

1491. Straube, A., *25 Years of progress in neurology: An indispensable part of emergency medical care today.* MMW-Fortschritte der Medizin, 2008. **150**(48): p. 154-156.

1492. Sun, Y., *Progress in diagnosis and treatment of hypertriglyceridemic acute pancreatitis.* World Chinese Journal of Digestology, 2020. **28**(24): p. 1223-1228.

1493. Sunderkotter, C. and K. De Groot, *Therapy of vasculitides and vasculopathies.* Hautarzt, 2008. **59**(5): p. 382-393.

1494. Tanaka, I. and N. Shima, *[Acquired hemophilia: current status in Japan and immuno-biochemical features of autoantibodies to factor VIII].* Rinsho Ketsueki, 2005. **46**(2): p. 91-8.

1495. Tang, Z.X., et al., *Non-bioartificial liver plasma exchange in auxiliary treatment of chronic severe hepatitis.* Journal of Clinical Rehabilitative Tissue Engineering Research, 2008. **12**(53): p. 10487-10491.

1496. Trekova, N.A., et al., *Ways of reducing donor blood components during reconstructive surgery of the heart valves under extracorporeal circulation.* Anesteziologiia i reanimatologiia, 2008(5): p. 36-40.

1497. Ueda, Y., *[Treatment of hemolytic uremic syndrome].* Nihon Rinsho, 1993. **51**(1): p. 215-21.

1498. Ueda, Y., *[Treatment of thrombotic thrombocytopenic purpura].* Rinsho Ketsueki, 2014. **55**(10): p. 2076-86.

1499. Wang, B., et al., *[Determination of selenium species in food by high performance liquid chromatography with inductively coupled plasma mass spectrometry].* Se Pu, 2011. **29**(3): p. 223-7.

1500. Wang, F., et al., *[Study on preparation and property of a new adsorbent for endotoxin removal in blood purification].* Sheng Wu Yi Xue Gong Cheng Xue Za Zhi, 2013. **30**(3): p. 635-40.

1501. Weiner, S.M., et al., *Neuropsychiatric involvement in systemic lupus erythematosus. Part 2: Diagnostics and therapy.* Medizinische Klinik, 2003. **98**(2): p. 79-90.

1502. Xu, X., et al., *[Clinical application of blood purification (artificial liver) in treatment of acute liver failure in children].* Zhonghua Er Ke Za Zhi, 2014. **52**(6): p. 433-7.

1503. Xu, Y.W., et al., *[Attention should be paid to the application of regional citrate anticoagulation in blood purification].* Zhonghua Yi Xue Za Zhi, 2023. **103**(8): p. 541-544.

1504. Yamabuki, K., et al., *[Mitral valve re-replacement for a patient with multiple myeloma].* Nihon Kyobu Geka Gakkai Zasshi, 1993. **41**(9): p. 1582-5.

1505. Yamashita, M., et al., *[Anticoagulant in plasmapheresis].* Nihon Rinsho, 1991. **49 Suppl**: p. 572-5.

1506. Yamazaki, M., *[Catastrophic antiphospholipid syndrome: CAPS].* Nihon Rinsho Meneki Gakkai Kaishi, 2005. **28**(6): p. 357-64.

1507. Yang, D.H. and C.H. Chen, *Catastrophic antiphospholipid syndrome.* Journal of Internal Medicine of Taiwan, 2009. **20**(4): p. 335-343.

1508. Yoshida, M., *[Pathogenesis of antineutrophil-cytoplasmic antibody associated vasculitis].* Arerugi, 2008. **57**(1): p. 32-6.

1509. Yoshizawa, T., T. Suzuki, and K. Kanmatsuse, *[Usefulness of LDL-apheresis for treatment of steroid-resistant nephrotic syndrome].* Nihon Jinzo Gakkai Shi, 2003. **45**(1): p. 25-31.

1510. Zhao, S., et al., *[Application value of continuous blood purification in pediatric intensive care unit: analysis of 203 cases].* Zhonghua Wei Zhong Bing Ji Jiu Yi Xue, 2018. **30**(12): p. 1150-1153.

1511. Zhong, S., D.P. Yang, and Z. Cui, *[Studies on anticoagulant constituents in dried Whitmania pigra].* Zhongguo Zhong Yao Za Zhi, 2008. **33**(23): p. 2781-4.

1512. Abbasi, S., et al., *Vaccine-Induced Thrombotic Thrombocytopenia: A Case of Splanchnic Veins Thrombosis.* Cureus, 2022. **14**(3): p. e23507.

1513. Abd-Elsayed, A.A., et al., *Simultaneous antiphospholipid syndrome and heparin-induced thrombocytopenia in a single patient.* A and A Case Reports, 2014. **2**(1): p. 9-10.

1514. Abdo, M.S., et al., *Greasy blood: A case of profound hypertriglyceridemia and ensuing complications.* American Journal of Gastroenterology, 2021. **116**(SUPPL): p. S673-S674.

1515. Abdullah, A., et al., *Severe hypertriglyceridemia in pregnancyand plasmapheresis: A case report.* Obstetric Medicine, 2019. **12**(2 SUPPL): p. 3.

1516. Abrams, R.M.C. and G.A. Elder, *Safety of Therapeutic Plasma Exchange for the Treatment of Guillain-Barre Syndrome in Polycythemia Vera.* Neurologist, 2018. **23**(6): p. 185-187.

1517. Abu Sayf, A., F. Virk, and G. Tatem, *A case of catastrophic antiphospholipid antibody syndrome.* Chest, 2016. **150**(4 Supplement 1): p. 233A.

1518. Abuzamel, M., et al., *A 21-YEAR-OLD PREGNANT FEMALE WITH BILATERAL PULMONARY CAVITARY LESIONS AND KIDNEY DISEASE.* Chest, 2021. **160**(4 Supplement): p. A2094-A2095.

1519. Acar, Y.A., et al., *Concomitant hypertriglyceridemia-induced pancreatitis in pregnant monozygotic twin siblings.* Gynecol Endocrinol, 2020. **36**(7): p. 654-656.

1520. Acik, D.Y., *Acquired Combined Factor Deficiency: Case Report.* Clin Lab, 2020. **66**(9).

1521. Adhikari, A., et al., *Rare case of catastrophic antiphospholipid syndrome with spontaneous intracranial haemorrhage.* BMJ Case Reports, 2019. **12**(3): p. e227171.

1522. Ahmed, S., et al., *CATASTROPHIC ANTIPHOSPHOLIPID SYNDROME PRESENTING AS DIFFUSE PERIPHERAL AND CENTRAL THROMBOSES.* Rheumatology Advances in Practice, 2021. **5**(Supplement 1): p. i21.

1523. Ahmed, S., T. Nasrin, and L. Steel, *A rare cause of diffuse peripheral and central thromboses.* British Journal of Haematology, 2021. **193**(SUPPL 1): p. 224.

1524. Ahmed, S.H., et al., *Use of extracorporeal membrane oxygenation in a patient with diffuse alveolar hemorrhage.* Chest, 2004. **126**(1): p. 305-309.

1525. Ahn, E.R., et al., *Long-term remission from life-threatening hypercoagulable state associated with lupus anticoagulant (LA) following rituximab therapy.* Am J Hematol, 2005. **78**(2): p. 127-9.

1526. Ahonkai, A. and N. Lechtzin, *A Case for Vaccination.* American Journal of Medicine, 2007. **120**(4): p. 319-321.

1527. Al Hamdani, S., et al., *Child with Guillain-Barre Syndrome Responding to Plasmapheresis: A Case Report.* Case Reports in Acute Medicine, 2020. **3**(1): p. 4-11.

1528. Al sanani, A. and N.J. Poznanski, *Therapeutic plasma exchange improved pregnancy outcomes in a patient with triple positive anti-phospholipid antibody syndrome.* Journal of the American Society of Nephrology, 2021. **32**: p. 682.

1529. Al Turk, Y., et al., *COVID-19-RELATED CATASTROPHIC ANTIPHOSPHOLIPID SYNDROME: CASE REPORT.* Chest, 2021. **160**(4 Supplement): p. A720.

1530. Aldrete, K., K. Taylor, and J. Dong, *TACROLIMUS IN RENAL TRANSPLANT AND THE DEVELOPMENT OF THROMBOTIC MICROANGIOPATHY.* Chest, 2020. **158**(4 Supplement): p. A984.

1531. Alexandre, A., et al., *Percutaneous patent foramen ovale closure after a thrombotic storm: A case report.* Cardiovascular Research, 2022. **118**(Supplement 2): p. ii101.

1532. Alexopoulos, E., et al., *Cryofibrinogenemia due to Henoch-Schonlein purpura in a patient on peritoneal dialysis.* Peritoneal Dialysis International, 2003. **23**(1): p. 85-87.

1533. Algethamy, H.M., Y.A. Shikdar, and T.A. Alansari, *A case of severe hypercalcemia with arterial and venous thrombosis.* Anaesthesia, Pain and Intensive Care, 2020. **24**(1): p. 111-114.

1534. Ali Hassan, M., et al., *Catastrophic antiphospholipid syndrome associated with mitral valve thrombosis.* Journal of Investigative Medicine, 2021. **69**(2): p. 546-547.

1535. Ali, M. and S. Faryad, *Catastrophic antiphospholipid syndrome and COVID-19: A clinical conundrum.* Critical Care Medicine, 2022. **50**(1 SUPPL): p. 72.

1536. Aliasgarzadeh, S., et al., *Sudden development of the upper and lower limb ischemia as the first manifestation of COVID-19 infection.* Int J Surg Case Rep, 2022. **96**: p. 107332.

1537. Allee, L. and T. Buck, *Heparin induced thrombocytopenia in patient requiring heart transplantation: A case report.* Journal of Investigative Medicine, 2012. **60**(1): p. 409-410.

1538. Alshehri, S., et al., *Transfusion Plasma Exchange (TPE) and The Supratheraputic Effects of Unfractionated Heparin (UFH): A Case Report and Review of Literature.* JACCP Journal of the American College of Clinical Pharmacy, 2022. **5**(7): p. 782.

1539. Ambaglio, C., et al., *Plasma exchange and immunosuppressive therapy in a case of mild haemophilia A with inhibitors and a life-threatening lower limb haemorrhage.* Blood Transfus, 2014. **12**(1): p. 119-23.

1540. Amoura, Z., et al., *Thrombotic thrombocytopenic purpura with severe ADAMTS-13 deficiency in two patients with primary antiphospholipid syndrome.* Arthritis and Rheumatism, 2004. **50**(10): p. 3260-3264.

1541. Ananthanarayanan, V., S.M. Meehan, and S.R. Marino, *Thrombotic microangiopathy (TMA) in patients with antiphospholipid syndrome is an important cause of acute renal graft failure: A case report.* American Journal of Clinical Pathology, 2011. **136**(3): p. 469-470.

1542. Andermatt, R., et al., *Elimination of Herpes Simplex Virus-2 and Epstein-Barr Virus With Seraph 100 Microbind Affinity Blood Filter and Therapeutic Plasma Exchange: An Explorative Study in a Patient With Acute Liver Failure.* Crit Care Explor, 2022. **4**(8): p. e0745.

1543. Andev, R.S., et al., *CATASTROPHIC ANTIPHOSPHOLIPID CRISIS TRIGGERED BY ANTICOAGULANT SWITCH.* Rheumatology Advances in Practice, 2021. **5**(Supplement 1): p. i16.

1544. Andrievskaya, M., et al., *SEVERE METABOLIC ALKALOSIS IN PREGNANT PATIENT DUE TO CITRATE LOAD WITH PLASMA EXCHANGE.* American Journal of Kidney Diseases, 2019. **73**(5): p. 652.

1545. Andronesi, A., et al., *Catastrophic antiphospholipid syndrome due to systemic lupus erythematosus: The beneficial effect of rituximab treatment.* Journal of the American Society of Nephrology, 2018. **29**: p. 1119.

1546. Antonijevic, N.M., et al., *Salvage late plasmapheresis in a patient with pulmonary embolism caused by heparin-induced thrombocytopenia primarily resistant to danaparoid sodium and lepirudin.* J Clin Apher, 2006. **21**(4): p. 252-5.

1547. Arakawa, M., et al., *[A case of hemolytic uremic syndrome associated with circulating anticoagulant].* Nihon Jinzo Gakkai Shi, 1995. **37**(9): p. 523-8.

1548. Aribandi, A., et al., *Refractory thrombotic thrombocytopenic purpura-a case report.* Research and Practice in Thrombosis and Haemostasis, 2021. **5**(SUPPL 2).

1549. Arogundade, F.A., et al., *Filter membrane-based automated therapeutic plasma exchange: A report of two cases from Nigeria.* Journal of Clinical Apheresis, 2013. **28**(1): p. 78-83.

1550. Arora, K., D. Feldman, and G. Meny, *Effectiveness of standard plasmapheresis for genetic ldlhypercholesterolemia.* Journal of Clinical Apheresis, 2014. **29**(1): p. 50-51.

1551. Asaka, M., et al., *Hemolytic uremic syndrome associated with influenza A virus infection in an adult renal allograft recipient: case report and review of the literature.* Nephron, 2000. **84**(3): p. 258-66.

1552. Assante, W., et al., *26 THROMBOTIC MICROANGIOPATHY FROM APLS NEPHROPATHY RESPONSIVE TO THERAPEUTIC PLASMA EXCHANGE: A CASE REPORT.* American Journal of Kidney Diseases, 2021. **77**(4): p. 575.

1553. Assar, S., et al., *Successful treatment of COVID-19 induced neutrophilic myositis with intravenous immunoglobulin and corticosteroids: A case report.* Reumatismo, 2021. **73**(4): p. 232-235.

1554. Atiya, S., R.I. Marar, and A. Bobr, *Successful use of plasma exchange preceding RBC exchange in sickle cell disease hyperhemolytic crisis with hepatic sequestration: A case report.* Blood, 2021. **138**(SUPPL 1): p. 4279.

1555. Atluri, S., et al., *Dramatic improvement in severe triglyceride-induced acute pancreatitis after one treatment with plasmapheresis: A case report.* American Journal of Gastroenterology, 2013. **108**(SUPPL. 1): p. S261.

1556. Atrash, S., et al., *Fatal thrombotic microangiopathy developing within 24 hours of carfilzomib in a patient with relapsed multiple myeloma (MM).* Blood, 2012. **120**(21).

1557. Atrash, S., et al., *Three cases of patients with complement regulatory factor genetic mutations and acquired thrombotic thrombocytopenic purpura (TTP).* Blood, 2016. **128**(22).

1558. Ayanniyi, A.A., et al., *Blinding bilateral hyperviscosity retinopathy in a 43-year-old nigerian male with lymphoplasmacytic lymphoma: a case report and management challenges.* Case Rep Oncol Med, 2014. **2014**: p. 567632.

1559. Babarczy, K., et al., *A longitudinally extensive H3 K27M-mutant diffuse midline glioma in an elderly patient clinically mimicking central nervous system inflammation: A case report.* Folia Neuropathologica, 2021. **58**(4): p. 377-385.

1560. Bae, J., et al., *An unusual presentation of new systemic lupus erythematous: Concomitant methicillin-susceptible staphylococcus aureus bacteremia and severe hyponatremia.* American Journal of Respiratory and Critical Care Medicine, 2021. **203**(9).

1561. Bae, J.H., et al., *Acute pancreatitis due to hypertriglyceridemia: report of 2 cases.* Korean J Gastroenterol, 2005. **46**(6): p. 475-80.

1562. Bae, J.Y., et al., *Seronegative Goodpasture's syndrome associated with organising pneumonia.* BMJ Case Reports, 2021. **14**(2): p. e239390.

1563. Bahgat, J., et al., *A HISTOLOGICAL DIAGNOSIS OF CATASTROPHIC ANTIPHOSPHOLIPID SYNDROME.* Chest, 2022. **161**(1 Supplement): p. A182.

1564. Bahlavouni, A. and A. Paul, *Heart failure in a previously healthy adolescent presenting with lower extremity rash.* Pediatrics, 2021. **147**(3): p. 864-866.

1565. Bajpai, M., B. Kakkar, and D. Patale, *Role of high-volume plasma exchange in a case of a G6PD deficient patient presenting with HAV related acute liver failure and concomitant acute renal failure.* Transfusion and Apheresis Science, 2019. **58**(6): p. 102677.

1566. Balas, J.S., et al., *ATYPICAL HEMOLYTIC UREMIC SYNDROME LOOKING BEYOND THE TYPICAL DIAGNOSIS.* Journal of General Internal Medicine, 2022. **37**(Supplement 2): p. S422-S423.

1567. Banothu, K.K., et al., *Case Report: Extensive Gangrene: A Rare Presentation of Multisystem Inflammatory Syndrome in Children.* American Journal of Tropical Medicine and Hygiene, 2022. **107**(6): p. 1245-1249.

1568. Barroso, J., K. Alcorn, and Y. Wu, *Therapeutic plasma exchange in a case of refractory post transfusion purpura.* Journal of Clinical Apheresis, 2016. **31**(2): p. 127.

1569. Bartley, C.M., et al., *Case Report: A False Negative Case of Anti-Yo Paraneoplastic Myelopathy.* Frontiers in Neurology, 2021. **12**: p. 728700.

1570. Basiri, K., F. Fatehi, and F. Derakhshan, *Pharyngeal-cervical-brachial variant of Guillain-Barre syndrome in a patient with thalassemia intermedia.* Neurosciences, 2009. **14**(1): p. 71-74.

1571. Battelino, N., et al., *Surgical thrombectomy of central venous catheter related thrombus in the right atrium in a girl treated with plasmapheresis.* Pediatric Nephrology, 2016. **31**(10): p. 1904.

1572. Batum, M., A. Kisabay Ak, and H. Mavioglu, *Covid-19 infection-induced neuromyelitis optica: a case report.* International Journal of Neuroscience, 2022. **132**(10): p. 999-1004.

1573. Beck, E. and L.M. Keenan, *Diffuse alveolar hemorrhage necessitating extracorporeal membrane oxygenation rescue therapy.* American Journal of Respiratory and Critical Care Medicine, 2018. **197**(MeetingAbstracts).

1574. Beg, M., et al., *Catastrophic antiphospholipid syndrome or peripartum lupus flare: A diagnostic and therapeutic challenge.* Chest, 2017. **152**(4 Supplement 1): p. A315.

1575. Beigmohammadi, M.T., et al., *Quadriplegia due to lead-contaminated opium--case report.* Middle East J Anaesthesiol, 2008. **19**(6): p. 1411-6.

1576. Bek, S.G., et al., *Rh (D) alloimmunization treated by double filtration plasmapheresis.* Transfusion and Apheresis Science, 2019. **58**(1): p. 83-86.

1577. Beliard, S., et al., *Efficient rituximab therapy in a patient with an autoimmune type I hyperlipidemia.* Atherosclerosis, 2014. **235**(2): p. e59.

1578. Belobradkova, M., et al., *Overlap of the symptoms of thrombotic thrombocytopenic purpura and antiphospholipid syndrome in a patient with juvenile systemic lupus erythematosus - Case report.* Ceska Revmatologie, 2017. **25**(4): p. 190-198.

1579. Benitez Vazquez, E.A., et al., *Juvenile systemic lupus erythematosus and thrombotic thrombocytopenia purpura, case report simultaneous diagnsosis in a 5 years old girl.* Pediatric Rheumatology, 2018. **16**(Supplement 2).

1580. Berezne, A., et al., *Acute alveolar haemorrhage and primary anti-phospholipid syndome: A case report and review of the literature.* Nephrologie, 2004. **25**(2): p. 53-57.

1581. Bergh, C.C. and R. Malhotra, *Treatment of ECMO-related heparin-induced thrombocytopenia through plasmapheresis and bilvalirudin with subsequent reduction of antibody titer.* American Journal of Respiratory and Critical Care Medicine, 2013. **187**(MeetingAbstracts).

1582. Bernardini, K., et al., *Monolateral renal infarction and erythromelalgia in a case of chronic myelogenous leukemia [9].* American Journal of Hematology, 2006. **81**(3): p. 224-225.

1583. Betrosian, A.P., et al., *Meningococcal purpura fulminans in a patient with systemic lupus erythematosus: A mimic for catastrophic antiphospholipid antibody syndrome?* American Journal of the Medical Sciences, 2004. **327**(6): p. 373-375.

1584. Bhalla, S. and I. Swanenberg, *Severe thrombocytopenia induced by vancomycin-dependent anti-platelet antibodies.* Journal of Hospital Medicine, 2018. **13**(4 Supplement 1).

1585. Bhatt, N., *A CASE OF INTERMITTENT HEMOPTYSIS: THE SPECTRUM OF ANTIPHOSPHOLIPID ANTIBODY SYNDROME.* Chest, 2022. **161**(1 Supplement): p. A427.

1586. Bikhchandani, M., *Unusual case of concurrent myeloproliferative neoplasm and catastrophic anti-phospholipid syndrome.* Journal of General Internal Medicine, 2018. **33**(2 Supplement 1): p. 656.

1587. Bittar, P.G., M.S. Nickolich, and O.A. Onwuemene, *ASFA Category IV becomes Category I: Idiopathic thrombotic thrombocytopenic purpura in a patient with presumed gemcitabine-induced thrombotic microangiopathy.* Journal of Clinical Apheresis, 2018. **33**(3): p. 423-426.

1588. Boggio, F., et al., *Plasma Exchange in a Patient with Immune Thrombocytopenia Associated with Antiphospholipid Syndrome Hospitalized for COVID-19.* Rheumatol Ther, 2022. **9**(4): p. 1213-1219.

1589. Bokhoven, K.C., et al., *Impending extracorporeal life support system shutdown in a patient with hypertriglyceridemia and heparin resistance.* European Journal of Heart Failure, 2017. **19**(Supplement 2): p. 36.

1590. Bornacelly, A. and E. Moreno-Pallares, *Thrombotic microangiopathy in a patient with covid-19: A case report.* Journal of Clinical Rheumatology, 2021. **27**(SUPPL 1): p. S47-S48.

1591. Borra, P. and C.E. Mellon, *A Case of Mistaken Identity: TTP-like State Due to Heroin Overdose.* Blood, 2019. **134**(Supplement 1): p. 4905.

1592. Bortolati, M., et al., *Case reports of the use of immunoadsorption or plasma exchange in high-risk pregnancies of women with antiphospholipid syndrome.* Ther Apher Dial, 2009. **13**(2): p. 157-60.

1593. Boseniuk, S. and C. Rieger, *[Acute oral acetic acid poisoning--case report].* Anaesthesiol Reanim, 1994. **19**(3): p. 80-2.

1594. Boseniuk, S. and C. Rieger, *Acute oral acetic acid intoxication - Case report.* Anaesthesiologie und Reanimation, 1994. **19**(3): p. 80-82.

1595. Brady, J., et al., *Plasmapheresis. A therapeutic option in the management of heparin-associated thrombocytopenia with thrombosis.* Am J Clin Pathol, 1991. **96**(3): p. 394-7.

1596. Bremaud, M., et al., *Three cases of IgM monoclonal gammopathy with haemostasis dysfunction.* Annales Francaises d'Anesthesie et de Reanimation, 2009. **28**(10): p. 892-896.

1597. Bridges, B.C., D. Hardison, and J. Pietsch, *A case series of the successful use of ECMO, continuous renal replacement therapy, and plasma exchange for thrombocytopenia-associated multiple organ failure.* Journal of Pediatric Surgery, 2013. **48**(5): p. 1114-1117.

1598. Broussard, J., M. Berlinger, and D. Lauret, *A Clot (Possibly); Due to Loss of TNF-α Supression.* J La State Med Soc, 2017. **169**(2): p. 52.

1599. Brown, L., L. Tilzer, and F. Plapp, *Factor V and VIII deficiency treated with therapeutic plasma exchange prior to redo mitral valve replacement.* Journal of Clinical Apheresis, 2017. **32**(3): p. 196-199.

1600. Buitendag, J.J.P., et al., *Using fresh frozen plasma as an emergency treatment modality for hypertriglyceridaemic acute pancreatitis in a resource-constrained environment: A case report and review of literature.* Tropical Doctor, 2021. **51**(4): p. 650-651.

1601. Bunker, D.R., et al., *A 26-year-old woman with respiratory decompensation in the immediate postpartum period at Mount Sinai Medical Center in New York City.* Thorax, 2015. **70**(11): p. 1095-1097.

1602. Buntak, V., et al., *CATASTROPHIC ANTIPHOSPHOLIPID SYNDROME: A VICIOUS CIRCLE OF CLOTTING AND BLEEDING.* Chest, 2022. **162**(4 Supplement): p. A815.

1603. Burger, E., et al., *Acute syndrome of pan-epidermolysis and thrombotic storm arising in a patient with systemic lupus erythematosus.* JAAD Case Reports, 2018. **4**(9): p. 877-879.

1604. Burgos Pratx, L.D., et al., *Management of factor XI deficiency in oncological liver and colorectal surgery by therapeutic plasma exchange: A case report.* Transfusion and Apheresis Science, 2021. **60**(5): p. 103176.

1605. Butt, N., F. Sheikh, and J. Grinstein, *Successful Heartmate 3 LVAD Implantation in a Patient with Heparin Induced Thrombocytopenia Using Pre-Implantation Plasmapheresis and Intraoperative Continuous Prostacyclin.* Journal of Cardiac Failure, 2019. **25**(8 Supplement): p. S114.

1606. Buturović-Ponikvar, J., A.M. Pernat, and R. Ponikvar, *Citrate anticoagulation during plasma exchange in a patient with thrombotic thrombocytopenic purpura: short heparin-free hemodialysis helps to attenuate citrate load.* Ther Apher Dial, 2005. **9**(3): p. 258-61.

1607. Cakar Turhan, K.S., et al., *Selective plasma exchange with evaclio in a patient with angiotensin receptor blocker and calcium channel blocker intoxication.* Transfusion and Apheresis Science, 2012. **47**(SUPPL. 1): p. S50-S51.

1608. Calonge Arribas, A., A. Goyache Moreno, and A. Castiella, *Castatrophic antiphospholipid syndrome in a young woman.* Research and Practice in Thrombosis and Haemostasis, 2022. **6**(Supplement 1).

1609. Campbell, A. and O.A. Ogundipe, *Neuromyelitis optica spectrum disorder presenting in an octogenarian.* BMJ Case Reports, 2018. **2018**: p. bcr-2018-225601.

1610. Canales, M.A., et al., *Successful therapy with intravenous immunoglobulins and plasmapheresis in a pregnant woman with both antiphospholipid antibodies and Rh alloimmunization.* Medicina Clinica, 1999. **113**(11): p. 438-439.

1611. Candar, O., et al., *Therapeutic plasma exchange in gastric signet ring cell carcinoma presenting as microangiopathic hemolytic anemia: A rare case report.* Journal of Clinical Apheresis, 2022. **37**(6): p. 606-610.

1612. Cangialosi, P., et al., *HEPARIN-INDUCED THROMBOCYTOPENIA COMPLICATING CARDIOGENIC SHOCK REQUIRING DURABLE MECHANICAL CIRCULATORY SUPPORT.* Journal of the American College of Cardiology, 2023. **81**(8 Supplement): p. 3602.

1613. Canova, C.R., et al., *[Fatal central pulmonary embolism under heparin therapy: white-clot syndrome].* Schweiz Med Wochenschr, 1997. **127**(18): p. 762-5.

1614. Cao, L., et al., *Magnetic resonance imaging and magnetic resonance venography features in heat stroke: A case report.* BMC Neurology, 2019. **19**(1): p. 133.

1615. Cao, Y., et al., *Management of dermatomyositis patients amidst the COVID-19 pandemic: Two case reports.* Medicine (Baltimore), 2022. **101**(38): p. e30634.

1616. Caridi-Scheible, M., M. Connor, and J. Zivot, *Successful treatment of pulmonary-renal syndrome in HIT-positive patient using ECMO with argatroban.* Critical Care Medicine, 2013. **41**(12 SUPPL. 1): p. A292.

1617. Castro, P., et al., *[Catastrophic antiphospholipid syndrome and acute heart failure. Report of a case].* Rev Med Chil, 2003. **131**(9): p. 1037-41.

1618. Cede, A.Z., et al., *Hemodialysis venous catheter related thrombus in the right atrium: Report of three cases.* Nephrology Dialysis Transplantation, 2019. **34**(Supplement 1): p. a558.

1619. Celik, G., et al., *An uncommon presentation of Sjogren's syndrome and brucellosis.* Transfusion and Apheresis Science, 2014. **51**(1): p. 77-80.

1620. Cerier, E., B.A. Whitson, and A. Kilic, *Case report: Spontaneous iliac intramuscular bleed after orthotopic heart transplant.* Experimental and Clinical Transplantation, 2017. **15**(6): p. 700-701.

1621. Chai, M., J. Sims, and S. Pancholi, *Catastrophe averted-the importance of early diagnosis of catastrophic antiphospholipid syndrome.* Journal of Hospital Medicine, 2018. **13**(4 Supplement 1).

1622. Chakraborty, R., M. Bilal, and T. Bashir, *An unusual case of double antibody positive goodpasture's syndrome with immune mediated thrombocytopenia.* Internet Journal of Hematology, 2012. **8**(1).

1623. Chan, K.M., C.Y. Cheung, and K.F. Chau, *Heparin-induced thrombocytopenia due to heparin lock in a hemodialysis patient: A case report.* Hemodialysis International, 2014. **18**(2): p. 555-558.

1624. Chandok, T., et al., *Furosemide-Induced Thrombotic Thrombocytopenic Purpura: A Report of a Rare Case.* Cureus, 2022. **14**(6): p. e25689.

1625. Chandrasekar, M., et al., *Therapeutic plasma exchange (TPE) in a immune mediated hemolytic anaemia (IMHA) affected dog - A case report.* Indian Veterinary Journal, 2019. **96**(3): p. 63-64.

1626. Chang, B.S.F., et al., *Thrombotic microangiopathy (TMA) in a patient with COVID-19.* Journal of the American Society of Nephrology, 2020. **31**: p. 285.

1627. Channa, H. and P. Charoenpong, *PRESENTATION OF ACUTE CEREBROVASCULAR ACCIDENT IN A YOUNG ADULT WITH THROMBOTIC THROMBOCYTOPENIA PURPURA (TTP).* Chest, 2022. **162**(4 Supplement): p. A1158.

1628. Chatila, A., et al., *Clostridioides difficile-associated hemolytic uremic syndrome after orthotopic liver transplantation.* American Journal of Gastroenterology, 2019. **114**(Supplement): p. S1278.

1629. Chavda, S.J., et al., *A young woman with fever and loss of consciousness.* BMJ (Online), 2018. **363**: p. k4363.

1630. Chen, D.K., J.S. Kim, and D.M.C. Sutton, *Thrombotic thrombocytopenic purpura associated with ticlopidine use: A report of 3 cases and review of the literature.* Archives of Internal Medicine, 1999. **159**(3): p. 311-314.

1631. Chen, E., et al., *Transplantation in a patient on extracorporeal membrane oxygenation with infective endocarditis, pericarditis and heparininduced thrombocytopenia.* Interactive Cardiovascular and Thoracic Surgery, 2017. **24**(3): p. 462-463.

1632. Chen, G., et al., *Simultaneous ABO-incompatible living-donor liver transplantation and splenectomy without plasma exchange in China: Two case reports.* J Int Med Res, 2017. **45**(6): p. 2146-2152.

1633. Chen, I., et al., *Illustrative Cases of Kidney Disease Unique to Women.* Seminars in Nephrology, 2017. **37**(4): p. 412-416.

1634. Chen, J., et al., *Successful treatment of plasma exchange-refractory thrombotic thrombocytopenic purpura with rituximab: A case report.* World Journal of Clinical Cases, 2020. **8**(12): p. 2617-2622.

1635. Chen, J. and S. Scrape, *Plasmapheresis to reduce antiphospholipid antibodies in a patient undergoing open heart surgery.* Transfusion, 2014. **54**(SUPPL. 2): p. 129A.

1636. Chen, M.J., H.F. Tien, and H.N. Ho, *Treatment of thrombotic microangiopathy in pregnancy with exchange: A report of two cases.* Journal of the Formosan Medical Association, 2002. **101**(12): p. 859-863.

1637. Chen, Q.T., et al., *Case report: Vaccine-induced immune thrombotic thrombocytopenia complicated by acute cerebral venous thrombosis and hemorrhage after AstraZeneca vaccines followed by Moderna COVID-19 vaccine booster and surgery.* Front Neurol, 2022. **13**: p. 989730.

1638. Chidharla, A., et al., *A case report of covid-associated catastrophic antiphospholipid syndrome successfully treated with eculizumab.* Journal of Blood Medicine, 2021. **12**: p. 929-933.

1639. Cho, J., A. Lateef, and G.S. Kew, *A case report of postpartum microangiopathic antiphospholipid antibody-associated syndrome secondary to retained products of conception.* International Journal of Rheumatic Diseases, 2016. **19**(Supplement 2): p. 57.

1640. Cho, J., et al., *Double-duty plasma exchange: patient with heparin-induced thrombocytopenia and bivadrelated acute intravascular hemolysis.* Journal of Clinical Apheresis, 2018. **33**(2): p. 173.

1641. Cho, J.H., et al., *Plasma exchange for heparin-induced thrombocytopenia in patients on extracorporeal circuits: A challenging case and a survey of the field.* J Clin Apher, 2019. **34**(1): p. 64-72.

1642. Choi, J.Y., et al., *Living donor renal transplantation in patients with antiphospholipid syndrome: A case report.* Medicine (Baltimore), 2016. **95**(46): p. e5419.

1643. Choi, J.Y., et al., *Living donor renal transplantation in patients with antiphospholipid syndrome.* Transplantation, 2016. **100**(7 Supplement 1): p. S584.

1644. Choi, M.Y., et al., *Metabolic alkalosis induced by plasmapheresis in a patient with systemic lupus erythematosus.* J Korean Med Sci, 1993. **8**(3): p. 207-9.

1645. Chopra, M., R. Garcia-Orr, and S. Sultan, *A case of oral sparing toxic epidermal necrolysis.* American Journal of Respiratory and Critical Care Medicine, 2017. **195**.

1646. Chowdhry, M., S. Agrawal, and M.L. S, *A case of Bickerstaff encephalitis with overlapping Gullian Barre syndrome in a pediatric patient treated with therapeutic plasma exchange.* Transfusion and Apheresis Science, 2021. **60**(6): p. 103260.

1647. Christiansen, T.K., et al., *Small intestine necrosis in catastrophic antiphospholipid syndrome: A rare and severe case.* Lupus, 2022. **31**(6): p. 754-758.

1648. Civelli, V.F., et al., *A Progressive Case of Eosinophilic Myocarditis Due to Eosinophilic Granulomatosis With Polyangiitis in a Caucasian Male.* Journal of Investigative Medicine High Impact Case Reports, 2020. **8**.

1649. Clay, E. and K. Johnson-Martinez, *Severe symptomatic hypocalcemia in a patient with hypertriglyceridemia-induced acute pancreatitis.* Journal of General Internal Medicine, 2017. **32**(2 Supplement 1): p. S590-S591.

1650. Cleri, D.J., et al., *Pulmonary Aspergillosis and Central Nervous System Hemorrhage as Complications of Autoimmune Hemolytic Anemia Treated with Corticosteroids.* Southern Medical Journal, 2003. **96**(6): p. 592-595.

1651. Cohee, B., et al., *Early empiric treatment prior to diagnosis of acute hemorrhagic leukoencephalitis.* Chest, 2013. **144**(4 MEETING ABSTRACT).

1652. Constantin, T., et al., *Antiphospholipid syndrome accompanied by a silent splenic infarct in a patient with juvenile SLE [2].* Rheumatology International, 2006. **26**(10): p. 951-952.

1653. Conti, F., et al., *Diagnosis of catastrophic anti-phospholipid syndrome in a patient tested negative for conventional tests.* Clin Exp Rheumatol, 2017. **35**(4): p. 678-680.

1654. Cook, O., et al., *Combined preoperative plasma exchange and red blood cell exchange transfusion in a renal transplant patient with protein S deficiency and hemoglobin SC disease.* Transfusion and Apheresis Science, 2022. **61**(3): p. 103345.

1655. Cortazar-Benitez, L.F., et al., *Hemolytic Intravascular Anemia (HIA) microangiopathic-like and Deep Venous Thrombosis (DVT) due to ozone exposure: Case report.* Blood, 2011. **118**(21).

1656. Cosmin, A., L. Juncos, and T. Fulop, *Therapeutic Plasma Exchange (TPE) for recurrent clotting during Continuous Renal Replacement Therapy (CRRT).* Blood Purification, 2009. **27**(3): p. 276.

1657. Costa, R., et al., *Successful plasma exchange combined with rituximab therapy in aggressive APS-related cutaneous necrosis.* Clin Rheumatol, 2013. **32 Suppl 1**: p. S79-82.

1658. Cottrell, J., et al., *Triple Positive Antiphospholipid Antibody Syndrome in Pregnancy with High Frequency Plasma Exchange: A Case Report.* Transfusion Medicine and Hemotherapy, 2022.

1659. Cristinar, M.D., J.C. Thiranos, and J.P. Dupeyron, *Heparin-induced thrombocytopenia (HIT): The management before and after the cardiac transplantation of a patient with positive anti-pf4/heparin antibodies. case report.* Intensive Care Medicine, 2010. **36**(SUPPL. 2): p. S148.

1660. Crowhurst, T., et al., *Progressive multifocal leukoencephalopathy in a lung transplant recipient presenting with memory impairment: Case report and literature review.* Transplant Infectious Disease, 2020. **22**(3): p. e13293.

1661. Crump, K.L. and R. Seshadri, *Use of therapeutic plasmapheresis in a case of canine immune-mediated hemolytic anemia.* J Vet Emerg Crit Care (San Antonio), 2009. **19**(4): p. 375-80.

1662. Cunningham, J. and S. Sadullah, *A case of 'double hit' thrombocytopaenia.* British Journal of Haematology, 2018. **181**(Supplement 1): p. 162.

1663. Dabar, G. and C. Harmouch, *[Alveolar haemorrhage associated with the primary antiphospholipid syndrome].* Rev Mal Respir, 2013. **30**(1): p. 71-6.

1664. Dahl, M.L.N. and B. Deleuran, *Positive pregnancy outcome in an anti-SSA positive female with SLE and factor V Leiden following bimonthly IVIG administration.* Scandinavian Journal of Rheumatology, 2022. **51**(5): p. 425-427.

1665. Dakowicz, L. and M. Krawczuk-Rybak, *Thrombotic thrombocytopenic purpura in a 15-year-old female.* Klin Padiatr, 2013. **225**(6): p. 362-3.

1666. D'Aloiso, B.D., et al., *Cardiopulmonary Bypass in the Setting of Waldenström's Macroglobulinemia.* J Extra Corpor Technol, 2018. **50**(2): p. 120-123.

1667. Damjanovska, L. and R. Rajcevski, *Antiphospholipid syndrome in patient with portal venous thrombosis: Case report.* Macedonian Journal of Medical Sciences, 2011. **4**(2): p. 192-195.

1668. D'Amore, F., C. Narduzzi, and A.M. Santoro, *[Thrombosis, LES, antiphospholipid antibodies: a case report].* Clin Ter, 1997. **148**(12): p. 675-8.

1669. Danielson, C., et al., *Immediate gross hemolysis due to hypotonic fluid administration during plasma exchange: A case report.* Journal of Clinical Apheresis, 1991. **6**(3): p. 161-162.

1670. Dar, W.R., et al., *Transverse myelitis in a patient with primary antiphospholipid syndrome.* Neurology India, 2015. **63**(6): p. 986-988.

1671. Das, D., S. Sitaula, and M. Chhetry, *Amniotic fluid embolism: A rare cause of maternal collapse-A case report.* Clinical Case Reports, 2020. **8**(12): p. 3359-3361.

1672. De Landtsheer, Q., et al., *Acute heart failure after thrombotic thrombocytopenic purpura successfully treated by ECLS.* Transfusion Medicine, 2013. **23**(3): p. 199-201.

1673. De Marchi, L., et al., *Catastrophic antiphospholipid syndrome with bilateral adrenal hemorrhage: A case report.* Acta Clinica Belgica, 2020. **74**(Supplement 1): p. 65-66.

1674. de Miguel-Sanchez, C.J., et al., *Plasmaexchangeasaneffectivesalvage therapy in AZD1222 vaccine-induced thrombotic thrombocytopenia: a case report.* Blood Transfusion, 2022. **20**(2): p. 152-155.

1675. Dean, R. and I. Amzuta, *MANAGEMENT CONSIDERATIONS IN A COMPLICATED CASE OF HUGHES-STOVIN SYNDROME.* Chest, 2020. **158**(4 Supplement): p. A1579.

1676. Decker, P., et al., *Thrombotic Thrombocytopenic Purpura Without Schistocytes: Beware of Misdiagnosis.* Neurology: Clinical Practice, 2021. **11**(5): p. E798-E800.

1677. Dedhia, P., et al., *Eculizumab and Belatacept for De Novo Atypical Hemolytic Uremic Syndrome Associated With CFHR3-CFHR1 Deletion in a Kidney Transplant Recipient: A Case Report.* Transplantation Proceedings, 2017. **49**(1): p. 188-192.

1678. Deepak, S., S. Rangaraj, and K. Warrier, *Pulmonary emboli in a teenager with GPA: Management dilemmas.* Rheumatology Advances in Practice, 2020. **4**(SUPPL 1): p. i15-i16.

1679. Demir, N.A., et al., *A case of Evans syndrome secondary to COVID-19.* Blood Transfus, 2021. **19**(1): p. 85-88.

1680. Demirkaya, E., et al., *Purpura fulminans as the presenting manifestation in a patient with juvenile SLE.* Turkish Journal of Pediatrics, 2009. **51**(4): p. 378-380.

1681. Destrampe, E. and W. Rose, *Plasmapheresis for the treatment of iatrogenic antithrombin excess: A case report.* Journal of Clinical Apheresis, 2019. **34**(2): p. 152.

1682. Dhal, U., et al., *Beyond hellp: peripartum catastrophic thromboembolism due to hereditary diffuse gastric cancer.* Critical Care Medicine, 2015. **43**(12 SUPPL. 1): p. 309.

1683. D'Hont, A., et al., *Successful Rituximab Therapy for Pediatric Antiphospholipid-Related Chorea: A Case Report and Review of the Literature.* Neuropediatrics, 2022. **53**(5): p. 366-369.

1684. Diaz Coto, J.F. and R. Barahona Cuellar, *Catastrophic antiphospholipid syndrome. Case report.* Revista Clinica Espanola, 1995. **195**(1): p. 29-30.

1685. Dickens, B., et al., *ARDS and Massive Pulmonary Embolism: The Combined Use of Extracorporeal Membrane Oxygenation (ECMO) with Thrombolytics.* Case Reports in Critical Care, 2020. **2020**: p. 1032629.

1686. Disel, N.R., et al., *Utilization of plasmapheresis in the management of bismuth intoxication with acute renal failure.* Saudi journal of kidney diseases and transplantation : an official publication of the Saudi Center for Organ Transplantation, Saudi Arabia, 2017. **28**(3): p. 629-632.

1687. Ditzel, K., D.J. Mons, and R. Fijnheer, *Fatal cerebral hemorrhage in a patient with thrombotic thrombocytopenic purpura with a normal platelet count during treatment with caplacizumab.* Platelets, 2022. **33**(3): p. 484-485.

1688. Doğru, A., et al., *Catastrophic antiphospholipid syndrome treated with rituximab: A case report.* Eur J Rheumatol, 2017. **4**(2): p. 145-147.

1689. Doll, J.A. and J.P. Kelly, *ST-segment elevation myocardial infarction treated with thrombolytic therapy in a patient with thrombotic thrombocytopenic purpura.* Journal of Thrombosis and Thrombolysis, 2014. **38**(1): p. 124-126.

1690. Donohue, K., et al., *Catastrophic antiphospholipid antibody syndrome presenting as intraabdominal abscess: An unusual case of thrombotic storm.* Chest, 2016. **150**(4 Supplement 1): p. 377A.

1691. Dreyer, G. and S. Fan, *Therapeutic implications of coexisting severe pulmonary hemorrhage and pulmonary emboli in a case of Wegener granulomatosis.* Am J Kidney Dis, 2009. **53**(5): p. e5-8.

1692. Duggal, N., et al., *Pulmonary Endarterectomy under Hypothermic Circulatory Arrest in a Patient with Heparin-Induced Thrombocytopenia.* Journal of Cardiothoracic and Vascular Anesthesia, 2016. **30**(3): p. 741-745.

1693. Duhaut, P., et al., *Idiopathic vasculitis with abdominal tropism and fatal case.* Revue de Medecine Interne, 1993. **14**(6): p. 516.

1694. Dukic, L., N. Maric, and A.M. Simundic, *Dark brown serum and plasma samples: A case report.* Biochemia Medica, 2020. **30**(2): p. 1-5.

1695. Dunkley, L., M. Green, and A. Gough, *A case of Raynaud's phenomenon in mixed connective tissue disease responding to Rituximab therapy - Response [14].* Rheumatology, 2007. **46**(10): p. 1628-1629.

1696. Duong-Quy, S., et al., *Guillain-Barre Syndrome in Patient With SARS-CoV-2 PCR Positivity Treated Successfully With Therapeutic Exchange Plasma: A First Case Report From Vietnam.* Frontiers in Neurology, 2022. **13**: p. 868667.

1697. Duong-Quy, S., et al., *Bradycardia unresponded to atropin testing was successfully treated with therapeutic plasma exchange in a patient with severe COVID-19 complicated by Guillain-Barre syndrome: A case report.* Frontiers in Cardiovascular Medicine, 2023. **9**: p. 1035896.

1698. Durak, C., et al., *A case of severe DRESS syndrome treated with therapeutic plasma exchange and intravenous immunoglobulin therapy.* Journal of Clinical Apheresis, 2022. **37**(6): p. 600-605.

1699. Dzhumabaeva, B.T., et al., *[A case of hemolytic-uremic syndrome with development of catastrophic antiphospholipid syndrome: diagnosis and clinical tactics].* Ter Arkh, 2010. **82**(3): p. 56-60.

1700. Ebrahim, J., et al., *Pulmonary and cardiac manifestations of the primary antiphospholipid antibody syndrome (APS).* Chest, 2014. **146**(4 MEETING ABSTRACT).

1701. Efthymiadis, A., et al., *A case of ChAdOx1 vaccine-induced thrombocytopenia and thrombosis syndrome leading to bilateral adrenal haemorrhage and adrenal insufficiency.* Endocrinol Diabetes Metab Case Rep, 2022. **2022**.

1702. Ekinci, F., et al., *Pulmonary embolism complicated the course of anti-N-methyl-D aspartate receptor encephalitis in a pediatric intensive care unit setting: a case report.* Postgraduate Medicine, 2021. **133**(1): p. 102-107.

1703. Elikowski, W., et al., *[Multifocal ischaemic stroke and myocardial infarction in a woman with occult lung cancer complicated with chronic DIC and thrombotic endocarditis].* Neurol Neurochir Pol, 2006. **40**(6): p. 530-5.

1704. Elkington, T., M. Ghrew, and R. Kishen, *Hemofiltration Fluid Warming and Predilution Facilitate Continuous Venovenous Hemofiltration in a Patient With Cryoglobulinemia.* American Journal of Kidney Diseases, 2009. **53**(4): p. 720.

1705. Elmas, A.T., et al., *Abdominal pain, nausea, vomiting, and ascites in a 14-year-old girl with systemic lupus erythematosus: Answers.* Pediatr Nephrol, 2019. **34**(3): p. 431-433.

1706. El-Rayes, B.F. and M. Edelstein, *Unusual case of antiphospholipid antibody syndrome presenting with extensive cutaneous infarcts in a patient on long-term procainamide therapy [5].* American Journal of Hematology, 2003. **72**(2): p. 154.

1707. Emami, F., et al., *A Case of Guillain-Barre Syndrome After Anterolateral Myocardial Infarction and Percutaneous Coronary Intervention for it.* Iranian Heart Journal, 2022. **23**(3): p. 120-125.

1708. Ermis Turak, E., et al., *Therapeutic plasma exchange in g6pd deficient patient complicated with intravascular hemolysis: A case report.* Erciyes Medical Journal, 2020. **42**(2): p. 223-225.

1709. Ernst, E., M. Girndt, and R.U. Pliquett, *A case of granulomatosis with polyangiitis complicated by cyclophosphamide toxicity and opportunistic infections: Choosing between Scylla and Charybdis.* BMC Nephrology, 2014. **15**(1): p. 28.

1710. Espiritu, J.D., et al., *Fatal tumor thrombosis due to an inferior vena cava leiomyosarcoma in a patient with antiphospholipid antibody syndrome.* Mayo Clinic Proceedings, 2002. **77**(6): p. 595-599.

1711. Estep, Z., et al., *A YOUNG WOMAN PRESENTED WITH SEPTIC SHOCK AND ENDOCARDITIS.* Journal of the American College of Cardiology, 2021. **77**(18 Supplement 1): p. 2876.

1712. Exbrayat, V., et al., *Hypertriglycideraemia-induced pancreatitis in pregnancy. A case report.* Annales Francaises d'Anesthesie et de Reanimation, 2007. **26**(7-8): p. 677-679.

1713. Fan, R., et al., *Severe hemolysis in a patient with erythrocytosis during coupled plasma filtration adsorption therapy was prevented by changing from membrane-based technique to a centrifuge-based one.* American Journal of Therapeutics, 2016. **23**(4): p. e1124-e1127.

1714. Faqihi, F., et al., *Reverse takotsubo cardiomyopathy in fulminant COVID-19 associated with cytokine release syndrome and resolution following therapeutic plasma exchange: a case-report.* BMC Cardiovasc Disord, 2020. **20**(1): p. 389.

1715. Farooq, A., A. Treml, and J.M. Colon-Franco, *A woman with pancreatitis and hypertriglyceridemia.* Clinical Chemistry, 2019. **65**(10): p. 1216-1218.

1716. Farooq, A., A. Treml, and J.M. Colón-Franco, *A Woman with Pancreatitis and Hypertriglyceridemia.* Clin Chem, 2019. **65**(10): p. 1216-1218.

1717. Farris, L., S. Nhieu, and S. Saatee, *Plasma exchange and heparin use for vad placement in a patient with heparin-induced thrombocytopenia.* Critical Care Medicine, 2018. **46**(Supplement 1): p. 49.

1718. Fazal, S., et al., *Successful plasma exchange combined with rituximab therapy in aggressive APS-related cutaneous necrosis.* Clinical Rheumatology, 2013. **32**(SUPPL. 1): p. 79-82.

1719. Fen, T. and B. Goren, *A Waldenstrom's macroglobulinaemia case secreting IgM monoclonal protein that shows lupus anticoagulant activity.* HAEMA, 2005. **8**(4): p. 651-656.

1720. Fernandes, P., et al., *A 24-hour perioperative case study on argatroban use for left ventricle assist device insertion during cardiopulmonary bypass and veno-arterial extracorporeal membrane oxygenation.* Perfusion, 2019. **34**(4): p. 337-344.

1721. Fernandez, A.G., et al., *DIFFUSE ALVEOLAR HEMORRHAGE AND EXTRACORPOREAL MEMBRANE OXYGENATION (ECMO): a BLOODY CONUNDRUM.* Chest, 2019. **156**(4): p. A1911.

1722. Fernandez-Perez, E.R., E. Grabscheid, and N.S. Scheinfeld, *A case of systemic malignant atrophic papulosis (Kohlmeier-Degos' disease).* Journal of the National Medical Association, 2005. **97**(3): p. 421-425.

1723. Filippini, D., et al., *[Central nervous system involvement in patients with HCV-related cryoglobulinemia: literature review and a case report].* Reumatismo, 2002. **54**(2): p. 150-5.

1724. Finsterer, J. and A. Wilfing, *Anticoagulated de novo atrial flutter complicated by transitory ischemic attack in fatal COVID-19.* Clinical Case Reports, 2022. **10**(1): p. e05246.

1725. Fonseca, A., et al., *Rapidly progressive glomerulonephritis, thrombotic microangiopathy and amebic colitis: A challenging case report.* Pediatric Rheumatology, 2014. **12**(SUPPL. 1).

1726. Francisco, M.T., et al., *Therapeutic Plasma Exchange for Apixaban Removal in the Setting of Delayed Clearance and Life-Threatening Bleeding.* Blood, 2021. **138**(Supplement 1): p. 4271.

1727. Francisco, M.T., et al., *Relapsed refractory acquired thrombotic thrombocytopenic purpura (ATTP) following COVID-19 vaccination.* Blood, 2021. **138**(SUPPL 1): p. 4218.

1728. Francisco, M.T., et al., *Therapeutic plasma exchange for apixaban removal in the setting of delayed clearance and life-threatening bleeding.* Blood, 2021. **138**(SUPPL 1): p. 4271.

1729. Frank, M.G., C. Ladanyi, and P. Brittain, *Bugs, drugs, or immune system flubs, what causes limbic encephalitis? Moreover, what causes antin-methyl-d-aspartate receptor encephalitis?* Journal of General Internal Medicine, 2013. **28**(SUPPL. 1): p. S301.

1730. Fujii, H., *[A study of plasmapheresis in a case of primary macroglobulinemia associated with intraventricular hemorrhage].* Rinsho Ketsueki, 1984. **25**(9): p. 1466-72.

1731. Fujita, Y., et al., *[A case of an elderly SLE patient associated with acute renal failure].* Nihon Jinzo Gakkai Shi, 1993. **35**(10): p. 1201-4.

1732. Fukunari, K., et al., *A case of anti-neutrophil cytoplasmic antibody-associated vasculitis with anti-glomerular basement membrane antibodies that was successfully treated with mizoribine as a safe and effective remission maintenance therapy with prednisolone and plasma exchange.* CEN Case Reports, 2020. **9**(1): p. 42-47.

1733. Furmanczyk, A., et al., *The catastrophic antiphospholipid syndrome - A case report.* Lupus, 2010. **19**(4): p. 523.

1734. Gaddy, A.R. and M.S. Yaqub, *Antiphospholipid antibody syndrome causing thrombotic microangiopathy in the immediate post-transplant patient: A case report.* Journal of the American Society of Nephrology, 2020. **31**: p. 776.

1735. Gajkowski, E., et al., *Diffuse alveolar hemorrhage, and extracorporeal membrane oxygenation: A bloody conundrum.* ASAIO Journal, 2019. **65**(Supplement 2): p. 37.

1736. Galesic, K. and I. Ratkovic, *Acute transfusion reaction. A case report [5].* Nephron, 1997. **77**(1): p. 121.

1737. Gamba, G., et al., *Purpura fulminans as clinical manifestation of atypical SLE with antiphospholipid antibodies: a case report.* Haematologica, 1991. **76**(5): p. 426-8.

1738. Gandhi, R., et al., *Budd-chiari syndrome as a complication of newly-diagnosed catastrophic antiphospholipid syndrome despite anticoagulant therapy.* American Journal of Gastroenterology, 2021. **116**(SUPPL): p. S1168.

1739. Gangemi, A., S. Durgam, and P. Cristoforo Giulianotti, *Acute thrombotic thrombocytopenic purpura after sleeve gastrectomy: a case report and review of the literature.* Surgery for Obesity and Related Diseases, 2016. **12**(9): p. e80-e82.

1740. Gani, I., R. Kapoor, and M. Saeed, *Spontaneous breast haematoma after heparin anticoagulation.* European Journal of Case Reports in Internal Medicine, 2020. **7**(9).

1741. Garcia Rincon, C.I., et al., *Extensive skin necrosis secondary to antiphospholipid syndrome in an HIV infected patient: A case report.* Revista Colombiana de Reumatologia, 2014. **21**(3): p. 155-159.

1742. Gayam, V., et al., *A Rare Case of Acute Pancreatitis Due to Very Severe Hypertriglyceridemia (>10 000 mg/dL) Successfully Resolved With Insulin Therapy Alone: A Case Report and Literature Review.* Journal of Investigative Medicine High Impact Case Reports, 2018. **6**.

1743. Geethakumari, P.R., et al., *Complement inhibition with eculizumab for thrombotic microangiopathy rescues a living-donor kidney transplant in a patient with antiphospholipid antibody syndrome.* Transfus Apher Sci, 2017. **56**(3): p. 400-403.

1744. Georgiades, F., et al., *Takotsubo cardiomyopathy and thrombotic thrombocytopenic purpura preceding a lupus diagnosis: A case report.* Lupus, 2015. **24**(13): p. 1443-1447.

1745. Georgiou, G.K., et al., *Non-secreting benign glucagonoma diagnosed incidentally in a patient with refractory thrombocytopenic thrombotic purpura: report of a case.* Surgery Today, 2015. **45**(10): p. 1317-1320.

1746. Gerges, H. and A. Szkotak, *Acquired factor V inhibitor in a patient with mixed ischemic and dilated cardiomyopathy.* International Journal of Laboratory Hematology, 2017. **39**(Supplement 2): p. 37.

1747. Gharib, N., *MULTIORGAN THROMBOSIS TRIGGERED BY CEFAZOLIN IN CONTEXT OF ACITRETIN USE.* Chest, 2019. **156**(4 Supplement): p. A2156.

1748. Ghazarian, Z., et al., *Therapeutic plasma exchange in the management of a patient with hyperlipidemic pancreatitis.* Chest, 2016. **150**(4 Supplement 1): p. 246A.

1749. Gheevarghese John, S., et al., *Acute hypertriglyceridemic pancreatitis: Rapid recovery with therapeutic apheresis.* American Journal of Respiratory and Critical Care Medicine, 2018. **197**(MeetingAbstracts).

1750. Gheith, Z., A. Abulsoaud, and K. Satish, *A rare case of thrombotic thrombocytopenic purpura presenting as non-ST elevation myocardial infarction.* American Journal of Respiratory and Critical Care Medicine, 2018. **197**(MeetingAbstracts).

1751. Gherman, R.B., J. Tramont, and D.J. Connito, *Postpartum hemolytic-uremic syndrome associated with lupus anticoagulant. A case report.* J Reprod Med, 1999. **44**(5): p. 471-4.

1752. Gkalea, V., et al., *HEPARIN-INDUCED THROMBOCYTOPENIA ANTIBODIES CAN MAINTAIN PLATELET-ACTIVATING PROPERTIES AFTER TWO SESSIONS OF THERAPEUTIC PLASMA EXCHANGE.* HemaSphere, 2022. **6**(Supplement 3): p. 3960-3961.

1753. Goda, S., S. Gando, and B.W. Berg, *Veno-venous extracorporeal membrane oxygenation (VV-ECMO) for life-threatening isolated pulmonary anti-GBM disease.* Respir Med Case Rep, 2022. **38**: p. 101680.

1754. Goddard, I.R., et al., *Plasma exchange as a treatment for endogenous glycosaminoglycan anticoagulant induced haemorrhage in a patient with myeloma kidney.* Nephron, 1990. **56**(1): p. 94-6.

1755. Goel, M.K., et al., *A Case of Refractory Hypoxemic Respiratory Failure due to Antineutrophil Cytoplasmic Antibodies-associated Diffuse Alveolar Hemorrhage Rescued by Extracorporeal Membrane Oxygenation.* Indian J Crit Care Med, 2020. **24**(9): p. 879-881.

1756. Goel, M.K., et al., *A case of refractory hypoxemic respiratory failure due to anti-neutrophil cytoplasmic antibodies-associated diffuse alveolar hemorrhage rescued by extracorporeal membrane oxygenation.* Indian Journal of Critical Care Medicine, 2020. **24**(9): p. 879-881.

1757. Golse, M., et al., *Case 294: Catastrophic Antiphospholipid Syndrome.* Radiology, 2021. **301**(1): p. 242-246.

1758. Gomathy, S.B., et al., *Enoxaparin-induced Wunderlich syndrome in a young patient with anti-GAD 65-associated opsoclonus and limbic encephalitis: a rare complication in a rare disease.* BMJ Case Rep, 2021. **14**(10).

1759. Gong, S., et al., *Cerebral venous sinus thrombosis caused by traumatic brain injury complicating thyroid storm: a case report and discussion.* BMC Neurology, 2022. **22**(1): p. 248.

1760. Gonzalez, A., J. Alperin, and S. Yates, *The utility of therapeutic plasma exchange in the setting of hyperviscosity syndrome associated with juvenile rheumatoid arthritis: A case report.* Journal of Clinical Apheresis, 2020. **35**(6): p. 556-557.

1761. Grant, M.L., et al., *Complete heart block in thrombotic microangiopathy syndrome improved after plasma exchange.* Journal of Clinical Apheresis, 2015. **30**(2): p. 107-108.

1762. Griffith, K.E., et al., *Long-term use of the CentriMag Ventricular Assist System as a right ventricular assist device: A case report.* Perfusion, 2012. **27**(1): p. 65-70.

1763. Griggs, J., E. Lockhart, and K. Crookston, *Using antithrombin activity for plasma titration during therapeutic plasma exchange in the setting of heparin therapy: A case report.* American Journal of Clinical Pathology, 2018. **149**(Supplement 1): p. S197.

1764. Grill, F., et al., *Clinical microbiological case: A necrotic skin lesion in a patient with renal failure.* Clinical Microbiology and Infection, 2003. **9**(6): p. 538-539.

1765. Grinstein, J., E.J. Molina, and F.H. Sheikh, *Successful HeartMate 3 LVAD implantation in a patient with active heparin-induced thrombocytopaenia with thrombosis using pre-implantation plasmapheresis and intraoperative continuous prostacyclin.* Interact Cardiovasc Thorac Surg, 2021. **33**(1): p. 161-162.

1766. Guillot, M., et al., *Eculizumab for catastrophic antiphospholipid syndrome - A case report and literature review.* Rheumatology (United Kingdom), 2018. **57**(11): p. 2055-2057.

1767. Gulati, G., et al., *Cold hemagglutinin hemolysis and hypercoagulable state due to mycoplasma pneumoniae.* Blood, 2009. **114**(22).

1768. Gunther, A., et al., *Complicated long term vaccine induced thrombotic immune thrombocytopenia-a case report.* Vaccines, 2021. **9**(11): p. 1344.

1769. Günther, A., et al., *Complicated Long Term Vaccine Induced Thrombotic Immune Thrombocytopenia-A Case Report.* Vaccines (Basel), 2021. **9**(11).

1770. Guntz, J., N. Layios, and P. Damas, *Catastrophic antiphospholipid syndrome: Case reports and review of the literature.* Acta Anaesthesiologica Belgica, 2014. **65**(3): p. 87-94.

1771. Guo, Z., et al., *Extracorporeal membrane oxygenation for the management of respiratory failure caused by diffuse alveolar hemorrhage.* Journal of Extra-Corporeal Technology, 2009. **41**(1): p. 37-40.

1772. Gupta, A. and L. Zakko, *Catastrophic antiphospholipid syndrome in a patient with lung malignancy.* Journal of General Internal Medicine, 2014. **29**(SUPPL. 1): p. S330.

1773. Gupta, L., et al., *Pursuit of diagnosis and wine in California-When a DVT is more than a blood clot!* Chest, 2013. **144**(4 MEETING ABSTRACT).

1774. Gupta, M., et al., *Eculizumab as salvage therapy in a patient with APLS undergoing renal transplantation.* Journal of the American Society of Nephrology, 2018. **29**: p. 1202.

1775. Hachey, B., et al., *Vision loss: A unique presentation of sle with concomitant catastrophic antiphospholipid syndrome (CAPS).* Journal of General Internal Medicine, 2015. **30**(SUPPL. 2): p. S322-S323.

1776. Hakeem, I., *Pauci-immune crescentic GN in a patient with catastrophic anti-phospholipid antibody syndrome.* Blood Purification, 2019. **47**(1-3): p. 292-293.

1777. Hama Amin, B.J., et al., *Post COVID-19 hemorrhagic pericardial effusion; A case report with literature review.* Annals of Medicine and Surgery, 2022. **74**: p. 103300.

1778. Hamed, E., et al., *A CASE OF CMV-INDUCED COLLAPSING FSGS IN IMMUNOCOMPETENT PATIENT WITH RECOVERING KIDNEY FUNCTION.* American Journal of Kidney Diseases, 2020. **75**(4): p. 567-568.

1779. Hanafusa, N., N. Aozasa, and T. Fujita, *A patient whose factor xiii level was decreased by double filtrate plasmapheresis and successfully recovered by infusion of factor xiii concentrate.* Therapeutic Apheresis and Dialysis, 2010. **14**(4): p. 432-433.

1780. Hansen, T., et al., *Acute renal failure, systemic lupus erythematosus and thrombotic microangiopathy following treatment with beta-interferon for multiple sclerosis: Case report and review of the literature.* NDT Plus, 2009. **2**(6): p. 466-468.

1781. Haque, W., et al., *Osteonecrosis secondary to antiphospholipid syndrome: a case report, review of the literature, and treatment strategy.* Rheumatol Int, 2010. **30**(6): p. 719-23.

1782. Hasan, B. and T. Asif, *Catastrophic antiphospholipid syndrome - A rare but life threatening condition.* Journal of Investigative Medicine, 2017. **65**(4): p. 876.

1783. Hasan, H., et al., *Corticosteroids, Plasmapheresis, Argatroban, Rituximab, and Sirolimus Provided Clinical Benefit for Catastrophic Antiphospholipid Syndrome in a Patient with a History of Heparin-Induced Thrombocytopenia.* Case Rep Rheumatol, 2023. **2023**: p. 3226278.

1784. Hasan, M., C. Natha, and R. Chandra, *Antiphospholipid antibody syndrome: an atypical presentation with a potentially delayed diagnosis.* American Journal of the Medical Sciences, 2023. **365**(Supplement 1): p. S42-S43.

1785. Hayano, K., et al., *[A case of anti-GBM nephritis (crescentic glomerulonephritis) associated with membranous nephropathy].* Nihon Jinzo Gakkai Shi, 1992. **34**(7): p. 821-6.

1786. Hayrabedian, M. and R. Sreedhar, *Disseminated intravascular coagulation (dic) as a predecessor of thromboinflammation and multiorgan organ failure in a patient with coronavirus disease 2019 (COVID-19), a case report.* American Journal of Respiratory and Critical Care Medicine, 2021. **203**(9).

1787. He, J., C. Ma, and F. Wang, *Segmental citrate anticoagulation for double-filtration plasmapheresis: A case report and literature review.* Med Int (Lond), 2022. **2**(3): p. 18.

1788. Hegerova, L., et al., *A rapidly progressive case of hemorrhagic pancreatitis in a patient with polycythemia vera, or not?* American Journal of Gastroenterology, 2012. **107**(SUPPL. 1): p. S338.

1789. Hensch, L., et al., *Clinical description and laboratory characterization of heparin-like substance causing bleeding.* Research and Practice in Thrombosis and Haemostasis, 2018. **2**(Supplement 1): p. 101.

1790. Herrmann, M., et al., *[Severe, non-infectious mitral valve endocarditis after mitral valve reconstruction in a 32-year old female with primary antiphospholipid syndrome].* Z Kardiol, 2004. **93**(7): p. 546-54.

1791. Hickstein, H., et al., *Protein A immunoadsorption in a pregnant women with habitual abortion.* Transfusion and Apheresis Science, 2002. **27**(3): p. 259-261.

1792. Hindi, Z., et al., *The role of hydroxychloroquine in catastrophic antiphospholipid syndrome case: Series of two case reports and review of literature.* SAGE Open Medical Case Reports, 2018. **6**.

1793. Hirakawa, E., et al., *A case of catastrophic antiphospholipid antibody syndrome complicated with systemic lupus erythematosus, double positive for anti-cardiolipin/β₂ glycoprotein I and anti-phosphatidylserine/prothrombin autoantibodies.* Mod Rheumatol, 2012. **22**(5): p. 769-73.

1794. Ho, A. and S. Koenig, *Diagnostic dilemma: Multiorgan failure due to catastrophic antiphospholipid syndrome in a young woman with six healthy children.* Chest, 2017. **152**(4 Supplement 1): p. A278.

1795. Hofmann, G., et al., *Plasmapheresis reverses all side-effects of a cisplatin overdose - A case report and treatment recommendation.* BMC Cancer, 2006. **6**: p. 1.

1796. Hohenforst-Schmidt, W., et al., *Successful application of extracorporeal membrane oxygenation due to pulmonary hemorrhage secondary to granulomatosis with polyangiitis.* Drug Design, Development and Therapy, 2013. **7**: p. 627-633.

1797. Holahan, B., et al., *CONCURRENT CATASTROPHIC ANTIPHOSPHOLIPID SYNDROME AND HEPARIN-INDUCED THROMBOCYTOPENIA.* Chest, 2019. **156**(4 Supplement): p. A90-A91.

1798. Horimoto, S., et al., *Off-pump coronary artery bypass in a patient with the antiphospholipid syndrome.* J Cardiovasc Surg (Torino), 2005. **46**(1): p. 81-3.

1799. Howell, L., A. Elson, and C. Dunn, *Two's company but three's a crowd.* Journal of General Internal Medicine, 2011. **26**(SUPPL. 1): p. S403.

1800. Hsia, S.H., P.W. Connelly, and R.A. Hegele, *Successful outcome in severe pregnancy-associated hyperlipemia: a case report and literature review.* Am J Med Sci, 1995. **309**(4): p. 213-8.

1801. Hu, K., A. Agarwal, and A. Chopra, *A CASE OF CATASTROPHIC ANTIPHOSPHOLIPID SYNDROME (APS) PRESENTING WITH ADRENAL HEMORRHAGE.* Chest, 2019. **156**(4 Supplement): p. A2056.

1802. Hua, J., et al., *Successful treatment of severe guillain-barre syndrome in an older woman.* Journal of the American Geriatrics Society, 2014. **62**(SUPPL. 1): p. S31-S32.

1803. Huang, D.F., S.T. Tsai, and S.R. Wang, *Recovery of both acute massive pulmonary hemorrhage and acute renal failure in a systemic lupus erythematosus patient with lupus anticoagulant by the combined therapy of plasmapheresis plus cyclophosphamide.* Transfus Sci, 1994. **15**(3): p. 283-8.

1804. Hucker, W.J., et al., *Myocardial catastrophe: A case of severe, sudden systolic dysfunction.* Circulation, 2013. **128**(22 SUPPL. 1).

1805. Hucker, W.J., et al., *Myocardial catastrophe: a case of sudden, severe myocardial dysfunction.* Circulation, 2014. **130**(10): p. 854-62.

1806. Hunt, E.A.K. and D.R. Stein, *Pheresis during retransplantation of a patient with elevated lipoprotein(a) and first renal transplant loss due to thrombosis.* Blood Purification, 2013. **35**(1-3): p. 160.

1807. Huq Ronny, F.M., J. Karp, and A. Peedin, *Therapeutic plasma exchange for the treatment of autoimmune cerebellar ataxia with anti-gad antibodies: A case report.* Journal of Clinical Apheresis, 2018. **33**(2): p. 192-193.

1808. Huraib, S., *Right atrial thrombus as a complication of subclavian vein catheterization - A case report.* Angiology, 1992. **43**(5): p. 439-442.

1809. Hussain, H., et al., *Eculizumab treatment for refractory thrombosis in antiphospholipid syndrome.* Research and Practice in Thrombosis and Haemostasis, 2021. **5**(SUPPL 1).

1810. Hussain, H., et al., *Eculizumab for refractory thrombosis in antiphospholipid syndrome.* Blood, 2020. **136**(SUPPL 1): p. 10-11.

1811. Hyder, S., et al., *Clotting crisis: A rare case of catastrophic anti-phospholipid syndrome (CAPS) with multiorgan failure.* American Journal of Respiratory and Critical Care Medicine, 2021. **203**(9).

1812. Igneri, L.A., Q.A. Czosnowski, and C.B. Whitman, *Methylprednisolone sodium succinate-associated macroglossia in a critically Ill patient.* Pharmacotherapy, 2013. **33**(2): p. e14-e18.

1813. Ijaz, M., N. Abbas, and D. Lvovsky, *Severe Uncompensated Metabolic Alkalosis due to Plasma Exchange in a Patient with Pulmonary-Renal Syndrome: A Clinician's Challenge.* Case Rep Crit Care, 2015. **2015**: p. 802186.

1814. Ijaz, M., N. Abbas, and D. Lvovsky, *Severe uncompensated metabolic alkalosis following treatment of double positive goodpasture's syndrome: A challenge for the clinician.* American Journal of Respiratory and Critical Care Medicine, 2015. **191**(MeetingAbstracts).

1815. Iluonakhamhe, E. and A. Zakaria, *Plasma exchange effective in acute management of heparin induced thrombocytopenia in setting of acute inter-cerebral hemorrhage.* Neurocritical Care, 2013. **19**(1 SUPPL. 1): p. S294.

1816. Imtiaz, M.A., et al., *Catastrophic antiphospholipid syndrome in a patient with upper gastrointestinal bleed, portal hypertension, deep venous thrombosis, and cardiogenic shock.* Chest, 2014. **146**(4 MEETING ABSTRACT).

1817. Ioannis, M.P.I.P. and E.N. Evagoras Nikolaides, *Thrombotic thrombopenic purpura in a patient with Takotsubo cardiomyopathy: A case report.* European Journal of Heart Failure, 2014. **16**(SUPPL. 2): p. 232-233.

1818. Iseki, K., et al., *[A case of ABO blood group incompatibility treated by exchange transfusion].* Masui, 1998. **47**(2): p. 225-9.

1819. Ivanovic, J., et al., *D-dimer elevation after first alemtuzumab administration in a multiple sclerosis patient: case report.* Acta Neurologica Belgica, 2023. **123**(1): p. 275-277.

1820. Iwashima, S., T. Ishikawa, and T. Ohzeki, *Brain natriuretic peptide levels in Kawasaki disease: A case report.* Pediatrics International, 2009. **51**(3): p. 415-418.

1821. Izumi, Y., et al., *Enterohemorrhagic Escherichia coli O157 infection in an elderly patient with secondary hemolytic uremic syndrome who developed recurrent acute exacerbation of chronic cholecystitis.* Japanese Journal of Geriatrics, 1998. **35**(7): p. 559-565.

1822. Jaben, E.A., et al., *Use of plasma exchange in patients with heparin-induced thrombocytopenia: A report of two cases and a review of the literature.* Journal of Clinical Apheresis.

1823. Jabr, F.I., et al., *Thrombotic thrombocytopenic purpura in a patient with Behçet's disease.* Arthritis Rheum, 2003. **48**(5): p. 1468-9; author reply 1469.

1824. Jacobsen, S.E., P. Petersen, and P. Jensen, *Acute abdomen in rheumatoid arthritis due to mesenteric arteritis. A case report and review.* Dan Med Bull, 1985. **32**(3): p. 191-3.

1825. Janssen, S., et al., *Abdominal wall phlebitis due to Prevotella bivia following renal transplantation in a patient with an occluded inferior vena cava.* Infection, 2013. **41**(1): p. 271-274.

1826. Java, A., et al., *Familial pregnancy-associated ahus and acute heart failure successfully treated with eculizumab.* Journal of the American Society of Nephrology, 2019. **30**: p. 328-329.

1827. Jiao, L.P., et al., *Plasma exchange in Goodpasture syndrome associated with Turner's syndrome: A case report.* African Health Sciences, 2012. **12**(4): p. 572-575.

1828. Jimoh, R.O., I. Ali, and L.M. Belalcazar, *Abstract #1183744: The challenge of unresolving chylomicronemia and recurring pancreatitis: Lessons learned on the treatment of severe hypertriglyceridemia.* Endocrine Practice, 2022. **28**(5 Supplement): p. S63-S64.

1829. Jin, S. and P. Jha, *A case of purpura fulminans (PF) in a background of septic shock caused by streptococcus pneumoniae.* Journal of General Internal Medicine, 2016. **31**(2 SUPPL. 1): p. S494-S495.

1830. John, S. and K. McCrae, *Catastrophic antiphospholipid syndrome in a patient with immune thrombocytopenic purpura.* UHOD - Uluslararasi Hematoloji-Onkoloji Dergisi, 2011. **21**(3): p. 200-202.

1831. Jones, B., et al., *De novo crescentic glomerulonephritis in a renal transplant.* Am J Kidney Dis, 1990. **16**(5): p. 501-3.

1832. Joshi, U., et al., *Clot crisis: A case of bilateral central retinal artery occlusion from catastrophic antiphospholipid syndrome.* Journal of Hospital Medicine, 2018. **13**(4 Supplement 1).

1833. Kaedbey, R., et al., *Therapeutic plasma exchange using heparin as the anticoagulant in a patient with citrate induced igm agglutination.* Journal of Clinical Apheresis, 2021. **36**(2): p. 245-246.

1834. Kajtazi, N.I., et al., *Chronic inflammatory demyelinating polyneuropathy evolving to primary CNS lymphoma.* BMJ Case Reports, 2021. **14**(11): p. e244767.

1835. Kalinova, D. and R. Rashkov, *Coincidence of Guillain-Barre syndrome presenting with Landry's acute flaccid paralysis and transverse myelitis.* Reumatologia, 2019. **57**(2): p. 120-122.

1836. Kandah, E., et al., *A Novel Case of Cytomegalovirus Pneumonia in an Acquired Thrombotic Thrombocytopenic Purpura Patient Treated With Rituximab.* Cureus, 2021. **13**(3): p. e14182.

1837. Kanduri, S.R., et al., *Multiple Myeloma, Hyperviscosity, Hemodialysis Filter Clogging, and Antigen Excess Artifact: A Case Report.* Kidney Medicine, 2021. **3**(4): p. 649-652.

1838. Kansagra, A., et al., *Severe metabolic alkalosis induced by daily plasmapheresis in a patient with thrombotic thrombocytopenic purpura.* Critical Care Medicine, 2010. **38**(SUPPL. 12): p. A278.

1839. Kant, R., et al., *Inhibition of thrombin action improves insulin sensitivity in an obese patient with type 2 diabetes.* Endocrine Reviews, 2013. **34**(3 SUPPL. 1).

1840. Karathanasis, D., et al., *Ischemic stroke as the first clinical manifestation of thrombotic thrombocytopenic purpura (TTP) in a pregnant woman.* International Journal of Stroke, 2020. **15**(1 SUPPL): p. 712-713.

1841. Karim, M., et al., *Renal failure due to scleroderma with thrombotic microangiopathy developing in a woman treated with carboplatin for ovarian cancer.* Clinical Nephrology, 2002. **58**(5): p. 384-388.

1842. Katabi, A., et al., *Nasal myiasis in myasthenic crisis, a case report and literature review.* Respiratory Medicine Case Reports, 2020. **31**: p. 101212.

1843. Kattwinkel, N., et al., *Myocardial infarction caused by cardiac microvasculopathy in a patient with the primary antiphospholipid syndrome.* Ann Intern Med, 1992. **116**(12 Pt 1): p. 974-6.

1844. Kauke, M., et al., *Full facial retransplantation in a female patient-Technical, immunologic, and clinical considerations.* American Journal of Transplantation, 2021. **21**(10): p. 3472-3480.

1845. Kawaguchi, T., et al., *A new therapeutic strategy for streptococcal toxic shock syndrome: A key target for cytokines.* Internal Medicine, 2003. **42**(2): p. 211-218.

1846. Kears, A., et al., *RASH PROVOKES CATASTROPHIC ANTIPHOSPHOLID SYNDROME.* Chest, 2019. **156**(4 Supplement): p. A2067.

1847. Kefas, J., et al., *Small vessel vasculitis and dry gangrene secondary to combined CTLA-4 and PD-1 blockade in malignant mesothelioma.* BMC Rheumatology, 2022. **6**(1): p. 10.

1848. Kelly, D., D. Makkuni, and D. Ail, *Rare cause of respiratory failure in a young woman: isolated diffuse alveolar haemorrhage requiring extracorporeal membrane oxygenation.* BMJ Case Rep, 2017. **2017**.

1849. Kerr, M., et al., *NHSBT's favourable experience treating 5 patients with Acute Liver Failure (ALF) secondary to paracetamol overdose (POD) in Leeds Teaching Hospitals Trust.* Transfusion Medicine, 2018. **28**(Supplement 1): p. 58.

1850. Kew, G.S., J. Cho, and A. Lateef, *Microangiopathic antiphospholipid antibody-associated syndrome in a pregnant lady.* Lupus, 2017. **26**(4): p. 435-437.

1851. Khaleq, K., et al., *Guillain-Barre syndrome and pregnancy. About a case.* Cahiers d'Anesthesiologie, 2003. **51**(3): p. 195-197.

1852. Khan, A.H. and E. Khan, *Bleeding in a broken heart: A rare case of immune thrombocytopenia and takotsubo cardiomyopathy.* Journal of General Internal Medicine, 2017. **32**(2 Supplement 1): p. S458.

1853. Khan, F.G. and S. Namran, *Coexistence of myasthenia gravis with hypokalemic periodic paralysis: A rare presentation.* BMJ Case Reports, 2019. **12**(10): p. e231241.

1854. Khan, J., Z. Dhanani, and C. Woods, *AN UNUSUAL CASE OF THROMBOCYTOPENIA UNRESPONSIVE TO TRANSFUSION.* Chest, 2020. **158**(4 Supplement): p. A1030.

1855. Khan, S., et al., *Minimizing the risk of perioperative cardiovascular complications in homozygous familial hypercholesterolemia: a case report.* J Dent Anesth Pain Med, 2020. **20**(1): p. 39-44.

1856. Khattar, R., et al., *AUTOIMMUNE DIFFUSE ALVEOLAR HEMORRHAGE TREATED WITH CYCLOPHOSPHAMIDE WHILE ON EXTRACORPOREAL MEMBRANE OXYGENATION.* Chest, 2020. **158**(4 Supplement): p. A738-A739.

1857. Khedr, E.M., A.A. Karim, and R.K. Soliman, *Case Report: Acute Spinal Cord Myelopathy in Patients With COVID-19.* Frontiers in Neurology, 2020. **11**: p. 610648.

1858. Khetarpal, A., V. Gupta, and U. Kotwal, *Efficacy of cascade plasmapheresis in refractory familial hypercholesterolemia-a case report.* Vox Sanguinis, 2019. **114**(Supplement 1): p. 212.

1859. Kiani, R., et al., *Central Nervous System and Cardiac Involvement in the Hypereosinophilic Syndrome: A Case Report.* Immunological Investigations, 2021. **50**(4): p. 356-362.

1860. Kim, A. and D. Kotok, *CATASTROPHIC ANTIPHOSPHOLIPID SYNDROME: A UNIQUE CAUSE OF ARDS.* Chest, 2021. **160**(4 Supplement): p. A622.

1861. Kim, D.H., et al., *Successful plasma exchange combined with unfractionated heparin anticoagulation in a patient exhibiting anaphylaxis to acid-citrate-dextrose formula A.* Transfusion, 2018. **58**(Supplement 2): p. 100A.

1862. Kisioglu, M., et al., *Successful therapeutic plasma exchange in a case with extremely severe hypertriglyceridemia secondary to diabetic ketoacidosis concomitant with type IX glycogen storage disease.* Transfusion and Apheresis Science, 2022. **61**(1): p. 103289.

1863. Kits, A., et al., *Fatal Acute Hemorrhagic Encephalomyelitis and Antiphospholipid Antibodies following SARS-CoV-2 Vaccination: A Case Report.* Vaccines, 2022. **10**(12): p. 2046.

1864. Kleffner, I., et al., *An enigmatic case of acute mercury poisoning: Clinical, immunological findings and platelet function.* Frontiers in Neurology, 2017. **8**(SEP): p. 517.

1865. Kniaz, D., et al., *Postpartum hemolytic uremic syndrome associated with antiphospholipid antibodies. A case report and review of the literature.* Am J Nephrol, 1992. **12**(1-2): p. 126-33.

1866. Knower, M.T., et al., *Quinine-induced disseminated intravascular coagulation: Case report and review of the literature.* Intensive Care Medicine, 2003. **29**(6): p. 1007-1011.

1867. Ko, Y.M., et al., *A fatal case of acute pulmonary embolism caused by right ventricular masses of acute lymphoblastic lymphoma-leukemia in a 13 year old girl.* Korean J Pediatr, 2012. **55**(7): p. 249-53.

1868. Koenen, M. and A.C. Djadoenath, *[Susac's syndrome: psychosis as a presenting symptom of rare angiopathy].* Ned Tijdschr Geneeskd, 2015. **159**: p. A8237.

1869. Koga, T., et al., *Renal thrombotic microangiopathies/thrombotic thrombocytopenic purpura in a patient with primary Sjogren's syndrome complicated with IgM monoclonal gammopathy of undetermined significance.* Rheumatology International, 2013. **33**(1): p. 227-230.

1870. Koh, H., et al., *Plasma exchange and early thyroidectomy in thyroid storm requiring extracorporeal membrane oxygenation.* Endocrinology, Diabetes and Metabolism Case Reports, 2019. **2019**(1): p. 19-0051.

1871. Kojima, S., et al., *Effects of losartan on blood pressure and humoral factors in a patient who suffered from anaphylactoid reactions when treated with ACE inhibitors during LDL apheresis.* Hypertens Res, 2001. **24**(5): p. 595-8.

1872. Kojima, Y., et al., *Early manifestation of thrombotic thrombocytopenic purpura during the hospitalization of a patient with a hemorrhagic stroke.* American Journal of Clinical Pathology, 2014. **142**(SUPPL. 1): p. A011.

1873. Kolandra, L., et al., *Autoimmune disease and multiorgan failure: A case of catastrophic antiphospholipid antibody syndrome.* Critical Care Medicine, 2019. **47**(1 Supplement 1).

1874. Kolbe, K., A. Papaila, and S. Freeman, *Management of digital auto-amputation in tricuspid valve endocarditis in a non-surgical candidate.* Journal of General Internal Medicine, 2021. **36**(SUPPL 1): p. S244-S245.

1875. Kolman, L., C. Kim, and D. Patel, *Here a clot, there a clot, everywhere a clot clot.* Journal of General Internal Medicine, 2010. **25**(SUPPL. 3): p. S517.

1876. Komarnicka, J., et al., *Computed tomography (CT) angiography in pre- embolization assessment of location of gastrointestinal bleeding in paediatric patient with granulomatosis with polyangiitis (Wegener's Granulomatosis) - Case report.* Polish Journal of Radiology, 2017. **82**: p. 589-592.

1877. Kon, Z.N., et al., *Venovenous extracorporeal membrane oxygenation as a bridge to lung transplantation: Successful transplantation after 155 days of support.* Annals of Thoracic Surgery, 2015. **99**(2): p. 704-707.

1878. Konca, C., et al., *The First Case of Multisystem Inflammatory Syndrome in Children Successfully Treated with Combined Therapies Including Extracorporeal Membrane Oxygenation and Plasmapheresis.* Journal of Pediatric Infectious Diseases, 2022. **17**(1): p. 53-58.

1879. Kosaraju, N., et al., *Adult purpura fulminans associated with non-steroidal anti-inflammatory drug use.* Journal of Postgraduate Medicine, 2011. **57**(2): p. 145-146.

1880. Koschmieder, S., et al., *Combined plasmapheresis and immunosuppression as rescue treatment of a patient with catastrophic antiphospholipid syndrome occurring despite anticoagulation: a case report.* Blood Coagul Fibrinolysis, 2003. **14**(4): p. 395-9.

1881. Koštál, M., et al., *Beneficial effect of plasma exchange in the treatment of toxic epidermal necrolysis: a series of four cases.* J Clin Apher, 2012. **27**(4): p. 215-20.

1882. Krajewski, A., M.J. Mazurek, and E. Mlynska-Krajewska, *Successful therapy of recurrent toxic epidermal necrolysis using total plasma exchange, continuous venovenous hemodiafiltration, and intravenous immunoglobulin-Case report.* Dermatologic Therapy, 2020. **33**(4): p. e13442.

1883. Kramer, R., et al., *Heparin-induced thrombocytopenia with thrombosis syndrome managed with plasmapheresis.* Interact Cardiovasc Thorac Surg, 2009. **8**(4): p. 439-41.

1884. Kramer, R., et al., *Proposal for bail-out procedures - Cardiac general heparin-induced thrombocytopenia with thrombosis syndrome managed with plasmapheresis.* Interactive Cardiovascular and Thoracic Surgery, 2009. **8**(4): p. 439-441.

1885. Krishnamoorthy, V., et al., *All that scatters is not heart: A case of ttp presenting as multi-territorial stroke.* Romanian Journal of Neurology/ Revista Romana de Neurologie, 2020. **19**(4): p. 292-294.

1886. Krol, J.J., et al., *Case 213: primary splenic angiosarcoma.* Radiology, 2015. **274**(1): p. 298-303.

1887. Kumar, D., et al., *A CASE OF RECURRENT PANCREATITIS DUE TO HYPERTRIGLYCERIDEMIA.* Chest, 2021. **160**(4 Supplement): p. A851.

1888. Kumar, V., et al., *Stop the bleeding! A novel approach to reversing factorxa inhibitors using plasma exchange.* Journal of General Internal Medicine, 2016. **31**(2 SUPPL. 1): p. S735.

1889. Kumari, S., et al., *Efficacy of therapeutic plasma exchange in a patient with coagulation inhibitors (acquired haemophilia A) - A case report.* Transfus Apher Sci, 2020. **59**(4): p. 102809.

1890. Kurnik, B.R., F. Singer, and W.C. Groh, *Case report: dextran-induced acute anuric renal failure.* Am J Med Sci, 1991. **302**(1): p. 28-30.

1891. Kwak, S. and M. Green, *Rare yet catastrophic presentation of undiagnosed antiphospholipid syndrome.* BMJ Case Reports, 2022. **15**(1): p. e245838.

1892. La Mura, V., et al., *Acute liver necrosis in a SARS-CoV-2 positive patient with triple positive antiphospholipid syndrome.* Research and Practice in Thrombosis and Haemostasis, 2021. **5**(SUPPL 2).

1893. Lai, S., D.H. Walker, and M.T. Elghetany, *Catastrophic antiphospholipid syndrome: a rare cause of disseminated microvascular thrombotic injury - a case report with pathological and molecular correlative studies.* Pathol Int, 2005. **55**(3): p. 144-9.

1894. Lam, M., *A young woman presenting with pancytopenia and ischemic stroke.* Journal of General Internal Medicine, 2019. **34**(2 Supplement): p. S462.

1895. Lam, W.W., M.A. Reyes, and J.J. Seger, *Plasma Exchange for Urgent Apixaban Reversal in a Case of Hemorrhagic Tamponade after Pacemaker Implantation.* Tex Heart Inst J, 2015. **42**(4): p. 377-80.

1896. LaMoreaux, B., et al., *Two cases of thrombosis in patients with antiphospholipid antibodies during treatment of immune thrombocytopenia with romiplostim, a thrombopoietin receptor agonist.* Seminars in Arthritis and Rheumatism, 2016. **45**(4): p. e10-e12.

1897. Larakeb, A.S., et al., *Acute renal cortical necrosis due to acquired antiprotein S antibodies.* Pediatric Nephrology, 2009. **24**(1): p. 207-209.

1898. Larrubia, A.F.G., et al., *Unexpected side effects without hypocalcemia during therapeutic plasma exchange.* Vox Sanguinis, 2011. **101**(SUPPL. 1): p. 297-298.

1899. Leon-Sanchez, A.R. and A. Escobar-Naranjo, *Hemophagocytic lymphohystiocytosis in miliary tuberculosis.* American Journal of Respiratory and Critical Care Medicine, 2010. **181**(1 MeetingAbstracts).

1900. Leung, S., et al., *Isolation and purification of anticardiolipin antibody from plasma of a patient with antiphospholipid syndrome: induced generation of platelet thromboxane A2 synthesis.* Prostaglandins Leukot Essent Fatty Acids, 1996. **55**(6): p. 385-93.

1901. Levai, T., Z.S. Marjanek, and D. Brenner, *Supportive therapy of acute pancreatitis with CytoSorb Adsorber. Case report.* Infection, 2019. **47**(Supplement 1): p. S49-S50.

1902. Levine, M. and D.F.M. Brown, *Succinylcholine-induced hyperkalemia in a patient with multiple sclerosis.* Journal of Emergency Medicine, 2012. **43**(2): p. 279-282.

1903. Lew, S.Q. and J.A. Watson, *Urea and creatinine generation and removal in a pregnant patient receiving peritoneal dialysis.* Adv Perit Dial, 1992. **8**: p. 131-5.

1904. Lewandowska, M.D., A. Cieciuch, and O. Kozyreva, *Cancer related microangiopathic hemolytic anemia (CR-MAHA) unresponsive to chemotherapy: A rare case.* American Journal of Respiratory and Critical Care Medicine, 2014. **189**(MeetingAbstracts).

1905. Li, C.Z., et al., *Fatal antiphospholipid syndrome following endoscopic transnasal-transsphenoidal surgery for a pituitary tumor: A case report.* Medicine (Baltimore), 2017. **96**(1): p. e5774.

1906. Li, Y., et al., *Therapeutic plasma exchange with 5% albumin replacement in a patient with catastrophic antiphospholipid syndrome on fondaparinux.* Vox Sanguinis, 2016. **111**(Supplement 1): p. 252-253.

1907. Liao, J.L., et al., *A 28-Year-Old Woman Presenting with a Clinical Flare of Systematic Lupus Erythematosus and Abdominal Pain Due to Rectus Sheath Hematoma.* American Journal of Case Reports, 2022. **23**: p. e935472.

1908. Liew, J., et al., *Catastrophic antiphospholipid syndrome in a patient with systemic sclerosis and hereditary angioedema: case report and literature review.* Modern Rheumatology Case Reports, 2018. **2**(1): p. 33-38.

1909. Lim, W., et al., *A Difficult and Rare Case of Warfarin Refractory Antiphospholipid Syndrome Presenting With Catastrophic Antiphospholipid Syndrome Complicated by Gastrointestinal Bleeding.* Cureus, 2021. **13**(8): p. e17106.

1910. Lin, C., D. Li, and B. Hu, *Lower Limb Necrosis Secondary to Purpura Fulminans: A Case Report.* J Burn Care Res, 2023. **44**(2): p. 477-480.

1911. Lin, J., et al., *Thrombosis in the portal venous system caused by hypereosinophilic syndrome: A case report.* Medicine (Baltimore), 2018. **97**(48): p. e13425.

1912. Linder, G.E., et al., *Use of extracorporeal photopheresis for treatment of antibody-mediated heart transplant rejection: A pediatric case report.* Journal of Clinical Apheresis, 2021. **36**(2): p. 265-266.

1913. Ling, L., S.M. Bagshaw, and P.M. Villeneuve, *Guillain-Barre syndrome after SARS-CoV-2 vaccination in a patient with previous vaccine-associated Guillain-Barre syndrome.* CMAJ, 2021. **193**(46): p. E1766-E1769.

1914. Liu, L.L., et al., *A case report of successful treatment with plasma exchange for adult-onset still's disease with autoimmune hepatitis.* Journal of Clinical Apheresis, 2010. **25**(2): p. 74-76.

1915. Liu, L.W., et al., *A Case of Probable Catastrophic Antiphospholipid Syndrome Treated with Rituximab and without Anticoagulation.* Journal of Clinical Rheumatology, 2021. **27**(8 S): p. S541-S542.

1916. Liu, Y., T.Y. Yang, and C.K. Tsai, *Pulmonary Embolism and Deep Vein Thrombosis after ChAdOx1 nCov-19 (Oxford-AstraZeneca) Vaccination: A Case of Vaccine-induced Thrombotic Thrombocytopenia.* Cerebrovascular Diseases, 2022. **51**(Supplement 1): p. 33-34.

1917. Lo, K.Y., C.Y. Chen, and C.S. Lee, *Hepatitis C virus-associated type II mixed cryoglobulinemia vasculitis complicated with membranous proliferative glomerulonephritis.* Renal Failure, 2009. **31**(2): p. 149-152.

1918. Lopez, R.R., et al., *Acute pancreatitis cause by severe hypertriglyceridemia: A case report.* Clinica Chimica Acta, 2019. **493**(Supplement 1): p. S211.

1919. Losos, M., et al., *The tipping point: The critical role of therapeutic apheresis in a case of refractory acquired hemophilia.* Journal of Clinical Apheresis, 2017. **32**(6): p. 564-566.

1920. Lowentritt, J.E., M.J. Kahn, and V. Batuman, *Use of recombinant hirudin as an anticoagulant in plasmapheresis in a patient with heparin-induced thrombocytopenia.* Nephrol Dial Transplant, 2002. **17**(8): p. 1533-4.

1921. Lucania, G., et al., *Multidisciplinary approach in pregnancy-associated thrombotic thrombocytopenic purpura: a case report.* Blood Transfus, 2014. **12 Suppl 1**(Suppl 1): p. s137-40.

1922. Luchsinger, D., et al., *Heparin-induced thromboytopenia (HIT) in a patient with systemic lupus erythematodes (SLE) and antiphospholipid antibody syndrome (APLAS).* Vasa - European Journal of Vascular Medicine, 2015. **44**(SUPPL. 89): p. 25.

1923. Madan, M., et al., *Combined diffuse alveolar hemorrhage and venous thrombosis in a patient with granulomatosis with polyangiitis: Case report and systematic review of literature.* Lung India, 2022. **39**(1): p. 70-73.

1924. Mader, E.C., Jr., et al., *Autoimmune Myelitis and Myocarditis in a Patient With Anti-Aquaporin-4, Antinuclear, and Antiphospholipid Autoantibodies: The Neuromyelitis Optica-Systemic Lupus Erythematosus (NMO-SLE) Overlap Syndrome.* Cureus, 2022. **14**(11): p. e31334.

1925. Madkaiker, S., *Catastrophic Antiphospholid Syndrome - An Unusual Case Report.* Indian J Crit Care Med, 2019. **23**(6): p. 276-280.

1926. Mahoney, R.C., R. Hagino, and E. Masuda, *Successful nonoperative management of mycotic radial artery pseudoaneurysm in patient with absent superficial palmar arch.* Journal of Vascular Surgery Cases and Innovative Techniques, 2020. **6**(3): p. 409-412.

1927. Malahfji, M., et al., *Catastrophic anti-phospholipid syndrome masquerading as hellp syndrome.* American Journal of Respiratory and Critical Care Medicine, 2014. **189**(MeetingAbstracts).

1928. Mandai, S., et al., *Recovery of renal function in a dialysis-dependent patient with microscopic polyangiitis and both myeloperoxidase anti-neutrophil cytoplasmic antibodies and anti-glomerular basement membrane antibodies.* Intern Med, 2011. **50**(15): p. 1599-603.

1929. Mandal, K., et al., *Severe hemolytic anemia : An extreme extrapulmonary manifestation of mycoplasma pneumoniae, it's management and successful recovery.* Journal of General Internal Medicine, 2015. **30**(SUPPL. 2): p. S318.

1930. Mao, M.A., et al., *Abdominal pain, flank pain, blurry vision, and lower extremity weakness in a 16-year-old female.* Arthritis Care and Research, 2014. **66**(9): p. 1423-1429.

1931. Mar, N., R. Kosowicz, and K. Hook, *Recurrent thrombosis prevention with intravenous immunoglobulin and hydroxychloroquine during pregnancy in a patient with history of catastrophic antiphospholipid syndrome and pregnancy loss.* Journal of Thrombosis and Thrombolysis, 2014. **38**(2): p. 196-200.

1932. Maroz, N., M. Burbank, and S. Adekoya, *Successful preoperative treatment of coagulopathy with therapeutic plasma exchange in the setting of acute liver failure and infective endocarditis.* Journal of Clinical Apheresis, 2020. **35**(6): p. 553-554.

1933. Mashhadi, M.A. and Z. Bari, *Thrombotic thrombocytopenic purpura and deep vein thrombosis as the presenting manifestations of systemic lupus erythematosus: A case report and review of literature.* Journal of Research in Medical Sciences, 2011. **16**(8).

1934. Matsumoto, S. and S. Nakagawa, *Extracorporeal Membrane Oxygenation for Diffuse Alveolar Hemorrhage Caused by Idiopathic Pulmonary Hemosiderosis: A Case Report and a Review of the Literature.* Journal of Pediatric Intensive Care, 2019. **8**(3): p. 181-186.

1935. Matsumura, T., et al., *A case of Duchenne muscular dystrophy complicated by thrombotic thrombocytopenic purpura.* Clinical Neurology, 2003. **43**(1-2): p. 31-34.

1936. Mayor Bastida, C., et al., *Encephalitis and thrombotic microangiopathy after mrna sarscov2 vaccine in a patient with chronic GVHD: A case report.* Bone Marrow Transplantation, 2022. **57**(Supplement 1): p. 129.

1937. McCollom, J.W., et al., *Catastrophic multi-organ failure with bone marrow necrosis in a sickle cell beta plus thalassemia patient.* Blood, 2014. **124**(21).

1938. McKinley, L., *A case of blue toe syndrome in a patient with lupus anticoagulant and proteus mirabilis septicemia.* Journal of the American Academy of Dermatology, 2010. **62**(3 SUPPL. 1): p. AB93.

1939. McLain, E., et al., *A case report of tandem plasma exchange and continuous renal replacement therapy.* Journal of Clinical Apheresis, 2011. **26**(2): p. 94-95.

1940. McLaughlin, D.C., et al., *Hypertriglyceridemia causing continuous renal replacement therapy dysfunction in a patient with end-stage liver disease.* Indian Journal of Nephrology, 2018. **28**(4): p. 303-306.

1941. McNeer, B., et al., *Pediatric orthotopic heart transplant requiring perioperative exchange transfusion: a case report.* J Extra Corpor Technol, 2004. **36**(4): p. 361-3.

1942. McRae, H.L., et al., *A rare case of catastrophic antiphospholipid syndrome triggered by estrogen-containing oral contraceptives in a patient with double heterozygous factor V Leiden and prothrombin G20210A mutations.* American Journal of Hematology, 2022. **97**(2): p. 239-242.

1943. Mehbali, Z., et al., *Liver infarction following an eclamptic seizure and cardiac arrest: Case report.* BJOG: An International Journal of Obstetrics and Gynaecology, 2018. **125**(Supplement 2): p. 78.

1944. Mehta, A.M., et al., *Essential thrombocythemia with portal vein thrombosis and splenic infarction successfully treated with platelet apheresis.* BMJ Case Rep, 2021. **14**(9).

1945. Meletti, L., et al., *Coupled plasma filtration absorption (CPFA) in one case of hepatocellular and obstructive jaundice.* Blood Purification, 2018. **46**(3): p. 185.

1946. Meyer, A.L., et al., *Implantation of a left ventricular assist device in a patient with primary antiphospholipid syndrome.* Ann Thorac Surg, 2008. **86**(2): p. 639-40.

1947. Miesbach, W., I. Scharrer, and R.A. Asherson, *Recurrent life-threatening thromboembolism and catastrophic antiphospholipid syndrome in a patient despite sufficient oral anticoagulation.* Clinical Rheumatology, 2004. **23**(3): p. 256-261.

1948. Miller, J.P. and P.D. Mintz, *Falsely low calcium measurements after high volume plasma exchange in a patient with liver failure.* Transfus Sci, 1994. **15**(3): p. 299-302.

1949. Miyamae, T., et al., *[Effective combination therapy of plasma exchange and subsequent cyclophosphamide pulses for catastrophic antiphospholipid antibody syndrome: a case report].* Ryumachi, 1999. **39**(3): p. 591-7.

1950. Mohamed, S., et al., *Operative consideration in patient with cryoglobulinaemia undergoing cardiac surgery with use of cardiopulmonary bypass.* Journal of Surgical Case Reports, 2020. **2020**(7): p. rjaa214.

1951. Mohanakrishnan, B.P.E., et al., *A RARE MICROTHROMBI DISEASE: CATASTROPHIC ANTIPHOSPHOLIPID ANTIBODY SYNDROME.* Chest, 2022. **162**(4 Supplement): p. A982.

1952. Mohnle, P., et al., *Emicizumab in the Treatment of Acquired Haemophilia: A Case Report.* Transfusion Medicine and Hemotherapy, 2019. **46**(2): p. 121-123.

1953. Moonla, C., et al., *Successful treatment of a rare acquired thrombin inhibitor in a patient with hemodialysis-dependent end-stagerenal disease.* Research and Practice in Thrombosis and Haemostasis, 2020. **4**(SUPPL 1): p. 389.

1954. Moranne, O., et al., *Longitudinal myelitis in a pregnant patient with sle [3].* American Journal of Medicine, 2004. **116**(5): p. 355-357.

1955. Morioka, S., et al., *[A case of systemic lupus erythematosus associated with severe fibrinoid necrosis located mainly in the glomerular afferent arteriole].* Nihon Jinzo Gakkai Shi, 1995. **37**(1): p. 69-73.

1956. Morita, M., et al., *Acute non-heparin-induced thrombocytopenia during hemodiafiltration in a patient with multiple myeloma.* Clinical Case Reports, 2019. **7**(4): p. 699-702.

1957. Moritoh, Y., et al., *Importance of blood pressure control in Kawasaki disease with expanded multiple giant coronary aneurysms with a 32 mm maximum diameter: A case report.* European Heart Journal - Case Reports, 2021. **5**(6): p. 1-7.

1958. Moroni, L., et al., *Catastrophic antiphospholipid syndrome presenting with aortic barrage: case report and review of the literature.* Lupus, 2021. **30**(6): p. 1005-1009.

1959. Morrow-Sutton, M.A., et al., *Therapeutic plasma exchange in left ventricular assist device patient with heparin induced thrombocytopenia.* Journal of Clinical Apheresis, 2019. **34**(2): p. 149-150.

1960. Morrow-Sutton, M.A., et al., *Use of therapeutic plasma exchange in the treatment of acute flaccid myelitis in a thirteen year old male with recent fever and upper respiratory illness.* Journal of Clinical Apheresis, 2019. **34**(2): p. 157.

1961. Mucha, S. and C. Samamras, *Multisystem organ failure from cryoglobulinemia, precipitated by therapeutic.* American Journal of Respiratory and Critical Care Medicine, 2018. **197**(MeetingAbstracts).

1962. Muller-Tidow, C., et al., *Heparin-induced thrombocytopenia vs. plasmapheresis- induced platelet loss in a case of thrombotic thrombocytopenic purpura.* Transfusion Medicine and Hemotherapy, 2007. **34**(1): p. 74-77.

1963. Mungee, S., et al., *Percutaneous coronary intervention in a patient with congenital factor XI deficiency and acquired inhibitor.* Cardiology, 2007. **107**(1): p. 69-72.

1964. Munir, A., et al., *A dilemma in vasculitis.* Chest, 2017. **152**(4 Supplement 1): p. A477.

1965. Mutlu, Y.G., et al., *Bortezomib and rituximab combination as a salvage therapy in a patient with refractory thrombotic thrombocytopenic purpura: Case report.* Leukemia Research, 2017. **61**(Supplement 1): p. S29.

1966. Nabizadeh, P., D.C. Patel, and H.M. Alnuaimat, *Successful application of extracorporeal membrane oxygenation without anticoagulation for diffuse alveolar hemorrhage secondary to microscopic polyangiitis.* American Journal of Respiratory and Critical Care Medicine, 2019. **199**(9).

1967. Naganuma, T., et al., *A case of anti-aquaporin-4 antibody-positive optic neuritis treated by selective immunoadsorption.* Transfusion and Apheresis Science, 2021. **60**(1): p. 102969.

1968. Naiden, T.V., S.J. Bartosh-Zelenaya, and N.N. Kovaleva, *Acute coronary syndrome and stroke in a yong woman.* European Heart Journal Cardiovascular Imaging, 2017. **18**(Supplement 3): p. iii29-iii30.

1969. Nair, N., R. Patel, and M. Athar, *A rare case of multiple organ thrombotic disorder: Is it caps?* Critical Care Medicine, 2009. **37**(12 SUPPL.): p. A516.

1970. Nakamura, H., et al., *Deep vein thrombosis and pulmonary thromboembolism in a patient with eosinophilia and obesity.* Acta Medica Nagasakiensia, 2007. **52**(3): p. 87-91.

1971. Nakano, K., et al., *[A case of systemic lupus erythematosus with pulmonary hypertension].* Ryumachi, 2000. **40**(3): p. 612-9.

1972. Nakaya, I., et al., *Pulmonary hemorrhage, due to rupture of small muscular arteries, in an autopsy case of systemic lupus erythematosus with antiphospholipid antibodies.* Modern Rheumatology, 2008. **18**(5): p. 529-531.

1973. Nascimento, S., et al., *Pregnancy with anti-PP1Pk antibody managed with prednisolone and low-molecular-weight heparin - A case report and literature review.* Case Rep Womens Health, 2020. **27**: p. e00238.

1974. Nasr, S., et al., *Pfizer-biontech COVID-19 RNA vaccination induces phosphatidylserine autoantibodies, cryoglobulinemia, and digital necrosis in a patient with pre-existing autoimmunity.* Clinical Immunology Communications, 2021. **1**: p. 1-3.

1975. Nathani, A. and N. Bhandari, *CATASTROPHIC ANTIPHOSPHOLIPID SYNDROME: A RARE BUT SERIOUS CONDITION.* Chest, 2020. **158**(4 Supplement): p. A924.

1976. Navaratnam, M., et al., *Epoprostenol Therapy for a Pediatric Patient with Subacute Heparin-Induced Thrombocytopenia and a Ventricular Assist Device Undergoing Heart Transplant: A Case Report.* A and A Practice, 2018. **11**(12): p. 329-331.

1977. Nawas, M.T., et al., *A protean protein.* Journal of Hospital Medicine, 2019. **14**(2): p. 117-122.

1978. Nelson, N., V.A. L, and R.A.D.U. Postelnicu, *SEVERE IMMUNE-MEDIATED DIFFUSE ALVEOLAR HEMORRHAGE IN THE AGE OF COVID-19 AND EXTRACORPOREAL MEMBRANE OXYGENATION.* Chest, 2022. **162**(4 Supplement): p. A2241.

1979. Ninan, E.C. and E. James, *Acute disseminated encephalomyelitis due to abrus precatorius poisoning - A case report.* Saudi Pharmaceutical Journal, 2019. **27**(4): p. 521-524.

1980. Nishida, M., et al., *[A case of fulminant hepatitis treated with heparin and plasma exchange using blood cell separator (celltrifuge) (author's transl)].* Nihon Naika Gakkai Zasshi, 1978. **67**(7): p. 723-8.

1981. Nishimura, H., et al., *Immunoadsorption plasmapheresis treatment for the recurrent exacerbation of neuromyelitis optica spectrum disorder with a fluctuating anti-aquaporin-4 antibody level.* Journal of Artificial Organs, 2018. **21**(3): p. 378-382.

1982. Nishimura, H., et al., *Combination treatment using percutaneous transluminal angioplasty and low-density lipoprotein apheresis in a patient with peripheral arterial disease and a history of chronic hemodialysis.* Journal of Clinical Apheresis, 2013. **28**(4): p. 330-334.

1983. Nonaka, T., et al., *Two cases of multisystem inflammatory syndrome in adults after improvement in severe acute respiratory distress syndrome due to coronavirus disease 2019.* Acute Medicine and Surgery, 2022. **9**(1): p. e737.

1984. Nunuk, I., et al., *Rare case of anti-GBM antibody disease with thrombotic microangiopathy caused by hypertensive emergency.* Journal of the American Society of Nephrology, 2019. **30**: p. 1189.

1985. Nussbaumer, W., et al., *Concomitant plasma exchange (TPE) during extra corporeal circulation (ECC) for a patient undergoing coronary artery bypass graft (CABG) pretreated with ticagrelor.* Journal of Clinical Apheresis, 2013. **28**(2): p. 120-121.

1986. Obara, K., et al., *Severe course of neuromyelitis optica in a female patient with chronic C hepatitis.* Neurologia i Neurochirurgia Polska, 2018. **52**(3): p. 397-400.

1987. Ochiai, K., et al., *A case of drug-induced liver injury successfully treated with plasma exchange therapy and ursodeoxycholic acid.* Acta Hepatologica Japonica, 2009. **50**(6): p. 297-302.

1988. Ogawa, H., et al., *A high titer of acquired factor V inhibitor in a hemodialysis patient who developed arterial thrombosis.* Int J Hematol, 2019. **109**(2): p. 214-220.

1989. Ogriki, T. and B. Fomberstein, *A fatal case of heparin-induced thrombocytopenia coexisting with secondary antiphospholipid syndrome.* Journal of General Internal Medicine, 2017. **32**(2 Supplement 1): p. S408.

1990. Ogunbameru, A., et al., *Acute pancreatitis as initial presentation of cocaine-induced vasculitis: A case report.* Journal of the Pancreas, 2015. **16**(2): p. 192-194.

1991. Ohhashi, J., et al., *Crescentic glomerulonephritis with positive antineutrophil cytoplasmic autoantibody specific for myeloperoxidase associated with autoimmune hemolytic anemia and thrombocytopenic purpura.* Intern Med, 2000. **39**(8): p. 650-4.

1992. Ohta, S., et al., *Venovenous Extracorporeal Membrane Oxygenation in Diffuse Alveolar Hemorrhage Secondary to Anti-neutrophil Cytoplasmic Autoantibody-associated Vasculitis: Starting without Systemic Anticoagulation.* Internal Medicine, 2022. **61**(23): p. 3569-3573.

1993. O'Laughlin, A. and C. Grossman, *A Case of Hyperhemolysis Syndrome Complicating Vasoocclusive Crisis, Pulmonary Embolism, and Acute Chest Syndrome.* American Journal of Respiratory and Critical Care Medicine, 2022. **205**(1).

1994. Olejarski, J.M., et al., *Bilateral adrenal hemorrhage: A rare complication of heparin induced thrombocytopenia.* Endocrine Reviews, 2014. **35**(SUPPL. 3).

1995. Olson, M.M., P.B. Ilada, and K.N. Apelgren, *Portal vein thrombosis.* Surg Endosc, 2003. **17**(8): p. 1322.

1996. Olszewski, A.J. and J.M. Shapiro, *Thrombotic thrombocytopenic purpura associated with abacavir in a patient with HIV infection.* J Intensive Care Med, 2003. **18**(3): p. 156-9.

1997. Orsino, A., et al., *Childhood Acute Myelomonocytic Leukemia (AML-M4) Presenting as Catastrophic Antiphospholipid Antibody Syndrome.* Journal of Pediatric Hematology/Oncology, 2004. **26**(5): p. 327-330.

1998. Ou, T.Y., et al., *Therapeutic plasma exchange in the treatment of complicated Plasmodium falciparum malaria: A case report.* Journal of Clinical Apheresis, 2018. **33**(3): p. 419-422.

1999. Ouyang, J., et al., *210 ACUTE RENAL FAILURE BY MASSIVE IVC THROMBOSES TREATED WITH MECHANICAL THROMBECTOMY: A CASE REPORT OF ATYPICAL CATASTROPHIC ANTIPHOSPHOLIPID SYNDROME.* American Journal of Kidney Diseases, 2021. **77**(4): p. 633.

2000. Overton, S.D., et al., *Autoamputation in a patient with cryoglobulinemia.* Journal of Rheumatology, 2004. **31**(10): p. 2088-2089.

2001. Owaidah, T.M., et al., *Successful treatment of a case of catastrophic antiphospholipid syndrome with autologous bone marrow transplantation.* Journal of Thrombosis and Haemostasis, 2009. **7**(S2): p. 721.

2002. Owaidah, T.M., et al., *Successful treatment of a case of catastrophic antiphospholipid syndrome with autologous BMT: Case report and review of literature.* Bone Marrow Transplantation, 2011. **46**(4): p. 597-600.

2003. Ozturk, K., et al., *Efficiency of rifampicin in emergency treatment of severe hyperbilirubinemia : Report of two cases and review of literature.* Acta Gastro-Enterologica Belgica, 2015. **78**(2): p. 256-258.

2004. Pais, F., M. Fayed, and T. Evans, *The successful use of extracorporeal membrane oxygenation in systemic lupus erythematosus-induced diffuse alveolar haemorrhage.* European Journal of Case Reports in Internal Medicine, 2017. **4**(1).

2005. Panchal, Y.N., M.M. Patel, and M.V. Patel, *THROMBOTIC THROMBOCYTOPENIC PURPURA AFTER VACCINATION AGAINST COVID-19 - A CASE REPORT.* Asian Journal of Pharmaceutical and Clinical Research, 2022. **15**(4): p. 1-3.

2006. Park, K., et al., *Catastrophic thrombotic syndrome in systemic lupus erythematosus without antiphospholipid antibodies.* Research and Practice in Thrombosis and Haemostasis, 2021. **5**(SUPPL 1).

2007. Patecki, M., et al., *A case report of severe calciphylaxis - suggested approach for diagnosis and treatment.* BMC Nephrology, 2017. **18**(1): p. 137.

2008. Patel, D. and J. Abunasser, *Plasma exchange as a therapeutic option for heparin-induced thrombocytopenia.* Chest, 2012. **142**(4 SUPPL. 1).

2009. Patel, N.J., M.E. Werlang, and M.C. Burton, *79-Year-Old Woman with Dyspnea, Cough, and Renal Failure.* Mayo Clinic Proceedings, 2015. **90**(3): p. e23-e27.

2010. Patel, S., *Caps: A catastrophic event.* Journal of General Internal Medicine, 2019. **34**(2 Supplement): p. S501.

2011. Patel, S., S. Ellis, and D. D'Cruz, *A SERIES OF CATASTROPHIC EVENTS.* Rheumatology Advances in Practice, 2021. **5**(Supplement 1): p. i14-i15.

2012. Patel, T.N., M. Kreindel, and A.M. Lincoff, *Use of ticlopidine and cilostazol after intracoronary drug-eluting stent placement in a patient with previous clopidogrel-induced thrombotic thrombocytopenic purpura: A case report.* Journal of Invasive Cardiology, 2006. **18**(7): p. E211-E213.

2013. Pena, A., et al., *A CASE OF MYOPERICARDITIS AND CATASTROPHIC ANTIPHOSPHOLIPID SYNDROME.* Journal of the American College of Cardiology, 2019. **73**(9 Supplement 1): p. 2949.

2014. Pereira, L. and M. O'Callaghan, *Plasmapheresis on ECMO: Case study.* Pediatric Nephrology, 2015. **30**(12): p. 2240.

2015. Peters, B.J., et al., *Effect of plasma exchange on antifactor Xa activity of enoxaparin and serum levetiracetam levels.* Am J Health Syst Pharm, 2018. **75**(23): p. 1883-1888.

2016. Petit, J.S., et al., *[Headaches in a 21-year-old man with Goodpasture disease].* Ann Fr Anesth Reanim, 2009. **28**(9): p. 799-802.

2017. Petmezci, M.T., et al., *A case of fulminant wilson's disease complicated with citrate toxicity.* Journal of Medical and Surgical Intensive Care Medicine, 2017. **8**(1): p. 25-27.

2018. Phelps, A. and W.B. Rothwell, *Not simply a heparin bridge.* Journal of General Internal Medicine, 2020. **35**(SUPPL 1): p. S523.

2019. Pirasath, S., et al., *Saw-scaled viper envenoming complicated with acute myocardial infarction.* SAGE Open Medical Case Reports, 2021. **9**.

2020. Pivalizza, E.G., *Heparinase and thromboelastography in liver transplantation for a patient with von Willebrand's disease.* Anesthesiology, 1996. **84**(5): p. 1236-1239.

2021. Plaza, M.G., et al., *Pyoderma gangrenosum in the elderly associated with collagen diseases. A propos of one case.* Revista Argentina de Dermatologia, 2004. **85**(4): p. 220-226.

2022. Plüß, M., et al., *Therapeutic response to glucocorticoids, anticoagulation and plasma exchange in a patient with primary antiphospholipid syndrome presenting with purpura fulminans.* Lupus, 2018. **27**(13): p. 2170-2173.

2023. Poullin, P., P.A. Pietri, and P. Lefèvre, *[Heparin-induced thrombopenia: rapid regression of thrombopenia and thrombosis after plasma exchange. Case report].* Rev Med Interne, 1998. **19**(11): p. 819-22.

2024. Qadir, A., et al., *PRESUMED CATASTROPHIC ANTIPHOSPHOLIPID SYNDROME.* Chest, 2020. **158**(4 Supplement): p. A747.

2025. Qiu, J., et al., *Therapeutic plasma exchange in a patient with hypertriglyceridemic necrotizing pancreatitis.* Journal of Clinical Apheresis, 2014. **29**(1): p. 32-33.

2026. Qiu, J., et al., *Management of an apheresis patient with a mechanical aortic valve on heparin and warfarin anticoagulation.* Journal of Clinical Apheresis, 2013. **28**(2): p. 108-109.

2027. Qorchi, H., N. Kissani, and M. Chraa, *Concomitant Guillain-Barre syndrome and cerebral venous thrombosis complicating a SARS-CoV-2 infection: a case report.* Pan African Medical Journal, 2022. **42**(212): p. 212.

2028. Quaye, E.N., *A rare auto-immune-like syndrome associated with nivolumab therapy.* Journal of General Internal Medicine, 2019. **34**(2 Supplement): p. S449-S450.

2029. Quigley, J., A. Keating, and L. Byrd, *Fatigue and breathlessness in pregnancy: A rare and sinister cause.* BMJ Case Reports, 2014. **2014**: p. 1971.

2030. Quinn, R.E. and E. Bernardo, *Investigating the use of ECMO in children with ANCA+pulmonary hemorrhage: A case report.* Pediatrics, 2021. **147**(3): p. 442-443.

2031. Quintero-Munoz, E., et al., *Is there any relationship between massive ascites and elevated CA-125 in systemic lupus erythematosus? Case report and review of the literature.* Modern Rheumatology Case Reports, 2021. **5**(2): p. 292-299.

2032. Raflores, M.B., R.B. Kaplan, and J.A. Spero, *Pre-operative management of a patient with hypoprothrombinemia-lupus anticoagulant syndrome.* Thromb Haemost, 2007. **98**(1): p. 248-50.

2033. Raj, A., et al., *Recurrent Hepatic Artery Thrombosis Following Living Donor Liver Transplant as Sequelae of SARS-CoV-2 Infection-a Case Report.* SN Comprehensive Clinical Medicine, 2021. **3**(12): p. 2629-2634.

2034. Rajabally, H., C. Morley, and S. Srivastava, *A case of granulomatosis with polyangiitis complicated by COVID-19: Challenges in diagnosis and management.* Rheumatology Advances in Practice, 2020. **4**(SUPPL 1): p. i20-i21.

2035. Rakiro, J. and D. Sokhi, *Fatal autoimmune anti-nmda-receptor encephalitis with poor prognostication score in a young kenyan female.* International Medical Case Reports Journal, 2021. **14**: p. 343-347.

2036. Ramesh, R., et al., *Allthat scatters is not heart: A case of ttp presenting as multiterritorial stroke.* International Journal of Stroke, 2020. **15**(1 SUPPL): p. 697.

2037. Rao, A.A.N., et al., *Rituximab in the management of pediatric catastrophic antiphospholipid syndrome: A case report.* Haemophilia, 2009. **15**(2): p. 626-627.

2038. Ratiani, M., J. Cho, and A. Treml, *Therapeutic plasma exchange in a patient with pure red cell aphasia post peripheral blood stem cell transplant with major abo mismatch.* Journal of Clinical Apheresis, 2021. **36**(2): p. 262-263.

2039. Raza, A., et al., *Catastrophic anti-phospholipid syndrome in a 27-year old female.* Journal of Hospital Medicine, 2012. **7**(SUPPL. 2): p. S285.

2040. Razo, D., C. Dela Cruz-Tan, and J. Suguran, *A 23 year old female with systemic lupus erythematosus presenting with multiple organ thrombosis: A case of catastrophic antiphospholipid syndrome.* International Journal of Rheumatic Diseases, 2021. **24**(SUPPL 2): p. 135.

2041. Rehak, M., et al., *Occlusion of choroidal vessels in a patient with catastrophic antiphospholipid syndrome.* Acta Ophthalmologica, 2011. **89**(6): p. 595-596.

2042. Reis, T., et al., *Regional Hypertonic Citrate Anticoagulation in Membrane Therapeutic Plasma Exchange: A Case Series.* Can J Kidney Health Dis, 2021. **8**: p. 20543581211054736.

2043. Rela, M., et al., *First report of auxiliary liver transplantation for severe cholangiopathy after SARS-CoV-2 respiratory infection.* American Journal of Transplantation, 2022. **22**(12): p. 3143-3145.

2044. Ren, F., et al., *Amniotic fluid and pulmonary emboli in a patient with SLE and a twin gestation.* Rheumatology (Bulgaria), 2020. **59**(11): p. E107-E108.

2045. Renard, D., et al., *Anti-MuSK positivity on plasmapheresis liquid in a double seronegative myasthenia gravis patient.* Acta Neurologica Belgica, 2018. **118**(1): p. 119-120.

2046. Reynaud, A., et al., *Acquired factor v inhibitor: Case discussion.* Fundamental and Clinical Pharmacology, 2010. **24**(SUPPL. 1): p. 86.

2047. Richter, J.R., et al., *Management of hemolysis and multiorgan failure with therapeutic plasma exchange in a patient with sickle cell disease.* Journal of Clinical Apheresis, 2018. **33**(2): p. 181-182.

2048. Riemekasten, G., et al., *Shwartzman phenomenon in a patient with active systemic lupus erythematosus preceding fatal disseminated intravascular coagulation.* Lupus, 2002. **11**(4): p. 204-207.

2049. Ries, W., et al., *Therapeutic plasma exchange membrane plasmapheresis versus centrifugal plasmapheresis: A case report.* Therapeutic Apheresis and Dialysis, 2011. **15**(4): p. A13.

2050. Ries, W., et al., *A women with a chylomicronemia syndrome complicated by acute pancreatitis treated by cDFPP.* International Journal of Artificial Organs, 2012. **35**(8): p. 564.

2051. Ries, W., et al., *Deteriorating neurological function during an outbreak with shiga toxin-producing escherichia coli in northern germany.* Therapeutic Apheresis and Dialysis, 2011. **15**(4): p. A14.

2052. Rigamonti, E., et al., *A case-report of combined Shiga-toxin associated hemolytic uremic syndrome and heparin-induced thrombocytopenia.* Swiss Medical Weekly, 2020. **150**(SUPPL 248): p. 31S-32S.

2053. Rigamonti, E., et al., *Heparin-induced thrombocytopenia following Shiga-toxin-associated hemolytic uremic syndrome: a case report.* Journal of Medical Case Reports, 2022. **16**(1): p. 384.

2054. Robbins, L., *Migraine and anticardiolipin antibodies - Case reports of 13 patients, and the prevalence of antiphospholipid antibodies in migraineurs.* Headache, 1991. **31**(8): p. 537-539.

2055. Robinett, K.S., M. McCurdy, and A.C. Verceles, *An unusual case of thrombocytopenia, altered mental status with renal and splenic infarctions in a 25-year old patient with systemic lupus erythematosus.* American Journal of Respiratory and Critical Care Medicine, 2010. **181**(1 MeetingAbstracts).

2056. Rodgers, S.A., et al., *Paradoxical embolic strokes in a liver transplant recipient with atrial septal defect undergoing therapeutic plasma exchange.* J Clin Apher, 2021. **36**(1): p. 206-210.

2057. Roit, Z., J. Weil, and I. Llovera, *More Than Skin Deep: A Case of Catastrophic Antiphospholipid Syndrome.* Journal of Emergency Medicine, 2019. **57**(6): p. 880-882.

2058. Romano, J., P. Mehrotra, and E. Wasserman, *Development of Cryoglobulinemia and Acute Limb Ischemia in Patient with Underlying Inflammatory Bowel Disease.* American Journal of Respiratory and Critical Care Medicine, 2022. **205**(1).

2059. Romano, J., P. Mehrotra, and E. Wasserman, *CRYOGLOBULINEMIA AND ACUTE LIMB ISCHEMIA IN A PATIENT WITH UNDERLYING INFLAMMATORY BOWEL DISEASE.* Critical Care Medicine, 2023. **51**(1 Supplement): p. 159.

2060. Romic, M., et al., *Urgent cardiac surgery in a patient with heparin induced thrombocytopenia.* Research and Practice in Thrombosis and Haemostasis, 2021. **5**(SUPPL 2).

2061. Roncin, K.L., et al., *Role of therapeutic plasma exchange in management of prolonged and severe myasthenic exacerbation.* Journal of Clinical Apheresis, 2019. **34**(2): p. 155-156.

2062. Rong, J., et al., *Fructus Psoraleae-Induced Severe Liver Injury and Treatment With Two Artificial Liver Support Systems: A Case Series Study.* Therapeutic Apheresis and Dialysis, 2020. **24**(3): p. 324-332.

2063. Ronthal, M., et al., *Case 21-2003: A 72-year-old man with repetitive strokes in the posterior circulation.* New England Journal of Medicine, 2003. **349**(2): p. 170-180.

2064. Rose, A. and D. Sajkov, *A 29-year-old man with pulmonary complications of a thrombotic disorder.* Chest, 2007. **132**(6): p. 2008-11.

2065. Rosenbaum, A.N., et al., *A case of catastrophic antiphospholipid syndrome: first report with advanced cardiac imaging using MRI.* Lupus, 2015. **24**(12): p. 1338-41.

2066. Routy, B., et al., *Vascular endothelial cell function in catastrophic antiphospholipid syndrome: a case report and review of the literature.* Case Rep Hematol, 2013. **2013**: p. 710365.

2067. Ruffatti, A., et al., *Insights into the pathogenesis of catastrophic antiphospholipid syndrome. A case report of relapsing catastrophic antiphospholipid syndrome and review of the literature on ischemic colitis.* Clinical Rheumatology, 2020. **39**(4): p. 1347-1355.

2068. Ruiz, M., et al., *Limb salvation: Prophylactic heparin for purpura fulminans.* Critical Care Medicine, 2019. **47**(1 Supplement 1).

2069. Russell, S., P. Manning, and R. Cruz, *Tricky to pinpoint: An unusual cause of chest pain in a young man.* Journal of General Internal Medicine, 2019. **34**(2 Supplement): p. S671.

2070. Rymarz, A. and S. Niemczyk, *The complex treatment including rituximab in the Management of Catastrophic Antiphospholid Syndrome with renal involvement.* BMC Nephrology, 2018. **19**(1): p. 132.

2071. Saadi, M.W., T. Evans, and S. Khwaja, *First reported case of successful use of veno-arterial extracorporeal membrane oxygenation without the use of systemic anticoagulation in the management of profound hypoxemia in diffuse alveolar hemorrhage complicating systemic lupus erythematosus.* American Journal of Respiratory and Critical Care Medicine, 2014. **189**(MeetingAbstracts).

2072. Sachais, B.S., J.E. Thompson, and F.J. Strobl, *Use of plasma exchange, steroids, and anticoagulation in a patient with multiple arterial thromboses and IgM anticardiolipin antibodies.* J Clin Apher, 2002. **17**(3): p. 138-9.

2073. Sacks, L., et al., *Prolonged va-ecmo for pediatric multi-organ failure: 195 days of successful support.* ASAIO Journal, 2021. **67**(SUPPL 3): p. 33.

2074. Safi, F., et al., *Management of familial hypertriglyceridemia induced pancreatitis during pregnancy with therapeutic plasma exchange.* Chest, 2012. **142**(4 SUPPL. 1).

2075. Safi, F., et al., *Management of familial hypertriglyceridemia-induced pancreatitis during pregnancy with therapeutic plasma exchange: A case report and review of literature.* American Journal of Therapeutics, 2014. **21**(5): p. e134-e136.

2076. Saito, T., et al., *Microscopic polyangiitis associated with marked systemic bleeding tendency caused by disseminated intravascular coagulation.* Intern Med, 2003. **42**(9): p. 850-5.

2077. Salehi, N., E.D. Choi, and R.C. Garrison, *A case of miller fisher syndrome, thromboembolic disease, and angioedema: Association or coincidence?* American Journal of Case Reports, 2017. **18**: p. 52-59.

2078. Samimagham, H.R., M.K. Jahromi, and L.K. Jahromi, *Covid-19 with pneumomediastinum and emphysema, a case report.* Shiraz E Medical Journal, 2020. **21**(12): p. 1-3.

2079. Sandoval, E., et al., *Heparin induced thrombocytopenia, VA-ECMO and total plasma exchange.* Perfusion (Germany), 2019. **34**(1 Supplement): p. 169.

2080. Santarelli, I.M., et al., *Raynaud Phenomenon With Severe Ulcers Associated With Cryoglobulins in an HIV+ but HCV Negative Patient.* J Clin Rheumatol, 2016. **22**(6): p. 335-7.

2081. Santos, A., et al., *Plasmapheresis as an Alternative Treatment of Hypertriglyceridemia-Induced Pancreatitis: A Case Report.* Cureus, 2022. **14**(11): p. e32000.

2082. Sasaki, Y., et al., *[A case of intractable hemoptysis due to malignant hemangioendothelioma].* Nihon Kyobu Shikkan Gakkai Zasshi, 1992. **30**(1): p. 123-7.

2083. Sathawarawong, W., *Thrombotic thrombocytopenic purpura (TTP): 4 Case reports and review of the literature.* Journal of the Medical Association of Thailand, 1995. **78**(6): p. 322-331.

2084. Sathiagnanam, P., et al., *Thrombotic thrombocytopenic purpura presenting as a stroke.* International Journal of Stroke, 2013. **8**(SUPPL. 3): p. 44-45.

2085. Sato, R., et al., *Minimal change nephrotic syndrome sequentially complicated by acute kidney injury and painful skin ulcers due to calciphylaxis.* Internal Medicine, 2016. **55**(22): p. 3315-3320.

2086. Satoh, K., et al., *Continuous Plasma Exchange With Dialysis for Severe Sepsis: Case Series of a Novel Blood Purification Method.* Cureus, 2021. **13**(1): p. e12495.

2087. Satomi, A., et al., *Plasma exchange for thrombocytopenia in antiphospholipid syndrome: a case report.* Ther Apher, 1998. **2**(2): p. 157-9.

2088. Scagnelli, A.M., et al., *Effects of therapeutic plasma exchange on serum immunoglobulin concentrations in a dog with refractory immune-mediated hemolytic anemia.* J Am Vet Med Assoc, 2018. **252**(9): p. 1108-1112.

2089. Scala, E., et al., *Cardiac Surgery Successfully Managed With Cangrelor in a Patient With Persistent Anti-PF4/Heparin Antibodies 8 Years After Heparin-Induced Thrombocytopenia.* J Cardiothorac Vasc Anesth, 2019. **33**(11): p. 3073-3077.

2090. Schenk, S., et al., *Triple bridge-to-transplant in a case of giant cell myocarditis complicated by human leukocyte antigen sensitization and heparin-induced thrombocytopenia type II.* Annals of Thoracic Surgery, 2006. **81**(3): p. 1107-1109.

2091. Schleinitz, N., et al., *[Acquired factor V inhibitor: etiology, bleeding risk and therapeutic management with regard to three cases].* Rev Med Interne, 2001. **22**(11): p. 1119-23.

2092. Schuler, U.S., et al., *Fulminant improvement in a patient with prostate cancerassociated-TTP (CA-TTP) after initiation of enzalutamide therapy in a Palliative Care Unit (PCU).* Oncology Research and Treatment, 2016. **39**(Supplement 3): p. 175-176.

2093. Schulman, R., et al., *Skim the fat: Plex for hypertriglyceridemia-induced CRRT clotting.* Journal of the American Society of Nephrology, 2020. **31**: p. 398-399.

2094. Schultz, C., et al., *Hemoperitoneum complicating an oocyte puncture in a chronic hemodialysis patient.* J Nephrol, 2022. **35**(9): p. 2433-2435.

2095. Schweiberer, G. and W. Vogelgesang, *Acute renal failure in a case of bismuth intoxication.* Nieren- und Hochdruckkrankheiten, 1993. **22**(8): p. 381-385.

2096. Seda, G. and D. Amundson, *Acute management of severe hypertriglyceridemia with plasmapheresis in diabetic ketoacidosis.* Chest, 2009. **136**(4).

2097. Seishima, M., et al., *Decreased factor XIII activity in a patient with subcutaneous bleeding after double filtration plasmapheresis.* Therapeutic Apheresis and Dialysis, 2009. **13**(3): p. 229-231.

2098. Senda, Y., et al., *Microangiopathic antiphospholipid antibody syndrome due to anti-phosphatidylserine/prothrombin complex IgM antibody.* Pediatrics International, 2017. **59**(3): p. 378-380.

2099. Serrano, I.A., et al., *Controversy of heparin use in hyper triglyceridemia-induced pancreatitis: A case report.* Journal of General Internal Medicine, 2018. **33**(2 Supplement 1): p. 497.

2100. Sevy, A., et al., *Stroke in a young patient treated by alteplase heralding an acquired thrombotic thrombocytopenic purpura.* Journal of Clinical Apheresis, 2011. **26**(3): p. 152-155.

2101. Shah, P., et al., *COVID-19-INDUCED EVANS SYNDROME: UNUSUAL COMPLICATION OF THE NEW USUAL.* Chest, 2022. **162**(4 Supplement): p. A627.

2102. Sharma, V.D., et al., *Blind and confused.* JAMA Neurology, 2013. **70**(7): p. 932-936.

2103. Sharma, Y., K. Humphreys, and C. Thompson, *Extensive abdominal wall ulceration as a late manifestation of antiphospholipid syndrome: A case report.* Journal of Medical Case Reports, 2018. **12**(1): p. 226.

2104. Sheares, K.K.K. and R. Mahadeva, *Recombinant factor VIIa and intravenous immunoglobulin therapy for diffuse alveolar haemorrhage: A cautionary tale?* Respiratory Medicine Extra, 2005. **1**(4): p. 120-123.

2105. Sheikh, B., et al., *IMPORTANCE OF IDENTIFYING CATASTROPHIC ANTIPHOSPHOLIPID SYNDROME IN THE ICU.* Chest, 2020. **158**(4 Supplement): p. A856.

2106. Shen, H., et al., *Acquired heparin-like anticoagulation process in a patient with multiple myeloma: A case report and literature review.* Translational Cancer Research, 2020. **9**(11): p. 7366-7371.

2107. Shimizu, M., et al., *A case of a 6-year-old girl with anti-neutrophil cytoplasmic autoantibody-negative pauci-immune crescentic glomerulonephritis.* Clinical and Experimental Nephrology, 2011. **15**(4): p. 596-601.

2108. Shinohara, T., et al., *Calcinosis cutis and intestinal pseudoobstruction in a patient with adult onset Still's disease associated with recurrent relapses of disordered coagulopathy.* Intern Med, 1999. **38**(6): p. 516-20.

2109. Shioya, N., et al., *Cardiopulmonary arrest caused by nafamostat mesylate during hemodialysis.* Clinical Case Reports, 2022. **10**(7): p. e6140.

2110. Shkodivskyi, P., et al., *A case of acute optic neuritis during pregnancy treated by membrane-based therapeutic plasma exchanges without systemic anticoagulation.* Transfus Apher Sci, 2021. **60**(5): p. 103178.

2111. Shmookler, A.D., R. Arays, and P. McCarthy, *Inpatient management strategies in a severe case of heparin-induced thrombocytopenia.* Transfus Apher Sci, 2019. **58**(4): p. 525-528.

2112. Shyu, S., M. Rubin, and C.B. Drachenberg, *Fatal unsuspected fat embolism syndrome in a sickle cell patient.* Human Pathology: Case Reports, 2017. **8**: p. 62-64.

2113. Siami, G.A. and F.S. Siami, *Intensive tandem cryofiltration apheresis and hemodialysis to treat a patient with severe calciphylaxis, cryoglobulinemia, and end-stage renal disease.* Asaio j, 1999. **45**(3): p. 229-33.

2114. Silvey, M., S. Carpenter, and B. Wicklund, *Hemophilia a patient with high titer inhibitor and fatal pulmonary embolism-a single case report.* American Journal of Hematology, 2012. **87**(SUPPL. 1): p. S159.

2115. Singhal, A.L., et al., *Severe cytokine release syndrome after basiliximab induction therapy.* American Journal of Respiratory and Critical Care Medicine, 2021. **203**(9).

2116. Skoczynska, M., et al., *Thrombotic microangiopathy in the course of catastrophic antiphospholipid syndrome successfully treated with eculizumab: case report and systematic review of the literature.* Lupus, 2020. **29**(6): p. 631-639.

2117. Sleth, J.C., et al., *A case of hypertriglycideremia-induced pancreatitis in pregnancy: Value of heparin.* Annales Francaises d'Anesthesie et de Reanimation, 2004. **23**(8): p. 835-837.

2118. Sleth, J.C., et al., *[A case of hypertriglycideremia-induced pancreatitis in pregnancy: value of heparin].* Ann Fr Anesth Reanim, 2004. **23**(8): p. 835-7.

2119. Soboleva, M., et al., *Fatal outcome of mitral native valve endocarditis due to misdiagnosis and using heparin.* International Journal of Antimicrobial Agents, 2013. **41**(6 SUPPL. 1): p. S13.

2120. Sofue, T., et al., *Plasmapheresis in a patient with antiphospholipid syndrome before living-donor kidney transplantation: A case report.* BMC Nephrology, 2015: p. 1-6.

2121. Solimando, A.G., et al., *Multiple myeloma that progressed as type i cryoglobulinemia with skin ulcers and foot necrosis: A case report.* Medicine (United States), 2018. **97**(39): p. e12355.

2122. Soltanpour, K., et al., *A case of concurrent catastrophic antiphospholipid syndrome and IgA nephropathy.* Journal of the American Society of Nephrology, 2017. **28**: p. 1127.

2123. Sorour, A., et al., *Catastrophic Antiphospholipid Syndrome Following Lower Extremity Arterial Bypass Surgery.* EJVES Vascular Forum, 2022. **54**: p. e6.

2124. Sorour, A.A., et al., *Catastrophic Antiphospholipid Syndrome Following Lower Extremity Arterial Bypass Surgery: Case Report and Evidence Review.* Vascular and Endovascular Surgery, 2022. **56**(2): p. 196-200.

2125. Sourabh, S., et al., *A rare case of acute pancreatitis due to very severe hypertriglyceridemia successfully treated by long-acting insulin.* Journal of General Internal Medicine, 2020. **35**(SUPPL 1): p. S395.

2126. Sprenger-Mahr, H., et al., *Successful pregnancy in a patient with pulmonary renal syndrome double-positive for anti-GBM antibodies and p-ANCA.* Clinical Nephrology, 2019. **91**(2): p. 101-106.

2127. Sreckovic, M., et al., *Multisystem inflammatory syndrome in a young adult successfully treated with plasmapheresis, immunoglobulins, and corticosteroids: a case report.* Int J Infect Dis, 2022. **122**: p. 1052-1055.

2128. Stam-Slob, M.C., et al., *Thrombotic micro-angiopathy due to Strongyloides stercoralis hyperinfection.* Clinical Infection in Practice, 2022. **15**: p. 100153.

2129. Stanley, J. and O. Lateef, *Severe manifestations and treatment in catastrophic antiphospholipid syndrome.* Critical Care Medicine, 2013. **41**(12 SUPPL. 1): p. A349.

2130. Stapleton, A., D. Ling, and D. Kahn, *Diagnosing thrombotic thrombocytopenic purpura in a patient with systemic lupus erythematosus.* Journal of General Internal Medicine, 2014. **29**(SUPPL. 1): p. S343-S344.

2131. Stephenson, D. and A. Dave, *Catastrophic antiphospholipid antibody syndrome in a woman with recent renal transplant.* Journal of Hospital Medicine, 2010. **5**(SUPPL. 1): p. 185.

2132. Stolfa, J., et al., *Development of disseminated intravascular coagulation in a patient with systemic lupus erythematodes and secondary antiphospholipid syndrome: Case record.* Ceska Revmatologie, 1996. **4**(4): p. 179-183.

2133. Strakhan, M., et al., *36-year-old female with catastrophic antiphospholipid syndrome treated with eculizumab: a case report and review of literature.* Case Rep Hematol, 2014. **2014**: p. 704371.
[truncated: 597,261 more chars]
