# Supplementary material for: Comparison of efficacy and safety of different anticoagulation regimens in plasma exchange: A systematic review and meta-analysis
Source: PLoS One. 2024 Oct 24;19(10):e0311603. doi: 10.1371/journal.pone.0311603 (PMC11500872; doi:10.1371/journal.pone.0311603)
Supplement: S4 File — (DOCX) [file pone.0311603.s004.docx]

**Table S4. Reported outcomes of included studies.**

| Author/Year | Clotting, n | | | | Bleeding, n | | | | Post-treatment APTT (s), mean | | | | Post-treatment PLT counts (x10^9^/L), mean | | | |
| --- | --- | --- | --- | --- | --- | --- | --- | --- | --- | --- | --- | --- | --- | --- | --- | --- |
|  | UFH | LMWH | RCA | Saline | UFH | LMWH | RCA | Saline | UFH | LMWH | RCA | Saline | UFH | LMWH | RCA | Saline |
| Brunetta, 2017[1] | 183 | 69 | - | 75 | 9 | 2 | - | 0 | - | - | - | - | - | - | - | - |
| Yuan, 2018[2] | - | - | - | - | 23 | - | - | 9 | 104.1 | - | - | 60.6 | 81.8 | - | - | 84.9 |
| Yuan, 2020[3] | 9 | - | 0 | 14 | 4 | - | 0 | 0 | 48.7 | - | 40.1 | 41.0 | 98.0 | - | 107.0 | 99.0 |
| Teh S, 2022[4] | 3 | - | 10 | - | 0 | - | 3 | - | - | - | - | - | - | - | - | - |
| Ma, 2019[5] | 20 | - | 16 | - | 3 | - | 0 | - | 162.7 | - | 122.5 | - | - | - | - | - |
| Zhang, 2022[6] | 5 | 0 | - | - | 3 | 1 | - | - | 61.3 | 49.4 | - | - | 110.6 | 128.4 | - | - |
| Pan, 2015[7] | 19 | 3 | - | - | 32 | 0 | - | - | 71.7 | 62.1 | - | - | 88.8 | 114.4 | - | - |

Abbreviations: APTT, activated partial thrombotic time; L, liter; LMWH, low molecular weight heparin; n, number; PLT, platelet; PT, prothrombin time; RCA, regional citrate acid; s, second; UFH, unfractionated heparin.

**References**

1. Brunetta Gavranić B, Bašić-Jukić N, Premužić V, Kes P: **Membrane therapeutic plasma exchange with and without heparin anticoagulation**. *J Clin Apher* 2017, **32**(6):479-485.

2. Yuan S, Qian Y, Tan D, Mo D, Li X: **Therapeutic plasma exchange: A prospective randomized trial to evaluate 2 strategies in patients with liver failure**. *Transfus Apher Sci* 2018, **57**(2):253-258.

3. Yuan F, Li Z, Li X, Liu H: **Application of regional citrate anticoagulation in membrane therapeutic plasma exchange**. *International urology and nephrology* 2020, **52**(12):2379-2384.

4. Teh SP, Ho QY, Kee YST, Thangaraju S, Tan RY, Teo SH, Tan HK, Tan CS, Choong HLL, Ng LC *et al*: **Regional citrate anticoagulation vs systemic heparin anticoagulation for double-filtration plasmapheresis**. *J Clin Apher* 2023, **38**(1):16-23.

5. Ma Y, Chen F, Xu Y, Wang M, Zhou T, Lu J, Feng P, Wang Y, Bai L, Tang H: **Safety and Efficacy of Regional Citrate Anticoagulation during Plasma Adsorption Plus Plasma Exchange Therapy for Patients with Acute-on-Chronic Liver Failure: A Pilot Study**. *Blood Purif* 2019, **48**(3):223-232.

6. Zhang Jie ZC, Zheng Rongjiong, Deng Zerun, Sun Lihua, Yao Lei, Lu Xiaobo: **Comparison of the efficay and safety of different anticoagulant drugs in the treatmetn of liver failure with dual bilirubin adsorption combined wit plasma exchange**. *Journal of Xingjiang Medical University* 2022, **45**(2):5.

7. Pan Ye OH, Zeng Jianyong, Wu Xiaolu, Liu Jiajun, Wei Jihong: **Analysis of unfractionated heparin and low molecular weight heparin in plasma exchange**. *China Medical Engineering* 2015, **23**(1):2.
