# Supplementary material for: Comparison of efficacy and safety of different anticoagulation regimens in plasma exchange: A systematic review and meta-analysis
Source: PLoS One. 2024 Oct 24;19(10):e0311603. doi: 10.1371/journal.pone.0311603 (PMC11500872; doi:10.1371/journal.pone.0311603)
Supplement: S5 File — (DOCX) [file pone.0311603.s005.docx]

Table S5. The bias risk for each study in this meta-analysis based on the Cochrane tool.

| Study | Selection bias | Performance bias | Attrition bias | Detection bias | Reporting bias |
| --- | --- | --- | --- | --- | --- |
| Brunetta 2017 | Low | Low | Low | Unclear | High |
| Ma Y 2019 | Low | Low | Low | Low | Low |
| Pan Y 2015 | Unclear | Low | Low | Low | Low |
| The S 2023 | Low | Unclear | High | High | Unclear |
| Yuan F 2020 | Low | Low | Low | Unclear | Low |
| Yuan S 2018 | Low | High | Unclear | Low | Unclear |
| Zhang J 2022 | High | Low | High | Unclear | High |
