# Supplementary material for: Comparison of efficacy and safety of different anticoagulation regimens in plasma exchange: A systematic review and meta-analysis
Source: PLoS One. 2024 Oct 24;19(10):e0311603. doi: 10.1371/journal.pone.0311603 (PMC11500872; doi:10.1371/journal.pone.0311603)
Supplement: Attachment — Submitted filename: PONE-D-24-02893_reviewer.pdf [file pone.0311603.s006.pdf]

PLOS ONE

Comparison of Efficacy and Safety of Different Anticoagulation Regimens in Plasma Exchange: A Systematic Review and Meta-analysis

--Manuscript Draft--

**Manuscript Number:**	PONE-D-24-02893
**Article Type:**	Research Article
**Full Title:**	Comparison of Efficacy and Safety of Different Anticoagulation Regimens in Plasma Exchange: A Systematic Review and Meta-analysis
**Short Title:**	Anticoagulation Regimens in Plasma Exchange: A Meta-analysis
**Corresponding Author:**	Song Ren<br>Sichuan Academy of Medical Sciences and Sichuan People's Hospital<br>Chengdu, CHINA
**Keywords:**	plasma exchange, anticoagulation, efficacy, safety, systematic review, meta-analysis
**Abstract:**	Background Extracorporeal line clotting during plasma exchange (PE) not only delays efficient treatment, but also cause great waste of nursing resources. There is a lack of comprehensive comparison of the efficacy and safety among different anticoagulation regimens in plasma exchange in literature.<br>Methods A systematic search was performed in EMBASE, MEDLINE via PubMed, Cochrane Central Library, and CNKI. Studies that had compared at least two anticoagulation regimens in PE were considered eligible. The anticoagulative efficacy outcome was assessed by the occurrence of extracorporeal circuit clotting. The safety outcome was assessed by the occurrence of bleeding events, post-treatment APTT values, and post-treatment platelets counts. The risk of bias was assessed by the AHRQ tool. Mean differences or standardized mean differences with 95% confidence intervals (CIs) of continuous variables and risk ratios (RRs) with 95% CIs of categorical variables were pooled using a random-effects or a fixed-effects model as appropriate.<br>Results In all, 7 studies with 1638 patients and 10951 sessions of PE treatment were included. Pooled results indicated the anticoagulative efficacy of UFH was better than that of saline flushing, yet did not differ with those of LMWH or RCA. Although the occurrence of bleeding events had no difference among different pairs of anticoagulation regimens, anticoagulation using UFH might lead to longer post-treatment APTT value and lower post-treatment platelet counts. Only one study was judged to have low risk of bias in each of the five domains in the AHRQ tool.<br>Conclusions The current anticoagulation regimens are generally effective and well tolerated in PE; however, the number of included studies was too limited to draw definitive conclusions.
**Order of Authors:**	Song Ren
	Liming Huang
	Yi Li
	Yunlin Feng
**Additional Information:**	
**Question**	**Response**
**Financial Disclosure**<br><br>Enter a financial disclosure statement that describes the sources of funding for the work included in this submission. Review the submission guidelines for detailed requirements. View published research articles from *PLOS ONE* for specific examples.	The author(s) received no specific funding for this work.

Powered by Editorial Manager® and ProduXion Manager® from Aries Systems Corporation

This statement is required for submission and **will appear in the published article** if the submission is accepted. Please make sure it is accurate.<br><br>**Funded studies**<br>Enter a statement with the following details:<br>• Initials of the authors who received each award<br>• Grant numbers awarded to each author<br>• The full name of each funder<br>• URL of each funder website<br>• Did the sponsors or funders play any role in the study design, data collection and analysis, decision to publish, or preparation of the manuscript?<br><br>Did you receive funding for this work?	
**Competing Interests**<br><br>Use the instructions below to enter a competing interest statement for this submission. On behalf of all authors, disclose any competing interests that could be perceived to bias this work—acknowledging all financial support and any other relevant financial or non-financial competing interests.<br><br>This statement is required for submission and **will appear in the published article** if the submission is accepted. Please make sure it is accurate and that any funding sources listed in your Funding Information later in the submission form are also declared in your Financial Disclosure statement.<br><br>View published research articles from *PLOS ONE* for specific examples.	The authors have declared that no competing interests exist.

Powered by Editorial Manager® and ProduXion Manager® from Aries Systems Corporation

**NO authors have competing interests**<br><br>Enter: *The authors have declared that no competing interests exist.*<br><br>**Authors with competing interests**<br><br>Enter competing interest details beginning with this statement:<br><br>*I have read the journal's policy and the authors of this manuscript have the following competing interests: [insert competing interests here]*<br><br>* typeset	
**Ethics Statement**<br><br>Enter an ethics statement for this submission. This statement is required if the study involved:<br><br>• Human participants<br>• Human specimens or tissue<br>• Vertebrate animals or cephalopods<br>• Vertebrate embryos or tissues<br>• Field research<br><br>Write "N/A" if the submission does not require an ethics statement.<br><br>General guidance is provided below. Consult the submission guidelines for detailed instructions. **Make sure that all information entered here is included in the Methods section of the manuscript.**	N/A

**Format for specific study types**<br><br>**Human Subject Research (involving human participants and/or tissue)**<br>• Give the name of the institutional review board or ethics committee that approved the study<br>• Include the approval number and/or a statement indicating approval of this research<br>• Indicate the form of consent obtained (written/oral) or the reason that consent was not obtained (e.g. the data were analyzed anonymously)<br><br>**Animal Research (involving vertebrate animals, embryos or tissues)**<br>• Provide the name of the Institutional Animal Care and Use Committee (IACUC) or other relevant ethics board that reviewed the study protocol, and indicate whether they approved this research or granted a formal waiver of ethical approval<br>• Include an approval number if one was obtained<br>• If the study involved *non-human primates*, add *additional details* about animal welfare and steps taken to ameliorate suffering<br>• If anesthesia, euthanasia, or any kind of animal sacrifice is part of the study, include briefly which substances and/or methods were applied<br><br>**Field Research**<br><br>Include the following details if this study involves the collection of plant, animal, or other materials from a natural setting:<br>• Field permit number<br>• Name of the institution or relevant body that granted permission	
**Data Availability**<br><br>Authors are required to make all data underlying the findings described fully available, without restriction, and from the time of publication. PLOS allows rare exceptions to address legal and ethical concerns. See the PLOS Data Policy and FAQ for detailed information.	Yes - all data are fully available without restriction

Powered by Editorial Manager® and ProduXion Manager® from Aries Systems Corporation

A Data Availability Statement describing where the data can be found is required at submission. Your answers to this question constitute the Data Availability Statement and **will be published in the article**, if accepted.<br><br>**Important:** Stating 'data available on request from the author' is not sufficient. If your data are only available upon request, select 'No' for the first question and explain your exceptional situation in the text box.<br><br>Do the authors confirm that all data underlying the findings described in their manuscript are fully available without restriction?	
**Describe where the data may be found in full sentences. If you are copying our sample text, replace any instances of XXX with the appropriate details.**<br><br>• If the data are **held or will be held in a public repository**, include URLs, accession numbers or DOIs. If this information will only be available after acceptance, indicate this by ticking the box below. For example: *All XXX files are available from the XXX database (accession number(s) XXX, XXX.)*.<br>• If the data are all contained **within the manuscript and/or Supporting Information files**, enter the following: *All relevant data are within the manuscript and its Supporting Information files.*<br>• If neither of these applies but you are able to provide **details of access elsewhere**, with or without limitations, please do so. For example:<br><br>*Data cannot be shared publicly because of [XXX]. Data are available from the XXX Institutional Data Access / Ethics Committee (contact via XXX) for researchers who meet the criteria for access to confidential data.*<br><br>*The data underlying the results presented in the study are available from (include the name of the third party*	All relevant data are within the manuscript and its Supporting Information files.

and contact information or URL).<br>• This text is appropriate if the data are owned by a third party and authors do not have permission to share the data.<br><br>* typeset	
Additional data availability information:	

Powered by Editorial Manager® and ProduXion Manager® from Aries Systems Corporation

Click here to access/download;Manuscript;Manuscript.docx

Comparison of Efficacy and Safety of Different Anticoagulation Regimens in Plasma Exchange: A Systematic Review and Meta-analysis

Song Ren[1], Liming Huang [1], Yi Li[1], and Yunlin Feng [1,*]

[1] Department of Nephrology, Sichuan Academy of Medical Sciences & Sichuan Provincial People's Hospital, University of Electronic Science and Technology of China, Chengdu 610072, China

* Correspondence:

Yunlin Feng, MD, Nephrology department, Sichuan Provincial People's Hospital, Chengdu, 610072, China.

Email: fengyunlin@med.uestc.edu.cn.

Abstract

Background Extracorporeal line clotting during plasma exchange (PE) not only delays efficient treatment, but also cause great waste of nursing resources. There is a lack of comprehensive comparison of the efficacy and safety among different anticoagulation regimens in plasma exchange in literature.

Methods A systematic search was performed in EMBASE, MEDLINE via PubMed, Cochrane Central Library, and CNKI. Studies that had compared at least two anticoagulation regimens in PE were considered eligible. The anticoagulative efficacy outcome was assessed by the occurrence of

extracorporeal circuit clotting. The safety outcome was assessed by the occurrence of bleeding events, post-treatment APTT values, and post-treatment platelets counts. The risk of bias was assessed by the AHRQ tool. Mean differences or standardized mean differences with 95% confidence intervals (CIs) of continuous variables and risk ratios (RRs) with 95% CIs of categorical variables were pooled using a random-effects or a fixed-effects model as appropriate.

Results In all, 7 studies with 1638 patients and 10951 sessions of PE treatment were included. Pooled results indicated the anticoagulative efficacy of UFH was better than that of saline flushing, yet did not differ with those of LMWH or RCA. Although the occurrence of bleeding events had no difference among different pairs of anticoagulation regimens, anticoagulation using UFH might lead to longer post-treatment APTT value and lower post-treatment platelet counts. Only one study was judged to have low risk of bias in each of the five domains in the AHRQ tool.

Conclusions The current anticoagulation regimens are generally effective and well tolerated in PE; however, the number of included studies was too limited to draw definitive conclusions.

Keywords: plasma exchange, anticoagulation, efficacy, safety, systematic review, meta-analysis

Introduction

Plasma exchange (PE) is a well-established mode of blood purification that is theoretically able to clear all undesired molecules in the plasma [1]. Its application has been extensive in the treatment of various conditions, including liver failure, kidney diseases, autoimmune disorders, neurological diseases, sepsis, and intoxication [2-5]. Adequate coagulation is a critical prerequisite to ensure the effective implementation of PE therapies, thus presenting an important factor to consider during such treatments. Insufficient anticoagulation may lead to premature failure of

treatment and great waste of nursing resources, whereas excessive anticoagulation bears high risk of bleeding.

Current pharmaceutical options for anticoagulation in PE include unfractionated heparin (UFH), low molecular weight heparin (LMWH), regional citrate acid (RCA), nafamostat, bivalirudin, and saline flushing [1]. Interestingly, we found in literature most PE treatments had used RCA, especially in Europe [6, 7]; however, in our own clinical practice, LMWH is the most commonly employed anticoagulation regimen, which has demonstrated satisfactory efficacy and safety outcomes. Notably, there is a lack of comprehensive comparison of the efficacy and safety among different anticoagulation regimens in PE in existing literature and no conclusion has been made about the best anticoagulation regimen.

Therefore, we conducted this systematic review and meta-analysis to evaluate the efficacy and safety of different anticoagulation regimens in PE, identify the potentially best regimen, and provide evidence for future development of relevant operation procedures.

Materials and Methods

Data sources and searches

We conducted a systematic search on 22nd March, 2023 according to the Preferred Reporting Items for Systematic Review and Meta-Analyses (PRISMA) statement [8] for eligible studies in the following electronic data resources without date restriction: EMBASE, MEDLINE via PubMed, Cochrane Central Library, and China National Knowledge Infrastructure (CNKI). The search terms were medical subject headings and text words relevant to PE and anticoagulation (Table S1). This study has been registered on PROSPERO (Identifier# CRD42023413640).

Study selection

Studies that had compared the outcomes of at least two anticoagulation regimens in PE were considered eligible for inclusion. Based on the preliminary screening experience, eligible studies were restricted to publications after 1990.

Two reviewers (R.S. and H.L.M.) independently conducted the review following a standardized approach. Duplications, non-original studies (e.g., reviews, editorial commentaries, protocols, and guidelines), studies published before 1990, case reports, non-human studies, pediatric studies, studies irrelevant to PE, studies on PE yet without reports on anticoagulation agents, and studies in neither English or Chinese were excluded after careful screening of titles and abstracts. Studies that had only used a single anticoagulation regimen or had not reported detailed information on coagulation outcomes to allow comparisons were also excluded. Reference lists from full text reviewed articles were further manually screened to identify any other relevant studies. Any discrepancy was adjudicated by a third reviewer (F.Y.L.).

Definitions of Outcomes

The efficacy outcome was assessed by the occurrence of extracorporeal circuit clotting. The safety outcome was assessed by the occurrence of bleeding events, post-treatment APTT values, and post-treatment platelets counts.

Data extraction

Two reviewers (R.S. and H.L.M.) independently extracted and compiled data from included studies after screening following a double-check procedure. Disagreements were resolved by the third reviewer (F.Y.L.). The data extracted included authors, year of publication, geographical origin, study duration, numbers of patients and procedures, indications for PE, treatment parameters, details of anticoagulation regimens, and details of studied outcomes. Information

about potential sources of significant clinical heterogeneity, such as age and gender composition of participants, was also collected for potential sensitivity analysis.

Critical appraisal

Since the included studies contained randomized, nonrandomized, and case-control designs, the study quality was independently assessed by two reviewers (R.S. and H.L.M.) based on the Agency for Healthcare Research and Quality (AHRQ) tool [9].

Data synthesis and analysis

Data synthesis used Review Manager software (Version 5.2; Cochrane, Oxford, UK). For continuous values, mean differences (MDs) or standardized mean differences (SMDs) with 95% confidence intervals (CIs) of studied outcomes between different paired groups were pooled using a random-effects or a fixed-effects model as appropriate. For categorical values, risk ratios (RRs) with 95% CIs of studied outcomes between different paired groups were pooled using a random-effects or a fixed-effects model as appropriate. Statistical heterogeneity was estimated using I^2 statistic [10]. The statistical heterogeneity of pooled outcomes was deemed as low if I^2 <25%, moderate if I^2 ranged from 26% to 75%, and high if I^2 >75%. The statistical significance was set at a two-sided $p < 0.05$. Funnel plot analysis for publication bias or sensitivity analysis were not performed due to the limited number of studies.

Results

Literature searching

5412 records were returned from literature searching after removing duplications. 5305 records excluded after title and abstract screening, leaving 107 records for full text review. After further excluding 100 studies due to having reported only one anticoagulation regimen or lacking

sufficient information to allow comparison, seven studies were finally included in this systematic review (Figure 1).

Study characteristics

In all, the seven studies involved 1638 patients and 10951 sessions of PE treatment (Table 1). Among these studies, five were respective observational case-control studies [11-15], one was a prospective nonrandomized trial [16], and one was a prospective randomized controlled trial [17]. The most common indication for PE was liver failure. Fresh frozen plasma was the most ultilized replacement fluid. Two studies had compared three anticoagulation regimens [11, 14], whereas the other five studies had compared two anticoagulation regimens [12, 13, 15-17] (Table 2). All seven studies had reported the outcomes of UFH. There were three studies each that had reported the outcomes of LMWH, RCA, and saline flushing, respectively. Outcomes of extracorporeal circuit clotting, bleeding, post-treatment APTT values, and post-treatment platelet counts had been reported in six [11-16], seven [11-17], five [12, 14-17], and four [12, 14, 15, 17] studies, respectively (Table S2).

Comparison of anticoagulative efficacy

Pooled results of the occurrence of extracorporeal circuit clotting indicated the anticoagulative efficacy of UFH was better than that of saline flushing (RR: 0.36, 95% CI: 0.28 to 0.46, $p<0.0001$; heterogeneity: $I^2=36\%$, $p=0.21$), yet did not differ with those of LMWH (RR: 2.73, 95% CI: 0.14 to 54.31, $p=0.51$; heterogeneity: $I^2=95\%$, $p<0.00001$) or RCA (RR: 1.28, 95% CI: 0.31 to 5.22, $p=0.73$; heterogeneity: $I^2=71\%$, $p=0.03$) (Figure 2).

Comparison of safety

The occurrence of bleeding events of UFH did not differ with those of RVA (RR: 2.14, 95% CI:

0.19 to 24.64, p=0.54; heterogeneity: I^2=52%, p=0.12), saline flushing (RR: 2.09, 95% CI: 0.68 to 6.42, p=0.20; heterogeneity: I^2=43%, p=0.17), or LMWH (RR: 4.30, 95% CI: 0.10 to 192.47, p=0.45; heterogeneity: I^2=90%, p<0.0001) (Figure 3). Pooled results indicated the post-treatment APTT value of UFH was consistently longer than those of RCA (SMD: 1.51s, 95% CI: 1.09s to 1.93s, p<0.001; heterogeneity: I^2=0%, p=0.62), saline flushing (SMD: 1.42s, 95% CI: 0.99s to 1.85s, p<0.001; heterogeneity: I^2=48%, p=0.17), and LMWH (SMD: 9.89s, 95% CI: 5.21s to 14.58s, p<0.001; heterogeneity: I^2=0%, p=0.71) (Figure 4). The pooled post-treatment platelet count of UFH was significantly less than that of LMWH (MD: $-25.45x10^9$/L, 95% CI: $-30.83x10^9$/L to $-20.07x10^9$/L, p<0.001; heterogeneity: I^2=0%, p=0.66), yet did not differ with that of saline flushing (MD: $-1.66x10^9$/L, 95% CI: $-6.30x10^9$/L to $2.99x10^9$/L, p<0.001; heterogeneity: I^2=0%, p=0.68) (Figure 5).

Critical appraisal

Only one study was judged to have low risk of bias in each of the five domains in the AHRQ tool [16], and four studies had domains with high risk of bias (Figure 6). The two domains with the highest proportions of high risk of bias were attrition bias and reporting bias (both 2/7, 28.6%).

Discussion

Pooled results of comparisons between different pairs of anticoagulation regimens in PE indicated the anticoagulative efficacy of each anticoagulation regimen did not differ among each other, yet consistently better than that of saline flushing. Although the occurrence of bleeding events had no difference, anticoagulation using UFH might lead to longer post-treatment APTT value and lower post-treatment platelet counts. Critical appraisal showed more than half of the studies had high risk of bias based on the AHRQ assessment. It should be noted that the number of included studies

was too limited to draw definitive conclusion on the best anticoagulation regimen in PE.

Anticoagulative drug is not the only determinant of anticoagulative efficacy in PE, which is influenced by multiple other factors including but not limited to filter membrane, blood flow rate, plasma separation speed, and replacement fluid speed [18]. It should bear in mind when interpret the findings of this study, the limited number of included studies precluded comparisons of anticoagulation regimens with the above cofounders adjusted. The choice of anticoagulation regimen is also influenced by the indications of PE, experience and preference of practitioners, and local operation procedures. For example, although RCA has been reported safe in patients with liver diseases [16, 19], we usually use UFH or LMWH in PE for liver failure patients in our local practice. Saline flushing is also used in patients with low platelet counts or coagulative disorders. In addition, the most commonly reported indication for PE in European countries such as Italy is neurological disease, which might partly explain why RCA is most often used [20-22].

Generally, all current anticoagulation regimens are well tolerated. UFH interacts with multiple targets in the coagulative cascade, including Factors IIa, IXa, Xa, XIa, and XIIa [23]. The anticoagulative effects of moderate and high dose of UFH can be monitored by APTT and ACT, respectively [23]. Although the pooled results showed the APTT and PLT values after treatment were worse in UFH anticoagulation settings, the occurrence of bleeding events did not differ among all anticoagulation regimens. Therefore, UFH did not exhibited apparent disadvantages in PE; however, its use should be carefully balanced in patients with pre-existing coagulative disorders and/or low PLT counts. These two clinical settings are commonly observed in patients with liver failure or thrombotic microangiopathy, which are both important indications for PE treatment. The growing utilization of novel anticoagulant agents, such as rivaroxaban, has the

potential to introduce new clinical scenarios for PE. For instance, patients with nephrotic syndrome who are receiving rivaroxaban may require PE treatment under specific clinical settings, such as during the outbreak of underlying autoimmune diseases. In such instances, rivaroxaban becomes a crucial consideration when prescribing anticoagulants for PE. Unfortunately, there is a lack of literature addressing this particular application. Future investigations are warranted to provide further insights into this area.

To the best of our acknowledgment, this is the first systematic review and meta-analysis on comparisons of different anticoagulation regimens in PE. Several limitations need to be acknowledged. Firstly, the limited number of studies included in this review precluded the ability to derive definitive conclusions, conduct sensitivity analysis, or analyze publication bias. It was also the reason that network meta-analysis was deemed unfeasible. Secondly, the majority of the included studies focused on liver failure populations, thereby failing to encompass the broader indications for PE. Lastly, the comparisons were unable to account for factors that might have influenced the observed anticoagulative outcomes beyond anticoagulative drugs, such as blood flow. More studies especially well-designed randomized controlled trials (RCTs) are needed for further investigations on the benefits and risks of different anticoagulation regimens in PE.

Conclusions

The findings of this study indicate the current anticoagulation regimens are generally effective and well-tolerated to ensure successful delivering of PE treatments. Although the occurrence of bleeding events had no difference, UFH anticoagulation might lead to longer post-treatment APTT value and lower post-treatment platelet counts. The number of included studies was too limited to draw definitive conclusion in this field. More studies especially well-designed RCTs are needed to

balance the benefits and risks of different anticoagulation regimens in PE.

Supplementary Materials: Table S1: Literature search strategies. Table S2: Reported outcomes of included studies.

Author Contributions: Conceptualization, F.Y.L.; methodology, R.S. and F.Y.L.; software, R.S. and H.L.M.; data curation and formal analysis, R.S., H.L.M., and F.Y.L.; investigation, R.S., F.Y.L., H.L.M., and L.Y.; writing—original draft preparation, R.S. and F.Y.L.; writing—review and editing, H.L.M., and L.Y.; project administration, F.Y.L.; funding acquisition, F.Y.L.. All authors have read and agreed to the published version of the manuscript.

Funding: This research was partly funded by Sichuan Province Science and Technology Support Program (23SYSX0132).

Institutional Review Board Statement: Not applicable.

Informed Consent Statement: Not applicable.

Data Availability Statement: The data analyzed or generated during the study is available from the corresponding author on reasonable request.

Conflicts of Interest: The authors declared no conflict of interest.

Acknowledgment: None.

References

[1] C.E. Cervantes, E.M. Bloch, C.J. Sperati, Therapeutic Plasma Exchange: Core Curriculum 2023, Am J Kidney Dis, (2023).

[2] A.P. Sanchez, D.M. Ward, R. Cunard, Therapeutic plasma exchange in the intensive care unit: Rationale, special considerations, and techniques for combined circuits, Ther Apher Dial, 26 Suppl 1 (2022) 41-52.

[3] R. Maiwall, S.K. Sarin, Plasma Exchange in Acute and Acute on Chronic Liver Failure, Seminars in Liver Disease, 41 (2021) 476-494.

[4] D. Ranganathan, G.T. John, Therapeutic plasma exchange in renal disorders, Indian journal of nephrology, 29 (2019) 151-159.
[5] E. Rimmer, B.L. Houston, A. Kumar, A.M. Abou-Setta, C. Friesen, J.C. Marshall, G. Rock, A.F. Turgeon, D.J. Cook, D.S. Houston, R. Zarychanski, The efficacy and safety of plasma exchange in patients with sepsis and septic shock: a systematic review and meta-analysis, Critical care (London, England), 18 (2014) 699.
[6] H. Kim, K.S. Han, National apheresis registry results for 2016 by the Korean society for apheresis, Transfusion, 57 (2017) 99A.
[7] B. Stegmayr, J. Ptak, B. Wikstrom, G. Berlin, C.G. Axelsson, A. Griskevicius, P. Centoni, G. Liumbruno, P. Molfettini, J. Audzijoniene, K. Mokvist, B.N. Sojka, R. Norda, F. Knutson, W. Ramlow, M. Blaha, V. Witt, M. Evergren, J. Tomaz, World apheresis registry 2003-2007 data, Transfusion and Apheresis Science, 39 (2008) 247-254.
[8] A. Liberati, D.G. Altman, J. Tetzlaff, C. Mulrow, P.C. Gotzsche, J.P. Ioannidis, M. Clarke, P.J. Devereaux, J. Kleijnen, D. Moher, The PRISMA statement for reporting systematic reviews and meta-analyses of studies that evaluate healthcare interventions: explanation and elaboration, BMJ, 339 (2009) b2700.
[9] R. Chou, N. Aronson, D. Atkins, A.S. Ismaila, P. Santaguida, D.H. Smith, E. Whitlock, T.J. Wilt, D. Moher, AHRQ series paper 4: assessing harms when comparing medical interventions: AHRQ and the effective health-care program, J Clin Epidemiol, 63 (2010) 502-512.
[10] J.P. Ioannidis, Interpretation of tests of heterogeneity and bias in meta-analysis, J Eval Clin Pract, 14 (2008) 951-957.
[11] B. Brunetta Gavranić, N. Bašić-Jukić, V. Premužić, P. Kes, Membrane therapeutic plasma exchange with and without heparin anticoagulation, J Clin Apher, 32 (2017) 479-485.
[12] O.H. Pan Ye, Zeng Jianyong, Wu Xiaolu, Liu Jiajun, Wei Jihong, Analysis of unfractionated heparin and low molecular weight heparin in plasma exchange, China Medical Engineering, 23 (2015) 2.
[13] S.P. Teh, Q.Y. Ho, Y.S.T. Kee, S. Thangaraju, R.Y. Tan, S.H. Teo, H.K. Tan, C.S. Tan, H.L.L. Choong, L.C. Ng, M. Abdul Rahman, A.E.L. Lim, M. Kaushik, Regional citrate anticoagulation vs systemic heparin anticoagulation for double-filtration plasmapheresis, J Clin Apher, 38 (2023) 16-23.
[14] F. Yuan, Z. Li, X. Li, H. Liu, Application of regional citrate anticoagulation in membrane therapeutic plasma exchange, International urology and nephrology, 52 (2020) 2379-2384.
[15] Z.C. Zhang Jie, Zheng Rongjiong, Deng Zerun, Sun Lihua, Yao Lei, Lu Xiaobo, Comparison of the efficay and safety of different anticoagulant drugs in the treatmetn of liver failure with dual bilirubin adsorption combined wit plasma exchange, Journal of Xingjiang Medical University, 45 (2022) 5.
[16] Y. Ma, F. Chen, Y. Xu, M. Wang, T. Zhou, J. Lu, P. Feng, Y. Wang, L. Bai, H. Tang, Safety and Efficacy of Regional Citrate Anticoagulation during Plasma Adsorption Plus Plasma Exchange Therapy for Patients with Acute-on-Chronic Liver Failure: A Pilot Study, Blood Purif, 48 (2019) 223-232.
[17] S. Yuan, Y. Qian, D. Tan, D. Mo, X. Li, Therapeutic plasma exchange: A prospective randomized trial to evaluate 2 strategies in patients with liver failure, Transfus Apher Sci, 57 (2018) 253-258.
[18] J. Kossler, Clinical use of therapeutic plasmapheresis, Transfusion Medicine and Hemotherapy, 43 (2016) 6.
[19] K.G. Khalikulov, Role of plasmapheresis in liver failure management, Hepatology International, 16 (2022) S124-S125.
[20] B. Naznin, T. Afroz, Outcomes of therapeutic plasma exchange; single tertiary center experience in Bangladesh, Vox Sanguinis, 115 (2020) 313.

[21] S. Korkmaz, S. Solmaz Medeni, F. Demirkan, S. Kalayoglu Besisik, S. Altay Dadin, G. Akgun Cagliyan, S. Kabukcu Hacioglu, I. Sari, D. Goren Sahin, M. Arat, S. Dagdas, G. Ozet, N. Kutlu, T. Karaagac Akyol, O.I. Ozcebe, H. Uskudar Teke, D. Kiper Unal, N. Guner, A. Tombak, H. Celik, I. Bay, I. Kiki, G. Ozgur, M.A. Erkurt, D. Ozatli, O. Meletli, S. Demircioglu, C. Demir, E. Kurtoglu, F. Vural, M. Tobu, A. Karakus, O. Ayyildiz, M.S. Dal, B. Afacan Ozturk, M. Albayrak, S. Ocakci, Z. Bolaman, M. Sonmez, V. Karakus, O. Gokmen Sevindik, I. Berber, M.H. Dogu, E. Gulturk, T. Ulas, B. Payzin, I. Kuku, S. Cagirgan, F. Altuntas, The Turkish experience with therapeutic plasma exchange: A national survey, Transfus Apher Sci, 58 (2019) 287-292.

[22] D. Delphine, K. Tarik, P. Laura, B. Aurele, B. Noemie, B. Vincent, L. Pascal, J. Boris, K. Kada, Plasma exchange in the intensive care unit: A 14 year retrospective audit, Annals of Intensive Care, 8 (2018).

[23] A. Kaplan, P. Raut, G. Totoe, S. Morgan, N.D. Zantek, Management of systemic unfractionated heparin anticoagulation during therapeutic plasma exchange, J Clin Apher, 31 (2016) 507-515.

[24] Reçber T, Haznedaroğlu İC, Çelebier M. Review on Characteristics and Analytical Methods of Rivaroxaban. Crit Rev Anal Chem. 2022;52(4):865-877

Table 1. Characteristics of included studies.

Author/Year	Region	Study Design	Study duration	Population, n	Age	Male, n	Targeted diseases	PE Parameters			
								BFV (ml/min)	Separation speed (ml/min)	Replacement fluid	Replacement fluid speed (ml/min)
Brunetta, 2017[11]	Croatia	Respective observation	1982 to 2014	1140	NR	476	60 conditions, including MG, TMA, SLE, GBS, MS, RPGN, intoxications, etc.	50-100	20-30	5% albumin either alone or combined with Ringer's solution or saline; FFP	20-30
Yuan, 2018 [17]	China	Prospective randomized trial	2012 to 2014	164	Median: 45	148	Liver failure	120-130	20-40	FFP	NR
Yuan, 2020[14]	China	Respective observation	2016 to 2017	85	Mean: 54.0	50	Autoimmune disease, liver dysfunction, renal transplantation	150	20	FFP	NR
Teh S, 2022[13]	Singapore	Retrospective cohort study	2018 to 2021	23	NR	NR	Kidney transplant recipients	120-250	NR	5% albumin; FFP or cryoprecipitate when needed	NR
Ma, 2019[16]	China	Prospective nonrandomized controlled trial	July to August, 2017	52	NR	41	HBV-ACLF	130	30	FFP	30
Zhang, 2022[15]	China	Respective observation	2020.09 to 2021.03	62	Mean: 50.0	42	Liver failure	80-110	20-50	FFP	NR
Pan, 2015[12]	China	Respective observation	2004.04 to 2014.01	112	Mean: 39.0	63	Liver Failure	80-180	20-35	FFP	NR

Abbreviations: ACLF, acute-on-chronic liver failure; BFV, blood flow velocity; FFP, fresh frozen plasma; GBS, Gillian-Barre syndrome; MG, myasthenia gravis; min, minutes; ml, milliliter; MS, multiple sclerosis; n, number; NR, not reported; PE, plasma exchange; RPGN, rapidly progressive glomerulonephritis; SD, standard deviation; SLE, systemic lupus erythematosus; TMA, thrombotic microangiopathy.

Table 2. Anticoagulation regimens and outcomes of included studies.

Author/Year	Procedures, n	UFH		LMWH		RCA		Saline flushing
		n*	Protocol	n*	Protocol	n*	Protocol	n*
Brunetta, 2017[11]	9611	7733	50 IU/kg+1000 IU/h	575	nadroparin: 65 IU/kg; enoxaparin: 100 IU/kg; daltaparin: 65 IU/kg; reviparin: 50 IU/kg	-	-	1193
Yuan, 2018 [17]	398	168	2500 IU+50 IU/h	-	-	-	-	230
Yuan, 2020[14]	255	120	40 IU/kg+625-1000 IU/h	-	-	93	170ml/h, adjusted to match post-filter iCa of 0.25-0.45 mmol/L	42
Teh S, 2022[13]	112	50	2000 IU+1000 IU/h or 500-1000 IU+250-500 IU/h	-	-	62	120-150 ml/h, adjusted to match post-filter iCa of 0.25-0.35 mmol/L	-
Ma, 2019[16]	120	94	3125 IU+500 IU/h	-	-	106	100 ml/h	-
Zhang, 2022[15]	83	62	NR	21	NR	-	-	-
Pan, 2015[12]	372	108	NR	264	NR	-	-	-

* number of procedures.

Note: "-" represents treatment regimens that were not included in the study.

Abbreviations: iCa, ionized calcium; kg, kilogram; L, liter; LMWH, low molecular weight heparin; n, number; NR, not reported; RCA, regional citrate acid; UFH, unfractionated heparin.

Figure Legends

Figure 1. PRISMA flow chart of this systematic review.

Figure 2. Comparisons of the effects of different anticoagulation regimens on extracorporeal circuit clotting.

Figure 3. Comparisons of the effects of different anticoagulation regimens on bleeding events.

Figure 4. Comparisons of the effects of different anticoagulation regimens on post-treatment APTT.

Figure 5. Comparisons of the effects of different anticoagulation regimens on post-treatment platelet counts.

Figure 6. Quality assessment results of included studies based on the AHRQ tool.

Click here to access/download;Figure;Figure 1.jpg

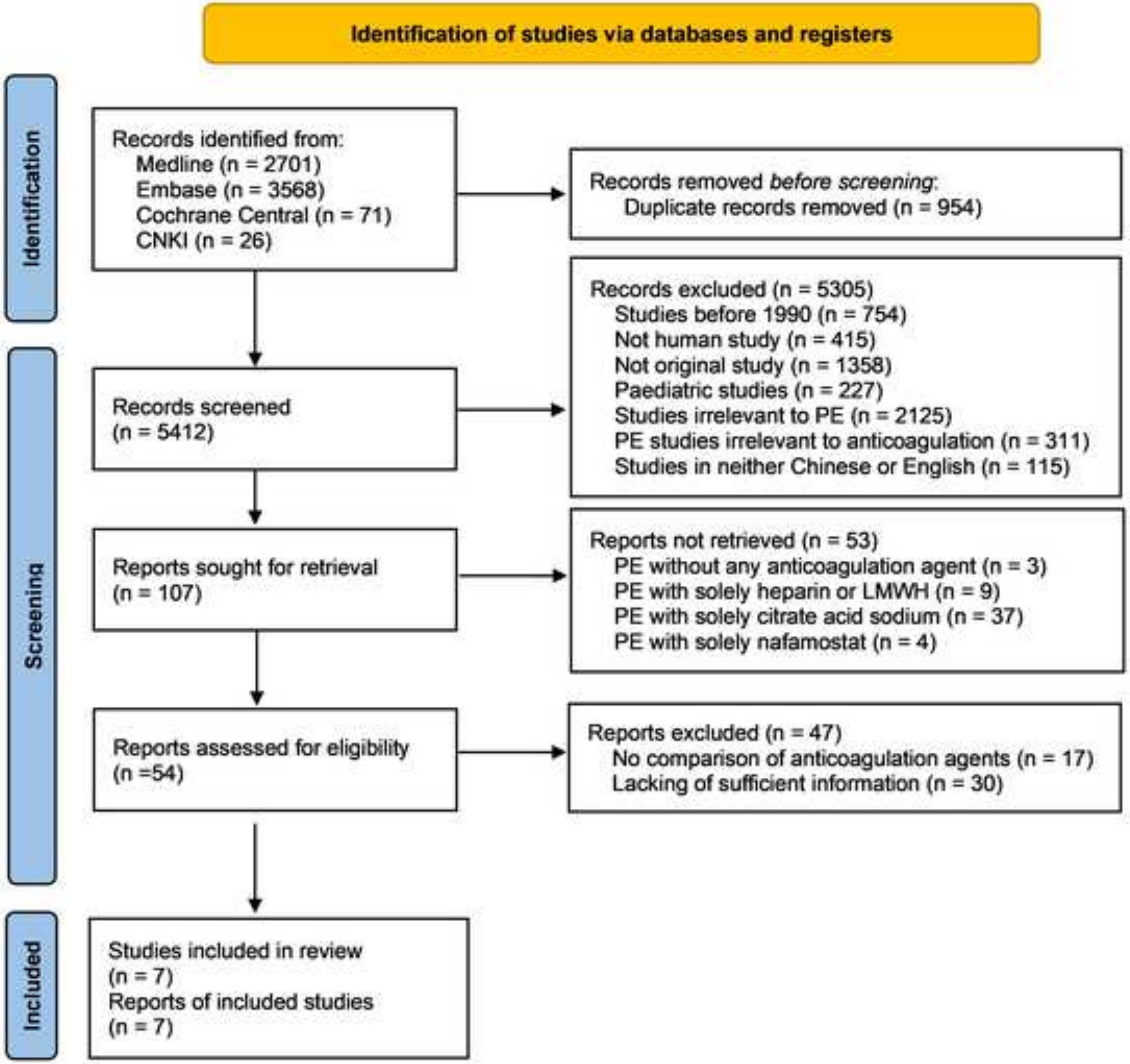

Click here to access/download;Figure;Figure 2.jpg

(A) Comparison of the effects of UFH and RCA on extracorporeal circuit clotting

	UFH		RCA			Risk Ratio
Study or Subgroup	Events	Total	Events	Total	Weight	M-H, Random, 95% CI
Ma Y 2019	20	94	16	106	46.7%	1.41 [0.78, 2.56]
Teh S 2023	3	50	10	62	36.6%	0.37 [0.11, 1.28]
Yuan F2020	9	120	0	93	16.6%	14.76 [0.87, 250.38]
**Total (95% CI)**		**264**		**261**	**100.0%**	**1.28 [0.31, 5.22]**
Total events	32		26			

Heterogeneity: Tau² = 1.01; Chi² = 6.92, df = 2 (P = 0.03); I² = 71%
Test for overall effect: Z = 0.34 (P = 0.73)

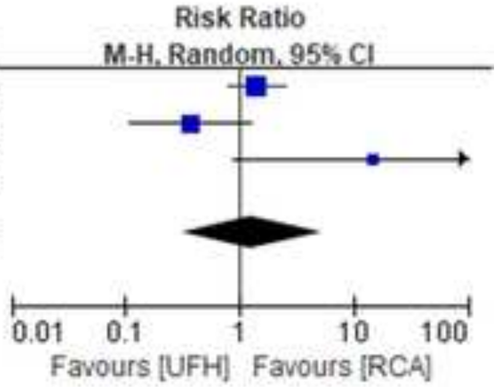

(B) Comparison of the effects of UFH and saline flushing on extracorporeal circuit clotting

	UFH		Saline			Risk Ratio
Study or Subgroup	Events	Total	Events	Total	Weight	M-H, Fixed, 95% CI
Brunetta 2017	183	7733	75	1193	86.2%	0.38 [0.29, 0.49]
Yuan F2020	9	120	14	42	13.8%	0.23 [0.11, 0.48]
**Total (95% CI)**		**7853**		**1235**	**100.0%**	**0.36 [0.28, 0.46]**
Total events	192		89			

Heterogeneity: Chi² = 1.57, df = 1 (P = 0.21); I² = 36%
Test for overall effect: Z = 8.22 (P < 0.00001)

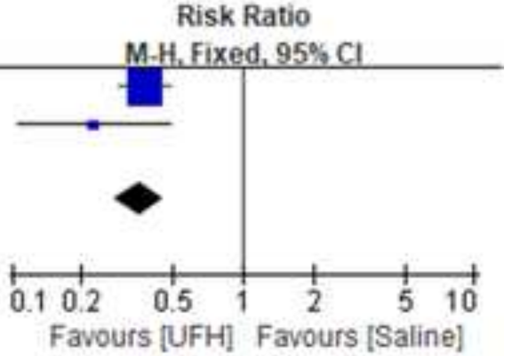

(C) Comparison of the effects of UFH and LMWH on extracorporeal circuit clotting

	UFH		LMWH			Risk Ratio
Study or Subgroup	Events	Total	Events	Total	Weight	M-H, Random, 95% CI
Brunetta 2017	183	7733	69	1193	37.1%	0.41 [0.31, 0.54]
Pan Y2015	19	108	3	264	35.1%	15.48 [4.68, 51.24]
Zhang J2022	5	62	0	21	27.8%	3.84 [0.22, 66.68]
**Total (95% CI)**		**7903**		**1478**	**100.0%**	**2.73 [0.14, 54.31]**
Total events	207		72			

Heterogeneity: Tau² = 6.26; Chi² = 39.50, df = 2 (P < 0.00001); I² = 95%
Test for overall effect: Z = 0.66 (P = 0.51)

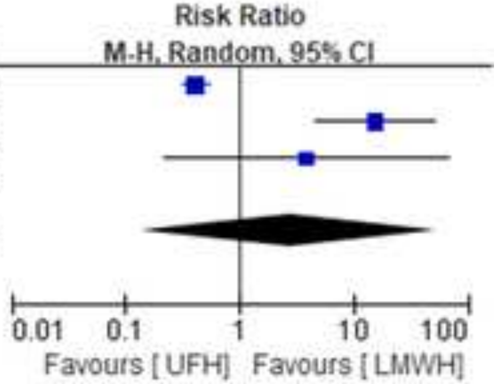

Click here to access/download;Figure;Figure 3.jpg

(A) Comparison of the effects of UFH and RCA on bleeding events

Study or Subgroup	UFH Events	UFH Total	RCA Events	RCA Total	Weight	Risk Ratio M-H, Random, 95% CI
Ma Y 2019	3	94	0	106	33.1%	7.88 [0.41, 150.68]
Teh S 2023	0	50	3	62	33.3%	0.18 [0.01, 3.34]
Yuan F2020	4	120	0	93	33.6%	6.99 [0.38, 128.25]
**Total (95% CI)**		**264**		**261**	**100.0%**	**2.14 [0.19, 24.64]**
Total events	7		3			

Heterogeneity: Tau^2 = 2.42; Chi^2 = 4.16, df = 2 (P = 0.12); I^2 = 52%
Test for overall effect: Z = 0.61 (P = 0.54)

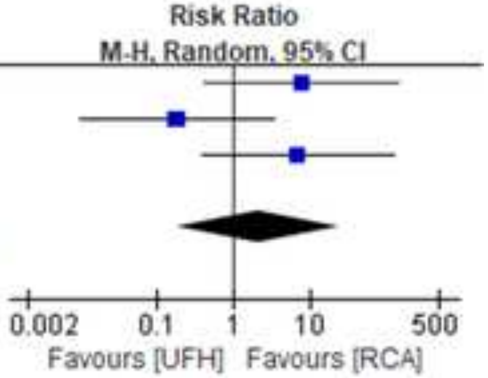

(B) Comparison of the effects of UFH and saline flushing on bleeding events

Study or Subgroup	UFH Events	UFH Total	Saline Events	Saline Total	Weight	Risk Ratio M-H, Random, 95% CI
Brunetta 2017	9	7733	2	1193	31.3%	0.69 [0.15, 3.21]
Yuan F2020	4	120	0	42	12.5%	3.20 [0.18, 58.18]
Yuan S2018	23	168	9	230	56.2%	3.50 [1.66, 7.37]
**Total (95% CI)**		**8021**		**1465**	**100.0%**	**2.09 [0.68, 6.42]**
Total events	36		11			

Heterogeneity: Tau^2 = 0.44; Chi^2 = 3.49, df = 2 (P = 0.17); I^2 = 43%
Test for overall effect: Z = 1.28 (P = 0.20)

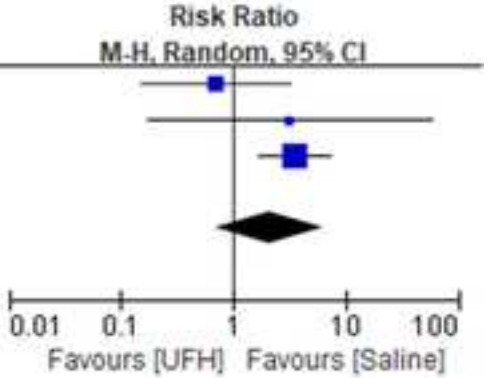

(C) Comparison of the effects of UFH and LMWH on bleeding events

Study or Subgroup	UFH Events	UFH Total	LMWH Events	LMWH Total	Weight	Risk Ratio M-H, Random, 95% CI
Brunetta 2017	9	7733	2	1193	35.4%	0.69 [0.15, 3.21]
Pan Y2015	32	108	0	264	31.3%	158.03 [9.76, 2557.79]
Zhang J2022	3	62	1	21	33.3%	1.02 [0.11, 9.25]
**Total (95% CI)**		**7903**		**1478**	**100.0%**	**4.30 [0.10, 192.47]**
Total events	44		3			

Heterogeneity: Tau^2 = 10.01; Chi^2 = 19.09, df = 2 (P < 0.0001); I^2 = 90%
Test for overall effect: Z = 0.75 (P = 0.45)

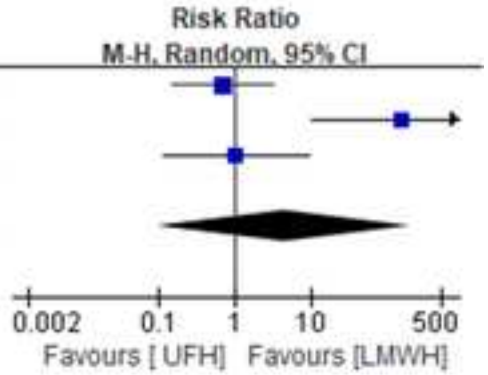

Click here to access/download;Figure;Figure 4.jpg

(A) Comparison of the effects of UFH and RCA on APTT

Study or Subgroup	UFH Mean	UFH SD	UFH Total	RCA Mean	RCA SD	RCA Total	Weight	Std. Mean Difference IV, Random, 95% CI
Ma Y 2019	162.7	27.2	24	122.5	29.3	28	46.3%	1.40 [0.78, 2.01]
Yuan F2020	48.7	3.6	34	40.1	6.7	30	53.7%	1.61 [1.04, 2.18]
**Total (95% CI)**			**58**			**58**	**100.0%**	**1.51 [1.09, 1.93]**

Heterogeneity: Tau² = 0.00; Chi² = 0.25, df = 1 (P = 0.62); I² = 0%
Test for overall effect: Z = 7.10 (P < 0.00001)

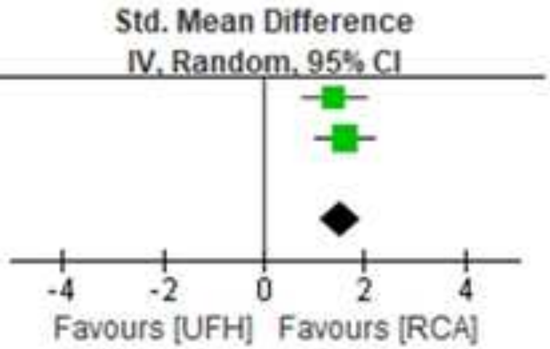

(B) Comparison of the effects of UFH and saline flushing on APTT

Study or Subgroup	UFH Mean	UFH SD	UFH Total	Saline Mean	Saline SD	Saline Total	Weight	Std. Mean Difference IV, Random, 95% CI
Yuan F2020	48.7	3.6	34	41	5.3	21	30.1%	1.76 [1.11, 2.40]
Yuan S2018	104.1	41.3	164	60.6	24.8	164	69.9%	1.27 [1.04, 1.51]
**Total (95% CI)**			**198**			**185**	**100.0%**	**1.42 [0.99, 1.85]**

Heterogeneity: Tau² = 0.06; Chi² = 1.91, df = 1 (P = 0.17); I² = 48%
Test for overall effect: Z = 6.41 (P < 0.00001)

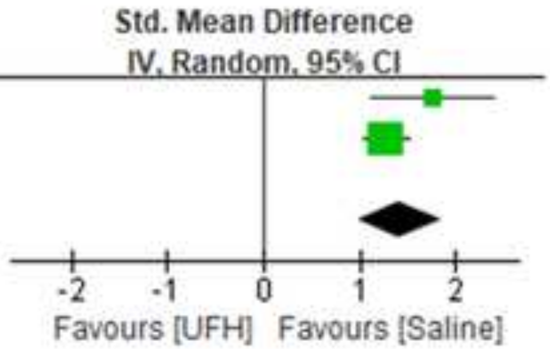

(C) Comparison of the effects of UFH and LMWH on APTT

Study or Subgroup	UFH Mean	UFH SD	UFH Total	LMWH Mean	LMWH SD	LMWH Total	Weight	Mean Difference IV, Random, 95% CI
Pan Y2015	71.67	22.47	108	62.15	23.32	264	84.9%	9.52 [4.43, 14.61]
Zhang J2022	61.37	38.74	62	49.38	16.93	21	15.1%	11.99 [-0.07, 24.05]
**Total (95% CI)**			**170**			**285**	**100.0%**	**9.89 [5.21, 14.58]**

Heterogeneity: Tau² = 0.00; Chi² = 0.14, df = 1 (P = 0.71); I² = 0%
Test for overall effect: Z = 4.14 (P < 0.0001)

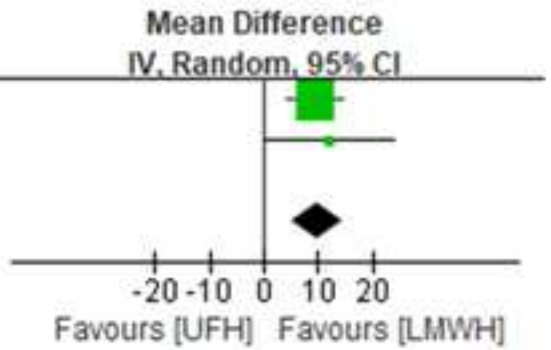

Click here to access/download;Figure;Figure 5.jpg

(A) Comparison of the effects of UFH and saline flushing on platelet counts

	UFH			Saline				Mean Difference
Study or Subgroup	Mean	SD	Total	Mean	SD	Total	Weight	IV, Fixed, 95% CI
Yuan F2020	98	11.2	34	99	9.7	21	68.7%	-1.00 [-6.60, 4.60]
Yuan S2018	81.8	45.7	164	84.9	29.2	164	31.3%	-3.10 [-11.40, 5.20]
**Total (95% CI)**			**198**			**185**	**100.0%**	**-1.66 [-6.30, 2.99]**

Heterogeneity: Chi² = 0.17, df = 1 (P = 0.68); I² = 0%
Test for overall effect: Z = 0.70 (P = 0.48)

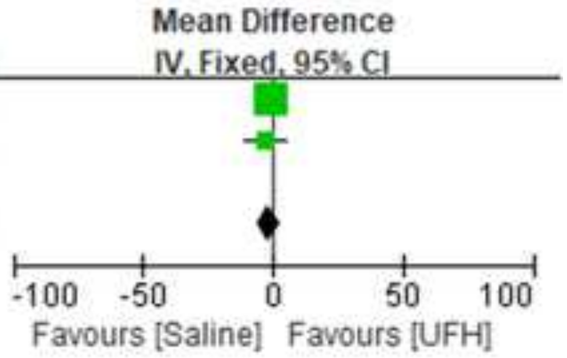

(B) Comparison of the effects of UFH and LMWH on platelet counts

	UFH			LMWH				Mean Difference
Study or Subgroup	Mean	SD	Total	Mean	SD	Total	Weight	IV, Fixed, 95% CI
Pan Y2015	88.76	26.7	108	114.4	17.2	264	97.5%	-25.64 [-31.09, -20.19]
Zhang J2022	110.58	57.06	62	128.42	72.83	21	2.5%	-17.84 [-52.07, 16.39]
**Total (95% CI)**			**170**			**285**	**100.0%**	**-25.45 [-30.83, -20.07]**

Heterogeneity: Chi² = 0.19, df = 1 (P = 0.66); I² = 0%
Test for overall effect: Z = 9.27 (P < 0.00001)

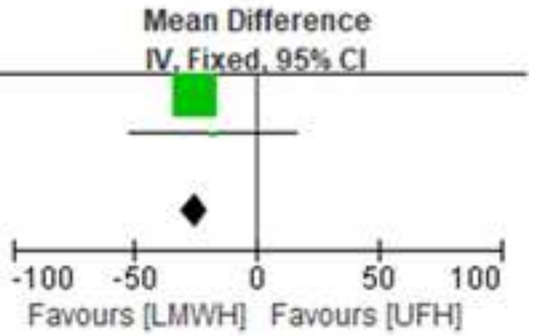

Click here to access/download;Figure;Figure 6.jpg

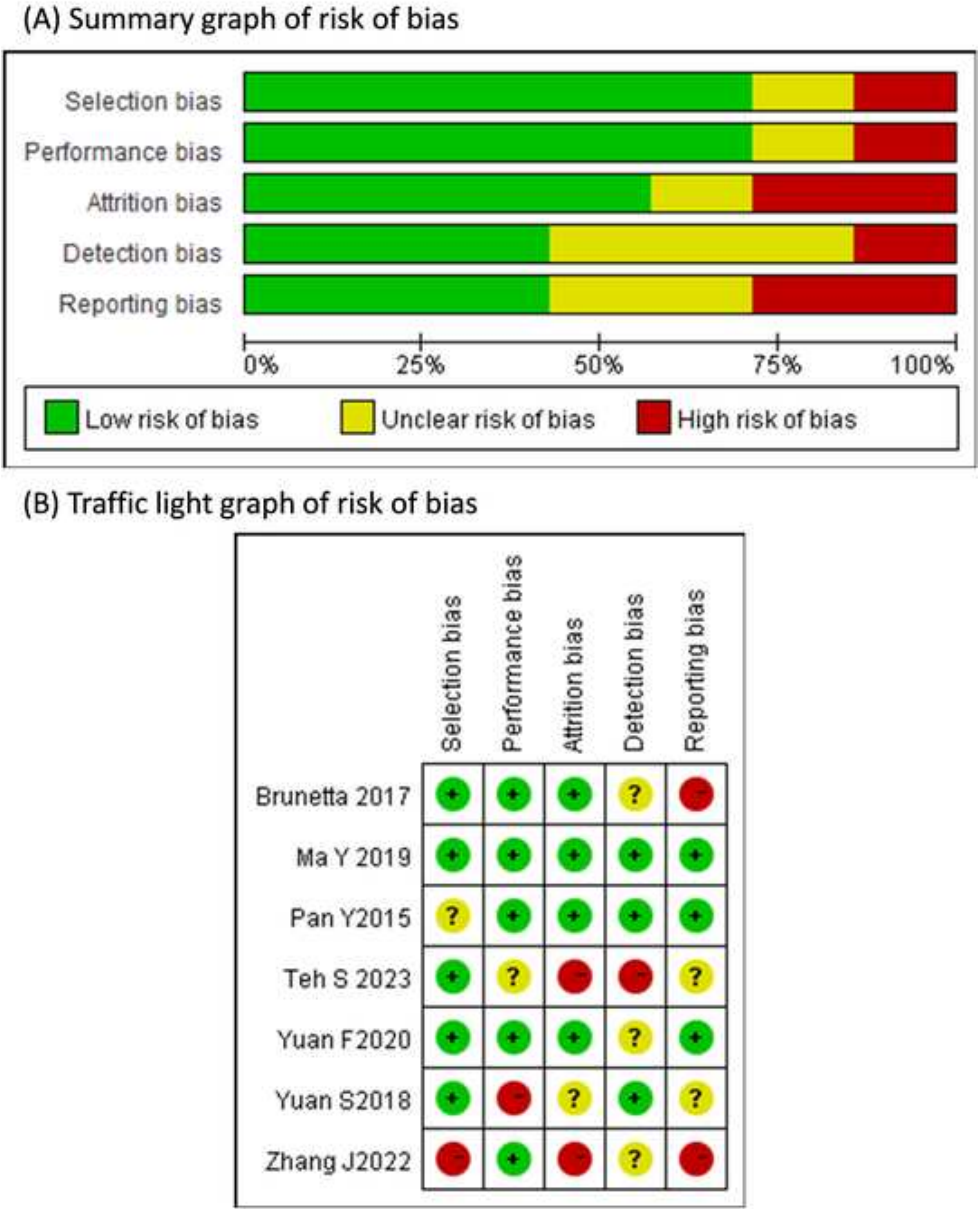

Click here to access/download
Supporting Information
PRISMA_2020_checklist.docx

Click here to access/download
Supporting Information
Supplementary Materials.docx
