# Supplementary material for: Comparison of efficacy and safety of different anticoagulation regimens in plasma exchange: A systematic review and meta-analysis
Source: PLoS One. 2024 Oct 24;19(10):e0311603. doi: 10.1371/journal.pone.0311603 (PMC11500872; doi:10.1371/journal.pone.0311603)
Supplement: Attachment — Submitted filename: Revision2 - Response.docx [file pone.0311603.s007.docx]

Dear Editor and Reviewers,

Thank you very much for the comments and the recognitions of our work. On behalf of all authors, I am submitting the revised manuscript with tracked changes for your further assessment. Please find our point-to-point response below to address the reviewers’ concerns. All the changes have been highlighted in yellow in the revised manuscript. We hope that you find our responses and modifications satisfactory.

Reviewer #3:

The manuscript "Comparison of Efficacy and Safety of Different Anticoagulation Regimens in Plasma Exchange: A Systematic Review and Meta-analysis" is technically sound, with appropriately performed and rigorously reported statistical analyses. The data support the conclusions, demonstrating that UFH is more effective than saline flushing and comparable to LMWH and RCA in anticoagulative efficacy. However, all underlying data is not made fully available, which is required by the PLOS Data Policy. They should ensure that all relevant data are provided within the manuscript, its supporting information, or deposited in a public repository. Additionally, given the complexity of the statistical methods, an additional review by a statistician is recommended to ensure accuracy. The manuscript addresses a critical clinical issue and offers valuable insights, making it suitable for highlighting on the PLOS ONE website to promote further research and discussion. There are no concerns about dual publication, research ethics, or publication ethics noted.

Thank you very much for the comment. The key data which we used for the data analysis are listed in Table 1 and Table 2 in the manuscript. To meet the requirement by PLOS Data Policy, all raw data required to replicate the results of this study are available in the Tables 1 and 2 in the manuscript. We have also made corresponding corrections in the “Data Availability” section in the manuscript which has been highlighted in yellow. We have also invited Mr. Qiang Li to review the data analysis process. Qiang is a senior statistician from The George Institute for Global Health in Sydney, Australia. He is an expert in medical statistic, has led statistical analysis in many international clinical studies, and is highly experienced. Based on Qiang’s assessment, we did not made change to the current results. Thank you very much for your help in this process.
